# High APLN Expression Predicts Poor Prognosis for Glioma Patients

**DOI:** 10.1155/2022/8393336

**Published:** 2022-09-22

**Authors:** Shuangyu Lv, Yang An, Huan Dong, Longxiang Xie, Hong Zheng, Xiaoxia Cheng, Lei Zhang, Tieshan Teng, Qiang Wang, Zhongyi Yan, Xiangqian Guo

**Affiliations:** ^1^Institute of Molecular Medicine, Department of Preventive Medicine, Cell Signal Transduction Laboratory, Bioinformatics Center, Henan Provincial Engineering Center for Tumor Molecular Medicine, Academy for Advanced Interdisciplinary Studies, School of Basic Medical Sciences, Henan University, Kaifeng 475004, China; ^2^School of Software, Henan University, Kaifeng 475004, China

## Abstract

Apelin (APLN) is an endogenous ligand of the G protein-coupled receptor APJ (APLNR). APLN/APLNR system was involved in a variety of pathological and physiological functions, such as tumorigenesis and development. However, its prognostic roles in patients with central nervous system (CNS) cancers remain unknown. The present study was designed to explore the expression profile, prognostic significance, and interaction network of APLN/APLNR by integrating data from Oncomine, GEPIA, LOGpc, STRING, GeneMANIA, and immunohistochemical staining. The results demonstrated that APLN and APLNR mRNA expression were significantly increased in CNS cancers, including both low-grade glioma (LGG) and glioblastoma (GBM), when compared with normal CNS tissues. The high APLN, but not APLNR, expression was significantly correlated with overall survival (OS), recurrence free survival (RFS), and progression free survival (PFS) of LGG patients. However, neither APLN nor APLNR expression was significantly related to prognostic value in terms of OS, disease free interval (DFI), disease specific survival (DSS), or progression free interval (PFI) for GBM patients. Additionally, immunohistochemistry staining confirmed the increased APLN expression in tissues of LGG patients with grade II than grade I. These results showed that an elevated APLN level could predict poor OS, RFS, and PFS for LGG patients, and it could be a promising prognostic biomarker for LGG.

## 1. Introduction

Apelin (APLN) was identified as a peptide ligand for APJ, named apelin receptor (APLNR). The human APLN is located on chromosome X at locus Xq25-q26.1, containing three exons and two introns. APLN gene encodes a secreted 77-amino acid precursor protein named preproapelin, and its amino acid sequences are conserved among human, cattle, mouse, and rat [1]. Preproapelin, following enzymatic cleavage, could be processed into different bioactive peptides, including apelin-12, apelin-13, apelin-17, and apelin-36 [2]. Human APLNR is a member of seven-transmembrane G protein-coupled receptors, consisting of 380 amino acid residues. APLN/APLNR mRNA and protein were detected in the central nervous system (CNS) and the peripheral human tissues, such as callosum, cortex, hippocampus, heart, lung, kidney, stomach [3, 4]. APLN/APLNR system was involved in a wide range of pathological and physiological processes, including cardiovascular function [5, 6], energy metabolism [7, 8], obesity [6], endocrine activity [9], ischemia/reperfusion injury [10], liver disease [11], and neuropathy [12].

Recently, APLN/APLNR was shown to be involved in several kinds of cancers, such as lung cancer, gastroesophageal cancer, colonic cancer, and hepatocellular carcinoma [13]. In non-small-cell lung cancer (NSCLC), APLN mRNA was significantly upregulated in tumor tissue compared with normal lung, and high level of APLN protein was reported to be associated with the increased microvessel densities and worse overall survival [14]. In patients with hepatocellular carcinoma, APLN mRNA was markedly higher in tumors than in nontumor tissues [15]. APLN/APLNR mRNA and protein expression in ovarian epithelial cancer and/or granulosa tumor cell lines were significantly higher than those of noncancer ovarian cell lines [16]. In patients with cholangiocarcinoma, APLN and APLNR genes were obviously upregulated in tumor tissues compare to nonmalignant liver tissues [17]. Kälin et al. found that APLN/APLNR mRNAs were upregulated within microvascular proliferations in glioblastoma specimens, compared with normal CNS tissues [18].

Glioma is the most common primary malignant brain tumor in adults, with 5-year survival of 20%-30% [19]. Glioma is subclassified and graded from I to IV according to histological criteria described by the World Health Organization (WHO) [20]. Low-grade glioma (LGG, grade I-II) is considered as infiltrative neoplasm that most frequently occurs in the cerebral hemispheres of adults [20]. Glioblastoma (GBM, grade IV) represents the most aggressive subtype of gliomas. Traditional histopathological methods do not adequately predict clinical outcomes of gliomas [21]. Therefore, the prognostic biomarkers are needed to predict and improve the clinical decision-making process. The prognosis of APLN and APLNR in gliomas remains unknown. The current study was designed to examine the prognostic values, gene expression profile, and interaction network of APLN/APLNR in LGG and GBM.

## 2. Materials and Methods

### 2.1. Oncomine Analysis

Oncomine (https://www.Oncomine.org) is an online gene expression array database with publicly accessible services. The database, consisting of 715 datasets and 86733 normal and tumor samples, provides genome-wide expression analyses for researchers [22, 23]. The normal samples were from normal human tissues. The thresholds (*p* value ≤0.05, fold-change ≥2, gene rank ≤10%, and data type: mRNA) were set, and the APLN and APLNR genes in gliomas and normal tissues were compared and analyzed using Oncomine.

### 2.2. GEPIA Analysis

Gene Expression Profiling Interactive Analysis (GEPIA, (http://gepia.cancer-pku.cn) is a web-based tool used for gene expression analysis based on the TCGA and the GTEx databases. GEPIA, containing 9736 tumors and 8587 normal samples, provides key interactive and customizable functions, including differential expression analysis, profiling plotting, and correlation analysis [24]. The normal samples were provided by normal human tissues. We examined the expression of APLN/APLR in LGG and GBM cancers by GEPIA.

### 2.3. Prognosis Analysis

The prognostic value of the APLN and APLNR in LGG and GBM patients was evaluated using LOGpc (Long-term Outcome and Gene Expression Profiling Database of pan-cancers) as we previously reported [25–32]. LOGpc (http://bioinfo.henu.edu.cn/DatabaseList.jsp) is a free online survival analysis web server to analyze the prognosis of a given gene in cancers. LOGpc contains 197 expression datasets, provides 13 types of survival terms for 28098 patients of 26 distinct malignancies. In this study, the prognostic values, including overall survival (OS), recurrence free survival (RFS), progression free survival (PFS), disease free interval (DFI), disease specific survival (DSS), and progression free interval (PFI), were evaluated by Kaplan-Meier (KM) survival plot with hazard ratio (HR) and log-rank test using LOGpc based on TCGA dataset of 525 LGG patients and GSE107850 dataset of 195 LGG patients and 6 datasets (TCGA, CGGAarray, CGGAseq, GSE42669, GSE7696, and GSE30472) of 684 GBM patients.

### 2.4. GeneMANIA and STRING Analysis

GeneMANIA (http://genemania.org) is a web tool for generating hypotheses about gene function, analyzing gene lists and prioritizing genes for functional assays [33, 34]. Analysis of APLN correlated genes was performed using GeneMANIA. Furthermore, we examined the protein interactions of APLN using STRING. The STRING database (http://string-db.org) aims to search for protein–protein interactions (PPI), including physical as well as functional associations. The latest version of STRING includes 5090 organisms, 24584628 proteins, and 3123056667 total interactions [35].

### 2.5. Immunohistochemical Staining and Evaluation

Tissue microarray, purchased from Wuhan Servicebio Technology Co., Ltd. (Wuhan, China), was routinely dewaxed, rehydrated, and immunohistochemistry staining was carried out following the standard procedure. The anti-apelin-36 (1 : 150 dilution, Phoenix Biotech Co., Ltd., Beijing, China) was used as primary antibody. The secondary antibody (HRP-labeled antirabbit antibody, 1 : 200 dilution, Servicebio Co., Ltd., Wuhan, China) was incubated with the sections, and the staining was visualized using diaminobenzidine (DAB) solution and counterstained with hematoxylin. PBS, instead of the primary antibody, was utilized as a negative control for excluding unspecific binding of secondary antibody. The protocol was approved by the Committee of Medical Ethics and Welfare, Henan University, School of Medicine.

The immunohistochemistry staining results were independently evaluated by two pathologists blinded to patient clinicopathologic parameters. APLN positivity was identified following the modified H-Score [36], including both intensity and percentage of tumor cell staining. The staining intensities of the APLN were scored from 0 to 3: 0-negative, 1-weak, 2-moderate, and 3-strong. The percentages of positively staining were scored from 0 to 4: 1 (0–25%), 2 (26%–50%), 3 (51%–75%), and 4 (76%–100%). The total score was calculated by multiplying the score of proportion and intensity, with a range from 0 to 12.

### 2.6. Statistical Analysis

Kaplan-Meier curves of OS, DFI, DSS, PFI, and RFS were constructed by setting the quarter (upper 25% vs lower 75%) of APLN/APLNR expression as the cut-off, while Kaplan-Meier curves of PFS were constructed by setting the median (upper 50% vs lower 50%) as the cut-off. Univariate and multivariate Cox regression analyses were performed to evaluate the prognostic value of APLN in terms of OS, RFS, and PFS for LGG patients using SPSS 18.0. Risk factors (*p* < 0.2) assessed by univariate analysis were utilized for the subsequent multivariate analysis. Kaplan-Meier curves of PFS, subgrouped by different IDH status and treatment therapies in GSE107875 dataset, were performed by GraphPad Prism 8.0, followingly a log-rank test was used to evaluate the difference between different subgroups. For immunohistochemistry analysis, the statistical difference of APLN expression between two groups was evaluated by Student's *t*-test. *p* < 0.05 was considered statistically significant.

## 3. Results

### 3.1. APLN and APLNR Were Upregulated in Gliomas

**Figure 1:**
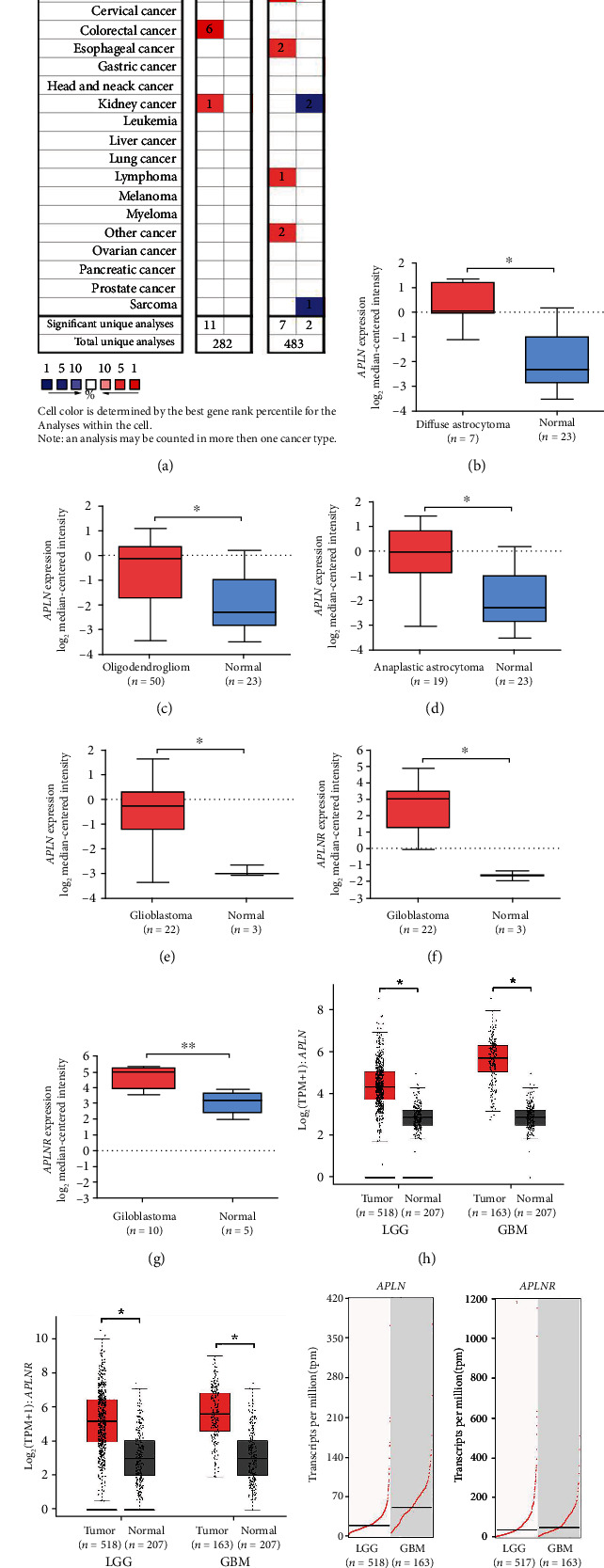
APLN and APLNR gene expression in central nervous system cancer tissue and normal tissue. (a) Summary of APLN/APLNR expression in various tumor types. The gene expression was evaluated using Oncomine database. The red square represents overexpression, and the blue square represents reduced expression in cancers compared to normal tissues. Box plots from Oncomine database show the expression of APLN gene in normal tissues and central nervous system cancer tissues, including diffuse astrocytoma (b), oligodendroglioma (c), anaplastic astrocytoma (d), and glioblastoma (e). APLNR gene expression in normal tissue and glioblastoma from Lee Brain dataset (f) and TCGA-brain dataset (g). APLN (h) and APLNR (i) gene expression in LGG/GBM tissue and normal tissue. Box plot was derived from Gene Expression Profiling Interactive Analysis (GEPIA). (j) Comparison of APLN/APLNR gene expression in LGG/GBM patients. The expression plot was originated from GEPIA. GBM: glioblastoma; LGG: low-grade glioma; ^∗^*p* < 0.01.

We compared the mRNA levels of APLN and APLNR in gliomas with ones in normal tissues by Oncomine database (Figures 1(a)–1(g)). In pan-tumor tissues, APLN was significantly increased in 15 datasets, while 3 datasets showed a decreased level. For APLNR, 11 datasets exhibited elevated level, whereas 6 datasets displayed reduced level in tumor tissues (Figure 1(a)).

For brain and CNS cancer, Sun Brain dataset [37] demonstrated that APLN is significantly increased in tissues of diffuse astrocytoma (fold change = 4.890, *p* = 2.83*E* − 5, Figure 1(b)), oligodendroglioma (fold change = 2.723, *p* = 1.66*E* − 6, Figure 1(c)), and anaplastic astrocytoma (fold change = 3.377, *p* = 3.48*E* − 5, Figure 1(d)), compared with corresponding normal brain tissues. Lee Brain dataset [38] indicated that both APLN and APLNR are significantly elevated in GBM tissue compared with normal neural stem cell (APLN: fold change = 5.632, *p* = 2.42*E* − 8, Figure 1(e); APLNR: fold change = 18.051, *p* = 4.14*E* − 10, Figure 1(f)). TCGA-Brain dataset [39] demonstrated that APLNR expression in GBM tissues were significantly higher than normal brain tissues (fold change = 3.131, *p* = 2*E* − 3, Figure 1(g)).

As showed in Figure 1(h), GEPIA analysis demonstrated that the mRNA expression level of APLN was significantly higher in LGG and GBM tissues than normal brain tissues (each *p* < 0.05). Likewise, the transcriptional expression level of APLNR displayed a significant upregulation in LGG or GBM tissues, compared with normal brain tissues (each *p* < 0.05, Figure 1(i)). In addition, we found that APLN and APLNR mRNA level in LGG patients was lower than that in GBM patients (Figure 1(j)).

### 3.2. Elevated APLN (Not APLNR) Expression Was Correlated with Worse OS, RFS, and PFS in LGG Patients

**Figure 2:**
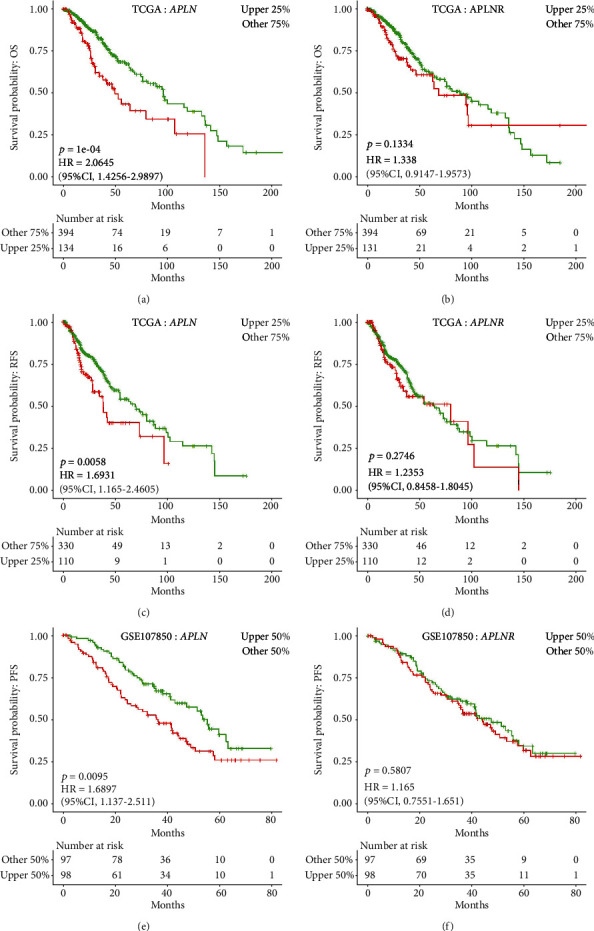
Kaplan–Meier plots measure the correlation between APLN/APLNR and survival outcomes in LGG. Kaplan-Meier curves of overall survival (OS, (a), b), recurrence free survival (RFS, c, d), and progression free survival (PFS, (e), f) for APLN/APLNR gene from TCGA and GSE107850 data in LGG patients.

Based on TCGA data in LOGpc, the prognosis analysis demonstrated that LGG patients with high APLN expression had inferior OS (*p* = 1*E* − 4; HR: 2.0645; 95% CI: 1.4256-2.9897; Figure 2(a)) in comparison to patients who had low APLN expression, whereas this association was not present for APLNR gene (*p* = 0.1334; Figure 2(b)). In addition, the increased APLN expression exhibited poor RFS in LGG patients (*p* = 5.80*E* − 3; HR: 1.6931; 95% CI: 1.165-2.4605; Figure 2(c)), but not for APLNR gene (*p* = 0.2746; Figure 2(d)). Based on data from GSE107850, the elevated APLN expression had a significant correlation with worse PFS in LGG patients (*p* = 9.50*E* − 3; HR: 1.6897; 95% CI: 1.137-2.511; Figure 2(e)), while high APLNR expression did not show significant correlation with PFS (*p* = 0.5807; Figure 2(f)).

### 3.3. Neither APLN nor APLNR Expression Was Significantly Related to OS, DFI, DSS, or PFI of GBM Patients

To examine whether the high APLN or APLNR expression is correlated with OS, DFI, DSS and PFI in GBM patients, their prognostic values were accessed by LOGpc. The results demonstrated that the high APLN expression had no significant correlation with prognosis of OS, DFI, DSS, or PFI in TCGA dataset for GBM patients. APLN did not display significant correlation with OS from CGGAarray, CGGAseq, GSE42669, or GSE7696 datasets for GBM patients (Table 1). The elevated APLNR expression did not show significant correlation with the prognosis in terms of OS, DFI, DSS, or PFI from TCGA dataset for GBM patients. APLNR did not display significant correlation with OS from CGGAarray, CGGAseq, GSE42669, GSE7696, or GSE30472 datasets for GBM patients (Table 1).

### 3.4. The Influence of Clinical Characteristics on Prognostic Outcome of LGG Patients

In order to determine whether the prognosis correlation of APLN was affected by clinical characteristics of LGG patients, LGG cases in TCGA dataset were further divided into subgroups and measured the prognostic association of APLN according to seizure history (with/without), sample type (primary tumor/recurrent tumor), targeted molecular therapy (yes/not), and histological type (astrocytoma/oligoastrocytoma/oligodendroglioma).

**Figure 3:**
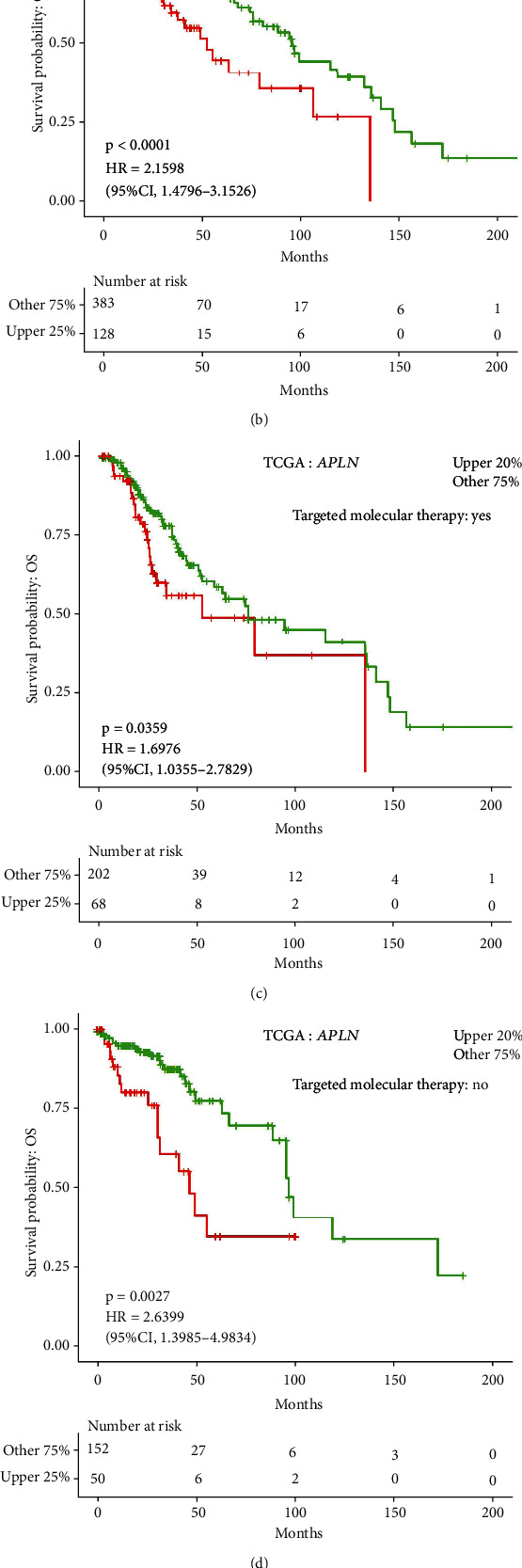
Kaplan-Meier curves of overall survival (OS) in LGG patients with seizure history (a), primary tumor (b), targeted molecular therapy (c), no targeted molecular therapy (d), and oligodendroglioma (e) from TCGA data.

The high APLN expression had poor OS in LGG patients with seizure history (*p* = 4*E* − 04; HR: 2.3864; 95% CI: 1.4689-3.877; Figure 3(a)), with primary tumor (*p* < 0.0001; HR: 2.1598; 95% CI: 1.4796-3.1526; Figure 3(b)), with targeted molecular therapy (*p* = 3.59*E* − 2; HR: 1.6976; 95% CI: 1.0355-2.7829; Figure 3(c)), without targeted molecular therapy (*p* = 2.7*E* − 3; HR: 2.6399; 95% CI: 1.3985-4.9834; Figure 3(d)), and with oligodendroglioma (*p* = 9*E* − 04; HR: 2.9098; 95% CI: 1.5501-5.4622; Figure 3(e)). Prognosis analysis for RFS in LOGpc demonstrated that the elevated APLN expression is associated with poor RFS in LGG patients with seizure history (*p* = 9.4*E* − 3; HR: 1.8683; 95% CI: 1.1654-2.995; Figure S1(a)), with primary tumor (*p* = 0.0237; HR: 1.5633; 95% CI: 1.0613-2.3027; Figure S1(b)), with targeted molecular therapy (*p* = 1.8*E* − 3; HR: 2.0505; 95% CI: 1.3049-3.2221; Figure S1(c)), and with oligodendroglioma (*p* = 0.012; HR: 2.1767; 95% CI: 1.1864-3.9936; Figure S1(d)).

However, the elevated APLN expression did not display inferior OS in LGG patients without seizure history (*p* = 0.1032), with recurrent tumor (*p* = 0.6446), with astrocytoma (*p* = 0.115) or oligoastrocytoma (*p* = 0.108; Figure S2). The high APLN expression was not correlated with RFS in LGG patients without seizure history (*p* = 0.7572), with recurrent tumor (*p* = 0.1468), without targeted molecular therapy (*p* = 0.462), with astrocytoma (*p* = 0.7805), or with oligoastrocytoma (*p* = 0.0809; Figure S3).

The LGG cases from GSE107850 dataset are subdivided according to treatment therapy and type of surgery. Elevated APLN expression displayed worse PFS in LGG patients with biopsy (*p* = 0.0429), but not in patients with partial resection (*p* = 0.4375) or total resection (*p* = 0.2321). The increased APLN expression is associated with inferior PFS in patients with temozolomide (*p* = 0.0396), but not in patients with radiation therapy (*p* = 0.063; Figure S4). For high APLN expression group (upper 50%), LGG patients with mutant IDH status had inferior PFS (*p* = 2.5*E* − 3) in comparison to patients with IDH wildtype status, but not for low APLN expression group (lower 50%, *p* = 0.7659; Figures S5(a, b)), indicating that combination of IDH mutation status and APLN could guide the prognostication more specifically for LGG patients. Selection of the most suitable treatment to each LGG patient is the goal of precision medicine. However, no matter whether APLN is high or low, there is no significant outcome difference between RT and TMZ treatment groups (Figures S5(c, d)).

### 3.5. Protein Expression of APLN in an Independent LGG Cohort

**Figure 4:**
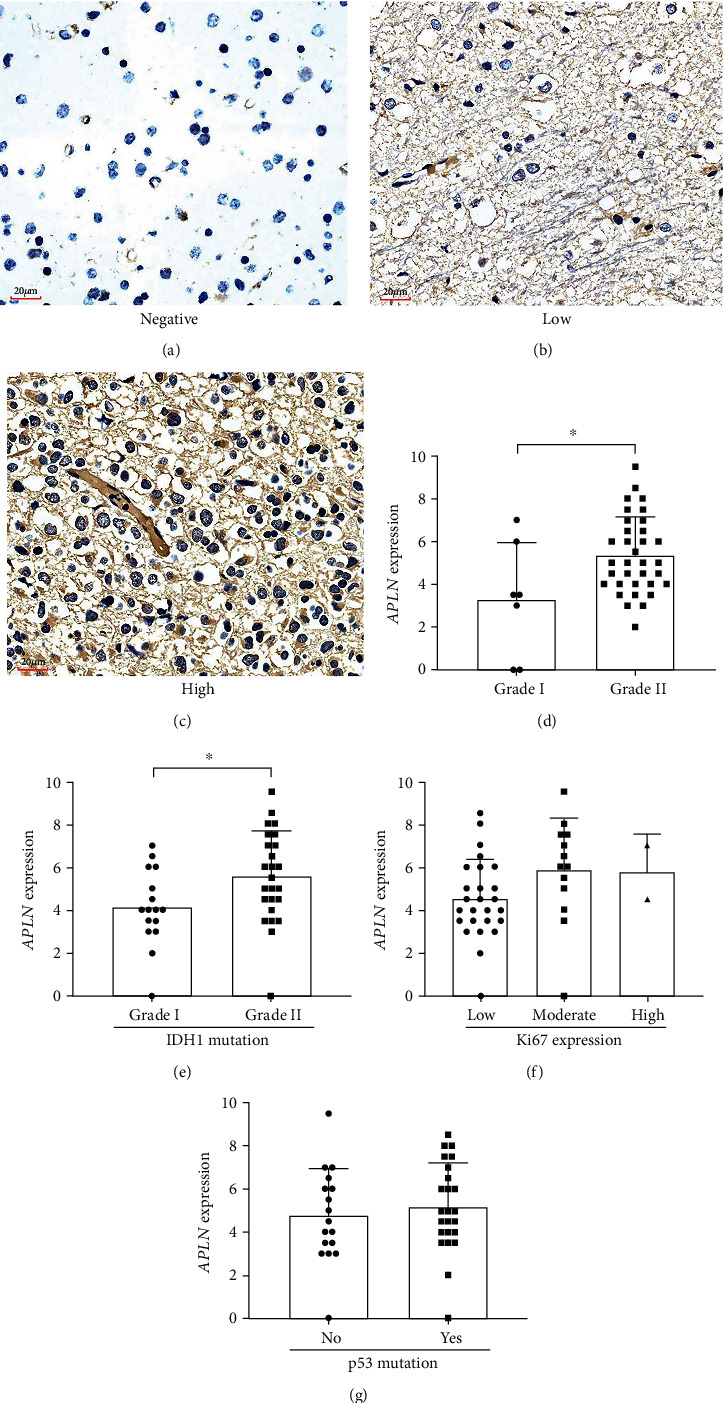
APLN expression in LGG clinical samples. (a–c) Photomicrographs show immunohistochemistry staining of APLN (Negative, a; Low, b; High, c). The staining scores of APLN in grade I/II LGG cases (d), with/without IDH1 mutation (e), with different Ki67 expression (f), and with/without p53 mutation (g).

To further validate the expression of APLN in LGG, APLN protein expression was examined on a tissue chip through an immunohistochemical assay. Overall, the samples from 147 LGG patients were included in the study. APLN positive staining was observed mainly in cytoplasm of cancer cells, and weak staining was showed in the membrane (Figures 4(a)–4(c)). The staining scores of APLN were significantly higher in grade II LGG cancers than grade I (*p* < 0.05), as shown in Figure 4(d). In addition, staining scores of APLN in LGG cases with IDH1 mutation were significantly higher than in patients without IDH1 mutation (*p* < 0.05, Figure 4(e)). However, there were no significant differences of staining scores of APLN in LGG cases with different Ki67 expression (*p* = 0.150, Figure 4(f)), and with/without p53 mutation (*p* = 0.549, Figure 4(g)).

### 3.6. APLN Is an Independent Prognostic Biomarker for Poor OS, RFS, and PFS of LGGs

Univariate analysis showed that the high expression correlated with inferior OS (Table 2), RFS (Table 3), and PFS (Table 4) for LGG. Multivariate analysis indicated that elevated APLN expression is an independent prognostic indicator in terms of OS (*p* = 2*E* − 3; HR: 1.971; 95% CI: 1.294-3.002, Table 2), RFS (*p* = 3*E* − 3; HR: 1.787; 95% CI: 1.222-2.612; Table 3), and PFS (*p* = 0.033; HR: 1.578; 95% CI: 1.037-2.400; Table 4).

### 3.7. Interaction Networks of APLN

**Figure 5:**
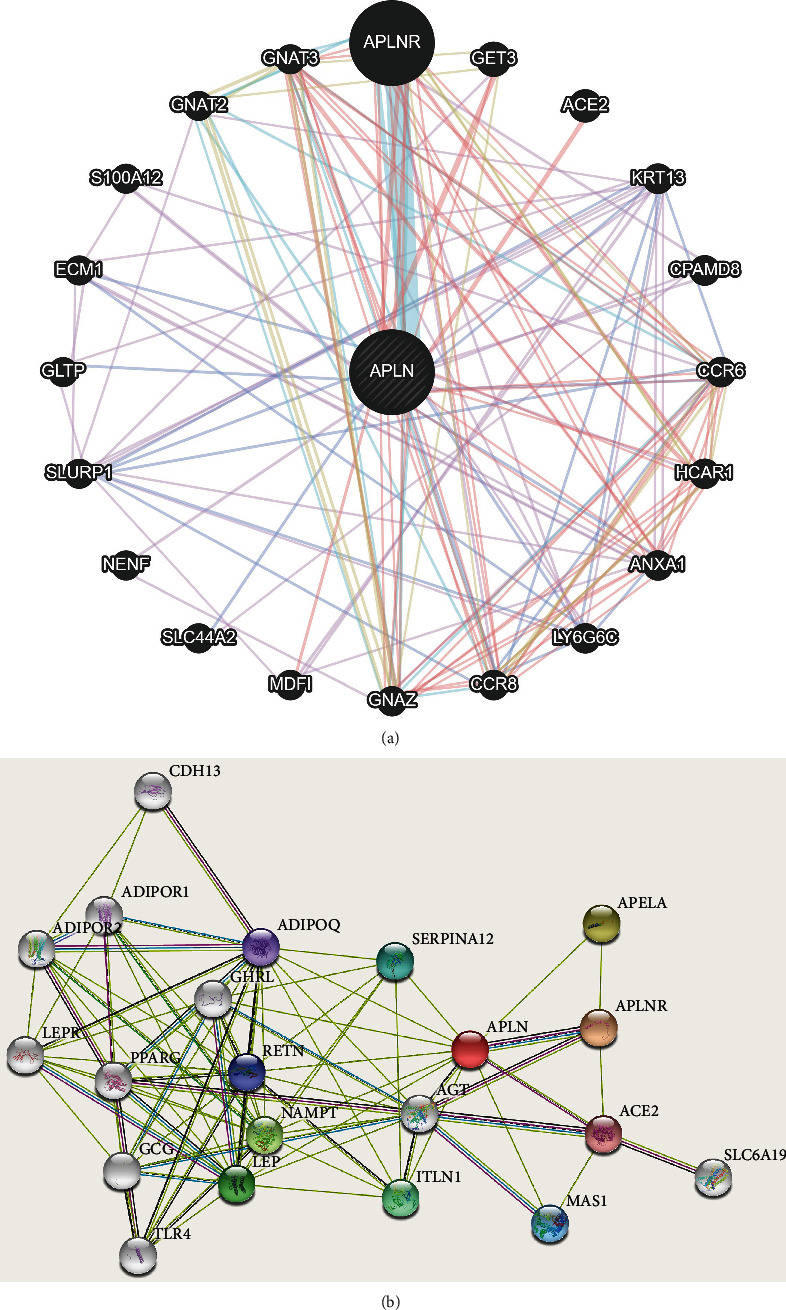
Interaction network of APLN. (a) Gene–gene interaction network for APLN in the GeneMANIA database. (b) Protein–protein interaction (PPI) network for APLN in the STRING database. The PPI network from (b) contains 21 nodes and 87 edges, and the line thickness represents the strength of data support.

The analysis using GeneMANIA demonstrated 20 correlated genes with APLN, including APLNR, GET3, ACE2, KRT13, CPAMD8, CCR6, HCAR1, ANXA1, LY6G6C, CCR8, GNAZ, MDFI, SLC44A2, NENF, SLURP1, GLTP, ECM1, S100A12, GNAT2, and GNAT3 (Figure 5(a)). To further identify the potentially interacting proteins with APLN, we constructed PPI network using STRING. The PPI network with 21 nodes and 87 edges was noticed after omitting unconnected nodes (Figure 5(b)). The top 10 hub proteins were identified as predicted functional partners with APLN, and they were APLNR, APELA, NAMPT, LEP, ITLN1, SERPINA12, MAS1, RETN, ADIPOQ, and ACE2.

## 4. Discussion

LGG is infiltrative neoplasm which generally occurred in the cerebral hemispheres of adults, and encompassed astrocytomas, oligodendrogliomas, and oligoastrocytomas [20]. In the current study, the definition of glioma was according to the WHO clasasification [20]. A part of LGG may be speedily developed to grade IV glioma, becoming GBM [40]. The survival of LGG patients ranged from 1 to 15 years, while the median survival time of GBM patients ranged from 12-15 months [41, 42]. Although different treatment approaches such as surgical resection, chemotherapy, and radiotherapy were applied, the survival rate for glioma patient was still low, and the discovery of novel biomarkers were required.

APLN/APLNR was reported to be involved in the regulation of tumor growth, cancer cell migration, neoangiogenesis, and apoptosis in different types of cancers. In the present study, we demonstrated that APLN and APLNR expression were upregulated in both LGG and GBM clinical cases, compared with corresponding normal tissues. The result was in line with the previous reports that APLN/APLNR gene expression were increased in gastric cancer, liver cancer, cholangiocarcinoma, lung cancer, ovarian cancer, prostate cancer, etc., compared with the equivalent normal tissues or cells [6]. Harford-Wright et al. reported that APLN/APLNR may act as a paracrine signal that sustains tumor cell expansion and progression in glioblastoma [43] indicating a crucial relation between APLN/APLNR and glioblastoma.

APLN/APLNR had been showed as prognostic markers in several types of cancers. High APLN level in tumor tissue predicted worse outcome for patients with gastric cancer and muscle-invasive bladder cancer [44, 45]. The elevated serum APLNR was correlated with inferior OS in patients with clear-cell renal cell carcinoma [46]. However, the relationship between APLN/APLNR and prognosis of brain tumor is still unknown. In current study, the correlation of APLN expression and prognostic value was evaluated and the results indicated that high APLN, but not APLNR, was significantly correlated with poor OS, RFS, and PFS in LGG patients. However, neither high APLN nor high APLNR was significantly correlated to prognostic values of OS, DFI, DSS, or PFI in GBM patients. Additionally, immunohistochemical staining confirmed APLN expression in cytoplasm of glioma cells, which was consistent with the distribution of APLN in colon adenocarcinomas [47] and breast carcinoma [48]. The APLN expression was significantly higher in grade II LGG cases than grade I. These results suggested that high APLN expression could predict poor prognosis for LGG patients.

Further analysis was performed to explore whether the correlation between high APLN expression and poor OS/RFS/PFS of LGG was influenced by clinical characteristics, including seizure history, sample type, targeted molecular therapy, histological type, chemo/radio-therapy, and type of surgery. Our results showed that the significant correlation between elevated APLN expression and inferior OS or RFS in LGG patients was restricted to patients with seizure history, primary tumor, targeted molecular therapy, oligodendroglioma, biopsy, and TMZ therapy. This result will facilitate clinicians to manage the personalized treatment for LGG.

In this study, GeneMANIA analysis showed that the top 20 correlated genes were screened. These genes were enriched in G protein subunits (including GNAZ, GNAT2, GNAT3) and chemokine receptors (CCR6, CCR8). The results indicated a correlationship between APLN and G protein, which was consistent with the previous report that APLN-activated APLNR couples to G protein, such as G*α*i and G*α*q [49, 50]. Based on the PPI network, we obtained top 10 hub proteins, and they were mainly classified as pathways of angiotensin (ACE2, MAS1) and adipokines (LEP, SERPINA12, ADIPOQ). The angiotensin and LEP played a regulatory role in glioma cells [51, 52].

## 5. Conclusions

In conclusion, both APLN and APLNR expression was significantly elevated in LGGs and GBMs compared with normal tissues, but only the high APLN expression was correlated with poor OS, RFS, and PFS in LGG patients, providing a clue that APLN might serve as a prognostic biomarker for LGG. Further studies are required to investigate the molecular mechanism of APLN in tumorigenesis and progression of LGG.

## Figures and Tables

**Table 1:** Kaplan-Meier plot curve showed no significant correlation between APLN/APLNR and GBM survival outcomes in TCGA, CGGAarray, CGGAseq, GSE42669, GSE7696, and GSE30472 datasets.

Dataset	Survival outcome	HR	95% CI	*p*
*APLN*				
TCGA	OS	1.1098	0.7248-1.6993	0.6371
TCGA	DFI	0.9897	0.5925-1.6531	0.9684
TCGA	DSS	1.0163	0.6413-1.6106	0.9451
TCGA	PFI	1.0351	0.6759-1.5849	0.8741
CGGAarray	OS	1.1471	0.7361-1.7876	0.5443
CGGAseq	OS	0.9578	0.5735-1.5998	0.8692
GSE42669	OS	0.7590	0.3919-1.4702	0.4137
GSE7696	OS	1.4013	0.7977-2.4615	0.2405
Combined	OS	1.0987	0.8793-1.3727	0.4076
*APLNR*				
TCGA	OS	0.9279	0.6102-1.4110	0.7265
TCGA	DFI	0.8711	0.5306-1.4301	0.5854
TCGA	DSS	1.2166	0.7852-1.8850	0.3801
TCGA	PFI	0.9518	0.6268-1.4452	0.8166
CGGAarray	OS	0.9294	0.5931-1.4565	0.7495
CGGAseq	OS	0.6724	0.4014-1.1264	0.1316
GSE42669	OS	0.6807	0.3511-1.3197	0.2548
GSE7696	OS	1.0759	0.6092-1.9000	0.8011
GSE30472	OS	0.5997	0.3026-1.1886	0.1429
Combined	OS	0.8135	0.6576-1.0065	0.0574

**Table 2:** Univariate and multivariate analysis of APLN and other clinicopathological factors of LGG for OS in TCGA dataset.

Parameters	Univariate analysis				Multivariate analysis			
	HR	95% CI		*p*	HR	95% CI		*p*
Age >50 vs. ≤50	3.523	2.459	5.049	≤0.001	3.775	2.472	5.766	≤0.001
Gender Female vs. male	1.143	0.810	1.614	0.446				
Headache history Yes vs. no	0.849	0.582	1.240	0.397				
Motor movement Yes vs. no	1.368	0.888	2.106	0.155	1.347	0.865	2.100	0.188
Radiation therapy Yes vs. no	1.840	1.205	2.810	0.005	0.976	0.598	1.594	0.923
Neoplasm histologic grade, G3 vs. G1/G2	3.354	2.298	4.895	≤0.001	3.175	1.966	5.128	≤0.001
Seizure history Yes vs. no	0.868	0.608	1.239	0.435				
Histology type OD vs. AC/OA	0.680	0.474	0.975	0.036	0.559	0.366	0.854	0.007
Sample type PT vs. RT	1.118	0.544	2.297	0.762				
Targeted molecular therapy, yes vs. no	1.383	0.951	2.010	0.090	0.812	0.515	1.280	0.370
APLN expression High vs. low	2.064	1.425	2.990	≤0.001	1.971	1.294	3.002	0.002

Notes: AC: astrocytoma; OA: oligoastrocytoma; OD: oligodendroglioma; PT: primary tumor; and RT: recurrent tumor.

**Table 3:** Univariate and multivariate analysis of APLN and other clinicopathological factors of LGG for RFS in TCGA dataset.

Parameters	Univariate analysis				Multivariate analysis			
	HR	95% CI		*p*	HR	95% CI		*p*
Age >50 vs. ≤50	1.735	1.201	2.504	0.003	1.789	1.224	2.616	0.003
Gender Female vs. male	0.838	0.603	1.164	0.292				
Headache history Yes vs. no	1.049	0.737	1.491	0.791				
Motor movement Yes vs. no	0.863	0.545	1.367	0.530				
Radiation therapy Yes vs. no	1.315	0.912	1.895	0.143	0.967	0.647	1.444	0.870
Neoplasm histologic grade, G3 vs. G1/G2	1.631	1.161	2.292	0.005	1.411	0.948	2.101	0.090
Seizure history Yes vs. no	0.981	0.697	1.382	0.914				
Histology type OD vs. AC/OA	0.821	0.581	1.160	0.263				
Sample type PT vs. RT	2.062	1.129	3.764	0.018	2.858	1.521	5.370	0.001
Targeted molecular therapy, yes vs. no	1.488	1.048	2.111	0.026	1.366	0.913	2.043	0.129
APLN expression High vs. low	1.693	1.165	2.460	0.006	1.787	1.222	2.612	0.003

Notes: AC: astrocytoma; OA: oligoastrocytoma; OD: oligodendroglioma; PT: primary tumor; and RT: recurrent tumor.

**Table 4:** Univariate and multivariate analysis of APLN and other clinicopathological factors of LGG for PFS in GSE107850 dataset.

Parameters	Univariate analysis				Multivariate analysis			
	HR	95% CI		*p*	HR	95% CI		*p*
Age >50 vs. ≤50	0.763	0.490	1.187	0.230				
Gender Female vs. male	1.360	0.911	2.031	0.133	1.175	0.763	1.808	0.464
Treatment therapy RT vs. TMZ	1.205	0.815	1.781	0.351				
Type of surgery Biopsy vs. PR/TR	0.918	0.567	1.487	0.729				
Histology AA vs. AOA/AOD	0.767	0.504	1.167	0.215				
IDH status Normal vs. mutated	0.454	0.235	0.880	0.019	0.473	0.242	0.926	0.029
APLN expression High vs. low	1.689	1.137	2.511	0.009	1.578	1.037	2.400	0.033

Notes: AA: anaplastic astrocytoma grade II; AOA: anaplastic oligoastrocytoma grade II; AOD: anaplastic oligodendroglioma grade II; PR: partial removal; RT: radiation therapy; TMZ; temozolomide; and TR: total removal.

## Data Availability

The data used to support the findings of this study are available from the corresponding author upon request.

## Supplementary Materials

Supplementary Materials Figure S1. Kaplan-Meier curves of recurrence free survival (RFS) in LGG patients with seizure history (A), primary tumor (B), targeted molecular therapy (C), and oligodendroglioma (D) from TCGA data. Figure S2. Kaplan-Meier curves of overall survival (OS) in LGG patients without seizure history (A), with recurrent tumor (B), with astrocytoma (C), and with oligoastrocytoma (D) from TCGA data. Figure S3. Kaplan-Meier curves of recurrence free survival (RFS) in LGG patients without seizure history (A), with recurrent tumor (B), without targeted molecular therapy (C), with astrocytoma (D), and with oligoastrocytoma (E) from TCGA data. Figure S4. Kaplan-Meier curves of progression free survival (PFS) in LGG patients with biopsy (A), partial resection (B), total resection (C), temozolomide (TMZ, D), and radiation therapy (RT, E) from GSE107850 dataset. Figure S5. Kaplan-Meier curves of PFS in LGG patients with different IDH status (mutated vs. normal) in high APLN expression group (A) and low APLN expression group (B). Kaplan-Meier curves of PFS in LGG patients with different treatment therapies (RT vs. TMZ, B) in high APLN expression group (C) and low APLN expression group (D). The high APLN expression was defined by upper 50% expression, and low APLN expression group was defined by lower 50% expression in GSE107850 dataset. RT, radiation therapy; TMZ, temozolomide. Table S1. Information of LGG clinical samples from TCGA. Table S2. Information of LGG clinical samples from GSE107850.
