# Supplementary material for: High APLN Expression Predicts Poor Prognosis for Glioma Patients
Source: Oxid Med Cell Longev. 2022 Sep 22;2022:8393336. doi: 10.1155/2022/8393336 (PMC9526648; doi:10.1155/2022/8393336)
Supplement: Supplementary Materials — Figure S1. Kaplan-Meier curves of recurrence free survival (RFS) in LGG patients with seizure history (A), primary tumor (B), targeted molecular therapy (C), and oligodendroglioma (D) from TCGA data. Figure S2. Kaplan-Meier curves of overall survival (OS) in LGG patients without seizure history (A), with recurrent tumor (B), with astrocytoma (C), and with oligoastrocytoma (D) from TCGA data. Figure S3. Kaplan-Meier curves of recurrence free survival (RFS) in LGG patients without seizure history (A), with recurrent tumor (B), without targeted molecular therapy (C), with astrocytoma (D), and with oligoastrocytoma (E) from TCGA data. Figure S4. Kaplan-Meier curves of progression free survival (PFS) in LGG patients with biopsy (A), partial resection (B), total resection (C), temozolomide (TMZ, D), and radiation therapy (RT, E) from GSE107850 dataset. Figure S5. Kaplan-Meier curves of PFS in LGG patients with different IDH status (mutated vs. normal) in high APLN expression group (A) and low APLN expression group (B). Kaplan-Meier curves of PFS in LGG patients with different treatment therapies (RT vs. TMZ, B) in high APLN expression group (C) and low APLN expression group (D). The high APLN expression was defined by upper 50% expression, and low APLN expression group was defined by lower 50% expression in GSE107850 dataset. RT, radiation therapy; TMZ, temozolomide. Table S1. Information of LGG clinical samples from TCGA. Table S2. Information of LGG clinical samples from GSE107850. [file 8393336.f1.zip › Figure S1 (1).docx]

**Supplementary Materials**


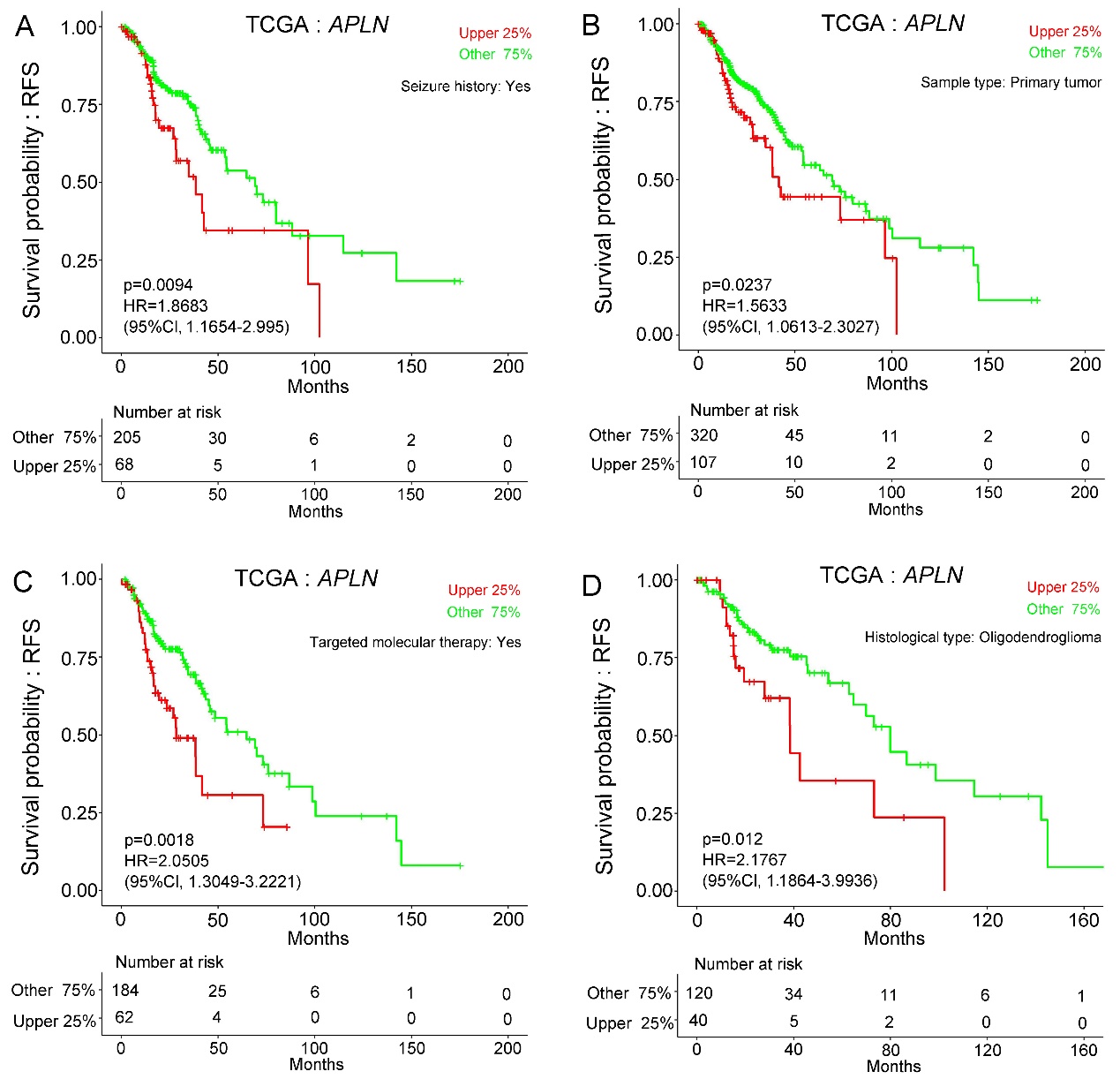


**Figure S1**. Kaplan-Meier curves of recurrence free survival (RFS) in LGG patients with seizure history (A), primary tumor (B), targeted molecular therapy (C), and oligodendroglioma (D) from TCGA data.
