# Supplementary material for: High APLN Expression Predicts Poor Prognosis for Glioma Patients
Source: Oxid Med Cell Longev. 2022 Sep 22;2022:8393336. doi: 10.1155/2022/8393336 (PMC9526648; doi:10.1155/2022/8393336)
Supplement: Supplementary Materials — Figure S1. Kaplan-Meier curves of recurrence free survival (RFS) in LGG patients with seizure history (A), primary tumor (B), targeted molecular therapy (C), and oligodendroglioma (D) from TCGA data. Figure S2. Kaplan-Meier curves of overall survival (OS) in LGG patients without seizure history (A), with recurrent tumor (B), with astrocytoma (C), and with oligoastrocytoma (D) from TCGA data. Figure S3. Kaplan-Meier curves of recurrence free survival (RFS) in LGG patients without seizure history (A), with recurrent tumor (B), without targeted molecular therapy (C), with astrocytoma (D), and with oligoastrocytoma (E) from TCGA data. Figure S4. Kaplan-Meier curves of progression free survival (PFS) in LGG patients with biopsy (A), partial resection (B), total resection (C), temozolomide (TMZ, D), and radiation therapy (RT, E) from GSE107850 dataset. Figure S5. Kaplan-Meier curves of PFS in LGG patients with different IDH status (mutated vs. normal) in high APLN expression group (A) and low APLN expression group (B). Kaplan-Meier curves of PFS in LGG patients with different treatment therapies (RT vs. TMZ, B) in high APLN expression group (C) and low APLN expression group (D). The high APLN expression was defined by upper 50% expression, and low APLN expression group was defined by lower 50% expression in GSE107850 dataset. RT, radiation therapy; TMZ, temozolomide. Table S1. Information of LGG clinical samples from TCGA. Table S2. Information of LGG clinical samples from GSE107850. [file 8393336.f1.zip › Table S1.pdf]

Table S1. Information of LGG clinical samples from TCGA.

No.	sampleID	_EVENT	_INTEGRATION	APLN Expression	OS.time	OS	OS.unit	_PATIENT	RFS.time	RFS	RFS.unit	_TIME_TO_EVENT	_TIME_TO_EVENT_UNIT	_cohort	_primary_disease	_primary_site
1	TCGA-CS-4938-01	0.00	TCGA-CS-4938-01	11.86	3574.00	0.00	days	TCGA-CS-4938			days	3574.00	days	TCGA Lower Grade Glioma (LGG)	brain lower grade glioma	Brain
2	TCGA-CS-4941-01	1.00	TCGA-CS-4941-01	11.09	234.00	1.00	days	TCGA-CS-4941	9.00	1.00	days	234.00	days	TCGA Lower Grade Glioma (LGG)	brain lower grade glioma	Brain
3	TCGA-CS-4942-01	1.00	TCGA-CS-4942-01	9.01	1335.00	1.00	days	TCGA-CS-4942	1184.00	1.00	days	1335.00	days	TCGA Lower Grade Glioma (LGG)	brain lower grade glioma	Brain
4	TCGA-CS-4943-01	1.00	TCGA-CS-4943-01	8.83	1106.00	1.00	days	TCGA-CS-4943			days	1106.00	days	TCGA Lower Grade Glioma (LGG)	brain lower grade glioma	Brain
5	TCGA-CS-4944-01	0.00	TCGA-CS-4944-01	10.52	1828.00	0.00	days	TCGA-CS-4944	1828.00	0.00	days	1828.00	days	TCGA Lower Grade Glioma (LGG)	brain lower grade glioma	Brain
6	TCGA-CS-5393-01	0.00	TCGA-CS-5393-01	8.88	1222.00	0.00	days	TCGA-CS-5393	1222.00	0.00	days	1222.00	days	TCGA Lower Grade Glioma (LGG)	brain lower grade glioma	Brain
7	TCGA-CS-5394-01	0.00	TCGA-CS-5394-01	9.31	8.00	0.00	days	TCGA-CS-5394			days	8.00	days	TCGA Lower Grade Glioma (LGG)	brain lower grade glioma	Brain
8	TCGA-CS-5395-01	1.00	TCGA-CS-5395-01	11.76	639.00	1.00	days	TCGA-CS-5395	287.00	1.00	days	639.00	days	TCGA Lower Grade Glioma (LGG)	brain lower grade glioma	Brain
9	TCGA-CS-5396-01	0.00	TCGA-CS-5396-01	8.94	1631.00	0.00	days	TCGA-CS-5396			days	1631.00	days	TCGA Lower Grade Glioma (LGG)	brain lower grade glioma	Brain
10	TCGA-CS-5397-01	1.00	TCGA-CS-5397-01	8.31	194.00	1.00	days	TCGA-CS-5397	194.00	0.00	days	194.00	days	TCGA Lower Grade Glioma (LGG)	brain lower grade glioma	Brain
11	TCGA-CS-6186-01	1.00	TCGA-CS-6186-01	10.15	538.00	1.00	days	TCGA-CS-6186	188.00	1.00	days	538.00	days	TCGA Lower Grade Glioma (LGG)	brain lower grade glioma	Brain
12	TCGA-CS-6188-01	1.00	TCGA-CS-6188-01	11.25	814.00	1.00	days	TCGA-CS-6188			days	814.00	days	TCGA Lower Grade Glioma (LGG)	brain lower grade glioma	Brain
13	TCGA-CS-6290-01	1.00	TCGA-CS-6290-01	10.80	1137.00	1.00	days	TCGA-CS-6290			days	1137.00	days	TCGA Lower Grade Glioma (LGG)	brain lower grade glioma	Brain
14	TCGA-CS-6665-01	0.00	TCGA-CS-6665-01	10.20	1568.00	0.00	days	TCGA-CS-6665			days	1568.00	days	TCGA Lower Grade Glioma (LGG)	brain lower grade glioma	Brain
15	TCGA-CS-6666-01	0.00	TCGA-CS-6666-01	11.12	1428.00	0.00	days	TCGA-CS-6666	1428.00	0.00	days	1428.00	days	TCGA Lower Grade Glioma (LGG)	brain lower grade glioma	Brain
16	TCGA-CS-6667-01	0.00	TCGA-CS-6667-01	8.73	1469.00	0.00	days	TCGA-CS-6667			days	1469.00	days	TCGA Lower Grade Glioma (LGG)	brain lower grade glioma	Brain
17	TCGA-CS-6668-01	0.00	TCGA-CS-6668-01	8.54	1519.00	0.00	days	TCGA-CS-6668			days	1519.00	days	TCGA Lower Grade Glioma (LGG)	brain lower grade glioma	Brain
18	TCGA-CS-6669-01	0.00	TCGA-CS-6669-01	8.24	1494.00	0.00	days	TCGA-CS-6669			days	1494.00	days	TCGA Lower Grade Glioma (LGG)	brain lower grade glioma	Brain
19	TCGA-CS-6670-01	0.00	TCGA-CS-6670-01	9.11	1426.00	0.00	days	TCGA-CS-6670			days	1426.00	days	TCGA Lower Grade Glioma (LGG)	brain lower grade glioma	Brain
20	TCGA-DB-5270-01	0.00	TCGA-DB-5270-01	10.14	3733.00	0.00	days	TCGA-DB-5270	3733.00	0.00	days	3733.00	days	TCGA Lower Grade Glioma (LGG)	brain lower grade glioma	Brain
21	TCGA-DB-5273-01	0.00	TCGA-DB-5273-01	10.53	2493.00	0.00	days	TCGA-DB-5273	2493.00	0.00	days	2493.00	days	TCGA Lower Grade Glioma (LGG)	brain lower grade glioma	Brain
22	TCGA-DB-5274-01	0.00	TCGA-DB-5274-01	9.23	2289.00	0.00	days	TCGA-DB-5274	2289.00	0.00	days	2289.00	days	TCGA Lower Grade Glioma (LGG)	brain lower grade glioma	Brain
23	TCGA-DB-5275-01	0.00	TCGA-DB-5275-01	10.69	1458.00	0.00	days	TCGA-DB-5275			days	1458.00	days	TCGA Lower Grade Glioma (LGG)	brain lower grade glioma	Brain
24	TCGA-DB-5276-01	0.00	TCGA-DB-5276-01	11.22	2218.00	0.00	days	TCGA-DB-5276	2218.00	0.00	days	2218.00	days	TCGA Lower Grade Glioma (LGG)	brain lower grade glioma	Brain
25	TCGA-DB-5277-01	1.00	TCGA-DB-5277-01	8.92	1547.00	1.00	days	TCGA-DB-5277	1156.00	1.00	days	1547.00	days	TCGA Lower Grade Glioma (LGG)	brain lower grade glioma	Brain
26	TCGA-DB-5278-01	0.00	TCGA-DB-5278-01	9.70	2772.00	0.00	days	TCGA-DB-5278	2772.00	0.00	days	2772.00	days	TCGA Lower Grade Glioma (LGG)	brain lower grade glioma	Brain
27	TCGA-DB-5279-01	0.00	TCGA-DB-5279-01	9.14	1354.00	0.00	days	TCGA-DB-5279			days	1354.00	days	TCGA Lower Grade Glioma (LGG)	brain lower grade glioma	Brain
28	TCGA-DB-5280-01	0.00	TCGA-DB-5280-01	10.26	1112.00	0.00	days	TCGA-DB-5280	1112.00	0.00	days	1112.00	days	TCGA Lower Grade Glioma (LGG)	brain lower grade glioma	Brain
29	TCGA-DB-5281-01	0.00	TCGA-DB-5281-01	9.87	2381.00	0.00	days	TCGA-DB-5281	2381.00	0.00	days	2381.00	days	TCGA Lower Grade Glioma (LGG)	brain lower grade glioma	Brain
30	TCGA-DB-A4X9-01	0.00	TCGA-DB-A4X9-01	10.36	1412.00	0.00	days	TCGA-DB-A4X9	1412.00	0.00	days	1412.00	days	TCGA Lower Grade Glioma (LGG)	brain lower grade glioma	Brain
31	TCGA-DB-A4XA-01	0.00	TCGA-DB-A4XA-01	10.84	573.00	0.00	days	TCGA-DB-A4XA	573.00	0.00	days	573.00	days	TCGA Lower Grade Glioma (LGG)	brain lower grade glioma	Brain
32	TCGA-DB-A4XB-01	0.00	TCGA-DB-A4XB-01	7.61	919.00	0.00	days	TCGA-DB-A4XB	919.00	0.00	days	919.00	days	TCGA Lower Grade Glioma (LGG)	brain lower grade glioma	Brain
33	TCGA-DB-A4XC-01	0.00	TCGA-DB-A4XC-01	9.62	1421.00	0.00	days	TCGA-DB-A4XC	1421.00	0.00	days	1421.00	days	TCGA Lower Grade Glioma (LGG)	brain lower grade glioma	Brain
34	TCGA-DB-A4XD-01	0.00	TCGA-DB-A4XD-01	9.27	1210.00	0.00	days	TCGA-DB-A4XD	1210.00	0.00	days	1210.00	days	TCGA Lower Grade Glioma (LGG)	brain lower grade glioma	Brain

No.	additional_pharmaceutical_therapy	additional_radiation_therapy	additional_surgery_locoregional_procedure	additional_surgery_metastatic_procedure	age_at_initial_pathologic_diagnosis	animal_insect_allergy_history	animal_insect_allergy_types	asthma_history	bcr_followup_barcode	bcr_patient_barcode	bcr_sample_barcode	days_to_additional_surgery_locoregional_procedure	days_to_additional_surgery_metastatic_procedure	days_to_birth	days_to_collection	days_to_death	days_to_initial_pathologic_diagnosis	days_to_last_followup	days_to_new_tumor_event_after_initial_treatment	days_to_performance_status_assessment	eastern_cancer_oncology_group
1					31.00	YES	Dog, Cat	NO	TCGA-CS-4938-F71559	TCGA-CS-4938	TCGA-CS-4938-01B			(11509.00)			0.00	3574.00		(1.00)	1.00
2	NO	YES	YES	NO	67.00	NO		NO	TCGA-CS-4941-F10024	TCGA-CS-4941		77.00	77.00	(24578.00)		234.00	0.00		9.00	82.00	1.00
3	YES	YES	YES	NO	44.00	NO		NO	TCGA-CS-4942-F10210	TCGA-CS-4942	TCGA-CS-4942-01A	1189.00	1189.00	(16297.00)		1335.00	0.00		1184.00	1226.00	2.00
4					37.00				TCGA-CS-4943-F71549	TCGA-CS-4943	TCGA-CS-4943-01A			(13565.00)		1106.00	0.00	481.00		9.00	4.00
5					50.00				TCGA-CS-4944-F71553	TCGA-CS-4944	TCGA-CS-4944-01A			(18494.00)			0.00	1828.00		(2.00)	1.00
6					39.00			NO	TCGA-CS-5393-F10073	TCGA-CS-5393	TCGA-CS-5393-01A			(14418.00)			0.00	1222.00		857.00	0.00
7					40.00	NO		NO	TCGA-CS-5394-F71692	TCGA-CS-5394	TCGA-CS-5394-01A			(14920.00)			0.00	8.00		(1.00)	
8	NO	NO	YES	YES	43.00				TCGA-CS-5395-F10075	TCGA-CS-5395	TCGA-CS-5395-01A	287.00	287.00	(15848.00)		639.00	0.00		287.00	379.00	2.00
9					53.00			NO	TCGA-CS-5396-F71560	TCGA-CS-5396	TCGA-CS-5396-01A			(19399.00)			0.00	1631.00		20.00	1.00
10					54.00	NO		NO	TCGA-CS-5397-F17567	TCGA-CS-5397	TCGA-CS-5397-01A			(19866.00)		194.00	0.00			(1.00)	1.00
11	YES	YES	YES	NO	58.00	NO		NO	TCGA-CS-6186-F18628	TCGA-CS-6186	TCGA-CS-6186-01A	213.00		(21321.00)		538.00	0.00		188.00	212.00	1.00
12					48.00	NO		NO	TCGA-CS-6188-F71551	TCGA-CS-6188	TCGA-CS-6188-01A			(17729.00)		814.00	0.00	725.00		(1.00)	1.00
13					31.00	NO		NO	TCGA-CS-6290-F71693	TCGA-CS-6290	TCGA-CS-6290-01A			(11666.00)		1137.00	0.00	546.00		(1.00)	1.00
14					51.00	NO		NO	TCGA-CS-6665-F71561	TCGA-CS-6665	TCGA-CS-6665-01A			(18977.00)			0.00	1568.00		(1.00)	1.00
15					22.00	NO		NO	TCGA-CS-6666-F71562	TCGA-CS-6666	TCGA-CS-6666-01A			(8340.00)			0.00	1428.00		(1.00)	1.00
16					39.00	NO		NO	TCGA-CS-6667-F71563	TCGA-CS-6667	TCGA-CS-6667-01A			(14375.00)			0.00	1469.00		(1.00)	1.00
17					57.00	NO		NO	TCGA-CS-6668-F71564	TCGA-CS-6668	TCGA-CS-6668-01A			(20836.00)			0.00	1519.00		(1.00)	1.00
18					26.00	NO		NO	TCGA-CS-6669-F71567	TCGA-CS-6669	TCGA-CS-6669-01A			(9809.00)			0.00	1494.00		0.00	1.00
19					43.00	NO		NO	TCGA-CS-6670-F71568	TCGA-CS-6670	TCGA-CS-6670-01A			(16066.00)			0.00	1426.00			
20					38.00	YES	Bee/Wasp	NO	TCGA-DB-5270-F42829	TCGA-DB-5270	TCGA-DB-5270-01A			(13904.00)			0.00	3733.00			
21					33.00	NO		NO	TCGA-DB-5273-F42830	TCGA-DB-5273	TCGA-DB-5273-01A			(12152.00)			0.00	2493.00			
22					37.00			NO	TCGA-DB-5274-F42834	TCGA-DB-5274	TCGA-DB-5274-01A			(13680.00)			0.00	2289.00		12.00	1.00
23					36.00	NO		NO	TCGA-DB-5275-F67391	TCGA-DB-5275	TCGA-DB-5275-01A			(13371.00)			0.00	1458.00			
24					32.00			NO	TCGA-DB-5276-F42832	TCGA-DB-5276	TCGA-DB-5276-01A			(11738.00)			0.00	2218.00			
25	YES	YES	NO		34.00			NO	TCGA-DB-5277-F42826	TCGA-DB-5277	TCGA-DB-5277-01A			(12725.00)		1547.00	0.00	1419.00	1156.00		
26					17.00	NO		NO	TCGA-DB-5278-F67393	TCGA-DB-5278	TCGA-DB-5278-01A			(6480.00)			0.00	2772.00			
27					59.00	NO		NO	TCGA-DB-5279-F67396	TCGA-DB-5279	TCGA-DB-5279-01A			(21908.00)			0.00	1354.00			
28					43.00	NO		NO	TCGA-DB-5280-F42831	TCGA-DB-5280	TCGA-DB-5280-01A			(15836.00)			0.00	1112.00			
29					61.00	NO		NO	TCGA-DB-5281-F42828	TCGA-DB-5281	TCGA-DB-5281-01A			(22342.00)			0.00	2381.00			
30					33.00			NO	TCGA-DB-A4X9-F60941	TCGA-DB-A4X9	TCGA-DB-A4X9-01A			(12131.00)	832.00		0.00	1412.00			
31					30.00	NO		NO	TCGA-DB-A4XA-F60947	TCGA-DB-A4XA	TCGA-DB-A4XA-01A			(11195.00)	224.00		0.00	573.00			
32					38.00			NO	TCGA-DB-A4XB-F60944	TCGA-DB-A4XB	TCGA-DB-A4XB-01A			(13990.00)	372.00		0.00	919.00			
33					26.00			NO	TCGA-DB-A4XC-F60948	TCGA-DB-A4XC	TCGA-DB-A4XC-01A			(9705.00)	849.00		0.00	1421.00			
34					32.00			NO	TCGA-DB-A4XD-F60950	TCGA-DB-A4XD	TCGA-DB-A4XD-01A			(11741.00)	719.00		0.00	1210.00			

No.	eczema_history	**family_history_of_cancer**	**family_history_of_primary_brain_tumor**	first_diagnosis_age_asth_ecz_hay_fev_mold_dust	first_diagnosis_age_of_animal_insect_allergy	first_diagnosis_age_of_food_allergy	first_presenting_symptom	first_presenting_symptom_longest_duration	followup_case_report_form_submission_reason	followup_treatment_success	food_allergy_history	food_allergy_types	form_completion_date	gender	hay_fever_history	headache_history	histological_type
1	NO	YES	YES		12 - 20 Years		Seizures	> 181 Days		Stable Disease	NO		42093.00	FEMALE	NO	NO	Astrocytoma
2	NO						Headaches	0 - 30 Days		Progressive Disease	NO		40618.00	MALE	NO	YES	Astrocytoma
3	NO	NO	NO				Mental Status Changes	0 - 30 Days		Progressive Disease	NO		40616.00	FEMALE	NO	NO	Astrocytoma
4							Seizures	> 181 Days					42089.00	MALE		YES	Astrocytoma
5							Mental Status Changes			Progressive Disease			42093.00	MALE		NO	Astrocytoma
6	NO		NO				Headaches	91 - 180 Days		Complete Remission/Response			40615.00	MALE		YES	Astrocytoma
7	NO						Seizures	0 - 30 Days			NO		42094.00	MALE	NO	NO	Astrocytoma
8										Progressive Disease			40615.00	MALE			Oligodendroglioma
9	NO	YES	YES				Headaches			Stable Disease			42093.00	FEMALE		YES	Oligodendroglioma
10	NO	YES	NO				Sensory Changes				NO		40809.00	FEMALE	NO	NO	Astrocytoma
11	NO	NO	NO				Seizures	0 - 30 Days	Scheduled Follow-up Submission	Progressive Disease	NO		40865.00	MALE	NO	NO	Oligoastrocytoma
12	NO	YES	YES				Seizures	0 - 30 Days			NO		42089.00	MALE	NO	NO	Astrocytoma
13	NO	YES	NO				Seizures	0 - 30 Days			NO		42093.00	MALE	NO	NO	Astrocytoma
14	NO	NO	NO				Seizures	0 - 30 Days		Stable Disease	YES	Hazelnuts	42093.00	FEMALE	NO	NO	Astrocytoma
15	NO	YES	NO				Headaches			Complete Remission/Response	NO		42093.00	MALE	NO	YES	Astrocytoma
16	NO	YES	NO				Motor/Movement Changes	91 - 180 Days		Stable Disease	NO		42093.00	FEMALE	YES	NO	Astrocytoma
17	NO	YES	NO				Seizures	> 181 Days		Stable Disease	NO		42093.00	FEMALE	NO	NO	Oligodendroglioma
18	NO	YES	NO				Headaches	31 - 90 Days		Stable Disease	NO		42093.00	FEMALE	NO	YES	Oligodendroglioma
19	NO	NO	NO				Headaches	31 - 90 Days		Progressive Disease	NO		42093.00	MALE	NO	YES	Oligodendroglioma
20	NO		NO				Seizures	0 - 30 Days	Scheduled Follow-up Submission	Complete Remission/Response	NO		41395.00	FEMALE	NO	NO	Oligoastrocytoma
21	NO	NO	NO				Seizures	0 - 30 Days	Scheduled Follow-up Submission	Complete Remission/Response	NO		41395.00	MALE	NO	NO	Astrocytoma
22	NO	NO	NO				Headaches	31 - 90 Days	Scheduled Follow-up Submission	Complete Remission/Response	NO		41395.00	FEMALE	NO	YES	Oligoastrocytoma
23	NO	NO	NO				Headaches		Scheduled Follow-up Submission		YES	lactose containing products	41947.00	MALE	NO	YES	Oligoastrocytoma
24	NO	NO	NO				Seizures	0 - 30 Days	Scheduled Follow-up Submission	Complete Remission/Response	NO		41395.00	MALE	NO	NO	Oligoastrocytoma
25	YES	YES	NO				Seizures	0 - 30 Days	Scheduled Follow-up Submission	Progressive Disease	YES	seafood	41395.00	MALE	YES	YES	Astrocytoma
26	NO	NO	NO				Seizures	> 181 Days	Scheduled Follow-up Submission		NO		41950.00	MALE	NO	NO	Oligodendroglioma
27	NO	NO	NO				Seizures	> 181 Days	Scheduled Follow-up Submission	Complete Remission/Response	NO		41947.00	MALE	NO	NO	Oligodendroglioma
28	NO	NO	NO				Seizures	0 - 30 Days	Scheduled Follow-up Submission	Complete Remission/Response	NO		41395.00	MALE	NO	NO	Oligoastrocytoma
29	NO	NO	NO				Seizures	91 - 180 Days	Scheduled Follow-up Submission	Stable Disease	NO		41395.00	MALE	NO	NO	Oligoastrocytoma
30	NO	YES	NO				Seizures	> 181 Days	Scheduled Follow-up Submission	Complete Remission/Response			41809.00	FEMALE	NO	NO	Oligoastrocytoma
31	NO	NO	NO				Seizures	> 181 Days	Scheduled Follow-up Submission		NO		41809.00	MALE	NO	NO	Oligoastrocytoma
32	NO	NO	NO				Seizures	31 - 90 Days	Scheduled Follow-up Submission	Complete Remission/Response			41809.00	MALE		NO	Astrocytoma
33	NO	YES	NO				Seizures	0 - 30 Days	Scheduled Follow-up Submission	Stable Disease			41809.00	MALE		NO	Oligoastrocytoma
34	YES	NO	NO					0 - 30 Days	Scheduled Follow-up Submission	Complete Remission/Response			41809.00	MALE		NO	Astrocytoma

35	TCGA-DB-A4XE-01	0.00	TCGA-DB-A4XE-01	9.21	1139.00	0.00	days	TCGA-DB-A4XE	1139.00	0.00	days	1139.00	days	TCGA Lower Grade Glioma (LGG)	brain lower grade glioma	Brain					27.00	NO		NO	TCGA-DB-A4XE-F60953	TCGA-DB-A4XE	TCGA-DB-A4XE-01A			(10150.00)	635.00		0.00	1139.00		294.00	0.00	NO	NO	NO				Seizures	31 - 90 Days	Scheduled Follow-up Submission	Complete Remission/Response	NO		41809.00	FEMALE	NO	NO	Oligoastrocytoma
36	TCGA-DB-A4XF-01	0.00	TCGA-DB-A4XF-01	11.56	1021.00	0.00	days	TCGA-DB-A4XF	1021.00	0.00	days	1021.00	days	TCGA Lower Grade Glioma (LGG)	brain lower grade glioma	Brain					41.00			NO	TCGA-DB-A4XF-F60956	TCGA-DB-A4XF	TCGA-DB-A4XF-01A			(15066.00)	456.00		0.00	1021.00				NO	YES	NO				Seizures	0 - 30 Days	Scheduled Follow-up Submission	Complete Remission/Response			41809.00	FEMALE		YES	Astrocytoma
37	TCGA-DB-A4XG-01	0.00	TCGA-DB-A4XG-01	8.66	2219.00	0.00	days	TCGA-DB-A4XG	2219.00	0.00	days	2219.00	days	TCGA Lower Grade Glioma (LGG)	brain lower grade glioma	Brain					34.00	NO		NO	TCGA-DB-A4XG-F60957	TCGA-DB-A4XG	TCGA-DB-A4XG-01A			(12550.00)	2137.00		0.00	2219.00				NO	NO	NO				Seizures	0 - 30 Days	Scheduled Follow-up Submission		NO		41809.00	MALE	NO	NO	Oligodendroglioma
38	TCGA-DB-A4XH-01	0.00	TCGA-DB-A4XH-01	9.66	962.00	0.00	days	TCGA-DB-A4XH	962.00	0.00	days	962.00	days	TCGA Lower Grade Glioma (LGG)	brain lower grade glioma	Brain					53.00			NO	TCGA-DB-A4XH-F60959	TCGA-DB-A4XH	TCGA-DB-A4XH-01A			(19710.00)	397.00		0.00	962.00				NO	NO	NO				Seizures	0 - 30 Days	Scheduled Follow-up Submission	Complete Remission/Response			41842.00	FEMALE		NO	Oligoastrocytoma
39	TCGA-DB-A64L-01	0.00	TCGA-DB-A64L-01	9.61	736.00	0.00	days	TCGA-DB-A64L	724.00	1.00	days	736.00	days	TCGA Lower Grade Glioma (LGG)	brain lower grade glioma	Brain	NO	NO	NO	NO	67.00	NO		NO	TCGA-DB-A64L-F62609	TCGA-DB-A64L	TCGA-DB-A64L-01A			(24690.00)	289.00		0.00	736.00	724.00	8.00		NO	YES	NO				Seizures	0 - 30 Days	Scheduled Follow-up Submission	Stable Disease			41950.00	FEMALE	NO	NO	Oligodendroglioma
40	TCGA-DB-A64O-01	1.00	TCGA-DB-A64O-01	11.56	775.00	1.00	days	TCGA-DB-A64O			days	775.00	days	TCGA Lower Grade Glioma (LGG)	brain lower grade glioma	Brain					59.00			NO	TCGA-DB-A64O-F62610	TCGA-DB-A64O	TCGA-DB-A64O-01A			(21811.00)	533.00	775.00	0.00	573.00				NO	YES	NO				Motor/Movement Changes	91 - 180 Days	Scheduled Follow-up Submission				41842.00	MALE	NO	NO	Oligoastrocytoma
41	TCGA-DB-A64P-01	0.00	TCGA-DB-A64P-01	10.87	916.00	0.00	days	TCGA-DB-A64P	916.00	0.00	days	916.00	days	TCGA Lower Grade Glioma (LGG)	brain lower grade glioma	Brain					40.00			NO	TCGA-DB-A64P-F62612	TCGA-DB-A64P	TCGA-DB-A64P-01A			(14936.00)	443.00		0.00	916.00				NO	YES	NO				Seizures	0 - 30 Days	Scheduled Follow-up Submission	Complete Remission/Response			41842.00	MALE	NO	NO	Oligodendroglioma
42	TCGA-DB-A64Q-01	0.00	TCGA-DB-A64Q-01	9.38	182.00	0.00	days	TCGA-DB-A64Q	182.00	0.00	days	182.00	days	TCGA Lower Grade Glioma (LGG)	brain lower grade glioma	Brain					31.00			NO	TCGA-DB-A64Q-F62614	TCGA-DB-A64Q	TCGA-DB-A64Q-01A			(11637.00)	251.00		0.00	182.00		(3.00)		NO	NO	NO				Seizures	31 - 90 Days	Scheduled Follow-up Submission				41842.00	FEMALE	NO	NO	Oligoastrocytoma
43	TCGA-DB-A64R-01	0.00	TCGA-DB-A64R-01	8.05	955.00	0.00	days	TCGA-DB-A64R	955.00	0.00	days	955.00	days	TCGA Lower Grade Glioma (LGG)	brain lower grade glioma	Brain					24.00	YES		NO	TCGA-DB-A64R-F62622	TCGA-DB-A64R	TCGA-DB-A64R-01A			(8947.00)	436.00		0.00	955.00		(6.00)		NO	NO	NO				Seizures	91 - 180 Days	Scheduled Follow-up Submission	Complete Remission/Response	YES	lactose	41947.00	FEMALE		NO	Oligodendroglioma
44	TCGA-DB-A64S-01	0.00	TCGA-DB-A64S-01	10.89	835.00	0.00	days	TCGA-DB-A64S	835.00	0.00	days	835.00	days	TCGA Lower Grade Glioma (LGG)	brain lower grade glioma	Brain					20.00	NO		NO	TCGA-DB-A64S-F67351	TCGA-DB-A64S	TCGA-DB-A64S-01A			(7389.00)	352.00		0.00	835.00		(29.00)		NO	NO	NO						Scheduled Follow-up Submission	Complete Remission/Response			41947.00	MALE	NO	NO	Oligoastrocytoma
45	TCGA-DB-A64U-01	0.00	TCGA-DB-A64U-01	9.75	846.00	0.00	days	TCGA-DB-A64U	846.00	0.00	days	846.00	days	TCGA Lower Grade Glioma (LGG)	brain lower grade glioma	Brain					38.00	YES	ant stings	NO	TCGA-DB-A64U-F67353	TCGA-DB-A64U	TCGA-DB-A64U-01A			(14019.00)	806.00		0.00	846.00		118.00		NO	NO	NO						Scheduled Follow-up Submission		YES	shellfish	41947.00	FEMALE	NO	NO	Oligoastrocytoma
46	TCGA-DB-A64V-01	0.00	TCGA-DB-A64V-01	11.07	889.00	0.00	days	TCGA-DB-A64V	889.00	0.00	days	889.00	days	TCGA Lower Grade Glioma (LGG)	brain lower grade glioma	Brain					54.00			NO	TCGA-DB-A64V-F67368	TCGA-DB-A64V	TCGA-DB-A64V-01A			(19950.00)	352.00		0.00	889.00		(17.00)		NO	YES	NO				Seizures	0 - 30 Days	Scheduled Follow-up Submission	Complete Remission/Response			41947.00	MALE	NO	NO	Oligodendroglioma
47	TCGA-DB-A64W-01	1.00	TCGA-DB-A64W-01	8.42	438.00	1.00	days	TCGA-DB-A64W	438.00	0.00	days	438.00	days	TCGA Lower Grade Glioma (LGG)	brain lower grade glioma	Brain					65.00			NO	TCGA-DB-A64W-F62623	TCGA-DB-A64W	TCGA-DB-A64W-01A			(23936.00)	289.00	438.00	0.00	415.00		(20.00)		NO						Seizures	> 181 Days	Scheduled Follow-up Submission	Stable Disease			41842.00	FEMALE		NO	Oligoastrocytoma
48	TCGA-DB-A64X-01	0.00	TCGA-DB-A64X-01	10.18	563.00	0.00	days	TCGA-DB-A64X	563.00	0.00	days	563.00	days	TCGA Lower Grade Glioma (LGG)	brain lower grade glioma	Brain					56.00			NO	TCGA-DB-A64X-F67369	TCGA-DB-A64X	TCGA-DB-A64X-01A			(20625.00)	247.00		0.00	563.00		(19.00)		NO	YES	NO				Motor/Movement Changes	91 - 180 Days	Scheduled Follow-up Submission	Complete Remission/Response			41950.00	FEMALE	NO	NO	Astrocytoma
49	TCGA-DB-A75K-01	0.00	TCGA-DB-A75K-01	10.03	368.00	0.00	days	TCGA-DB-A75K	368.00	0.00	days	368.00	days	TCGA Lower Grade Glioma (LGG)	brain lower grade glioma	Brain					55.00	NO		NO	TCGA-DB-A75K-F67635	TCGA-DB-A75K	TCGA-DB-A75K-01A			(20241.00)	114.00		0.00	368.00				NO	NO	NO				Seizures	0 - 30 Days	Scheduled Follow-up Submission	Complete Remission/Response	NO		41950.00	FEMALE	NO	NO	Oligoastrocytoma
50	TCGA-DB-A75L-01	0.00	TCGA-DB-A75L-01	9.77	342.00	0.00	days	TCGA-DB-A75L	342.00	0.00	days	342.00	days	TCGA Lower Grade Glioma (LGG)	brain lower grade glioma	Brain					36.00	NO		NO	TCGA-DB-A75L-F67636	TCGA-DB-A75L	TCGA-DB-A75L-01A			(13338.00)	75.00		0.00	342.00				NO	NO	NO				Seizures	91 - 180 Days	Scheduled Follow-up Submission	Complete Remission/Response	YES	lactose intolerance	41950.00	FEMALE	NO	NO	Astrocytoma
51	TCGA-DB-A75M-01	0.00	TCGA-DB-A75M-01	10.11	544.00	0.00	days	TCGA-DB-A75M	544.00	0.00	days	544.00	days	TCGA Lower Grade Glioma (LGG)	brain lower grade glioma	Brain					47.00	NO		NO	TCGA-DB-A75M-F67637	TCGA-DB-A75M	TCGA-DB-A75M-01A			(17289.00)	189.00		0.00	544.00				NO	YES	NO				Seizures	> 181 Days	Scheduled Follow-up Submission	Complete Remission/Response	NO		41950.00	MALE	NO	NO	Astrocytoma
52	TCGA-DB-A75O-01	0.00	TCGA-DB-A75O-01	10.80	935.00	0.00	days	TCGA-DB-A75O			days	935.00	days	TCGA Lower Grade Glioma (LGG)	brain lower grade glioma	Brain					29.00	NO		NO	TCGA-DB-A75O-F67378	TCGA-DB-A75O	TCGA-DB-A75O-01A			(10812.00)	981.00		0.00	935.00				NO	NO	NO				Seizures	31 - 90 Days	Scheduled Follow-up Submission		NO		41947.00	MALE	NO	NO	Astrocytoma
53	TCGA-DB-A75P-01	0.00	TCGA-DB-A75P-01	9.71	492.00	0.00	days	TCGA-DB-A75P	492.00	0.00	days	492.00	days	TCGA Lower Grade Glioma (LGG)	brain lower grade glioma	Brain					25.00	NO		NO	TCGA-DB-A75P-F67380	TCGA-DB-A75P	TCGA-DB-A75P-01A			(9413.00)	163.00		0.00	492.00				NO	NO	NO				Seizures	31 - 90 Days	Scheduled Follow-up Submission	Complete Remission/Response	NO		41947.00	FEMALE	NO	NO	Astrocytoma
54	TCGA-DH-5140-01	1.00	TCGA-DH-5140-01	6.48	607.00	1.00	days	TCGA-DH-5140	607.00	0.00	days	607.00	days	TCGA Lower Grade Glioma (LGG)	brain lower grade glioma	Brain					38.00	NO		NO	TCGA-DH-5140-F6484	TCGA-DH-5140	TCGA-DH-5140-01A			(13943.00)		607.00	0.00			259.00	1.00	NO	YES	NO				Headaches	91 - 180 Days		Progressive Disease	NO		40520.00	FEMALE	NO	YES	Oligoastrocytoma
55	TCGA-DH-5141-01	0.00	TCGA-DH-5141-01	9.78	968.00	0.00	days	TCGA-DH-5141			days	968.00	days	TCGA Lower Grade Glioma (LGG)	brain lower grade glioma	Brain					32.00	NO		NO	TCGA-DH-5141-F66668	TCGA-DH-5141	TCGA-DH-5141-01A			(11846.00)			0.00	968.00				NO	YES	NO				Headaches		Scheduled Follow-up Submission		NO		41934.00	MALE	NO	YES	Oligodendroglioma
56	TCGA-DH-5142-01	0.00	TCGA-DH-5142-01	9.27	1943.00	0.00	days	TCGA-DH-5142	1627.00	1.00	days	1943.00	days	TCGA Lower Grade Glioma (LGG)	brain lower grade glioma	Brain	YES	NO	NO		29.00	NO		YES	TCGA-DH-5142-F66689	TCGA-DH-5142	TCGA-DH-5142-01A			(10678.00)			0.00	1943.00	1627.00	10.00	0.00	NO						Seizures	0 - 30 Days	Scheduled Follow-up Submission		NO		41934.00	MALE	NO	NO	Astrocytoma
57	TCGA-DH-5143-01	0.00	TCGA-DH-5143-01	9.82	1401.00	0.00	days	TCGA-DH-5143	1401.00	0.00	days	1401.00	days	TCGA Lower Grade Glioma (LGG)	brain lower grade glioma	Brain					30.00				TCGA-DH-5143-F66710	TCGA-DH-5143	TCGA-DH-5143-01A			(11291.00)			0.00	1401.00		13.00	1.00							Seizures	91 - 180 Days	Scheduled Follow-up Submission	Stable Disease			41934.00	MALE		NO	Oligoastrocytoma
58	TCGA-DH-5144-01	0.00	TCGA-DH-5144-01	8.57	748.00	0.00	days	TCGA-DH-5144	748.00	0.00	days	748.00	days	TCGA Lower Grade Glioma (LGG)	brain lower grade glioma	Brain					56.00	NO		NO	TCGA-DH-5144-F66726	TCGA-DH-5144	TCGA-DH-5144-01A			(20558.00)			0.00	748.00		19.00	0.00	NO		NO				Seizures	0 - 30 Days	Scheduled Follow-up Submission	Stable Disease	NO		41934.00	FEMALE	NO	NO	Oligodendroglioma
59	TCGA-DH-A669-01	1.00	TCGA-DH-A669-01	12.01	919.00	1.00	days	TCGA-DH-A669			days	919.00	days	TCGA Lower Grade Glioma (LGG)	brain lower grade glioma	Brain					70.00			NO		TCGA-DH-A669				(25809.00)		919.00	0.00					NO	NO	NO				Mental Status Changes	> 181 Days			NO		41450.00	MALE	NO	NO	Oligodendroglioma
60	TCGA-DH-A669-02	1.00	TCGA-DH-A669-02	11.54	919.00	1.00	days	TCGA-DH-A669			days	919.00	days	TCGA Lower Grade Glioma (LGG)	brain lower grade glioma	Brain					70.00			NO		TCGA-DH-A669	TCGA-DH-A669-02A			(25809.00)	2274.00	919.00	0.00					NO	NO	NO				Mental Status Changes	> 181 Days			NO		41450.00	MALE	NO	NO	Oligodendroglioma
61	TCGA-DH-A66B-01	0.00	TCGA-DH-A66B-01	9.95	1279.00	0.00	days	TCGA-DH-A66B	1279.00	0.00	days	1279.00	days	TCGA Lower Grade Glioma (LGG)	brain lower grade glioma	Brain					52.00			NO	TCGA-DH-A66B-F60629	TCGA-DH-A66B	TCGA-DH-A66B-01A			(19156.00)	845.00		0.00	1279.00		901.00	1.00	NO	YES	NO				Mental Status Changes	> 181 Days	Scheduled Follow-up Submission	Stable Disease	NO		41803.00	MALE	NO	NO	Astrocytoma
62	TCGA-DH-A66D-01	0.00	TCGA-DH-A66D-01	8.70	846.00	0.00	days	TCGA-DH-A66D	846.00	0.00	days	846.00	days	TCGA Lower Grade Glioma (LGG)	brain lower grade glioma	Brain					43.00			NO	TCGA-DH-A66D-F60638	TCGA-DH-A66D	TCGA-DH-A66D-01A			(15866.00)	404.00		0.00	846.00		404.00	1.00	NO	NO	NO				Seizures	0 - 30 Days	Scheduled Follow-up Submission	Stable Disease	YES	lactose, cheese, gluten	41803.00	FEMALE	NO	NO	Astrocytoma
63	TCGA-DH-A66F-01	0.00	TCGA-DH-A66F-01	10.82	523.00	0.00	days	TCGA-DH-A66F	523.00	0.00	days	523.00	days	TCGA Lower Grade Glioma (LGG)	brain lower grade glioma	Brain					49.00			NO	TCGA-DH-A66F-F68872	TCGA-DH-A66F	TCGA-DH-A66F-01A			(18193.00)	166.00		0.00	523.00		18.00	1.00	NO	NO	NO				Seizures	0 - 30 Days	Scheduled Follow-up Submission		NO		42075.00	MALE		NO	Oligodendroglioma
64	TCGA-DH-A66G-01	0.00	TCGA-DH-A66G-01	10.16	523.00	0.00	days	TCGA-DH-A66G	523.00	0.00	days	523.00	days	TCGA Lower Grade Glioma (LGG)	brain lower grade glioma	Brain					49.00			NO	TCGA-DH-A66G-F61145	TCGA-DH-A66G	TCGA-DH-A66G-01A			(18199.00)	77.00		0.00	523.00		19.00	1.00	NO	YES	NO				Headaches	> 181 Days	Scheduled Follow-up Submission	Partial Remission/Response	NO		41815.00	FEMALE	NO	YES	Oligodendroglioma
65	TCGA-DH-A7UR-01	0.00	TCGA-DH-A7UR-01	10.18	1650.00	0.00	days	TCGA-DH-A7UR	1650.00	0.00	days	1650.00	days	TCGA Lower Grade Glioma (LGG)	brain lower grade glioma	Brain					59.00			NO	TCGA-DH-A7UR-F64479	TCGA-DH-A7UR				(21803.00)			0.00	1650.00		1174.00	0.00	NO	NO	NO				Headaches	> 181 Days	Scheduled Follow-up Submission		NO		41885.00	FEMALE	NO	YES	Oligodendroglioma
66	TCGA-DH-A7US-01	0.00	TCGA-DH-A7US-01	9.49	706.00	0.00	days	TCGA-DH-A7US	706.00	0.00	days	706.00	days	TCGA Lower Grade Glioma (LGG)	brain lower grade glioma	Brain					50.00			NO	TCGA-DH-A7US-F70994	TCGA-DH-A7US	TCGA-DH-A7US-01A			(18429.00)	208.00		0.00	706.00				NO	NO	NO				Seizures	> 181 Days	Scheduled Follow-up Submission	Stable Disease	NO		42075.00	MALE	NO	NO	Oligodendroglioma
67	TCGA-DH-A7UT-01	1.00	TCGA-DH-A7UT-01	9.76	531.00	1.00	days	TCGA-DH-A7UT	407.00	1.00	days	531.00	days	TCGA Lower Grade Glioma (LGG)	brain lower grade glioma	Brain					30.00			NO	TCGA-DH-A7UT-F70996	TCGA-DH-A7UT	TCGA-DH-A7UT-01A			(11014.00)	118.00	531.00	0.00	200.00	407.00			NO	NO	NO				Seizures	31 - 90 Days	Scheduled Follow-up Submission		NO		42075.00	MALE	NO	NO	Astrocytoma
68	TCGA-DH-A7UU-01	0.00	TCGA-DH-A7UU-01	10.37	417.00	0.00	days	TCGA-DH-A7UU	417.00	0.00	days	417.00	days	TCGA Lower Grade Glioma (LGG)	brain lower grade glioma	Brain					43.00			NO	TCGA-DH-A7UU-F66738	TCGA-DH-A7UU				(16037.00)			0.00	417.00		18.00		NO	YES	NO				Sensory Changes	91 - 180 Days	Scheduled Follow-up Submission	Stable Disease	NO		41934.00	MALE	YES	NO	Astrocytoma
69	TCGA-DH-A7UV-01	0.00	TCGA-DH-A7UV-01	10.18	566.00	0.00	days	TCGA-DH-A7UV	566.00	0.00	days	566.00	days	TCGA Lower Grade Glioma (LGG)	brain lower grade glioma	Brain					49.00				TCGA-DH-A7UV-F68876	TCGA-DH-A7UV				(18161.00)			0.00	566.00		19.00			NO	NO				Headaches	91 - 180 Days	Scheduled Follow-up Submission	Stable Disease			41995.00	MALE		YES	Astrocytoma
70	TCGA-DU-5847-01	0.00	TCGA-DU-5847-01	8.90	548.00	0.00	days	TCGA-DU-5847	548.00	0.00	days	548.00	days	TCGA Lower Grade Glioma (LGG)	brain lower grade glioma	Brain					34.00	NO		NO	TCGA-DU-5847-F14822	TCGA-DU-5847	TCGA-DU-5847-01A			(12606.00)			0.00	548.00				YES	NO	NO				Seizures	0 - 30 Days			NO		40688.00	FEMALE		NO	Astrocytoma

71	TCGA-DU-5849-01	0.00	TCGA-DU-5849-01	10.46	443.00	0.00	days	TCGA-DU-5849	443.00	0.00	days	443.00	days	TCGA Lower Grade Glioma (LGG)	brain lower grade glioma	Brain					48.00				TCGA-DU-5849-F14889	TCGA-DU-5849	TCGA-DU-5849-01A			(17663.00)			0.00	443.00						NO				Seizures	0 - 30 Days					40688.00	MALE		NO	Oligodendroglioma
72	TCGA-DU-5851-01	0.00	TCGA-DU-5851-01	9.84	531.00	0.00	days	TCGA-DU-5851	531.00	0.00	days	531.00	days	TCGA Lower Grade Glioma (LGG)	brain lower grade glioma	Brain					40.00				TCGA-DU-5851-F15071	TCGA-DU-5851	TCGA-DU-5851-01A			(14802.00)			0.00	531.00		17.00								Seizures	0 - 30 Days					40763.00	FEMALE		NO	Oligoastrocytoma
73	TCGA-DU-5852-01	1.00	TCGA-DU-5852-01	11.10	205.00	1.00	days	TCGA-DU-5852	24.00	1.00	days	205.00	days	TCGA Lower Grade Glioma (LGG)	brain lower grade glioma	Brain	YES	YES	NO	NO	61.00				TCGA-DU-5852-F17044	TCGA-DU-5852	TCGA-DU-5852-01A			(22348.00)		205.00	0.00		24.00	26.00								Seizures	> 181 Days		Progressive Disease			40812.00	FEMALE		NO	Oligoastrocytoma
74	TCGA-DU-5853-01	0.00	TCGA-DU-5853-01	9.59	407.00	0.00	days	TCGA-DU-5853	407.00	0.00	days	407.00	days	TCGA Lower Grade Glioma (LGG)	brain lower grade glioma	Brain					29.00				TCGA-DU-5853-F16881	TCGA-DU-5853	TCGA-DU-5853-01A			(10624.00)			0.00	407.00		18.00			YES	NO				Seizures	0 - 30 Days					40806.00	MALE		NO	Oligoastrocytoma
75	TCGA-DU-5854-01	0.00	TCGA-DU-5854-01	11.68	257.00	0.00	days	TCGA-DU-5854	202.00	1.00	days	257.00	days	TCGA Lower Grade Glioma (LGG)	brain lower grade glioma	Brain	YES	NO	NO	NO	57.00				TCGA-DU-5854-F14893	TCGA-DU-5854	TCGA-DU-5854-01A			(21106.00)			0.00	257.00	202.00	202.00			YES	NO				Motor/Movement Changes	91 - 180 Days					40688.00	FEMALE		NO	Astrocytoma
76	TCGA-DU-5855-01	0.00	TCGA-DU-5855-01	9.41	207.00	0.00	days	TCGA-DU-5855	207.00	0.00	days	207.00	days	TCGA Lower Grade Glioma (LGG)	brain lower grade glioma	Brain					49.00				TCGA-DU-5855-F14801	TCGA-DU-5855	TCGA-DU-5855-01A			(18234.00)			0.00	207.00		19.00			NO	NO				Headaches	0 - 30 Days					40688.00	FEMALE		YES	Oligoastrocytoma
77	TCGA-DU-5870-01	0.00	TCGA-DU-5870-01	10.32	5546.00	0.00	days	TCGA-DU-5870	4347.00	1.00	days	5546.00	days	TCGA Lower Grade Glioma (LGG)	brain lower grade glioma	Brain	YES	YES	YES	NO	34.00				TCGA-DU-5870-F14719	TCGA-DU-5870	TCGA-DU-5870-01A	4437.00		(12764.00)			0.00	5546.00	4347.00	4671.00			NO					Headaches	> 181 Days		Stable Disease			40751.00	FEMALE		YES	Oligodendroglioma
78	TCGA-DU-5870-02	0.00	TCGA-DU-5870-02	10.14	5546.00	0.00	days	TCGA-DU-5870	4347.00	1.00	days	5546.00	days	TCGA Lower Grade Glioma (LGG)	brain lower grade glioma	Brain	YES	YES	YES	NO	34.00				TCGA-DU-5870-F14719	TCGA-DU-5870	TCGA-DU-5870-02A	4437.00		(12764.00)	6469.00		0.00	5546.00	4347.00	4671.00			NO					Headaches	> 181 Days		Stable Disease			40751.00	FEMALE		YES	Oligodendroglioma
79	TCGA-DU-5871-01	0.00	TCGA-DU-5871-01	7.96	576.00	0.00	days	TCGA-DU-5871	576.00	0.00	days	576.00	days	TCGA Lower Grade Glioma (LGG)	brain lower grade glioma	Brain	NO	NO	NO	NO	37.00				TCGA-DU-5871-F15057	TCGA-DU-5871	TCGA-DU-5871-01A			(13673.00)			0.00	576.00		(6.00)			YES	NO				Seizures	0 - 30 Days					40690.00	FEMALE		NO	Oligoastrocytoma
80	TCGA-DU-5872-01	0.00	TCGA-DU-5872-01	11.25	532.00	0.00	days	TCGA-DU-5872	265.00	1.00	days	532.00	days	TCGA Lower Grade Glioma (LGG)	brain lower grade glioma	Brain	YES	NO	YES	NO	43.00				TCGA-DU-5872-F14771	TCGA-DU-5872	TCGA-DU-5872-01A	283.00		(15986.00)			0.00	532.00	265.00				NO	NO				Headaches	0 - 30 Days		Progressive Disease			40714.00	FEMALE		YES	Oligoastrocytoma
81	TCGA-DU-5872-02	0.00	TCGA-DU-5872-02	11.54	532.00	0.00	days	TCGA-DU-5872	265.00	1.00	days	532.00	days	TCGA Lower Grade Glioma (LGG)	brain lower grade glioma	Brain	YES	NO	YES	NO	43.00				TCGA-DU-5872-F14771	TCGA-DU-5872	TCGA-DU-5872-02A	283.00		(15986.00)	1317.00		0.00	532.00	265.00				NO	NO				Headaches	0 - 30 Days		Progressive Disease			40714.00	FEMALE		YES	Oligoastrocytoma
82	TCGA-DU-5874-01	0.00	TCGA-DU-5874-01	9.03	461.00	0.00	days	TCGA-DU-5874	461.00	0.00	days	461.00	days	TCGA Lower Grade Glioma (LGG)	brain lower grade glioma	Brain					62.00				TCGA-DU-5874-F14706	TCGA-DU-5874	TCGA-DU-5874-01A			(22847.00)			0.00	461.00		380.00								Seizures	31 - 90 Days					40711.00	FEMALE		NO	Oligodendroglioma
83	TCGA-DU-6392-01	0.00	TCGA-DU-6392-01	9.49	6423.00	0.00	days	TCGA-DU-6392	950.00	1.00	days	6423.00	days	TCGA Lower Grade Glioma (LGG)	brain lower grade glioma	Brain	NO	NO	YES	YES	35.00				TCGA-DU-6392-F14726	TCGA-DU-6392	TCGA-DU-6392-01A	950.00	1055.00	(12836.00)			0.00	6423.00	950.00									Seizures	0 - 30 Days		Stable Disease			40690.00	FEMALE			Astrocytoma
84	TCGA-DU-6393-01	1.00	TCGA-DU-6393-01	9.83	1585.00	1.00	days	TCGA-DU-6393			days	1585.00	days	TCGA Lower Grade Glioma (LGG)	brain lower grade glioma	Brain					66.00				TCGA-DU-6393-F14735	TCGA-DU-6393	TCGA-DU-6393-01A			(24282.00)		1585.00	0.00																	40690.00	MALE			Oligodendroglioma
85	TCGA-DU-6394-01	1.00	TCGA-DU-6394-01	9.64	682.00	1.00	days	TCGA-DU-6394	353.00	1.00	days	682.00	days	TCGA Lower Grade Glioma (LGG)	brain lower grade glioma	Brain	YES	YES	NO	NO	53.00				TCGA-DU-6394-F14761	TCGA-DU-6394	TCGA-DU-6394-01A			(19541.00)		682.00	0.00		353.00												Progressive Disease			40690.00	MALE			Oligodendroglioma
86	TCGA-DU-6395-01	1.00	TCGA-DU-6395-01	9.22	1491.00	1.00	days	TCGA-DU-6395	1197.00	1.00	days	1491.00	days	TCGA Lower Grade Glioma (LGG)	brain lower grade glioma	Brain	YES	NO	YES	NO	31.00				TCGA-DU-6395-F14942	TCGA-DU-6395	TCGA-DU-6395-01A	1197.00		(11584.00)		1491.00	0.00		1197.00				NO	NO				Seizures	91 - 180 Days		Progressive Disease			40689.00	MALE		NO	Oligoastrocytoma
87	TCGA-DU-6396-01	1.00	TCGA-DU-6396-01	9.46	2286.00	1.00	days	TCGA-DU-6396	106.00	1.00	days	2286.00	days	TCGA Lower Grade Glioma (LGG)	brain lower grade glioma	Brain	YES	YES	YES	NO	31.00				TCGA-DU-6396-F14916	TCGA-DU-6396	TCGA-DU-6396-01A	169.00		(11427.00)		2286.00	0.00		106.00	19.00	0.00		NO	NO				Motor/Movement Changes	0 - 30 Days		Progressive Disease			40751.00	FEMALE		YES	Oligoastrocytoma
88	TCGA-DU-6397-01	1.00	TCGA-DU-6397-01	10.39	1401.00	1.00	days	TCGA-DU-6397	837.00	1.00	days	1401.00	days	TCGA Lower Grade Glioma (LGG)	brain lower grade glioma	Brain	YES	YES	YES	NO	45.00				TCGA-DU-6397-F14794	TCGA-DU-6397	TCGA-DU-6397-01A	837.00		(16695.00)		1401.00	0.00		837.00				YES	NO				Headaches	0 - 30 Days		Progressive Disease			40689.00	MALE		YES	Oligodendroglioma
89	TCGA-DU-6397-02	1.00	TCGA-DU-6397-02	11.48	1401.00	1.00	days	TCGA-DU-6397	837.00	1.00	days	1401.00	days	TCGA Lower Grade Glioma (LGG)	brain lower grade glioma	Brain	YES	YES	YES	NO	45.00				TCGA-DU-6397-F14794	TCGA-DU-6397	TCGA-DU-6397-02A	837.00		(16695.00)	5830.00	1401.00	0.00		837.00				YES	NO				Headaches	0 - 30 Days		Progressive Disease			40689.00	MALE		YES	Oligodendroglioma
90	TCGA-DU-6399-01	1.00	TCGA-DU-6399-01	9.84	2000.00	1.00	days	TCGA-DU-6399	1629.00	1.00	days	2000.00	days	TCGA Lower Grade Glioma (LGG)	brain lower grade glioma	Brain	NO	NO	YES	NO	54.00				TCGA-DU-6399-F14710	TCGA-DU-6399	TCGA-DU-6399-01A	1629.00		(19906.00)		2000.00	0.00		1629.00					NO					31 - 90 Days		Progressive Disease			40694.00	MALE		NO	Oligodendroglioma
91	TCGA-DU-6400-01	1.00	TCGA-DU-6400-01	9.64	37.00	1.00	days	TCGA-DU-6400	37.00	0.00	days	37.00	days	TCGA Lower Grade Glioma (LGG)	brain lower grade glioma	Brain					66.00				TCGA-DU-6400-F14782	TCGA-DU-6400	TCGA-DU-6400-01A			(24122.00)		37.00	0.00											Mental Status Changes	91 - 180 Days					40694.00	FEMALE		YES	Oligodendroglioma
92	TCGA-DU-6401-01	1.00	TCGA-DU-6401-01	8.37	2660.00	1.00	days	TCGA-DU-6401	1886.00	1.00	days	2660.00	days	TCGA Lower Grade Glioma (LGG)	brain lower grade glioma	Brain	YES	NO	YES	NO	31.00				TCGA-DU-6401-F14751	TCGA-DU-6401	TCGA-DU-6401-01A	2464.00		(11595.00)		2660.00	0.00		1886.00	2454.00								Headaches	> 181 Days		Progressive Disease			40694.00	FEMALE		YES	Oligodendroglioma
93	TCGA-DU-6402-01	1.00	TCGA-DU-6402-01	10.60	214.00	1.00	days	TCGA-DU-6402	103.00	1.00	days	214.00	days	TCGA Lower Grade Glioma (LGG)	brain lower grade glioma	Brain	NO	NO	NO		52.00				TCGA-DU-6402-F14714	TCGA-DU-6402	TCGA-DU-6402-01A			(19207.00)		214.00	0.00		103.00									Seizures	0 - 30 Days		Progressive Disease			40694.00	MALE			Astrocytoma
94	TCGA-DU-6403-01	1.00	TCGA-DU-6403-01	9.02	354.00	1.00	days	TCGA-DU-6403			days	354.00	days	TCGA Lower Grade Glioma (LGG)	brain lower grade glioma	Brain					60.00				TCGA-DU-6403-F14743	TCGA-DU-6403	TCGA-DU-6403-01A			(22031.00)		354.00	0.00			207.00								Headaches	0 - 30 Days					40694.00	FEMALE		YES	Oligoastrocytoma
95	TCGA-DU-6404-01	1.00	TCGA-DU-6404-01	10.95	4068.00	1.00	days	TCGA-DU-6404	1154.00	1.00	days	4068.00	days	TCGA Lower Grade Glioma (LGG)	brain lower grade glioma	Brain	YES	YES	YES	NO	24.00	NO		NO	TCGA-DU-6404-F14781	TCGA-DU-6404	TCGA-DU-6404-01A	1194.00		(9021.00)		4068.00	0.00		1154.00	1398.00		NO	YES	NO			12 - 20 Years	Seizures	31 - 90 Days		Progressive Disease	YES	beef	40694.00	FEMALE	NO	NO	Oligodendroglioma
96	TCGA-DU-6404-02	1.00	TCGA-DU-6404-02	9.05	4068.00	1.00	days	TCGA-DU-6404	1154.00	1.00	days	4068.00	days	TCGA Lower Grade Glioma (LGG)	brain lower grade glioma	Brain	YES	YES	YES	NO	24.00	NO		NO	TCGA-DU-6404-F14781	TCGA-DU-6404	TCGA-DU-6404-02A	1194.00		(9021.00)	4892.00	4068.00	0.00		1154.00	1398.00		NO	YES	NO			12 - 20 Years	Seizures	31 - 90 Days		Progressive Disease	YES	beef	40694.00	FEMALE	NO	NO	Oligodendroglioma
97	TCGA-DU-6405-01	1.00	TCGA-DU-6405-01	10.28	605.00	1.00	days	TCGA-DU-6405	486.00	1.00	days	605.00	days	TCGA Lower Grade Glioma (LGG)	brain lower grade glioma	Brain	YES	NO	NO		51.00				TCGA-DU-6405-F14683	TCGA-DU-6405	TCGA-DU-6405-01A			(18686.00)		605.00	0.00		486.00	571.00								Seizures	0 - 30 Days		Progressive Disease			40714.00	FEMALE			Astrocytoma
98	TCGA-DU-6406-01	1.00	TCGA-DU-6406-01	10.82	512.00	1.00	days	TCGA-DU-6406			days	512.00	days	TCGA Lower Grade Glioma (LGG)	brain lower grade glioma	Brain					59.00				TCGA-DU-6406-F14899	TCGA-DU-6406	TCGA-DU-6406-01A			(21634.00)		512.00	0.00											Motor/Movement Changes	91 - 180 Days					40695.00	FEMALE		NO	Oligoastrocytoma
99	TCGA-DU-6407-01	1.00	TCGA-DU-6407-01	8.76	2875.00	1.00	days	TCGA-DU-6407	2397.00	1.00	days	2875.00	days	TCGA Lower Grade Glioma (LGG)	brain lower grade glioma	Brain	YES	YES	YES	YES	35.00				TCGA-DU-6407-F14950	TCGA-DU-6407	TCGA-DU-6407-01A	2415.00	2797.00	(13021.00)		2875.00	0.00		2397.00	2838.00			NO	NO				Seizures			Progressive Disease			40714.00	FEMALE		NO	Oligodendroglioma
100	TCGA-DU-6407-02	1.00	TCGA-DU-6407-02	10.19	2875.00	1.00	days	TCGA-DU-6407	2397.00	1.00	days	2875.00	days	TCGA Lower Grade Glioma (LGG)	brain lower grade glioma	Brain	YES	YES	YES	YES	35.00				TCGA-DU-6407-F14950	TCGA-DU-6407	TCGA-DU-6407-02B	2415.00	2797.00	(13021.00)	4524.00	2875.00	0.00		2397.00	2838.00			NO	NO				Seizures			Progressive Disease			40714.00	FEMALE		NO	Oligodendroglioma
101	TCGA-DU-6408-01	1.00	TCGA-DU-6408-01	8.15	3470.00	1.00	days	TCGA-DU-6408	2097.00	1.00	days	3470.00	days	TCGA Lower Grade Glioma (LGG)	brain lower grade glioma	Brain	YES		YES	YES	23.00				TCGA-DU-6408-F14907	TCGA-DU-6408	TCGA-DU-6408-01A	2552.00	2552.00	(8752.00)		3470.00	0.00		2097.00					NO				Motor/Movement Changes	0 - 30 Days		Progressive Disease			40714.00	FEMALE		NO	Oligodendroglioma
102	TCGA-DU-6410-01	0.00	TCGA-DU-6410-01	9.12	242.00	0.00	days	TCGA-DU-6410	242.00	0.00	days	242.00	days	TCGA Lower Grade Glioma (LGG)	brain lower grade glioma	Brain					56.00				TCGA-DU-6410-F15160	TCGA-DU-6410	TCGA-DU-6410-01A			(20567.00)			0.00	242.00		35.00								Mental Status Changes	0 - 30 Days					40714.00	MALE			Oligodendroglioma
103	TCGA-DU-6542-01	0.00	TCGA-DU-6542-01	9.04	73.00	0.00	days	TCGA-DU-6542			days	73.00	days	TCGA Lower Grade Glioma (LGG)	brain lower grade glioma	Brain					25.00				TCGA-DU-6542-F46623	TCGA-DU-6542	TCGA-DU-6542-01A			(9380.00)			0.00	73.00										Visual Changes	31 - 90 Days	Scheduled Follow-up Submission				41499.00	MALE		YES	Oligoastrocytoma
104	TCGA-DU-7006-01	1.00	TCGA-DU-7006-01	8.63	349.00	1.00	days	TCGA-DU-7006			days	349.00	days	TCGA Lower Grade Glioma (LGG)	brain lower grade glioma	Brain					60.00				TCGA-DU-7006-F17927	TCGA-DU-7006	TCGA-DU-7006-01A			(21990.00)		349.00	0.00											Seizures						40841.00	FEMALE		NO	Astrocytoma
105	TCGA-DU-7007-01	1.00	TCGA-DU-7007-01	11.45	1915.00	1.00	days	TCGA-DU-7007	1915.00	0.00	days	1915.00	days	TCGA Lower Grade Glioma (LGG)	brain lower grade glioma	Brain					33.00			YES	TCGA-DU-7007-F17216	TCGA-DU-7007	TCGA-DU-7007-01A			(12245.00)		1915.00	0.00											Headaches	> 181 Days					40814.00	MALE		YES	Astrocytoma
106	TCGA-DU-7008-01	0.00	TCGA-DU-7008-01	8.85	4752.00	0.00	days	TCGA-DU-7008	330.00	1.00	days	4752.00	days	TCGA Lower Grade Glioma (LGG)	brain lower grade glioma	Brain	NO	YES	YES	NO	41.00				TCGA-DU-7008-F17219	TCGA-DU-7008	TCGA-DU-7008-01A	330.00		(15289.00)			0.00	4752.00	330.00									Seizures	> 181 Days		Stable Disease			40814.00	FEMALE		YES	Oligodendroglioma

107	TCGA-DU-7009-01	1.00	TCGA-DU-7009-01	8.68	4695.00	1.00	days	TCGA-DU-7009	2960.00	1.00	days	4695.00	days	TCGA Lower Grade Glioma (LGG)	brain lower grade glioma	Brain	YES	YES	YES		32.00				TCGA-DU-7009-F46593	TCGA-DU-7009	TCGA-DU-7009-01A	3091.00		(11854.00)		4695.00	0.00	4206.00	2960.00				NO	NO				Sensory Changes	31 - 90 Days	Additional New Tumor Event	Progressive Disease			41498.00	FEMALE		NO	Oligodendroglioma
108	TCGA-DU-7010-01	1.00	TCGA-DU-7010-01	9.62	456.00	1.00	days	TCGA-DU-7010	193.00	1.00	days	456.00	days	TCGA Lower Grade Glioma (LGG)	brain lower grade glioma	Brain			YES	NO	58.00				TCGA-DU-7010-F17059	TCGA-DU-7010	TCGA-DU-7010-01A	193.00		(21199.00)		456.00	0.00		193.00									Seizures	31 - 90 Days		Progressive Disease			40814.00	FEMALE		NO	Astrocytoma
109	TCGA-DU-7011-01	1.00	TCGA-DU-7011-01	10.82	3200.00	1.00	days	TCGA-DU-7011			days	3200.00	days	TCGA Lower Grade Glioma (LGG)	brain lower grade glioma	Brain					25.00				TCGA-DU-7011-F17074	TCGA-DU-7011	TCGA-DU-7011-01A			(9322.00)		3200.00	0.00			149.00								Seizures	91 - 180 Days					40814.00	MALE		NO	Oligoastrocytoma
110	TCGA-DU-7012-01	1.00	TCGA-DU-7012-01	12.45	199.00	1.00	days	TCGA-DU-7012	199.00	0.00	days	199.00	days	TCGA Lower Grade Glioma (LGG)	brain lower grade glioma	Brain	NO	NO			74.00				TCGA-DU-7012-F17089	TCGA-DU-7012	TCGA-DU-7012-01A			(27039.00)		199.00	0.00											Mental Status Changes	0 - 30 Days		Progressive Disease			40814.00	FEMALE		NO	Astrocytoma
111	TCGA-DU-7013-01	1.00	TCGA-DU-7013-01	8.99	269.00	1.00	days	TCGA-DU-7013	187.00	1.00	days	269.00	days	TCGA Lower Grade Glioma (LGG)	brain lower grade glioma	Brain	NO	NO	NO	NO	59.00				TCGA-DU-7013-F17092	TCGA-DU-7013	TCGA-DU-7013-01A			(21824.00)		269.00	0.00		187.00	211.00								Seizures	31 - 90 Days		Progressive Disease			40812.00	MALE		NO	Astrocytoma
112	TCGA-DU-7014-01	1.00	TCGA-DU-7014-01	9.36	3571.00	1.00	days	TCGA-DU-7014	3437.00	1.00	days	3571.00	days	TCGA Lower Grade Glioma (LGG)	brain lower grade glioma	Brain	NO	NO	NO	NO	59.00				TCGA-DU-7014-F17101	TCGA-DU-7014	TCGA-DU-7014-01A			(21865.00)		3571.00	0.00		3437.00	3558.00								Seizures	0 - 30 Days		Progressive Disease			40812.00	MALE		YES	Oligodendroglioma
113	TCGA-DU-7015-01	0.00	TCGA-DU-7015-01	9.69	2761.00	0.00	days	TCGA-DU-7015	591.00	1.00	days	2761.00	days	TCGA Lower Grade Glioma (LGG)	brain lower grade glioma	Brain	YES	YES	YES	NO	41.00				TCGA-DU-7015-F18631	TCGA-DU-7015	TCGA-DU-7015-01A	591.00		(15041.00)			0.00	2761.00	591.00	613.00			YES	NO				Seizures	0 - 30 Days		Progressive Disease			40855.00	FEMALE		NO	Oligodendroglioma
114	TCGA-DU-7018-01	1.00	TCGA-DU-7018-01	9.58	933.00	1.00	days	TCGA-DU-7018	338.00	1.00	days	933.00	days	TCGA Lower Grade Glioma (LGG)	brain lower grade glioma	Brain	YES	NO	YES	NO	57.00				TCGA-DU-7018-F17070	TCGA-DU-7018	TCGA-DU-7018-01A	844.00		(21019.00)		933.00	0.00		338.00	343.00								Seizures	> 181 Days		Progressive Disease			40809.00	FEMALE		NO	Oligodendroglioma
115	TCGA-DU-7019-01	0.00	TCGA-DU-7019-01	9.96	800.00	0.00	days	TCGA-DU-7019	800.00	0.00	days	800.00	days	TCGA Lower Grade Glioma (LGG)	brain lower grade glioma	Brain					39.00				TCGA-DU-7019-F17096	TCGA-DU-7019	TCGA-DU-7019-01A			(14279.00)			0.00	800.00		21.00								Headaches	0 - 30 Days					40812.00	MALE		YES	Oligoastrocytoma
116	TCGA-DU-7290-01	1.00	TCGA-DU-7290-01	8.45	315.00	1.00	days	TCGA-DU-7290			days	315.00	days	TCGA Lower Grade Glioma (LGG)	brain lower grade glioma	Brain					45.00				TCGA-DU-7290-F17541	TCGA-DU-7290	TCGA-DU-7290-01A			(16667.00)		315.00	0.00											Headaches	0 - 30 Days					40827.00	FEMALE		YES	Astrocytoma
117	TCGA-DU-7292-01	1.00	TCGA-DU-7292-01	9.60	242.00	1.00	days	TCGA-DU-7292	91.00	1.00	days	242.00	days	TCGA Lower Grade Glioma (LGG)	brain lower grade glioma	Brain	YES	YES	NO	NO	69.00			YES	TCGA-DU-7292-F17515	TCGA-DU-7292	TCGA-DU-7292-01A			(25266.00)		242.00	0.00		91.00						< 12 Years			Seizures	91 - 180 Days		Progressive Disease			40821.00	MALE		NO	Astrocytoma
118	TCGA-DU-7294-01	0.00	TCGA-DU-7294-01	9.11	2869.00	0.00	days	TCGA-DU-7294	2869.00	0.00	days	2869.00	days	TCGA Lower Grade Glioma (LGG)	brain lower grade glioma	Brain					53.00				TCGA-DU-7294-F17513	TCGA-DU-7294	TCGA-DU-7294-01A			(19616.00)			0.00	2869.00		793.00											Stable Disease			40821.00	FEMALE		NO	Oligodendroglioma
119	TCGA-DU-7298-01	1.00	TCGA-DU-7298-01	9.44	576.00	1.00	days	TCGA-DU-7298	200.00	1.00	days	576.00	days	TCGA Lower Grade Glioma (LGG)	brain lower grade glioma	Brain	NO	NO	YES	NO	38.00				TCGA-DU-7298-F17497	TCGA-DU-7298	TCGA-DU-7298-01A	546.00		(14205.00)		576.00	0.00		200.00	(2.00)								Headaches	0 - 30 Days					40828.00	FEMALE		YES	Astrocytoma
120	TCGA-DU-7299-01	1.00	TCGA-DU-7299-01	8.82	1339.00	1.00	days	TCGA-DU-7299	675.00	1.00	days	1339.00	days	TCGA Lower Grade Glioma (LGG)	brain lower grade glioma	Brain	YES	YES	NO	NO	33.00				TCGA-DU-7299-F18389	TCGA-DU-7299	TCGA-DU-7299-01A			(12266.00)		1339.00	0.00		675.00	428.00			NO	NO				Seizures	31 - 90 Days		Progressive Disease			40855.00	MALE		NO	Astrocytoma
121	TCGA-DU-7300-01	1.00	TCGA-DU-7300-01	10.45	1886.00	1.00	days	TCGA-DU-7300	317.00	1.00	days	1886.00	days	TCGA Lower Grade Glioma (LGG)	brain lower grade glioma	Brain	YES	YES	NO	NO	53.00				TCGA-DU-7300-F17524	TCGA-DU-7300	TCGA-DU-7300-01A			(19610.00)		1886.00	0.00		317.00	317.00			NO	NO				Seizures	0 - 30 Days		Progressive Disease			40825.00	FEMALE		NO	Oligodendroglioma
122	TCGA-DU-7301-01	1.00	TCGA-DU-7301-01	10.62	788.00	1.00	days	TCGA-DU-7301	366.00	1.00	days	788.00	days	TCGA Lower Grade Glioma (LGG)	brain lower grade glioma	Brain	YES	NO	NO	NO	53.00				TCGA-DU-7301-F17533	TCGA-DU-7301	TCGA-DU-7301-01A			(19644.00)		788.00	0.00		366.00	366.00								Seizures	> 181 Days		Progressive Disease			40828.00	MALE		NO	Oligodendroglioma
123	TCGA-DU-7302-01	0.00	TCGA-DU-7302-01	8.54	1834.00	0.00	days	TCGA-DU-7302	1374.00	1.00	days	1834.00	days	TCGA Lower Grade Glioma (LGG)	brain lower grade glioma	Brain	YES	NO	NO	NO	48.00				TCGA-DU-7302-F17543	TCGA-DU-7302	TCGA-DU-7302-01A			(17656.00)			0.00	1834.00	1374.00	1404.00								Seizures	0 - 30 Days		Progressive Disease			40827.00	FEMALE		NO	Oligodendroglioma
124	TCGA-DU-7304-01	1.00	TCGA-DU-7304-01	10.96	709.00	1.00	days	TCGA-DU-7304	309.00	1.00	days	709.00	days	TCGA Lower Grade Glioma (LGG)	brain lower grade glioma	Brain	YES	NO	YES	NO	43.00				TCGA-DU-7304-F17502	TCGA-DU-7304	TCGA-DU-7304-01A	332.00		(15931.00)		709.00	0.00		309.00	350.00			NO	NO				Seizures	0 - 30 Days		Progressive Disease			40820.00	MALE		NO	Oligoastrocytoma
125	TCGA-DU-7304-02	1.00	TCGA-DU-7304-02	8.78	709.00	1.00	days	TCGA-DU-7304	309.00	1.00	days	709.00	days	TCGA Lower Grade Glioma (LGG)	brain lower grade glioma	Brain	YES	NO	YES	NO	43.00				TCGA-DU-7304-F17502	TCGA-DU-7304	TCGA-DU-7304-02A	332.00		(15931.00)	2066.00	709.00	0.00		309.00	350.00			NO	NO				Seizures	0 - 30 Days		Progressive Disease			40820.00	MALE		NO	Oligoastrocytoma
126	TCGA-DU-7306-01	0.00	TCGA-DU-7306-01	10.70	1277.00	0.00	days	TCGA-DU-7306	1250.00	1.00	days	1277.00	days	TCGA Lower Grade Glioma (LGG)	brain lower grade glioma	Brain	YES	NO	NO	NO	67.00				TCGA-DU-7306-F17490	TCGA-DU-7306	TCGA-DU-7306-01A			(24800.00)			0.00	1277.00	1250.00	(11.00)								Seizures	0 - 30 Days					40827.00	MALE		NO	Oligoastrocytoma
127	TCGA-DU-7309-01	0.00	TCGA-DU-7309-01	9.86	84.00	0.00	days	TCGA-DU-7309	84.00	0.00	days	84.00	days	TCGA Lower Grade Glioma (LGG)	brain lower grade glioma	Brain	NO	NO	NO	NO	41.00				TCGA-DU-7309-F17509	TCGA-DU-7309	TCGA-DU-7309-01A			(15197.00)			0.00	84.00		11.00			YES	YES				Seizures	31 - 90 Days					40820.00	FEMALE		YES	Oligodendroglioma
128	TCGA-DU-8158-01	1.00	TCGA-DU-8158-01	12.06	155.00	1.00	days	TCGA-DU-8158			days	155.00	days	TCGA Lower Grade Glioma (LGG)	brain lower grade glioma	Brain					57.00					TCGA-DU-8158	TCGA-DU-8158-01A			(21129.00)		155.00	0.00			8.00								Mental Status Changes	0 - 30 Days					41075.00	FEMALE			Astrocytoma
129	TCGA-DU-8161-01	1.00	TCGA-DU-8161-01	8.51	722.00	1.00	days	TCGA-DU-8161	111.00	1.00	days	722.00	days	TCGA Lower Grade Glioma (LGG)	brain lower grade glioma	Brain	YES	NO	YES		63.00				TCGA-DU-8161-F51985	TCGA-DU-8161	TCGA-DU-8161-01A	113.00		(23064.00)		722.00	0.00		111.00	14.00								Sensory Changes	31 - 90 Days	Scheduled Follow-up Submission	Progressive Disease			41604.00	FEMALE		NO	Oligoastrocytoma
130	TCGA-DU-8162-01	1.00	TCGA-DU-8162-01	10.12	444.00	1.00	days	TCGA-DU-8162	402.00	1.00	days	444.00	days	TCGA Lower Grade Glioma (LGG)	brain lower grade glioma	Brain	NO	YES			61.00				TCGA-DU-8162-F44364	TCGA-DU-8162	TCGA-DU-8162-01A			(22596.00)		444.00	0.00	121.00	402.00	21.00	1.00		YES	NO				Headaches	0 - 30 Days	Scheduled Follow-up Submission				41444.00	FEMALE		YES	Oligoastrocytoma
131	TCGA-DU-8163-01	0.00	TCGA-DU-8163-01	9.64	629.00	0.00	days	TCGA-DU-8163	509.00	1.00	days	629.00	days	TCGA Lower Grade Glioma (LGG)	brain lower grade glioma	Brain	YES	NO			29.00				TCGA-DU-8163-F44371	TCGA-DU-8163	TCGA-DU-8163-01A			(10824.00)			0.00	629.00	509.00	106.00			NO	NO				Seizures	31 - 90 Days	Scheduled Follow-up Submission				41444.00	MALE		NO	Oligoastrocytoma
132	TCGA-DU-8164-01	0.00	TCGA-DU-8164-01	9.79	651.00	0.00	days	TCGA-DU-8164	651.00	0.00	days	651.00	days	TCGA Lower Grade Glioma (LGG)	brain lower grade glioma	Brain					51.00				TCGA-DU-8164-F44373	TCGA-DU-8164	TCGA-DU-8164-01A			(18735.00)			0.00	651.00					NO	NO				Seizures	31 - 90 Days	Scheduled Follow-up Submission	Stable Disease			41444.00	MALE		NO	Oligodendroglioma
133	TCGA-DU-8165-01	0.00	TCGA-DU-8165-01	10.12	582.00	0.00	days	TCGA-DU-8165	582.00	0.00	days	582.00	days	TCGA Lower Grade Glioma (LGG)	brain lower grade glioma	Brain					60.00				TCGA-DU-8165-F46668	TCGA-DU-8165	TCGA-DU-8165-01A			(21964.00)			0.00	582.00		11.00			YES	NO				Seizures	31 - 90 Days	Scheduled Follow-up Submission	Stable Disease			41499.00	FEMALE		NO	Oligodendroglioma
134	TCGA-DU-8166-01	0.00	TCGA-DU-8166-01	7.93	516.00	0.00	days	TCGA-DU-8166	193.00	1.00	days	516.00	days	TCGA Lower Grade Glioma (LGG)	brain lower grade glioma	Brain	YES	NO	NO		29.00				TCGA-DU-8166-F46665	TCGA-DU-8166	TCGA-DU-8166-01A			(10641.00)			0.00	516.00	193.00				NO	NO				Seizures	0 - 30 Days	Scheduled Follow-up Submission	Stable Disease			41499.00	FEMALE		NO	Oligoastrocytoma
135	TCGA-DU-8167-01	0.00	TCGA-DU-8167-01	9.65	471.00	0.00	days	TCGA-DU-8167	471.00	0.00	days	471.00	days	TCGA Lower Grade Glioma (LGG)	brain lower grade glioma	Brain					69.00				TCGA-DU-8167-F44357	TCGA-DU-8167	TCGA-DU-8167-01A			(25262.00)			0.00	471.00		(69.00)								Seizures	> 181 Days	Scheduled Follow-up Submission	Stable Disease			41444.00	FEMALE		NO	Oligoastrocytoma
136	TCGA-DU-8168-01	0.00	TCGA-DU-8168-01	8.06	431.00	0.00	days	TCGA-DU-8168	431.00	0.00	days	431.00	days	TCGA Lower Grade Glioma (LGG)	brain lower grade glioma	Brain					55.00				TCGA-DU-8168-F44360	TCGA-DU-8168	TCGA-DU-8168-01A			(20136.00)			0.00	431.00		14.00	1.00		YES	NO				Headaches	0 - 30 Days	Scheduled Follow-up Submission	Stable Disease			41444.00	FEMALE		YES	Oligodendroglioma
137	TCGA-DU-A5TP-01	0.00	TCGA-DU-A5TP-01	7.22	1012.00	0.00	days	TCGA-DU-A5TP	203.00	1.00	days	1012.00	days	TCGA Lower Grade Glioma (LGG)	brain lower grade glioma	Brain	YES	NO			33.00	NO		NO	TCGA-DU-A5TP-F71612	TCGA-DU-A5TP	TCGA-DU-A5TP-01A			(12111.00)	301.00		0.00	1012.00	203.00	6.00		YES	YES	YES	> 20 Years			Seizures	0 - 30 Days	Scheduled Follow-up Submission	Stable Disease	NO		42090.00	MALE	YES	NO	Astrocytoma
138	TCGA-DU-A5TR-01	0.00	TCGA-DU-A5TR-01	10.63	908.00	0.00	days	TCGA-DU-A5TR	848.00	1.00	days	908.00	days	TCGA Lower Grade Glioma (LGG)	brain lower grade glioma	Brain	NO	NO	YES		51.00	NO		NO	TCGA-DU-A5TR-F71611	TCGA-DU-A5TR	TCGA-DU-A5TR-01A	848.00		(18791.00)	259.00		0.00	908.00	848.00	11.00		NO	YES	YES				Seizures	0 - 30 Days	Scheduled Follow-up Submission	Stable Disease	NO		42090.00	MALE	NO	NO	Oligoastrocytoma
139	TCGA-DU-A5TS-01	0.00	TCGA-DU-A5TS-01	9.49	964.00	0.00	days	TCGA-DU-A5TS	551.00	1.00	days	964.00	days	TCGA Lower Grade Glioma (LGG)	brain lower grade glioma	Brain	YES	NO	NO	NO	42.00				TCGA-DU-A5TS-F71496	TCGA-DU-A5TS	TCGA-DU-A5TS-01A			(15650.00)	259.00		0.00	964.00	551.00	13.00			YES	NO				Headaches	> 181 Days	Scheduled Follow-up Submission	Stable Disease			42089.00	MALE		YES	Oligodendroglioma
140	TCGA-DU-A5TT-01	0.00	TCGA-DU-A5TT-01	10.74	743.00	0.00	days	TCGA-DU-A5TT	584.00	1.00	days	743.00	days	TCGA Lower Grade Glioma (LGG)	brain lower grade glioma	Brain	NO	YES	NO	NO	70.00	YES	cat	NO	TCGA-DU-A5TT-F71494	TCGA-DU-A5TT	TCGA-DU-A5TT-01A			(25726.00)	24.00		0.00	743.00	584.00			NO	YES	NO	> 20 Years	> 20 Years		Seizures	91 - 180 Days	Scheduled Follow-up Submission	Progressive Disease	NO		42089.00	MALE	YES	NO	Oligodendroglioma
141	TCGA-DU-A5TU-01	0.00	TCGA-DU-A5TU-01	9.18	796.00	0.00	days	TCGA-DU-A5TU	186.00	1.00	days	796.00	days	TCGA Lower Grade Glioma (LGG)	brain lower grade glioma	Brain	YES	NO	NO		62.00	NO		NO	TCGA-DU-A5TU-F71322	TCGA-DU-A5TU	TCGA-DU-A5TU-01A			(22811.00)	21.00		0.00	796.00	186.00	47.00		NO	YES	NO			12 - 20 Years	Mental Status Changes	0 - 30 Days	Scheduled Follow-up Submission	Stable Disease	YES	Eggs	42087.00	FEMALE	NO	NO	Astrocytoma
142	TCGA-DU-A5TW-01	0.00	TCGA-DU-A5TW-01	8.20	760.00	0.00	days	TCGA-DU-A5TW	760.00	0.00	days	760.00	days	TCGA Lower Grade Glioma (LGG)	brain lower grade glioma	Brain	NO	NO	NO		33.00				TCGA-DU-A5TW-F71321	TCGA-DU-A5TW	TCGA-DU-A5TW-01A			(12107.00)	42.00		0.00	760.00		53.00								Seizures	> 181 Days	Scheduled Follow-up Submission	Progressive Disease			42087.00	FEMALE		NO	Astrocytoma

143	TCGA-DU-A5TY-01	1.00	TCGA-DU-A5TY-01	10.89	1033.00	1.00	days	TCGA-DU-A5TY	526.00	1.00	days	1033.00	days	TCGA Lower Grade Glioma (LGG)	brain lower grade glioma	Brain	YES	YES	YES	YES	46.00			NO	TCGA-DU-A5TY-F71302	TCGA-DU-A5TY	TCGA-DU-A5TY-01A	536.00	955.00	(16945.00)	276.00	1033.00	0.00	371.00	526.00			NO	NO	NO				Seizures	0 - 30 Days	Scheduled Follow-up Submission	Progressive Disease	NO		42087.00	FEMALE	NO	NO	Astrocytoma
144	TCGA-DU-A6S2-01	0.00	TCGA-DU-A6S2-01	8.95	777.00	0.00	days	TCGA-DU-A6S2	777.00	0.00	days	777.00	days	TCGA Lower Grade Glioma (LGG)	brain lower grade glioma	Brain	NO	NO			37.00	YES	long hair cats	NO	TCGA-DU-A6S2-F71620	TCGA-DU-A6S2	TCGA-DU-A6S2-01A			(13597.00)	153.00		0.00	777.00		11.00		YES	YES	NO	> 20 Years	< 12 Years		Headaches	31 - 90 Days	Scheduled Follow-up Submission	Stable Disease	NO		42090.00	FEMALE	NO	YES	Oligodendroglioma
145	TCGA-DU-A6S3-01	0.00	TCGA-DU-A6S3-01	9.22	656.00	0.00	days	TCGA-DU-A6S3	656.00	0.00	days	656.00	days	TCGA Lower Grade Glioma (LGG)	brain lower grade glioma	Brain	NO	NO			60.00				TCGA-DU-A6S3-F71619	TCGA-DU-A6S3	TCGA-DU-A6S3-01A			(22038.00)	55.00		0.00	656.00										Motor/Movement Changes	91 - 180 Days	Scheduled Follow-up Submission				42090.00	MALE		NO	Oligodendroglioma
146	TCGA-DU-A6S6-01	0.00	TCGA-DU-A6S6-01	9.61	2893.00	0.00	days	TCGA-DU-A6S6	2277.00	1.00	days	2893.00	days	TCGA Lower Grade Glioma (LGG)	brain lower grade glioma	Brain	YES	NO	YES		35.00	NO		NO	TCGA-DU-A6S6-F71617	TCGA-DU-A6S6		2277.00		(12809.00)			0.00	2893.00	2277.00	184.00		NO	NO	NO	< 12 Years			Headaches	31 - 90 Days	Scheduled Follow-up Submission	Stable Disease	NO		42090.00	FEMALE	YES	YES	Oligoastrocytoma
147	TCGA-DU-A6S7-01	0.00	TCGA-DU-A6S7-01	9.84	638.00	0.00	days	TCGA-DU-A6S7	638.00	0.00	days	638.00	days	TCGA Lower Grade Glioma (LGG)	brain lower grade glioma	Brain	NO	NO			27.00	NO		NO	TCGA-DU-A6S7-F71615	TCGA-DU-A6S7				(10170.00)			0.00	638.00		(14.00)		NO	NO	NO	12 - 20 Years			Seizures	> 181 Days	Scheduled Follow-up Submission	Stable Disease	NO		42090.00	FEMALE	YES	NO	Astrocytoma
148	TCGA-DU-A6S8-01	0.00	TCGA-DU-A6S8-01	9.88	678.00	0.00	days	TCGA-DU-A6S8	641.00	1.00	days	678.00	days	TCGA Lower Grade Glioma (LGG)	brain lower grade glioma	Brain	NO	NO			74.00	NO		NO	TCGA-DU-A6S8-F71614	TCGA-DU-A6S8	TCGA-DU-A6S8-01A			(27084.00)	76.00		0.00	678.00	641.00	14.00		NO	NO	NO				Mental Status Changes	> 181 Days	Scheduled Follow-up Submission		NO		42090.00	FEMALE	NO	NO	Oligodendroglioma
149	TCGA-DU-A76K-01	1.00	TCGA-DU-A76K-01	11.09	347.00	1.00	days	TCGA-DU-A76K			days	347.00	days	TCGA Lower Grade Glioma (LGG)	brain lower grade glioma	Brain					87.00					TCGA-DU-A76K	TCGA-DU-A76K-01A			(31776.00)	5192.00	347.00	0.00											Headaches	> 181 Days					41569.00	MALE		YES	Oligodendroglioma
150	TCGA-DU-A76L-01	1.00	TCGA-DU-A76L-01	10.79	814.00	1.00	days	TCGA-DU-A76L			days	814.00	days	TCGA Lower Grade Glioma (LGG)	brain lower grade glioma	Brain					54.00					TCGA-DU-A76L	TCGA-DU-A76L-01A			(19823.00)	4769.00	814.00	0.00						YES					Headaches	0 - 30 Days					41569.00	MALE		YES	Oligodendroglioma
151	TCGA-DU-A76O-01	0.00	TCGA-DU-A76O-01	9.16	522.00	0.00	days	TCGA-DU-A76O	522.00	0.00	days	522.00	days	TCGA Lower Grade Glioma (LGG)	brain lower grade glioma	Brain					30.00	NO		NO	TCGA-DU-A76O-F71621	TCGA-DU-A76O	TCGA-DU-A76O-01A			(10960.00)	138.00		0.00	522.00				NO	NO	NO				Seizures	> 181 Days	Scheduled Follow-up Submission		NO		42090.00	MALE	NO	NO	Astrocytoma
152	TCGA-DU-A76R-01	1.00	TCGA-DU-A76R-01	9.67	648.00	1.00	days	TCGA-DU-A76R			days	648.00	days	TCGA Lower Grade Glioma (LGG)	brain lower grade glioma	Brain					51.00					TCGA-DU-A76R	TCGA-DU-A76R-01A			(18939.00)	2739.00	648.00	0.00			60.00								Mental Status Changes	0 - 30 Days					41569.00	MALE		NO	Oligodendroglioma
153	TCGA-DU-A7T6-01	1.00	TCGA-DU-A7T6-01	10.64	547.00	1.00	days	TCGA-DU-A7T6			days	547.00	days	TCGA Lower Grade Glioma (LGG)	brain lower grade glioma	Brain					73.00					TCGA-DU-A7T6	TCGA-DU-A7T6-01A			(26766.00)	4265.00	547.00	0.00			53.00								Headaches	> 181 Days					41631.00	FEMALE		YES	Oligodendroglioma
154	TCGA-DU-A7T8-01	1.00	TCGA-DU-A7T8-01	9.11	4229.00	1.00	days	TCGA-DU-A7T8	3009.00	1.00	days	4229.00	days	TCGA Lower Grade Glioma (LGG)	brain lower grade glioma	Brain	YES	NO	YES	YES	35.00	NO		NO	TCGA-DU-A7T8-F71629	TCGA-DU-A7T8	TCGA-DU-A7T8-01A	3028.00	3759.00	(13108.00)	3705.00	4229.00	0.00	3787.00	3009.00	80.00		YES	NO	NO	< 12 Years			Mental Status Changes	91 - 180 Days	Scheduled Follow-up Submission	Progressive Disease	NO		42090.00	MALE	NO	YES	Oligoastrocytoma
155	TCGA-DU-A7TA-01	0.00	TCGA-DU-A7TA-01	10.86	2565.00	0.00	days	TCGA-DU-A7TA	2565.00	0.00	days	2565.00	days	TCGA Lower Grade Glioma (LGG)	brain lower grade glioma	Brain					32.00	NO		NO	TCGA-DU-A7TA-F71628	TCGA-DU-A7TA	TCGA-DU-A7TA-01A			(11764.00)	2287.00		0.00	2565.00		17.00		NO	NO	NO	12 - 20 Years			Visual Changes	> 181 Days	Scheduled Follow-up Submission		NO		42090.00	MALE	YES	NO	Oligodendroglioma
156	TCGA-DU-A7TB-01	0.00	TCGA-DU-A7TB-01	9.46	1567.00	0.00	days	TCGA-DU-A7TB	74.00	1.00	days	1567.00	days	TCGA Lower Grade Glioma (LGG)	brain lower grade glioma	Brain	YES	YES	NO		56.00				TCGA-DU-A7TB-F71627	TCGA-DU-A7TB	TCGA-DU-A7TB-01A			(20704.00)	1168.00		0.00	1567.00	74.00	1111.00			NO	NO				Seizures	0 - 30 Days	Scheduled Follow-up Submission	Stable Disease			42090.00	MALE		NO	Oligodendroglioma
157	TCGA-DU-A7TC-01	0.00	TCGA-DU-A7TC-01	8.73	1137.00	0.00	days	TCGA-DU-A7TC	1114.00	1.00	days	1137.00	days	TCGA Lower Grade Glioma (LGG)	brain lower grade glioma	Brain					32.00			NO	TCGA-DU-A7TC-F71626	TCGA-DU-A7TC				(11884.00)			0.00	1137.00	1114.00	[Discrepancy]		NO	YES	NO				Headaches	> 181 Days	Scheduled Follow-up Submission				42090.00	MALE	NO	YES	Astrocytoma
158	TCGA-DU-A7TD-01	1.00	TCGA-DU-A7TD-01	11.28	228.00	1.00	days	TCGA-DU-A7TD			days	228.00	days	TCGA Lower Grade Glioma (LGG)	brain lower grade glioma	Brain					52.00					TCGA-DU-A7TD	TCGA-DU-A7TD-01A			(19025.00)	616.00	228.00	0.00			170.00								Motor/Movement Changes	0 - 30 Days					41673.00	MALE		NO	Oligoastrocytoma
159	TCGA-DU-A7TG-01	1.00	TCGA-DU-A7TG-01	9.22	1351.00	1.00	days	TCGA-DU-A7TG			days	1351.00	days	TCGA Lower Grade Glioma (LGG)	brain lower grade glioma	Brain					40.00					TCGA-DU-A7TG	TCGA-DU-A7TG-01A			(14671.00)	1804.00	1351.00	0.00			59.00			YES	NO				Mental Status Changes	0 - 30 Days					41673.00	MALE		NO	Oligodendroglioma
160	TCGA-DU-A7TI-01	1.00	TCGA-DU-A7TI-01	10.05	1183.00	1.00	days	TCGA-DU-A7TI			days	1183.00	days	TCGA Lower Grade Glioma (LGG)	brain lower grade glioma	Brain					32.00					TCGA-DU-A7TI	TCGA-DU-A7TI-01A			(11906.00)	5441.00	1183.00	0.00											Seizures	91 - 180 Days					41675.00	MALE		NO	Astrocytoma
161	TCGA-DU-A7TJ-01	0.00	TCGA-DU-A7TJ-01	9.83	17.00	0.00	days	TCGA-DU-A7TJ			days	17.00	days	TCGA Lower Grade Glioma (LGG)	brain lower grade glioma	Brain					55.00				TCGA-DU-A7TJ-F71624	TCGA-DU-A7TJ	TCGA-DU-A7TJ-01A			(20394.00)	29.00		0.00	17.00										Seizures	31 - 90 Days	Scheduled Follow-up Submission				42090.00	MALE		YES	Astrocytoma
162	TCGA-E1-5302-01	1.00	TCGA-E1-5302-01	9.09	1525.00	1.00	days	TCGA-E1-5302	1242.00	1.00	days	1525.00	days	TCGA Lower Grade Glioma (LGG)	brain lower grade glioma	Brain	YES	YES	NO	NO	41.00			NO	TCGA-E1-5302-F7572	TCGA-E1-5302	TCGA-E1-5302-01A			(15230.00)		1525.00	0.00		1242.00	1254.00		NO	YES	NO				Seizures	0 - 30 Days		Progressive Disease			40556.00	MALE		NO	Astrocytoma
163	TCGA-E1-5303-01	1.00	TCGA-E1-5303-01	10.20	2052.00	1.00	days	TCGA-E1-5303	156.00	1.00	days	2052.00	days	TCGA Lower Grade Glioma (LGG)	brain lower grade glioma	Brain	YES	NO	NO	NO	38.00			NO	TCGA-E1-5303-F7453	TCGA-E1-5303	TCGA-E1-5303-01A			(14212.00)		2052.00	0.00		156.00	582.00		NO	YES	NO				Seizures	0 - 30 Days		Progressive Disease			40562.00	MALE		NO	Astrocytoma
164	TCGA-E1-5304-01	1.00	TCGA-E1-5304-01	7.91	1251.00	1.00	days	TCGA-E1-5304	903.00	1.00	days	1251.00	days	TCGA Lower Grade Glioma (LGG)	brain lower grade glioma	Brain		YES	YES	NO	42.00	NO		NO	TCGA-E1-5304-F7470	TCGA-E1-5304	TCGA-E1-5304-01A	980.00		(15481.00)		1251.00	0.00		903.00	160.00		NO	YES	NO				Headaches	> 181 Days		Stable Disease	NO		40563.00	MALE	NO	YES	Astrocytoma
165	TCGA-E1-5305-01	1.00	TCGA-E1-5305-01	8.75	2433.00	1.00	days	TCGA-E1-5305	1604.00	1.00	days	2433.00	days	TCGA Lower Grade Glioma (LGG)	brain lower grade glioma	Brain	YES	NO	YES	NO	34.00			YES	TCGA-E1-5305-F16185	TCGA-E1-5305	TCGA-E1-5305-01A	1623.00		(12655.00)		2433.00	0.00		1604.00	1631.00		NO	YES	NO				Seizures	0 - 30 Days		Progressive Disease			40763.00	MALE	NO	NO	Astrocytoma
166	TCGA-E1-5307-01	1.00	TCGA-E1-5307-01	7.34	1762.00	1.00	days	TCGA-E1-5307	1452.00	1.00	days	1762.00	days	TCGA Lower Grade Glioma (LGG)	brain lower grade glioma	Brain	YES	YES	NO	NO	62.00			NO	TCGA-E1-5307-F16198	TCGA-E1-5307	TCGA-E1-5307-01A			(22837.00)		1762.00	0.00		1452.00	[Discrepancy]		NO						Mental Status Changes	> 181 Days		Progressive Disease			40767.00	FEMALE		YES	Astrocytoma
167	TCGA-E1-5311-01	1.00	TCGA-E1-5311-01	8.51	4084.00	1.00	days	TCGA-E1-5311	2602.00	1.00	days	4084.00	days	TCGA Lower Grade Glioma (LGG)	brain lower grade glioma	Brain	YES	YES	YES	NO	31.00	NO		NO	TCGA-E1-5311-F7600	TCGA-E1-5311	TCGA-E1-5311-01A	2602.00		(11332.00)		4084.00	0.00		2602.00	(8.00)		NO	NO	NO				Headaches	> 181 Days		Progressive Disease	NO		40568.00	MALE	NO	YES	Oligodendroglioma
168	TCGA-E1-5318-01	1.00	TCGA-E1-5318-01	10.97	2379.00	1.00	days	TCGA-E1-5318	362.00	1.00	days	2379.00	days	TCGA Lower Grade Glioma (LGG)	brain lower grade glioma	Brain	YES	YES	YES	NO	42.00	NO		NO	TCGA-E1-5318-F7616	TCGA-E1-5318	TCGA-E1-5318-01A	362.00		(15372.00)		2379.00	0.00	2379.00	362.00	362.00		NO	YES	NO				Headaches	31 - 90 Days		Stable Disease	NO		40569.00	FEMALE	NO	YES	Oligodendroglioma
169	TCGA-E1-5319-01	1.00	TCGA-E1-5319-01	10.42	2907.00	1.00	days	TCGA-E1-5319	923.00	1.00	days	2907.00	days	TCGA Lower Grade Glioma (LGG)	brain lower grade glioma	Brain	YES	YES	YES	NO	48.00			NO	TCGA-E1-5319-F16173	TCGA-E1-5319	TCGA-E1-5319-01A	923.00		(17612.00)		2907.00	0.00		923.00	952.00		NO	YES					Visual Changes	0 - 30 Days		Stable Disease			40772.00	FEMALE	NO	NO	Oligodendroglioma
170	TCGA-E1-5322-01	1.00	TCGA-E1-5322-01	9.24	3978.00	1.00	days	TCGA-E1-5322	1085.00	1.00	days	3978.00	days	TCGA Lower Grade Glioma (LGG)	brain lower grade glioma	Brain	YES	YES	YES	NO	38.00				TCGA-E1-5322-F7464	TCGA-E1-5322	TCGA-E1-5322-01A	1085.00		(13903.00)		3978.00	0.00		1085.00	3305.00			NO	NO				Seizures			Stable Disease			40570.00	FEMALE			Oligoastrocytoma
171	TCGA-E1-A7YD-01	1.00	TCGA-E1-A7YD-01	10.43	435.00	1.00	days	TCGA-E1-A7YD			days	435.00	days	TCGA Lower Grade Glioma (LGG)	brain lower grade glioma	Brain					57.00			NO		TCGA-E1-A7YD	TCGA-E1-A7YD-01A			(21020.00)	5445.00	435.00	0.00					NO	NO	NO				Headaches	0 - 30 Days					41771.00	MALE		YES	Astrocytoma
172	TCGA-E1-A7YE-01	1.00	TCGA-E1-A7YE-01	11.12	886.00	1.00	days	TCGA-E1-A7YE	846.00	1.00	days	886.00	days	TCGA Lower Grade Glioma (LGG)	brain lower grade glioma	Brain	NO	NO	NO	NO	32.00	NO		NO	TCGA-E1-A7YE-F61220	TCGA-E1-A7YE				(11971.00)		886.00	0.00		846.00	81.00		NO	NO	NO				Seizures	0 - 30 Days	Additional New Tumor Event	Progressive Disease	NO		41865.00	FEMALE	NO	NO	Astrocytoma
173	TCGA-E1-A7YH-01	1.00	TCGA-E1-A7YH-01	9.59	2835.00	1.00	days	TCGA-E1-A7YH	1629.00	1.00	days	2835.00	days	TCGA Lower Grade Glioma (LGG)	brain lower grade glioma	Brain	NO	NO	NO	NO	47.00			NO	TCGA-E1-A7YH-F61239	TCGA-E1-A7YH	TCGA-E1-A7YH-01A			(17465.00)	3127.00	2835.00	0.00		1629.00	17.00		NO	YES	NO				Seizures	> 181 Days	Additional New Tumor Event	Progressive Disease			41865.00	FEMALE		NO	Astrocytoma
174	TCGA-E1-A7YI-01	1.00	TCGA-E1-A7YI-01	10.30	111.00	1.00	days	TCGA-E1-A7YI			days	111.00	days	TCGA Lower Grade Glioma (LGG)	brain lower grade glioma	Brain					33.00			NO		TCGA-E1-A7YI	TCGA-E1-A7YI-01A			(12229.00)	2022.00	111.00	0.00			11.00		NO	YES	NO				Motor/Movement Changes	31 - 90 Days					41771.00	FEMALE		NO	Astrocytoma
175	TCGA-E1-A7YJ-01	1.00	TCGA-E1-A7YJ-01	8.39	592.00	1.00	days	TCGA-E1-A7YJ	573.00	1.00	days	592.00	days	TCGA Lower Grade Glioma (LGG)	brain lower grade glioma	Brain	NO	NO	NO	NO	55.00			NO	TCGA-E1-A7YJ-F61259	TCGA-E1-A7YJ	TCGA-E1-A7YJ-01A			(20180.00)	1847.00	592.00	0.00		573.00	12.00		NO	NO	NO				Motor/Movement Changes	0 - 30 Days	Additional New Tumor Event	Progressive Disease			41866.00	MALE		YES	Astrocytoma
176	TCGA-E1-A7YK-01	1.00	TCGA-E1-A7YK-01	11.09	378.00	1.00	days	TCGA-E1-A7YK	378.00	0.00	days	378.00	days	TCGA Lower Grade Glioma (LGG)	brain lower grade glioma	Brain					52.00			NO	TCGA-E1-A7YK-F61287	TCGA-E1-A7YK				(19236.00)		378.00	0.00			6.00		NO	NO	NO				Seizures	0 - 30 Days					41866.00	MALE		NO	Astrocytoma
177	TCGA-E1-A7YL-01	1.00	TCGA-E1-A7YL-01	12.85	492.00	1.00	days	TCGA-E1-A7YL	329.00	1.00	days	492.00	days	TCGA Lower Grade Glioma (LGG)	brain lower grade glioma	Brain	NO	NO	NO	NO	46.00	NO		NO	TCGA-E1-A7YL-F61302	TCGA-E1-A7YL	TCGA-E1-A7YL-01A			(16982.00)	1372.00	492.00	0.00		329.00	14.00		NO	YES	YES				Headaches	31 - 90 Days	Additional New Tumor Event	Progressive Disease	NO		41866.00	MALE	NO	YES	Astrocytoma
178	TCGA-E1-A7YM-01	1.00	TCGA-E1-A7YM-01	9.10	648.00	1.00	days	TCGA-E1-A7YM	497.00	1.00	days	648.00	days	TCGA Lower Grade Glioma (LGG)	brain lower grade glioma	Brain	YES	NO	NO	NO	63.00	NO		NO	TCGA-E1-A7YM-F61425	TCGA-E1-A7YM	TCGA-E1-A7YM-01A			(23263.00)	1280.00	648.00	0.00		497.00	11.00		NO	YES	NO				Headaches	> 181 Days	Additional New Tumor Event	Progressive Disease	NO		41866.00	MALE	NO	YES	Astrocytoma

179	TCGA-E1-A7YN-01	1.00	TCGA-E1-A7YN-01	10.11	727.00	1.00	days	TCGA-E1-A7YN	485.00	1.00	days	727.00	days	TCGA Lower Grade Glioma (LGG)	brain lower grade glioma	Brain	YES	YES	NO	NO	63.00	NO		NO	TCGA-E1-A7YN-F61445	TCGA-E1-A7YN	TCGA-E1-A7YN-01A			(23180.00)	1148.00	727.00	0.00		485.00	20.00		NO	YES	NO				Visual Changes	0 - 30 Days	Additional New Tumor Event	Progressive Disease	NO		41866.00	FEMALE	NO	NO	Astrocytoma
180	TCGA-E1-A7YO-01	1.00	TCGA-E1-A7YO-01	8.96	2282.00	1.00	days	TCGA-E1-A7YO	2192.00	1.00	days	2282.00	days	TCGA Lower Grade Glioma (LGG)	brain lower grade glioma	Brain	NO	NO	NO	NO	45.00	NO		NO	TCGA-E1-A7YO-F61455	TCGA-E1-A7YO	TCGA-E1-A7YO-01A			(16770.00)	6582.00	2282.00	0.00		2192.00	1408.00		NO						Seizures	0 - 30 Days	Additional New Tumor Event	Progressive Disease	NO		41866.00	MALE	NO	NO	Oligodendroglioma
181	TCGA-E1-A7YQ-01	1.00	TCGA-E1-A7YQ-01	10.79	1578.00	1.00	days	TCGA-E1-A7YQ	1149.00	1.00	days	1578.00	days	TCGA Lower Grade Glioma (LGG)	brain lower grade glioma	Brain	NO	NO	NO	NO	58.00	NO		NO	TCGA-E1-A7YQ-F61543	TCGA-E1-A7YQ	TCGA-E1-A7YQ-01A			(21341.00)	4796.00	1578.00	0.00		1149.00	346.00		NO						Headaches	91 - 180 Days	Additional New Tumor Event	Progressive Disease	NO		41866.00	FEMALE	NO	YES	Oligodendroglioma
182	TCGA-E1-A7YS-01	1.00	TCGA-E1-A7YS-01	9.60	466.00	1.00	days	TCGA-E1-A7YS	466.00	0.00	days	466.00	days	TCGA Lower Grade Glioma (LGG)	brain lower grade glioma	Brain					71.00	NO		NO	TCGA-E1-A7YS-F61561	TCGA-E1-A7YS	TCGA-E1-A7YS-01A			(26159.00)	2123.00	466.00	0.00			16.00		NO	NO	NO				Mental Status Changes	0 - 30 Days		Stable Disease	NO		41866.00	MALE	NO	NO	Oligodendroglioma
183	TCGA-E1-A7YU-01	1.00	TCGA-E1-A7YU-01	9.39	23.00	1.00	days	TCGA-E1-A7YU			days	23.00	days	TCGA Lower Grade Glioma (LGG)	brain lower grade glioma	Brain					42.00			NO		TCGA-E1-A7YU				(15345.00)		23.00	0.00					NO	YES	YES				Headaches	31 - 90 Days					41862.00	MALE		YES	Oligoastrocytoma
184	TCGA-E1-A7YV-01	1.00	TCGA-E1-A7YV-01	9.03	987.00	1.00	days	TCGA-E1-A7YV	948.00	1.00	days	987.00	days	TCGA Lower Grade Glioma (LGG)	brain lower grade glioma	Brain	NO	NO	NO	NO	26.00	NO		NO	TCGA-E1-A7YV-F61594	TCGA-E1-A7YV	TCGA-E1-A7YV-01A			(9546.00)	1314.00	987.00	0.00		948.00	6.00		NO	NO	NO				Headaches	0 - 30 Days	Additional New Tumor Event	Progressive Disease	NO		41866.00	FEMALE	NO	YES	Oligoastrocytoma
185	TCGA-E1-A7YW-01	1.00	TCGA-E1-A7YW-01	8.49	1120.00	1.00	days	TCGA-E1-A7YW	613.00	1.00	days	1120.00	days	TCGA Lower Grade Glioma (LGG)	brain lower grade glioma	Brain	NO	YES	NO	NO	28.00	NO		NO	TCGA-E1-A7YW-F61613	TCGA-E1-A7YW	TCGA-E1-A7YW-01A			(10571.00)	4072.00	1120.00	0.00		613.00	13.00		NO	NO	NO				Seizures	91 - 180 Days	Additional New Tumor Event	Progressive Disease	NO		41866.00	MALE	NO	NO	Oligoastrocytoma
186	TCGA-E1-A7YY-01	1.00	TCGA-E1-A7YY-01	9.04	4445.00	1.00	days	TCGA-E1-A7YY	4266.00	1.00	days	4445.00	days	TCGA Lower Grade Glioma (LGG)	brain lower grade glioma	Brain	YES	NO	NO	NO	27.00	NO		YES	TCGA-E1-A7YY-F61637	TCGA-E1-A7YY	TCGA-E1-A7YY-01A			(9867.00)	5621.00	4445.00	0.00		4266.00	428.00		NO						Seizures	0 - 30 Days	Additional New Tumor Event	Progressive Disease	NO		41866.00	FEMALE	NO	NO	Oligodendroglioma
187	TCGA-E1-A7Z2-01	1.00	TCGA-E1-A7Z2-01	9.94	398.00	1.00	days	TCGA-E1-A7Z2	269.00	1.00	days	398.00	days	TCGA Lower Grade Glioma (LGG)	brain lower grade glioma	Brain	YES	YES	NO	NO	58.00	NO		NO	TCGA-E1-A7Z2-F61680	TCGA-E1-A7Z2	TCGA-E1-A7Z2-01A			(21341.00)	614.00	398.00	0.00		269.00	39.00		NO	YES	NO				Seizures	31 - 90 Days	Additional New Tumor Event	Progressive Disease	YES	clams	41866.00	FEMALE	NO	NO	Oligodendroglioma
188	TCGA-E1-A7Z3-01	1.00	TCGA-E1-A7Z3-01	9.56	2235.00	1.00	days	TCGA-E1-A7Z3	2072.00	1.00	days	2235.00	days	TCGA Lower Grade Glioma (LGG)	brain lower grade glioma	Brain		NO	NO	NO	31.00			NO	TCGA-E1-A7Z3-F61703	TCGA-E1-A7Z3	TCGA-E1-A7Z3-01A			(11330.00)	7912.00	2235.00	0.00		2072.00			NO						Seizures	0 - 30 Days	Additional New Tumor Event	Progressive Disease			41869.00	FEMALE		NO	Astrocytoma
189	TCGA-E1-A7Z4-01	1.00	TCGA-E1-A7Z4-01	8.72	4412.00	1.00	days	TCGA-E1-A7Z4	4339.00	1.00	days	4412.00	days	TCGA Lower Grade Glioma (LGG)	brain lower grade glioma	Brain	NO	NO	NO	NO	35.00	NO		NO	TCGA-E1-A7Z4-F61765	TCGA-E1-A7Z4				(12849.00)		4412.00	0.00		4339.00	974.00		NO	YES	NO				Headaches	31 - 90 Days	Additional New Tumor Event	Progressive Disease	NO		41869.00	MALE	NO	YES	Astrocytoma
190	TCGA-E1-A7Z6-01	1.00	TCGA-E1-A7Z6-01	9.62	984.00	1.00	days	TCGA-E1-A7Z6	971.00	1.00	days	984.00	days	TCGA Lower Grade Glioma (LGG)	brain lower grade glioma	Brain	NO	NO	NO	NO	41.00	NO		NO	TCGA-E1-A7Z6-F61776	TCGA-E1-A7Z6	TCGA-E1-A7Z6-01A			(14985.00)	1588.00	984.00	0.00		971.00	19.00		NO	YES	NO				Headaches	> 181 Days	Additional New Tumor Event	Progressive Disease	NO		41870.00	FEMALE	NO	YES	Astrocytoma
191	TCGA-EZ-7264-01	0.00	TCGA-EZ-7264-01	8.53	1201.00	0.00	days	TCGA-EZ-7264	1201.00	0.00	days	1201.00	days	TCGA Lower Grade Glioma (LGG)	brain lower grade glioma	Brain					47.00	NO		NO	TCGA-EZ-7264-F66594	TCGA-EZ-7264	TCGA-EZ-7264-01A			(17432.00)			0.00	1201.00				NO	NO	NO				Mental Status Changes	0 - 30 Days	Scheduled Follow-up Submission	Stable Disease	NO		41933.00	FEMALE	NO	NO	Oligodendroglioma
192	TCGA-F6-A8O3-01	0.00	TCGA-F6-A8O3-01	9.53	7.00	0.00	days	TCGA-F6-A8O3	7.00	0.00	days	7.00	days	TCGA Lower Grade Glioma (LGG)	brain lower grade glioma	Brain					34.00	NO		NO	TCGA-F6-A8O3-F67155	TCGA-F6-A8O3	TCGA-F6-A8O3-01A			(12578.00)	105.00		0.00	7.00				NO	NO	NO				Headaches		Scheduled Follow-up Submission		NO		41967.00	MALE	NO	YES	Oligodendroglioma
193	TCGA-F6-A8O4-01	0.00	TCGA-F6-A8O4-01	9.76	6.00	0.00	days	TCGA-F6-A8O4	6.00	0.00	days	6.00	days	TCGA Lower Grade Glioma (LGG)	brain lower grade glioma	Brain					44.00	NO		NO	TCGA-F6-A8O4-F67156	TCGA-F6-A8O4	TCGA-F6-A8O4-01A			(16202.00)	100.00		0.00	6.00				NO	NO	NO				Headaches		Scheduled Follow-up Submission		NO		41967.00	MALE	NO	YES	Astrocytoma
194	TCGA-FG-5962-01	0.00	TCGA-FG-5962-01	9.79	1453.00	0.00	days	TCGA-FG-5962	1453.00	0.00	days	1453.00	days	TCGA Lower Grade Glioma (LGG)	brain lower grade glioma	Brain					54.00	NO		NO	TCGA-FG-5962-F43040	TCGA-FG-5962	TCGA-FG-5962-01B			(19818.00)			0.00	1453.00				NO	YES	NO	> 20 Years			Visual Changes	> 181 Days	Scheduled Follow-up Submission	Stable Disease	NO		41403.00	MALE	YES	NO	Oligodendroglioma
195	TCGA-FG-5963-01	1.00	TCGA-FG-5963-01	10.64	775.00	1.00	days	TCGA-FG-5963	497.00	1.00	days	775.00	days	TCGA Lower Grade Glioma (LGG)	brain lower grade glioma	Brain	YES	NO	NO		23.00	YES		NO	TCGA-FG-5963-F42259	TCGA-FG-5963	TCGA-FG-5963-01A			(8571.00)		775.00	0.00	547.00	497.00	0.00		NO	YES	NO	12 - 20 Years	12 - 20 Years		Seizures	0 - 30 Days	Scheduled Follow-up Submission	Progressive Disease	NO		41401.00	MALE	NO	NO	Astrocytoma
196	TCGA-FG-5963-02	1.00	TCGA-FG-5963-02	9.43	775.00	1.00	days	TCGA-FG-5963	497.00	1.00	days	775.00	days	TCGA Lower Grade Glioma (LGG)	brain lower grade glioma	Brain	YES	NO	NO		23.00	YES		NO	TCGA-FG-5963-F42259	TCGA-FG-5963	TCGA-FG-5963-02A			(8571.00)	1095.00	775.00	0.00	547.00	497.00	0.00		NO	YES	NO	12 - 20 Years	12 - 20 Years		Seizures	0 - 30 Days	Scheduled Follow-up Submission	Progressive Disease	NO		41401.00	MALE	NO	NO	Astrocytoma
197	TCGA-FG-5964-01	0.00	TCGA-FG-5964-01	9.70	1588.00	0.00	days	TCGA-FG-5964	1588.00	0.00	days	1588.00	days	TCGA Lower Grade Glioma (LGG)	brain lower grade glioma	Brain					62.00	NO		NO	TCGA-FG-5964-F63736	TCGA-FG-5964	TCGA-FG-5964-01A			(22735.00)			0.00	1588.00				NO	YES	NO	> 20 Years		> 20 Years	Seizures	0 - 30 Days	Scheduled Follow-up Submission	Stable Disease	YES	Processed food	41866.00	MALE	NO	NO	Oligodendroglioma
198	TCGA-FG-5965-01	1.00	TCGA-FG-5965-01	8.84	1120.00	1.00	days	TCGA-FG-5965	1035.00	1.00	days	1120.00	days	TCGA Lower Grade Glioma (LGG)	brain lower grade glioma	Brain	YES	NO	YES	NO	39.00	NO		NO	TCGA-FG-5965-F32837	TCGA-FG-5965	TCGA-FG-5965-01B	1069.00		(14249.00)		1120.00	0.00	979.00	1035.00	259.00		NO	YES	NO				Seizures	0 - 30 Days	Additional New Tumor Event	Progressive Disease	NO		41073.00	FEMALE	NO	YES	Oligoastrocytoma
199	TCGA-FG-5965-02	1.00	TCGA-FG-5965-02	10.36	1120.00	1.00	days	TCGA-FG-5965	1035.00	1.00	days	1120.00	days	TCGA Lower Grade Glioma (LGG)	brain lower grade glioma	Brain	YES	NO	YES	NO	39.00	NO		NO	TCGA-FG-5965-F32837	TCGA-FG-5965	TCGA-FG-5965-02B	1069.00		(14249.00)	1437.00	1120.00	0.00	979.00	1035.00	259.00		NO	YES	NO				Seizures	0 - 30 Days	Additional New Tumor Event	Progressive Disease	NO		41073.00	FEMALE	NO	YES	Oligoastrocytoma
200	TCGA-FG-6688-01	0.00	TCGA-FG-6688-01	12.07	571.00	0.00	days	TCGA-FG-6688	405.00	1.00	days	571.00	days	TCGA Lower Grade Glioma (LGG)	brain lower grade glioma	Brain	YES	NO	NO	NO	59.00	NO		NO	TCGA-FG-6688-F32791	TCGA-FG-6688	TCGA-FG-6688-01A			(21745.00)			0.00	571.00	405.00	405.00		NO	YES	NO				Seizures	0 - 30 Days	Additional New Tumor Event	Stable Disease	NO		41072.00	FEMALE	NO	NO	Astrocytoma
201	TCGA-FG-6689-01	0.00	TCGA-FG-6689-01	9.06	190.00	0.00	days	TCGA-FG-6689	218.00	1.00	days	190.00	days	TCGA Lower Grade Glioma (LGG)	brain lower grade glioma	Brain			NO		30.00	YES	cats	NO	TCGA-FG-6689-F43039	TCGA-FG-6689	TCGA-FG-6689-01A			(11312.00)			0.00	190.00	218.00	(10.00)		NO	YES	NO	< 12 Years	> 20 Years		Headaches	31 - 90 Days	Scheduled Follow-up Submission		NO		41414.00	MALE	YES	YES	Astrocytoma
202	TCGA-FG-6690-01	0.00	TCGA-FG-6690-01	8.72	1294.00	0.00	days	TCGA-FG-6690	1294.00	0.00	days	1294.00	days	TCGA Lower Grade Glioma (LGG)	brain lower grade glioma	Brain					70.00	NO		NO	TCGA-FG-6690-F63742	TCGA-FG-6690	TCGA-FG-6690-01A			(25667.00)			0.00	1294.00		(23.00)		NO	NO	NO				Seizures	0 - 30 Days	Scheduled Follow-up Submission	Stable Disease	NO		41866.00	MALE	NO	NO	Oligodendroglioma
203	TCGA-FG-6691-01	0.00	TCGA-FG-6691-01	9.60	1257.00	0.00	days	TCGA-FG-6691	913.00	1.00	days	1257.00	days	TCGA Lower Grade Glioma (LGG)	brain lower grade glioma	Brain	YES	YES	YES		23.00	NO		NO	TCGA-FG-6691-F63744	TCGA-FG-6691	TCGA-FG-6691-01A	984.00		(8403.00)			0.00	1257.00	913.00	(9.00)		NO	NO	NO				Headaches	0 - 30 Days	Scheduled Follow-up Submission	Stable Disease	NO		41866.00	FEMALE	NO	YES	Astrocytoma
204	TCGA-FG-6692-01	1.00	TCGA-FG-6692-01	11.35	561.00	1.00	days	TCGA-FG-6692			days	561.00	days	TCGA Lower Grade Glioma (LGG)	brain lower grade glioma	Brain					63.00				TCGA-FG-6692-F41569	TCGA-FG-6692				(23374.00)		561.00	0.00	126.00		42.00	1.00		YES	NO				Seizures	0 - 30 Days	Scheduled Follow-up Submission				41354.00	MALE		NO	Oligodendroglioma
205	TCGA-FG-7634-01	0.00	TCGA-FG-7634-01	12.00	467.00	0.00	days	TCGA-FG-7634			days	467.00	days	TCGA Lower Grade Glioma (LGG)	brain lower grade glioma	Brain					28.00	NO		NO	TCGA-FG-7634-F41580	TCGA-FG-7634	TCGA-FG-7634-01A			(10476.00)			0.00	467.00				NO	YES	NO				Seizures	0 - 30 Days	Scheduled Follow-up Submission		NO		41354.00	MALE	NO	NO	Oligodendroglioma
206	TCGA-FG-7636-01	0.00	TCGA-FG-7636-01	8.73	544.00	0.00	days	TCGA-FG-7636	544.00	0.00	days	544.00	days	TCGA Lower Grade Glioma (LGG)	brain lower grade glioma	Brain					48.00	NO		NO	TCGA-FG-7636-F43425	TCGA-FG-7636	TCGA-FG-7636-01A			(17843.00)			0.00	544.00		(7.00)	0.00	NO	NO	NO				Headaches	> 181 Days	Scheduled Follow-up Submission	Complete Remission/Response	NO		41415.00	MALE	NO	YES	Astrocytoma
207	TCGA-FG-7637-01	0.00	TCGA-FG-7637-01	8.76	1219.00	0.00	days	TCGA-FG-7637	1219.00	0.00	days	1219.00	days	TCGA Lower Grade Glioma (LGG)	brain lower grade glioma	Brain					49.00	NO		NO	TCGA-FG-7637-F41579	TCGA-FG-7637	TCGA-FG-7637-01A			(18084.00)			0.00	1219.00				NO	YES	NO	> 20 Years			Seizures	> 181 Days	Scheduled Follow-up Submission	Stable Disease	NO		41354.00	MALE	YES	NO	Oligoastrocytoma
208	TCGA-FG-7638-01	0.00	TCGA-FG-7638-01	10.77	686.00	0.00	days	TCGA-FG-7638	407.00	1.00	days	686.00	days	TCGA Lower Grade Glioma (LGG)	brain lower grade glioma	Brain	YES	NO	NO		31.00	NO		NO	TCGA-FG-7638-F43363	TCGA-FG-7638	TCGA-FG-7638-01B			(11528.00)			0.00	686.00	407.00	(13.00)	0.00	NO	NO	NO				Headaches	0 - 30 Days	Scheduled Follow-up Submission	Stable Disease	NO		41414.00	FEMALE	NO	YES	Oligodendroglioma
209	TCGA-FG-7641-01	0.00	TCGA-FG-7641-01	8.86	627.00	0.00	days	TCGA-FG-7641	627.00	0.00	days	627.00	days	TCGA Lower Grade Glioma (LGG)	brain lower grade glioma	Brain					31.00	NO		NO	TCGA-FG-7641-F43341	TCGA-FG-7641	TCGA-FG-7641-01B			(11357.00)			0.00	627.00				NO	NO	NO				Headaches	> 181 Days	Scheduled Follow-up Submission	Stable Disease	NO		41411.00	MALE	NO	YES	Oligodendroglioma
210	TCGA-FG-7643-01	0.00	TCGA-FG-7643-01	10.32	611.00	0.00	days	TCGA-FG-7643	254.00	1.00	days	611.00	days	TCGA Lower Grade Glioma (LGG)	brain lower grade glioma	Brain	NO	NO	YES		49.00	YES	Cats	NO	TCGA-FG-7643-F45156	TCGA-FG-7643	TCGA-FG-7643-01A	286.00		(18206.00)			0.00	611.00	254.00			NO	YES	NO	> 20 Years	> 20 Years		Seizures	91 - 180 Days	Scheduled Follow-up Submission	Stable Disease	NO		41465.00	FEMALE	YES	NO	Oligoastrocytoma
211	TCGA-FG-8181-01	0.00	TCGA-FG-8181-01	9.44	862.00	0.00	days	TCGA-FG-8181	862.00	0.00	days	862.00	days	TCGA Lower Grade Glioma (LGG)	brain lower grade glioma	Brain					23.00				TCGA-FG-8181-F64421	TCGA-FG-8181	TCGA-FG-8181-01A			(8642.00)			0.00	862.00		(13.00)										Scheduled Follow-up Submission	Stable Disease			41884.00	MALE			Oligoastrocytoma
212	TCGA-FG-8182-01	0.00	TCGA-FG-8182-01	8.87	416.00	0.00	days	TCGA-FG-8182	416.00	0.00	days	416.00	days	TCGA Lower Grade Glioma (LGG)	brain lower grade glioma	Brain					35.00	NO		NO	TCGA-FG-8182-F43399	TCGA-FG-8182	TCGA-FG-8182-01A			(12972.00)			0.00	416.00				NO	NO	NO				Seizures	0 - 30 Days	Scheduled Follow-up Submission	Stable Disease	NO		41414.00	MALE	NO	NO	Oligodendroglioma
213	TCGA-FG-8185-01	0.00	TCGA-FG-8185-01	10.21	433.00	0.00	days	TCGA-FG-8185	433.00	0.00	days	433.00	days	TCGA Lower Grade Glioma (LGG)	brain lower grade glioma	Brain					37.00	NO		NO	TCGA-FG-8185-F43366	TCGA-FG-8185	TCGA-FG-8185-01A			(13621.00)			0.00	433.00		21.00		NO	NO	NO			> 20 Years	Headaches	91 - 180 Days	Scheduled Follow-up Submission	Complete Remission/Response	YES		41414.00	MALE	NO	YES	Astrocytoma
214	TCGA-FG-8186-01	0.00	TCGA-FG-8186-01	10.43	487.00	0.00	days	TCGA-FG-8186	487.00	0.00	days	487.00	days	TCGA Lower Grade Glioma (LGG)	brain lower grade glioma	Brain					42.00	NO		NO	TCGA-FG-8186-F42521	TCGA-FG-8186	TCGA-FG-8186-01A			(15431.00)			0.00	487.00		13.00		NO	YES	NO				Seizures	0 - 30 Days	Scheduled Follow-up Submission	Stable Disease	NO		41387.00	FEMALE	NO	YES	Oligoastrocytoma

215	TCGA-FG-8187-01	0.00	TCGA-FG-8187-01	10.21	611.00	0.00	days	TCGA-FG-8187	611.00	0.00	days	611.00	days	TCGA Lower Grade Glioma (LGG)	brain lower grade glioma	Brain					31.00				TCGA-FG-8187-F43456	TCGA-FG-8187	TCGA-FG-8187-01A			(11684.00)			0.00	611.00		0.00										Scheduled Follow-up Submission	Stable Disease			41416.00	MALE			Oligoastrocytoma
216	TCGA-FG-8188-01	0.00	TCGA-FG-8188-01	10.21	455.00	0.00	days	TCGA-FG-8188	384.00	1.00	days	455.00	days	TCGA Lower Grade Glioma (LGG)	brain lower grade glioma	Brain	NO	YES	NO		41.00	NO		NO	TCGA-FG-8188-F43454	TCGA-FG-8188	TCGA-FG-8188-01A			(15051.00)			0.00	455.00	384.00	(15.00)		NO	NO	NO				Mental Status Changes	0 - 30 Days	Scheduled Follow-up Submission		NO		41416.00	MALE	NO	NO	Oligoastrocytoma
217	TCGA-FG-8189-01	0.00	TCGA-FG-8189-01	8.85	685.00	0.00	days	TCGA-FG-8189	685.00	0.00	days	685.00	days	TCGA Lower Grade Glioma (LGG)	brain lower grade glioma	Brain					33.00	NO		YES	TCGA-FG-8189-F58503	TCGA-FG-8189	TCGA-FG-8189-01B			(12384.00)	203.00		0.00	685.00		83.00		YES	NO	NO	> 20 Years			Seizures	> 181 Days	Scheduled Follow-up Submission	Stable Disease	NO		41740.00	FEMALE	YES	NO	Oligodendroglioma
218	TCGA-FG-8191-01	0.00	TCGA-FG-8191-01	10.28	992.00	0.00	days	TCGA-FG-8191	992.00	0.00	days	992.00	days	TCGA Lower Grade Glioma (LGG)	brain lower grade glioma	Brain					30.00	NO		NO	TCGA-FG-8191-F66698	TCGA-FG-8191	TCGA-FG-8191-01A			(11252.00)			0.00	992.00				NO	NO	NO				Sensory Changes	> 181 Days	Scheduled Follow-up Submission	Stable Disease	NO		41934.00	MALE	NO	NO	Oligodendroglioma
219	TCGA-FG-A4MT-01	0.00	TCGA-FG-A4MT-01	9.95	1164.00	0.00	days	TCGA-FG-A4MT	499.00	1.00	days	1164.00	days	TCGA Lower Grade Glioma (LGG)	brain lower grade glioma	Brain	NO	YES	YES		27.00	NO		NO	TCGA-FG-A4MT-F56666	TCGA-FG-A4MT	TCGA-FG-A4MT-01A	647.00		(10082.00)	661.00		0.00	1164.00	499.00	499.00		NO	NO	NO				Seizures	0 - 30 Days	Scheduled Follow-up Submission	Complete Remission/Response	NO		41687.00	FEMALE	NO	YES	Oligodendroglioma
220	TCGA-FG-A4MT-02	0.00	TCGA-FG-A4MT-02	9.60	1164.00	0.00	days	TCGA-FG-A4MT	499.00	1.00	days	1164.00	days	TCGA Lower Grade Glioma (LGG)	brain lower grade glioma	Brain	NO	YES	YES		27.00	NO		NO	TCGA-FG-A4MT-F56666	TCGA-FG-A4MT	TCGA-FG-A4MT-02A	647.00		(10082.00)	827.00		0.00	1164.00	499.00	499.00		NO	NO	NO				Seizures	0 - 30 Days	Scheduled Follow-up Submission	Complete Remission/Response	NO		41687.00	FEMALE	NO	YES	Oligodendroglioma
221	TCGA-FG-A4MU-01	0.00	TCGA-FG-A4MU-01	12.23	326.00	0.00	days	TCGA-FG-A4MU			days	326.00	days	TCGA Lower Grade Glioma (LGG)	brain lower grade glioma	Brain					58.00	NO		NO	TCGA-FG-A4MU-F63739	TCGA-FG-A4MU	TCGA-FG-A4MU-01B			(21450.00)	258.00		0.00	326.00				NO	NO	NO				Headaches	0 - 30 Days	Scheduled Follow-up Submission		NO		41866.00	MALE	NO	YES	Oligoastrocytoma
222	TCGA-FG-A4MW-01	1.00	TCGA-FG-A4MW-01	11.43	559.00	1.00	days	TCGA-FG-A4MW	226.00	1.00	days	559.00	days	TCGA Lower Grade Glioma (LGG)	brain lower grade glioma	Brain	YES	NO	NO		63.00	NO		YES	TCGA-FG-A4MW-F61644	TCGA-FG-A4MW	TCGA-FG-A4MW-01A			(23077.00)	64.00	559.00	0.00	160.00	226.00	141.00	1.00	NO	YES	NO				Seizures	0 - 30 Days	Scheduled Follow-up Submission	Progressive Disease	NO		41822.00	MALE	NO	NO	Oligoastrocytoma
223	TCGA-FG-A4MX-01	0.00	TCGA-FG-A4MX-01	8.99	569.00	0.00	days	TCGA-FG-A4MX	569.00	0.00	days	569.00	days	TCGA Lower Grade Glioma (LGG)	brain lower grade glioma	Brain					47.00	NO		NO	TCGA-FG-A4MX-F57707	TCGA-FG-A4MX	TCGA-FG-A4MX-01A			(17381.00)	115.00		0.00	569.00		(23.00)	0.00	NO	NO	NO				Motor/Movement Changes	0 - 30 Days	Scheduled Follow-up Submission	Complete Remission/Response	NO		41719.00	MALE	NO	NO	Astrocytoma
224	TCGA-FG-A4MY-01	0.00	TCGA-FG-A4MY-01	10.59	721.00	0.00	days	TCGA-FG-A4MY	721.00	0.00	days	721.00	days	TCGA Lower Grade Glioma (LGG)	brain lower grade glioma	Brain					44.00	NO		NO	TCGA-FG-A4MY-F58505	TCGA-FG-A4MY	TCGA-FG-A4MY-01A			(16310.00)	212.00		0.00	721.00				NO	NO	NO				Seizures	31 - 90 Days	Scheduled Follow-up Submission	Stable Disease	NO		41740.00	FEMALE	NO	YES	Oligoastrocytoma
225	TCGA-FG-A60J-01	0.00	TCGA-FG-A60J-01	10.34	449.00	0.00	days	TCGA-FG-A60J	449.00	0.00	days	449.00	days	TCGA Lower Grade Glioma (LGG)	brain lower grade glioma	Brain					47.00	NO		NO	TCGA-FG-A60J-F65604	TCGA-FG-A60J	TCGA-FG-A60J-01A			(17339.00)	240.00		0.00	449.00		(18.00)	1.00	NO	NO	NO				Seizures	31 - 90 Days	Scheduled Follow-up Submission		NO		41907.00	FEMALE	NO	NO	Oligoastrocytoma
226	TCGA-FG-A60K-01	0.00	TCGA-FG-A60K-01	8.79	542.00	0.00	days	TCGA-FG-A60K	542.00	0.00	days	542.00	days	TCGA Lower Grade Glioma (LGG)	brain lower grade glioma	Brain					34.00	YES	Dogs	NO	TCGA-FG-A60K-F61189	TCGA-FG-A60K				(12445.00)			0.00	542.00				NO	YES	NO	> 20 Years	> 20 Years		Mental Status Changes	0 - 30 Days	Scheduled Follow-up Submission	Stable Disease	NO		41815.00	FEMALE	YES	NO	Oligoastrocytoma
227	TCGA-FG-A60L-01	0.00	TCGA-FG-A60L-01	10.41	655.00	0.00	days	TCGA-FG-A60L	655.00	0.00	days	655.00	days	TCGA Lower Grade Glioma (LGG)	brain lower grade glioma	Brain					34.00			NO	TCGA-FG-A60L-F65511	TCGA-FG-A60L	TCGA-FG-A60L-01A			(12580.00)	121.00		0.00	655.00		8.00	0.00	NO		NO				Seizures	0 - 30 Days	Scheduled Follow-up Submission	Complete Remission/Response			41906.00	FEMALE		NO	Astrocytoma
228	TCGA-FG-A6IZ-01	0.00	TCGA-FG-A6IZ-01	9.07	457.00	0.00	days	TCGA-FG-A6IZ	457.00	0.00	days	457.00	days	TCGA Lower Grade Glioma (LGG)	brain lower grade glioma	Brain					60.00	NO		NO	TCGA-FG-A6IZ-F63721	TCGA-FG-A6IZ	TCGA-FG-A6IZ-01A			(22275.00)	157.00		0.00	457.00				NO	NO	NO	> 20 Years			Seizures	0 - 30 Days	Scheduled Follow-up Submission	Complete Remission/Response	NO		41866.00	MALE	YES	NO	Oligodendroglioma
229	TCGA-FG-A6J1-01	0.00	TCGA-FG-A6J1-01	9.00	500.00	0.00	days	TCGA-FG-A6J1	500.00	0.00	days	500.00	days	TCGA Lower Grade Glioma (LGG)	brain lower grade glioma	Brain					44.00	NO		NO	TCGA-FG-A6J1-F63724	TCGA-FG-A6J1	TCGA-FG-A6J1-01A			(16106.00)	91.00		0.00	500.00		27.00		NO	YES	NO				Seizures	0 - 30 Days	Scheduled Follow-up Submission	Stable Disease	NO		41866.00	FEMALE	NO	NO	Oligodendroglioma
230	TCGA-FG-A6J3-01	0.00	TCGA-FG-A6J3-01	12.42	677.00	0.00	days	TCGA-FG-A6J3	677.00	0.00	days	677.00	days	TCGA Lower Grade Glioma (LGG)	brain lower grade glioma	Brain					52.00	NO			TCGA-FG-A6J3-F64032	TCGA-FG-A6J3	TCGA-FG-A6J3-01A			(19138.00)	267.00		0.00	677.00		106.00	1.00	NO						Sensory Changes	0 - 30 Days	Scheduled Follow-up Submission	Stable Disease	NO		41876.00	FEMALE	NO	NO	Astrocytoma
231	TCGA-FG-A70Y-01	0.00	TCGA-FG-A70Y-01	10.75	868.00	0.00	days	TCGA-FG-A70Y			days	868.00	days	TCGA Lower Grade Glioma (LGG)	brain lower grade glioma	Brain					20.00			NO	TCGA-FG-A70Y-F65608	TCGA-FG-A70Y	TCGA-FG-A70Y-01A			(7421.00)	804.00		0.00	868.00				NO	NO	NO				Headaches	0 - 30 Days	Scheduled Follow-up Submission				41907.00	FEMALE		YES	Oligodendroglioma
232	TCGA-FG-A70Z-01	0.00	TCGA-FG-A70Z-01	11.99	328.00	0.00	days	TCGA-FG-A70Z			days	328.00	days	TCGA Lower Grade Glioma (LGG)	brain lower grade glioma	Brain					53.00			NO	TCGA-FG-A70Z-F64424	TCGA-FG-A70Z	TCGA-FG-A70Z-01A			(19717.00)	720.00		0.00	328.00				NO	YES					Headaches	31 - 90 Days	Scheduled Follow-up Submission	Stable Disease			41884.00	FEMALE		YES	Oligoastrocytoma
233	TCGA-FG-A710-01	0.00	TCGA-FG-A710-01	8.75	1115.00	0.00	days	TCGA-FG-A710	1115.00	0.00	days	1115.00	days	TCGA Lower Grade Glioma (LGG)	brain lower grade glioma	Brain					50.00				TCGA-FG-A710-F64423	TCGA-FG-A710	TCGA-FG-A710-01A			(18415.00)	823.00		0.00	1115.00					NO	NO				Headaches	91 - 180 Days	Scheduled Follow-up Submission	Stable Disease			41884.00	FEMALE		YES	Oligodendroglioma
234	TCGA-FG-A711-01	1.00	TCGA-FG-A711-01	10.62	1481.00	1.00	days	TCGA-FG-A711	1277.00	1.00	days	1481.00	days	TCGA Lower Grade Glioma (LGG)	brain lower grade glioma	Brain	YES	NO	NO		33.00	NO		NO	TCGA-FG-A711-F65436	TCGA-FG-A711	TCGA-FG-A711-01A			(12248.00)	1265.00	1481.00	0.00	1329.00	1277.00	871.00		YES	NO	NO	< 12 Years			Seizures	0 - 30 Days	Scheduled Follow-up Submission	Progressive Disease	NO		41905.00	FEMALE	NO	NO	Oligodendroglioma
235	TCGA-FG-A713-01	0.00	TCGA-FG-A713-01	10.04	622.00	0.00	days	TCGA-FG-A713	622.00	0.00	days	622.00	days	TCGA Lower Grade Glioma (LGG)	brain lower grade glioma	Brain					74.00	NO		NO	TCGA-FG-A713-F70642	TCGA-FG-A713	TCGA-FG-A713-01A			(27232.00)	82.00		0.00	622.00		13.00		YES	NO	NO	> 20 Years		> 20 Years	Headaches	> 181 Days	Scheduled Follow-up Submission	Complete Remission/Response	YES	Melon	42062.00	FEMALE	YES	YES	Oligoastrocytoma
236	TCGA-FG-A87N-01	0.00	TCGA-FG-A87N-01	7.90	567.00	0.00	days	TCGA-FG-A87N	567.00	0.00	days	567.00	days	TCGA Lower Grade Glioma (LGG)	brain lower grade glioma	Brain					37.00			NO	TCGA-FG-A87N-F70255	TCGA-FG-A87N	TCGA-FG-A87N-01A			(13606.00)	113.00		0.00	567.00				NO	NO	NO				Seizures	0 - 30 Days	Scheduled Follow-up Submission	Stable Disease	NO		42037.00	FEMALE	NO	YES	Astrocytoma
237	TCGA-FG-A87Q-01	0.00	TCGA-FG-A87Q-01	10.17	174.00	0.00	days	TCGA-FG-A87Q	174.00	0.00	days	174.00	days	TCGA Lower Grade Glioma (LGG)	brain lower grade glioma	Brain					61.00			NO	TCGA-FG-A87Q-F72685	TCGA-FG-A87Q	TCGA-FG-A87Q-01A			(22565.00)	90.00		0.00	174.00		174.00		NO						Seizures	0 - 30 Days	Scheduled Follow-up Submission		YES	Dairy/lactose	42216.00	FEMALE	NO	NO	Astrocytoma
238	TCGA-FN-7833-01	0.00	TCGA-FN-7833-01	9.87	837.00	0.00	days	TCGA-FN-7833	837.00	0.00	days	837.00	days	TCGA Lower Grade Glioma (LGG)	brain lower grade glioma	Brain					25.00				TCGA-FN-7833-F66000	TCGA-FN-7833	TCGA-FN-7833-01A			(9228.00)			0.00	837.00										Mental Status Changes		Scheduled Follow-up Submission	Complete Remission/Response			41913.00	MALE		NO	Oligoastrocytoma
239	TCGA-HT-7467-01	0.00	TCGA-HT-7467-01	10.13	3.00	0.00	days	TCGA-HT-7467			days	3.00	days	TCGA Lower Grade Glioma (LGG)	brain lower grade glioma	Brain					54.00	NO		NO	TCGA-HT-7467-F28641	TCGA-HT-7467	TCGA-HT-7467-01A			(19944.00)			0.00	3.00				NO	YES	NO				Sensory Changes	0 - 30 Days	Scheduled Follow-up Submission	Complete Remission/Response	NO		40966.00	MALE	NO	NO	Oligodendroglioma
240	TCGA-HT-7468-01	0.00	TCGA-HT-7468-01	9.28	203.00	0.00	days	TCGA-HT-7468	203.00	0.00	days	203.00	days	TCGA Lower Grade Glioma (LGG)	brain lower grade glioma	Brain					30.00	NO		NO	TCGA-HT-7468-F21233	TCGA-HT-7468	TCGA-HT-7468-01A			(11209.00)			0.00	203.00		203.00		NO	NO	NO				Seizures	> 181 Days	Scheduled Follow-up Submission	Stable Disease	YES	shellfish	40946.00	MALE	NO	YES	Oligodendroglioma
241	TCGA-HT-7469-01	1.00	TCGA-HT-7469-01	9.37	351.00	1.00	days	TCGA-HT-7469	92.00	1.00	days	351.00	days	TCGA Lower Grade Glioma (LGG)	brain lower grade glioma	Brain	YES	NO	YES	NO	30.00	NO		NO	TCGA-HT-7469-F28780	TCGA-HT-7469	TCGA-HT-7469-01A	92.00		(11097.00)		351.00	0.00	238.00	92.00			NO	YES	NO				Motor/Movement Changes	0 - 30 Days	Additional New Tumor Event	Progressive Disease	NO		41005.00	MALE	NO	YES	Oligodendroglioma
242	TCGA-HT-7470-01	1.00	TCGA-HT-7470-01	9.77	1220.00	1.00	days	TCGA-HT-7470	1220.00	0.00	days	1220.00	days	TCGA Lower Grade Glioma (LGG)	brain lower grade glioma	Brain					37.00	NO		NO	TCGA-HT-7470-F66413	TCGA-HT-7470	TCGA-HT-7470-01A			(13611.00)		1220.00	0.00	268.00		133.00	0.00	NO	NO	NO				Headaches	> 181 Days	Scheduled Follow-up Submission	Progressive Disease	NO		41926.00	MALE	NO	YES	Oligodendroglioma
243	TCGA-HT-7471-01	0.00	TCGA-HT-7471-01	10.02	4.00	0.00	days	TCGA-HT-7471			days	4.00	days	TCGA Lower Grade Glioma (LGG)	brain lower grade glioma	Brain					37.00	NO		NO	TCGA-HT-7471-F50543	TCGA-HT-7471	TCGA-HT-7471-01A			(13564.00)			0.00	4.00				NO						Motor/Movement Changes	0 - 30 Days	Scheduled Follow-up Submission		NO		41571.00	FEMALE	NO	NO	Oligodendroglioma
244	TCGA-HT-7472-01	0.00	TCGA-HT-7472-01	11.25	1.00	0.00	days	TCGA-HT-7472	1.00	0.00	days	1.00	days	TCGA Lower Grade Glioma (LGG)	brain lower grade glioma	Brain					38.00	NO		NO	TCGA-HT-7472-F28774	TCGA-HT-7472	TCGA-HT-7472-01A			(13968.00)			0.00	1.00				NO	NO	NO				Seizures	91 - 180 Days	Scheduled Follow-up Submission	Stable Disease	NO		40969.00	MALE	NO	NO	Oligodendroglioma
245	TCGA-HT-7473-01	0.00	TCGA-HT-7473-01	10.97	503.00	0.00	days	TCGA-HT-7473	503.00	0.00	days	503.00	days	TCGA Lower Grade Glioma (LGG)	brain lower grade glioma	Brain					28.00	NO		NO	TCGA-HT-7473-F29569	TCGA-HT-7473	TCGA-HT-7473-01A			(10306.00)			0.00	503.00				NO	NO	NO				Headaches	0 - 30 Days	Scheduled Follow-up Submission		NO		40987.00	MALE	NO	YES	Oligoastrocytoma
246	TCGA-HT-7474-01	0.00	TCGA-HT-7474-01	9.94	114.00	0.00	days	TCGA-HT-7474	114.00	0.00	days	114.00	days	TCGA Lower Grade Glioma (LGG)	brain lower grade glioma	Brain					52.00	NO		NO	TCGA-HT-7474-F28779	TCGA-HT-7474	TCGA-HT-7474-01A			(19149.00)			0.00	114.00				NO	YES	NO				Seizures	0 - 30 Days	Scheduled Follow-up Submission	Stable Disease	NO		40969.00	MALE	NO	NO	Oligoastrocytoma
247	TCGA-HT-7475-01	0.00	TCGA-HT-7475-01	9.83	530.00	0.00	days	TCGA-HT-7475	530.00	0.00	days	530.00	days	TCGA Lower Grade Glioma (LGG)	brain lower grade glioma	Brain					67.00	NO		NO	TCGA-HT-7475-F29411	TCGA-HT-7475	TCGA-HT-7475-01A			(24686.00)			0.00	530.00		209.00		NO	YES	NO				Seizures	0 - 30 Days	Scheduled Follow-up Submission	Complete Remission/Response	NO		40984.00	MALE	NO	NO	Oligoastrocytoma
248	TCGA-HT-7476-01	0.00	TCGA-HT-7476-01	9.95	199.00	0.00	days	TCGA-HT-7476	199.00	0.00	days	199.00	days	TCGA Lower Grade Glioma (LGG)	brain lower grade glioma	Brain					26.00	NO		NO	TCGA-HT-7476-F21240	TCGA-HT-7476	TCGA-HT-7476-01A			(9614.00)			0.00	199.00				NO	YES	NO				Seizures	31 - 90 Days	Scheduled Follow-up Submission		NO		40946.00	MALE	NO	YES	Astrocytoma
249	TCGA-HT-7477-01	0.00	TCGA-HT-7477-01	9.03	738.00	0.00	days	TCGA-HT-7477	738.00	0.00	days	738.00	days	TCGA Lower Grade Glioma (LGG)	brain lower grade glioma	Brain					62.00	NO		NO	TCGA-HT-7477-F45880	TCGA-HT-7477	TCGA-HT-7477-01B			(22948.00)	417.00		0.00	738.00		24.00		NO	NO	NO				Motor/Movement Changes	31 - 90 Days	Scheduled Follow-up Submission	Complete Remission/Response	NO		41479.00	MALE	NO	NO	Astrocytoma
250	TCGA-HT-7478-01	0.00	TCGA-HT-7478-01	9.58	194.00	0.00	days	TCGA-HT-7478	194.00	0.00	days	194.00	days	TCGA Lower Grade Glioma (LGG)	brain lower grade glioma	Brain					36.00	NO		NO	TCGA-HT-7478-F29409	TCGA-HT-7478	TCGA-HT-7478-01A			(13216.00)			0.00	194.00				NO						Seizures	> 181 Days	Scheduled Follow-up Submission	Complete Remission/Response	NO		40984.00	MALE	NO	YES	Astrocytoma

251	TCGA-HT-7479-01	0.00	TCGA-HT-7479-01	10.69	1227.00	0.00	days	TCGA-HT-7479	705.00	1.00	days	1227.00	days	TCGA Lower Grade Glioma (LGG)	brain lower grade glioma	Brain	NO	NO	YES		44.00	NO		NO	TCGA-HT-7479-F66425	TCGA-HT-7479	TCGA-HT-7479-01A	705.00		(16137.00)			0.00	1227.00	705.00			NO	YES	YES				Headaches	> 181 Days	Scheduled Follow-up Submission	Complete Remission/Response	NO		41926.00	MALE	YES	YES	Astrocytoma
252	TCGA-HT-7480-01	0.00	TCGA-HT-7480-01	9.07	2287.00	0.00	days	TCGA-HT-7480	1943.00	1.00	days	2287.00	days	TCGA Lower Grade Glioma (LGG)	brain lower grade glioma	Brain	YES	NO	YES		33.00	NO		NO	TCGA-HT-7480-F28787	TCGA-HT-7480	TCGA-HT-7480-01A	1943.00		(12291.00)			0.00	2287.00	1943.00			NO	YES	NO				Seizures	> 181 Days	Scheduled Follow-up Submission	Progressive Disease	NO		40969.00	MALE	YES	NO	Oligodendroglioma
253	TCGA-HT-7481-01	0.00	TCGA-HT-7481-01	10.54	2918.00	0.00	days	TCGA-HT-7481	3071.00	1.00	days	2918.00	days	TCGA Lower Grade Glioma (LGG)	brain lower grade glioma	Brain	NO	NO	YES		39.00	NO		NO	TCGA-HT-7481-F66422	TCGA-HT-7481	TCGA-HT-7481-01A	3071.00		(14306.00)			0.00	2918.00	3071.00			NO	NO	NO				Seizures	0 - 30 Days	Scheduled Follow-up Submission	Partial Remission/Response	NO		41926.00	MALE	NO	YES	Oligodendroglioma
254	TCGA-HT-7482-01	0.00	TCGA-HT-7482-01	11.02	3253.00	0.00	days	TCGA-HT-7482	374.00	1.00	days	3253.00	days	TCGA Lower Grade Glioma (LGG)	brain lower grade glioma	Brain	NO	NO	YES		18.00	NO		NO	TCGA-HT-7482-F66423	TCGA-HT-7482	TCGA-HT-7482-01A	377.00		(6857.00)			0.00	3253.00	374.00			NO	NO	NO				Seizures	0 - 30 Days	Scheduled Follow-up Submission	Complete Remission/Response	NO		41926.00	FEMALE	NO	NO	Oligoastrocytoma
255	TCGA-HT-7483-01	0.00	TCGA-HT-7483-01	10.50	5255.00	0.00	days	TCGA-HT-7483	5255.00	0.00	days	5255.00	days	TCGA Lower Grade Glioma (LGG)	brain lower grade glioma	Brain					14.00	NO		NO	TCGA-HT-7483-F45917	TCGA-HT-7483	TCGA-HT-7483-01A			(5267.00)			0.00	5255.00				NO	YES	NO				Seizures		Scheduled Follow-up Submission	Complete Remission/Response	NO		41480.00	MALE	NO	YES	Oligoastrocytoma
256	TCGA-HT-7485-01	0.00	TCGA-HT-7485-01	10.38	122.00	0.00	days	TCGA-HT-7485	122.00	0.00	days	122.00	days	TCGA Lower Grade Glioma (LGG)	brain lower grade glioma	Brain					42.00	NO		NO	TCGA-HT-7485-F28639	TCGA-HT-7485	TCGA-HT-7485-01A			(15589.00)			0.00	122.00		24.00		NO	NO	NO				Headaches	91 - 180 Days	Scheduled Follow-up Submission	Complete Remission/Response	NO		40970.00	MALE	NO	YES	Astrocytoma
257	TCGA-HT-7601-01	0.00	TCGA-HT-7601-01	11.07	153.00	0.00	days	TCGA-HT-7601	153.00	0.00	days	153.00	days	TCGA Lower Grade Glioma (LGG)	brain lower grade glioma	Brain					30.00	NO		NO	TCGA-HT-7601-F29577	TCGA-HT-7601	TCGA-HT-7601-01A			(10989.00)			0.00	153.00				NO	NO	NO				Seizures	0 - 30 Days	Scheduled Follow-up Submission		NO		40976.00	FEMALE	NO	NO	Astrocytoma
258	TCGA-HT-7602-01	0.00	TCGA-HT-7602-01	11.15	908.00	0.00	days	TCGA-HT-7602			days	908.00	days	TCGA Lower Grade Glioma (LGG)	brain lower grade glioma	Brain					21.00	NO		NO	TCGA-HT-7602-F29573	TCGA-HT-7602	TCGA-HT-7602-01A			(7946.00)			0.00	908.00				NO	NO	NO				Headaches	0 - 30 Days	Scheduled Follow-up Submission		NO		40984.00	MALE	NO	YES	Oligodendroglioma
259	TCGA-HT-7603-01	0.00	TCGA-HT-7603-01	8.37	705.00	0.00	days	TCGA-HT-7603	705.00	0.00	days	705.00	days	TCGA Lower Grade Glioma (LGG)	brain lower grade glioma	Brain					29.00	NO		NO	TCGA-HT-7603-F29571	TCGA-HT-7603	TCGA-HT-7603-01A			(10883.00)			0.00	705.00				NO	NO	NO				Headaches	0 - 30 Days	Scheduled Follow-up Submission		NO		40975.00	MALE	NO	YES	Oligodendroglioma
260	TCGA-HT-7604-01	0.00	TCGA-HT-7604-01	8.06	3725.00	0.00	days	TCGA-HT-7604	3725.00	0.00	days	3725.00	days	TCGA Lower Grade Glioma (LGG)	brain lower grade glioma	Brain					50.00	NO		NO	TCGA-HT-7604-F48641	TCGA-HT-7604	TCGA-HT-7604-01A			(18443.00)			0.00	3725.00				NO	NO	NO				Headaches	31 - 90 Days	Scheduled Follow-up Submission	Partial Remission/Response	NO		41535.00	MALE	NO	YES	Astrocytoma
261	TCGA-HT-7605-01	0.00	TCGA-HT-7605-01	10.33	139.00	0.00	days	TCGA-HT-7605	139.00	0.00	days	139.00	days	TCGA Lower Grade Glioma (LGG)	brain lower grade glioma	Brain					38.00	NO		NO	TCGA-HT-7605-F29603	TCGA-HT-7605	TCGA-HT-7605-01A			(13892.00)			0.00	139.00				NO	YES	NO	< 12 Years			Motor/Movement Changes		Scheduled Follow-up Submission		NO		40983.00	MALE	YES	NO	Oligodendroglioma
262	TCGA-HT-7606-01	0.00	TCGA-HT-7606-01	11.03	526.00	0.00	days	TCGA-HT-7606	526.00	0.00	days	526.00	days	TCGA Lower Grade Glioma (LGG)	brain lower grade glioma	Brain					30.00	NO			TCGA-HT-7606-F29692	TCGA-HT-7606	TCGA-HT-7606-01A			(11233.00)			0.00	526.00				NO	NO	NO				Headaches	> 181 Days	Scheduled Follow-up Submission		NO		40995.00	FEMALE	NO	YES	Astrocytoma
263	TCGA-HT-7607-01	1.00	TCGA-HT-7607-01	10.71	96.00	1.00	days	TCGA-HT-7607	96.00	0.00	days	96.00	days	TCGA Lower Grade Glioma (LGG)	brain lower grade glioma	Brain					61.00	NO		NO	TCGA-HT-7607-F35003	TCGA-HT-7607	TCGA-HT-7607-01A			(22585.00)		96.00	0.00			8.00		NO	NO	NO				Seizures	31 - 90 Days	Scheduled Follow-up Submission	Progressive Disease	NO		41164.00	FEMALE	YES	YES	Astrocytoma
264	TCGA-HT-7608-01	0.00	TCGA-HT-7608-01	11.03	671.00	0.00	days	TCGA-HT-7608	671.00	0.00	days	671.00	days	TCGA Lower Grade Glioma (LGG)	brain lower grade glioma	Brain					61.00	NO		NO	TCGA-HT-7608-F28803	TCGA-HT-7608	TCGA-HT-7608-01A			(22302.00)			0.00	671.00				NO	NO	NO				Motor/Movement Changes	0 - 30 Days	Scheduled Follow-up Submission	Stable Disease	NO		40969.00	MALE	NO	NO	Oligoastrocytoma
265	TCGA-HT-7609-01	0.00	TCGA-HT-7609-01	9.83	1399.00	0.00	days	TCGA-HT-7609	1399.00	0.00	days	1399.00	days	TCGA Lower Grade Glioma (LGG)	brain lower grade glioma	Brain					34.00	NO		YES	TCGA-HT-7609-F28823	TCGA-HT-7609	TCGA-HT-7609-01A			(12607.00)			0.00	1399.00		1.00		NO	NO	NO				Headaches	31 - 90 Days	Scheduled Follow-up Submission	Stable Disease	NO		40969.00	MALE	NO	YES	Oligoastrocytoma
266	TCGA-HT-7610-01	0.00	TCGA-HT-7610-01	8.98	1706.00	0.00	days	TCGA-HT-7610	1205.00	1.00	days	1706.00	days	TCGA Lower Grade Glioma (LGG)	brain lower grade glioma	Brain	NO	YES	YES		25.00	NO		NO	TCGA-HT-7610-F28944	TCGA-HT-7610	TCGA-HT-7610-01A	1205.00		(9447.00)			0.00	1706.00	1205.00			NO	NO	NO				Seizures	> 181 Days	Scheduled Follow-up Submission	Stable Disease	NO		40970.00	FEMALE	NO	YES	Oligoastrocytoma
267	TCGA-HT-7611-01	0.00	TCGA-HT-7611-01	9.72	1752.00	0.00	days	TCGA-HT-7611	1752.00	0.00	days	1752.00	days	TCGA Lower Grade Glioma (LGG)	brain lower grade glioma	Brain					36.00	NO		NO	TCGA-HT-7611-F51026	TCGA-HT-7611	TCGA-HT-7611-01A			(13314.00)			0.00	1752.00				NO	NO	NO						Scheduled Follow-up Submission		NO		41583.00	MALE	NO	NO	Oligoastrocytoma
268	TCGA-HT-7616-01	1.00	TCGA-HT-7616-01	9.20	7.00	1.00	days	TCGA-HT-7616	7.00	0.00	days	7.00	days	TCGA Lower Grade Glioma (LGG)	brain lower grade glioma	Brain					75.00	NO		NO	TCGA-HT-7616-F28640	TCGA-HT-7616	TCGA-HT-7616-01A			(27685.00)		7.00	0.00					NO	NO	NO				Seizures	0 - 30 Days	Scheduled Follow-up Submission	Progressive Disease			40966.00	MALE	NO	NO	Oligodendroglioma
269	TCGA-HT-7620-01	0.00	TCGA-HT-7620-01	10.66	434.00	0.00	days	TCGA-HT-7620	280.00	1.00	days	434.00	days	TCGA Lower Grade Glioma (LGG)	brain lower grade glioma	Brain	YES	NO	NO		40.00	NO		NO	TCGA-HT-7620-F29125	TCGA-HT-7620	TCGA-HT-7620-01A			(14659.00)			0.00	434.00	280.00			NO	NO	NO				Headaches	0 - 30 Days	Scheduled Follow-up Submission	Progressive Disease	NO		40976.00	MALE	YES	YES	Oligodendroglioma
270	TCGA-HT-7676-01	0.00	TCGA-HT-7676-01	9.13	5.00	0.00	days	TCGA-HT-7676			days	5.00	days	TCGA Lower Grade Glioma (LGG)	brain lower grade glioma	Brain					26.00				TCGA-HT-7676-F51023	TCGA-HT-7676	TCGA-HT-7676-01A			(9667.00)			0.00	5.00										Headaches	0 - 30 Days	Scheduled Follow-up Submission				41583.00	MALE		YES	Oligodendroglioma
271	TCGA-HT-7677-01	0.00	TCGA-HT-7677-01	9.65	494.00	0.00	days	TCGA-HT-7677	494.00	0.00	days	494.00	days	TCGA Lower Grade Glioma (LGG)	brain lower grade glioma	Brain					53.00	NO		NO	TCGA-HT-7677-F28945	TCGA-HT-7677	TCGA-HT-7677-01A			(19610.00)			0.00	494.00				NO	NO	NO				Motor/Movement Changes	> 181 Days	Scheduled Follow-up Submission	Stable Disease	NO		40970.00	MALE	NO	NO	Oligodendroglioma
272	TCGA-HT-7680-01	0.00	TCGA-HT-7680-01	10.51	23.00	0.00	days	TCGA-HT-7680	23.00	0.00	days	23.00	days	TCGA Lower Grade Glioma (LGG)	brain lower grade glioma	Brain	NO	NO			32.00	NO		NO	TCGA-HT-7680-F28949	TCGA-HT-7680	TCGA-HT-7680-01A			(11970.00)			0.00	23.00				NO	NO	NO				Headaches	> 181 Days	Scheduled Follow-up Submission	Stable Disease	NO		40970.00	FEMALE	NO	YES	Astrocytoma
273	TCGA-HT-7681-01	0.00	TCGA-HT-7681-01	9.90	1359.00	0.00	days	TCGA-HT-7681	1359.00	0.00	days	1359.00	days	TCGA Lower Grade Glioma (LGG)	brain lower grade glioma	Brain					29.00				TCGA-HT-7681-F45882	TCGA-HT-7681	TCGA-HT-7681-01A			(10790.00)			0.00	1359.00												Scheduled Follow-up Submission	Complete Remission/Response			41479.00	FEMALE			Oligoastrocytoma
274	TCGA-HT-7684-01	0.00	TCGA-HT-7684-01	11.30	184.00	0.00	days	TCGA-HT-7684	184.00	0.00	days	184.00	days	TCGA Lower Grade Glioma (LGG)	brain lower grade glioma	Brain					58.00	NO		NO	TCGA-HT-7684-F29412	TCGA-HT-7684	TCGA-HT-7684-01A			(21352.00)			0.00	184.00				NO	NO	NO				Headaches	0 - 30 Days	Scheduled Follow-up Submission	Complete Remission/Response	NO		40984.00	MALE	NO	YES	Oligoastrocytoma
275	TCGA-HT-7686-01	0.00	TCGA-HT-7686-01	9.76	1300.00	0.00	days	TCGA-HT-7686	1300.00	0.00	days	1300.00	days	TCGA Lower Grade Glioma (LGG)	brain lower grade glioma	Brain					29.00	NO		NO	TCGA-HT-7686-F29415	TCGA-HT-7686	TCGA-HT-7686-01A			(10724.00)			0.00	1300.00		231.00		NO						Headaches	> 181 Days	Scheduled Follow-up Submission	Complete Remission/Response	NO		40984.00	FEMALE	NO	YES	Astrocytoma
276	TCGA-HT-7687-01	0.00	TCGA-HT-7687-01	9.14	3.00	0.00	days	TCGA-HT-7687			days	3.00	days	TCGA Lower Grade Glioma (LGG)	brain lower grade glioma	Brain					74.00				TCGA-HT-7687-F28642	TCGA-HT-7687	TCGA-HT-7687-01A			(27186.00)			0.00	3.00					YES	NO				Visual Changes	> 181 Days	Scheduled Follow-up Submission				40966.00	MALE		NO	Oligodendroglioma
277	TCGA-HT-7688-01	0.00	TCGA-HT-7688-01	8.93	964.00	0.00	days	TCGA-HT-7688	964.00	0.00	days	964.00	days	TCGA Lower Grade Glioma (LGG)	brain lower grade glioma	Brain					59.00	NO		NO	TCGA-HT-7688-F29413	TCGA-HT-7688	TCGA-HT-7688-01A			(21844.00)			0.00	964.00				NO	YES	NO				Headaches	> 181 Days	Scheduled Follow-up Submission	Complete Remission/Response	NO		40984.00	MALE	YES	YES	Oligodendroglioma
278	TCGA-HT-7689-01	0.00	TCGA-HT-7689-01	10.63	455.00	0.00	days	TCGA-HT-7689	455.00	0.00	days	455.00	days	TCGA Lower Grade Glioma (LGG)	brain lower grade glioma	Brain					58.00	NO		NO	TCGA-HT-7689-F29648	TCGA-HT-7689	TCGA-HT-7689-01A			(21331.00)			0.00	455.00		455.00		NO	NO	NO				Mental Status Changes	0 - 30 Days	Scheduled Follow-up Submission		NO		40995.00	FEMALE	NO	NO	Oligodendroglioma
279	TCGA-HT-7690-01	0.00	TCGA-HT-7690-01	10.83	3.00	0.00	days	TCGA-HT-7690	3.00	0.00	days	3.00	days	TCGA Lower Grade Glioma (LGG)	brain lower grade glioma	Brain					29.00	NO		NO	TCGA-HT-7690-F29644	TCGA-HT-7690	TCGA-HT-7690-01A			(10673.00)			0.00	3.00				NO	NO	NO				Headaches	31 - 90 Days	Scheduled Follow-up Submission		NO		40983.00	MALE	NO	YES	Oligoastrocytoma
280	TCGA-HT-7691-01	0.00	TCGA-HT-7691-01	10.08	3.00	0.00	days	TCGA-HT-7691	3.00	0.00	days	3.00	days	TCGA Lower Grade Glioma (LGG)	brain lower grade glioma	Brain					31.00	NO		NO	TCGA-HT-7691-F29641	TCGA-HT-7691	TCGA-HT-7691-01A			(11465.00)			0.00	3.00				NO	NO	NO				Headaches	> 181 Days	Scheduled Follow-up Submission		NO		40995.00	FEMALE	NO	YES	Astrocytoma
281	TCGA-HT-7692-01	0.00	TCGA-HT-7692-01	11.17	90.00	0.00	days	TCGA-HT-7692	90.00	0.00	days	90.00	days	TCGA Lower Grade Glioma (LGG)	brain lower grade glioma	Brain					43.00	NO		NO	TCGA-HT-7692-F29616	TCGA-HT-7692	TCGA-HT-7692-01A			(15727.00)			0.00	90.00		29.00		NO	YES	YES				Seizures	0 - 30 Days	Scheduled Follow-up Submission		NO		40984.00	MALE	NO	YES	Oligoastrocytoma
282	TCGA-HT-7693-01	0.00	TCGA-HT-7693-01	11.08	533.00	0.00	days	TCGA-HT-7693	533.00	0.00	days	533.00	days	TCGA Lower Grade Glioma (LGG)	brain lower grade glioma	Brain					51.00	NO		YES	TCGA-HT-7693-F29610	TCGA-HT-7693	TCGA-HT-7693-01A			(18956.00)			0.00	533.00		472.00		NO	YES	NO				Seizures	0 - 30 Days	Scheduled Follow-up Submission		NO		40984.00	FEMALE	NO	YES	Oligodendroglioma
283	TCGA-HT-7694-01	0.00	TCGA-HT-7694-01	10.03	210.00	0.00	days	TCGA-HT-7694	210.00	0.00	days	210.00	days	TCGA Lower Grade Glioma (LGG)	brain lower grade glioma	Brain					60.00	NO		NO	TCGA-HT-7694-F29607	TCGA-HT-7694	TCGA-HT-7694-01A			(21930.00)			0.00	210.00		210.00		NO	YES	NO				Motor/Movement Changes	0 - 30 Days	Scheduled Follow-up Submission		NO		40977.00	MALE	NO	NO	Oligodendroglioma
284	TCGA-HT-7695-01	0.00	TCGA-HT-7695-01	10.08	442.00	0.00	days	TCGA-HT-7695	442.00	0.00	days	442.00	days	TCGA Lower Grade Glioma (LGG)	brain lower grade glioma	Brain					29.00	NO		NO	TCGA-HT-7695-F29604	TCGA-HT-7695	TCGA-HT-7695-01A			(10827.00)			0.00	442.00				NO	YES	NO				Seizures	31 - 90 Days	Scheduled Follow-up Submission		NO		40984.00	FEMALE	NO	NO	Oligodendroglioma
285	TCGA-HT-7854-01	0.00	TCGA-HT-7854-01	10.56	1201.00	0.00	days	TCGA-HT-7854	1147.00	1.00	days	1201.00	days	TCGA Lower Grade Glioma (LGG)	brain lower grade glioma	Brain	NO	NO		YES	62.00	NO		NO	TCGA-HT-7854-F48001	TCGA-HT-7854	TCGA-HT-7854-01A		1191.00	(22738.00)			0.00	1201.00	1147.00	208.00		NO	NO	NO				Visual Changes	31 - 90 Days	Scheduled Follow-up Submission		NO		41520.00	MALE	NO	YES	Astrocytoma
286	TCGA-HT-7855-01	0.00	TCGA-HT-7855-01	9.58	585.00	0.00	days	TCGA-HT-7855	585.00	0.00	days	585.00	days	TCGA Lower Grade Glioma (LGG)	brain lower grade glioma	Brain					39.00	NO		NO	TCGA-HT-7855-F29420	TCGA-HT-7855	TCGA-HT-7855-01A			(14496.00)			0.00	585.00		151.00		NO	NO	NO				Headaches	91 - 180 Days	Scheduled Follow-up Submission	Complete Remission/Response	NO		40984.00	MALE	NO	YES	Astrocytoma

287	TCGA-HT-7856-01	0.00	TCGA-HT-7856-01	9.95	1189.00	0.00	days	TCGA-HT-7856	1189.00	0.00	days	1189.00	days	TCGA Lower Grade Glioma (LGG)	brain lower grade glioma	Brain					35.00	NO		NO	TCGA-HT-7856-F29423	TCGA-HT-7856	TCGA-HT-7856-01A			(13145.00)			0.00	1189.00		383.00		NO	NO	NO				Mental Status Changes	> 181 Days	Scheduled Follow-up Submission	Complete Remission/Response	NO		40984.00	MALE	NO	YES	Oligodendroglioma
288	TCGA-HT-7857-01	0.00	TCGA-HT-7857-01	7.44	7.00	0.00	days	TCGA-HT-7857	7.00	0.00	days	7.00	days	TCGA Lower Grade Glioma (LGG)	brain lower grade glioma	Brain					24.00	NO		NO	TCGA-HT-7857-F29669	TCGA-HT-7857	TCGA-HT-7857-01A			(8912.00)			0.00	7.00				NO						Motor/Movement Changes	0 - 30 Days	Scheduled Follow-up Submission		NO		40983.00	FEMALE	NO	YES	Astrocytoma
289	TCGA-HT-7858-01	0.00	TCGA-HT-7858-01	9.29	1540.00	0.00	days	TCGA-HT-7858	1540.00	0.00	days	1540.00	days	TCGA Lower Grade Glioma (LGG)	brain lower grade glioma	Brain					28.00	NO		NO	TCGA-HT-7858-F29426	TCGA-HT-7858	TCGA-HT-7858-01A			(10422.00)			0.00	1540.00		673.00		NO	NO	NO				Headaches	> 181 Days	Scheduled Follow-up Submission	Complete Remission/Response	NO		40984.00	MALE	NO	YES	Astrocytoma
290	TCGA-HT-7860-01	0.00	TCGA-HT-7860-01	10.39	15.00	0.00	days	TCGA-HT-7860			days	15.00	days	TCGA Lower Grade Glioma (LGG)	brain lower grade glioma	Brain					60.00	NO		NO	TCGA-HT-7860-F28643	TCGA-HT-7860	TCGA-HT-7860-01A			(22034.00)			0.00	15.00				NO						Seizures	> 181 Days	Scheduled Follow-up Submission		NO		40966.00	FEMALE	NO	NO	Astrocytoma
291	TCGA-HT-7873-01	0.00	TCGA-HT-7873-01	10.89	718.00	0.00	days	TCGA-HT-7873	718.00	0.00	days	718.00	days	TCGA Lower Grade Glioma (LGG)	brain lower grade glioma	Brain	NO	NO			29.00	NO		NO	TCGA-HT-7873-F48004	TCGA-HT-7873	TCGA-HT-7873-01B			(10805.00)			0.00	718.00				NO	NO	NO				Headaches	> 181 Days	Scheduled Follow-up Submission				41520.00	MALE	NO	YES	Oligoastrocytoma
292	TCGA-HT-7874-01	0.00	TCGA-HT-7874-01	9.46	1130.00	0.00	days	TCGA-HT-7874	1130.00	0.00	days	1130.00	days	TCGA Lower Grade Glioma (LGG)	brain lower grade glioma	Brain					41.00	NO		NO	TCGA-HT-7874-F29422	TCGA-HT-7874	TCGA-HT-7874-01A			(15045.00)			0.00	1130.00				NO	YES	NO				Headaches	> 181 Days	Scheduled Follow-up Submission	Complete Remission/Response	NO		40984.00	FEMALE	NO	YES	Oligodendroglioma
293	TCGA-HT-7875-01	0.00	TCGA-HT-7875-01	8.84	10.00	0.00	days	TCGA-HT-7875	10.00	0.00	days	10.00	days	TCGA Lower Grade Glioma (LGG)	brain lower grade glioma	Brain					56.00	NO		NO	TCGA-HT-7875-F28953	TCGA-HT-7875	TCGA-HT-7875-01A			(20613.00)			0.00	10.00		0.00		NO	YES	NO				Seizures	91 - 180 Days	Scheduled Follow-up Submission	Stable Disease	NO		40970.00	MALE	NO	NO	Oligodendroglioma
294	TCGA-HT-7877-01	0.00	TCGA-HT-7877-01	9.22	4.00	0.00	days	TCGA-HT-7877	4.00	0.00	days	4.00	days	TCGA Lower Grade Glioma (LGG)	brain lower grade glioma	Brain					20.00	NO		NO	TCGA-HT-7877-F29672	TCGA-HT-7877	TCGA-HT-7877-01A			(7581.00)			0.00	4.00				NO	NO	NO				Headaches	> 181 Days	Scheduled Follow-up Submission		NO		40987.00	FEMALE	YES	YES	Oligodendroglioma
295	TCGA-HT-7879-01	0.00	TCGA-HT-7879-01	10.49	112.00	0.00	days	TCGA-HT-7879	112.00	0.00	days	112.00	days	TCGA Lower Grade Glioma (LGG)	brain lower grade glioma	Brain					31.00	NO		NO	TCGA-HT-7879-F29673	TCGA-HT-7879	TCGA-HT-7879-01A			(11622.00)			0.00	112.00				NO	NO	NO				Seizures	0 - 30 Days	Scheduled Follow-up Submission		NO		40987.00	MALE	NO	YES	Oligoastrocytoma
296	TCGA-HT-7880-01	0.00	TCGA-HT-7880-01	10.57	162.00	0.00	days	TCGA-HT-7880	162.00	0.00	days	162.00	days	TCGA Lower Grade Glioma (LGG)	brain lower grade glioma	Brain					30.00	NO		NO	TCGA-HT-7880-F29680	TCGA-HT-7880	TCGA-HT-7880-01A			(11043.00)			0.00	162.00				NO	NO	NO				Seizures	31 - 90 Days	Scheduled Follow-up Submission		YES	peanuts	40987.00	MALE	NO	YES	Oligoastrocytoma
297	TCGA-HT-7881-01	0.00	TCGA-HT-7881-01	10.03	1079.00	0.00	days	TCGA-HT-7881	1079.00	0.00	days	1079.00	days	TCGA Lower Grade Glioma (LGG)	brain lower grade glioma	Brain					38.00	NO		NO	TCGA-HT-7881-F66426	TCGA-HT-7881	TCGA-HT-7881-01A			(14171.00)			0.00	1079.00				NO	YES	NO				Seizures	> 181 Days	Scheduled Follow-up Submission	Complete Remission/Response			41926.00	MALE	NO	NO	Oligodendroglioma
298	TCGA-HT-7882-01	1.00	TCGA-HT-7882-01	12.01	113.00	1.00	days	TCGA-HT-7882	113.00	0.00	days	113.00	days	TCGA Lower Grade Glioma (LGG)	brain lower grade glioma	Brain					66.00	NO		NO	TCGA-HT-7882-F28952	TCGA-HT-7882	TCGA-HT-7882-01A			(24450.00)		113.00	0.00	52.00				NO	NO	NO				Seizures	> 181 Days	Scheduled Follow-up Submission	Progressive Disease	NO		40970.00	MALE	NO	NO	Oligodendroglioma
299	TCGA-HT-7884-01	0.00	TCGA-HT-7884-01	8.55	343.00	0.00	days	TCGA-HT-7884	343.00	0.00	days	343.00	days	TCGA Lower Grade Glioma (LGG)	brain lower grade glioma	Brain					44.00	NO		YES	TCGA-HT-7884-F35005	TCGA-HT-7884	TCGA-HT-7884-01B			(16280.00)			0.00	343.00		112.00		NO	NO	NO	< 12 Years			Mental Status Changes	0 - 30 Days	Scheduled Follow-up Submission	Stable Disease	NO		41164.00	FEMALE	NO	YES	Astrocytoma
300	TCGA-HT-7902-01	0.00	TCGA-HT-7902-01	10.34	956.00	0.00	days	TCGA-HT-7902	956.00	0.00	days	956.00	days	TCGA Lower Grade Glioma (LGG)	brain lower grade glioma	Brain					30.00	NO		YES	TCGA-HT-7902-F28951	TCGA-HT-7902	TCGA-HT-7902-01A			(11118.00)			0.00	956.00				NO	YES	YES				Visual Changes	31 - 90 Days	Scheduled Follow-up Submission	Stable Disease	NO		40970.00	FEMALE	NO	YES	Oligoastrocytoma
301	TCGA-HT-8010-01	0.00	TCGA-HT-8010-01	10.77	50.00	0.00	days	TCGA-HT-8010	50.00	0.00	days	50.00	days	TCGA Lower Grade Glioma (LGG)	brain lower grade glioma	Brain					64.00	NO		NO	TCGA-HT-8010-F32622	TCGA-HT-8010	TCGA-HT-8010-01A			(23680.00)			0.00	50.00				NO	YES	NO				Motor/Movement Changes	> 181 Days	Scheduled Follow-up Submission	Stable Disease	NO		41064.00	FEMALE	NO	YES	Oligodendroglioma
302	TCGA-HT-8011-01	0.00	TCGA-HT-8011-01	9.50	494.00	0.00	days	TCGA-HT-8011	278.00	1.00	days	494.00	days	TCGA Lower Grade Glioma (LGG)	brain lower grade glioma	Brain	NO	NO	YES	NO	55.00	NO		NO	TCGA-HT-8011-F32623	TCGA-HT-8011	TCGA-HT-8011-01A	278.00		(20175.00)			0.00	494.00	278.00			NO	YES	YES				Seizures	0 - 30 Days	Scheduled Follow-up Submission	Progressive Disease	NO		41064.00	MALE	NO	YES	Astrocytoma
303	TCGA-HT-8012-01	0.00	TCGA-HT-8012-01	8.73	286.00	0.00	days	TCGA-HT-8012	133.00	1.00	days	286.00	days	TCGA Lower Grade Glioma (LGG)	brain lower grade glioma	Brain	NO	NO	YES	NO	30.00	NO		NO	TCGA-HT-8012-F32626	TCGA-HT-8012	TCGA-HT-8012-01A	133.00		(11295.00)			0.00	286.00	133.00			NO	NO	NO				Seizures	0 - 30 Days	Scheduled Follow-up Submission	Stable Disease	NO		41064.00	FEMALE	NO	NO	Oligodendroglioma
304	TCGA-HT-8013-01	1.00	TCGA-HT-8013-01	8.83	1933.00	1.00	days	TCGA-HT-8013	1306.00	1.00	days	1933.00	days	TCGA Lower Grade Glioma (LGG)	brain lower grade glioma	Brain	YES	YES	YES	NO	37.00	NO		NO	TCGA-HT-8013-F32627	TCGA-HT-8013	TCGA-HT-8013-01A	1306.00		(13753.00)		1933.00	0.00		1306.00	1587.00		NO	NO	NO				Seizures	0 - 30 Days	Scheduled Follow-up Submission		NO		41064.00	FEMALE	NO	NO	Oligoastrocytoma
305	TCGA-HT-8015-01	0.00	TCGA-HT-8015-01	10.46	3.00	0.00	days	TCGA-HT-8015			days	3.00	days	TCGA Lower Grade Glioma (LGG)	brain lower grade glioma	Brain					21.00					TCGA-HT-8015	TCGA-HT-8015-01B			(7760.00)	1483.00		0.00	3.00																41529.00	MALE			Astrocytoma
306	TCGA-HT-8018-01	1.00	TCGA-HT-8018-01	9.72	1152.00	1.00	days	TCGA-HT-8018	1152.00	0.00	days	1152.00	days	TCGA Lower Grade Glioma (LGG)	brain lower grade glioma	Brain					40.00	NO		NO	TCGA-HT-8018-F45912	TCGA-HT-8018	TCGA-HT-8018-01A			(14910.00)		1152.00	0.00	654.00				NO	NO	NO				Seizures	0 - 30 Days	Scheduled Follow-up Submission	Progressive Disease	NO		41480.00	FEMALE	NO	YES	Oligoastrocytoma
307	TCGA-HT-8019-01	0.00	TCGA-HT-8019-01	9.45	1004.00	0.00	days	TCGA-HT-8019	1004.00	0.00	days	1004.00	days	TCGA Lower Grade Glioma (LGG)	brain lower grade glioma	Brain					34.00	NO		NO	TCGA-HT-8019-F46178	TCGA-HT-8019	TCGA-HT-8019-01A			(12669.00)			0.00	1004.00				NO	YES	NO				Seizures	0 - 30 Days	Scheduled Follow-up Submission	Complete Remission/Response	NO		41535.00	FEMALE	NO	YES	Oligodendroglioma
308	TCGA-HT-8104-01	0.00	TCGA-HT-8104-01	11.42	372.00	0.00	days	TCGA-HT-8104			days	372.00	days	TCGA Lower Grade Glioma (LGG)	brain lower grade glioma	Brain					51.00	NO		NO	TCGA-HT-8104-F45987	TCGA-HT-8104	TCGA-HT-8104-01A			(18748.00)			0.00	372.00				NO	NO	NO				Motor/Movement Changes	0 - 30 Days	Scheduled Follow-up Submission		NO		41481.00	FEMALE	NO	NO	Astrocytoma
309	TCGA-HT-8105-01	0.00	TCGA-HT-8105-01	9.60	190.00	0.00	days	TCGA-HT-8105	190.00	0.00	days	190.00	days	TCGA Lower Grade Glioma (LGG)	brain lower grade glioma	Brain					54.00	NO		NO	TCGA-HT-8105-F32624	TCGA-HT-8105	TCGA-HT-8105-01A			(20041.00)			0.00	190.00		190.00		NO	NO	NO				Mental Status Changes	31 - 90 Days	Scheduled Follow-up Submission	Stable Disease	NO		41064.00	MALE	NO	NO	Oligodendroglioma
310	TCGA-HT-8106-01	0.00	TCGA-HT-8106-01	12.41	3.00	0.00	days	TCGA-HT-8106	3.00	0.00	days	3.00	days	TCGA Lower Grade Glioma (LGG)	brain lower grade glioma	Brain					53.00	NO		NO	TCGA-HT-8106-F32632	TCGA-HT-8106	TCGA-HT-8106-01A			(19555.00)			0.00	3.00				NO	NO	NO				Seizures	0 - 30 Days	Scheduled Follow-up Submission	Stable Disease	NO		41064.00	MALE	NO	NO	Astrocytoma
311	TCGA-HT-8107-01	0.00	TCGA-HT-8107-01	8.94	14.00	0.00	days	TCGA-HT-8107	14.00	0.00	days	14.00	days	TCGA Lower Grade Glioma (LGG)	brain lower grade glioma	Brain					62.00	NO		NO	TCGA-HT-8107-F32633	TCGA-HT-8107	TCGA-HT-8107-01A			(22880.00)			0.00	14.00				NO	NO	NO				Mental Status Changes	0 - 30 Days	Scheduled Follow-up Submission	Stable Disease	NO		41064.00	MALE	NO	NO	Oligodendroglioma
312	TCGA-HT-8108-01	0.00	TCGA-HT-8108-01	9.11	76.00	0.00	days	TCGA-HT-8108	76.00	0.00	days	76.00	days	TCGA Lower Grade Glioma (LGG)	brain lower grade glioma	Brain					26.00	NO		NO	TCGA-HT-8108-F32634	TCGA-HT-8108	TCGA-HT-8108-01A			(9539.00)			0.00	76.00				NO	YES	YES				Headaches	0 - 30 Days	Scheduled Follow-up Submission	Stable Disease	NO		41064.00	FEMALE	NO	YES	Oligodendroglioma
313	TCGA-HT-8109-01	0.00	TCGA-HT-8109-01	10.15	169.00	0.00	days	TCGA-HT-8109			days	169.00	days	TCGA Lower Grade Glioma (LGG)	brain lower grade glioma	Brain					64.00	NO		NO	TCGA-HT-8109-F45921	TCGA-HT-8109	TCGA-HT-8109-01A			(23486.00)			0.00	169.00				NO	NO	NO				Mental Status Changes	> 181 Days	Scheduled Follow-up Submission		NO		41480.00	MALE	NO	NO	Oligodendroglioma
314	TCGA-HT-8110-01	0.00	TCGA-HT-8110-01	11.26	419.00	0.00	days	TCGA-HT-8110	419.00	0.00	days	419.00	days	TCGA Lower Grade Glioma (LGG)	brain lower grade glioma	Brain					57.00	NO		NO	TCGA-HT-8110-F43794	TCGA-HT-8110	TCGA-HT-8110-01A			(21183.00)			0.00	419.00				NO	YES	NO				Visual Changes	0 - 30 Days	Scheduled Follow-up Submission	Complete Remission/Response	NO		41481.00	MALE	NO	YES	Astrocytoma
315	TCGA-HT-8111-01	0.00	TCGA-HT-8111-01	10.81	7.00	0.00	days	TCGA-HT-8111			days	7.00	days	TCGA Lower Grade Glioma (LGG)	brain lower grade glioma	Brain					32.00	NO		NO	TCGA-HT-8111-F66396	TCGA-HT-8111	TCGA-HT-8111-01A			(11810.00)			0.00	7.00				NO		NO				Seizures	0 - 30 Days	Scheduled Follow-up Submission		NO		41926.00	MALE	NO	YES	Oligoastrocytoma
316	TCGA-HT-8113-01	0.00	TCGA-HT-8113-01	9.58	900.00	0.00	days	TCGA-HT-8113	900.00	0.00	days	900.00	days	TCGA Lower Grade Glioma (LGG)	brain lower grade glioma	Brain					49.00	NO		YES	TCGA-HT-8113-F32197	TCGA-HT-8113	TCGA-HT-8113-01A			(18173.00)			0.00	900.00		102.00		NO	NO	NO				Seizures	0 - 30 Days	Scheduled Follow-up Submission		NO		41046.00	FEMALE	NO	YES	Oligodendroglioma
317	TCGA-HT-8114-01	0.00	TCGA-HT-8114-01	11.06	1040.00	0.00	days	TCGA-HT-8114	1040.00	0.00	days	1040.00	days	TCGA Lower Grade Glioma (LGG)	brain lower grade glioma	Brain					36.00	NO		NO	TCGA-HT-8114-F66394	TCGA-HT-8114	TCGA-HT-8114-01A			(13198.00)			0.00	1040.00				NO	NO	NO				Headaches	0 - 30 Days	Scheduled Follow-up Submission	Complete Remission/Response	NO		41926.00	MALE	NO	YES	Oligoastrocytoma
318	TCGA-HT-8558-01	0.00	TCGA-HT-8558-01	8.88	418.00	0.00	days	TCGA-HT-8558	418.00	0.00	days	418.00	days	TCGA Lower Grade Glioma (LGG)	brain lower grade glioma	Brain					29.00	NO		NO	TCGA-HT-8558-F48474	TCGA-HT-8558	TCGA-HT-8558-01A			(10950.00)			0.00	418.00										Seizures	31 - 90 Days	Scheduled Follow-up Submission	Complete Remission/Response	NO		41530.00	FEMALE	NO	YES	Oligodendroglioma
319	TCGA-HT-8563-01	0.00	TCGA-HT-8563-01	13.94	860.00	0.00	days	TCGA-HT-8563	860.00	0.00	days	860.00	days	TCGA Lower Grade Glioma (LGG)	brain lower grade glioma	Brain					30.00				TCGA-HT-8563-F61084	TCGA-HT-8563	TCGA-HT-8563-01A			(11305.00)			0.00	860.00						NO	> 20 Years			Headaches	0 - 30 Days	Scheduled Follow-up Submission	Complete Remission/Response			41813.00	FEMALE	YES	YES	Astrocytoma
320	TCGA-HT-8564-01	0.00	TCGA-HT-8564-01	9.19	478.00	0.00	days	TCGA-HT-8564	120.00	1.00	days	478.00	days	TCGA Lower Grade Glioma (LGG)	brain lower grade glioma	Brain	YES	NO			47.00	NO		NO	TCGA-HT-8564-F48467	TCGA-HT-8564	TCGA-HT-8564-01A			(17499.00)			0.00	478.00	120.00			NO	NO	NO				Mental Status Changes	> 181 Days	Scheduled Follow-up Submission	Progressive Disease	NO		41530.00	MALE	NO	NO	Astrocytoma
321	TCGA-HT-A4DS-01	0.00	TCGA-HT-A4DS-01	9.10	7.00	0.00	days	TCGA-HT-A4DS			days	7.00	days	TCGA Lower Grade Glioma (LGG)	brain lower grade glioma	Brain					55.00				TCGA-HT-A4DS-F61277	TCGA-HT-A4DS	TCGA-HT-A4DS-01A			(20296.00)	137.00		0.00	7.00				NO	YES	NO				Seizures	0 - 30 Days	Scheduled Follow-up Submission				41816.00	FEMALE		NO	Astrocytoma
322	TCGA-HT-A4DV-01	0.00	TCGA-HT-A4DV-01	10.34	792.00	0.00	days	TCGA-HT-A4DV	792.00	0.00	days	792.00	days	TCGA Lower Grade Glioma (LGG)	brain lower grade glioma	Brain					51.00				TCGA-HT-A4DV-F61274	TCGA-HT-A4DV	TCGA-HT-A4DV-01A			(18799.00)	141.00		0.00	792.00		14.00			YES	NO				Seizures	31 - 90 Days	Scheduled Follow-up Submission				41816.00	FEMALE		NO	Oligodendroglioma

323	TCGA-HT-A5R5-01	0.00	TCGA-HT-A5R5-01	11.69	4.00	0.00	days	TCGA-HT-A5R5			days	4.00	days	TCGA Lower Grade Glioma (LGG)	brain lower grade glioma	Brain					33.00				TCGA-HT-A5R5-F61085	TCGA-HT-A5R5	TCGA-HT-A5R5-01A			(12403.00)	194.00		0.00	4.00					NO	NO				Seizures	31 - 90 Days	Scheduled Follow-up Submission				41813.00	FEMALE			Oligodendroglioma
324	TCGA-HT-A5R7-01	0.00	TCGA-HT-A5R7-01	10.53	615.00	0.00	days	TCGA-HT-A5R7	615.00	0.00	days	615.00	days	TCGA Lower Grade Glioma (LGG)	brain lower grade glioma	Brain					33.00				TCGA-HT-A5R7-F61086	TCGA-HT-A5R7	TCGA-HT-A5R7-01A			(12092.00)	147.00		0.00	615.00					YES	NO				Seizures	0 - 30 Days	Scheduled Follow-up Submission	Complete Remission/Response			41813.00	FEMALE		NO	Astrocytoma
325	TCGA-HT-A5R9-01	0.00	TCGA-HT-A5R9-01	8.72	2.00	0.00	days	TCGA-HT-A5R9			days	2.00	days	TCGA Lower Grade Glioma (LGG)	brain lower grade glioma	Brain					48.00			NO	TCGA-HT-A5R9-F61080	TCGA-HT-A5R9	TCGA-HT-A5R9-01A			(17775.00)	79.00		0.00	2.00				NO	NO	NO				Seizures	0 - 30 Days	Scheduled Follow-up Submission				41816.00	FEMALE	NO	NO	Oligodendroglioma
326	TCGA-HT-A5RA-01	0.00	TCGA-HT-A5RA-01	12.04	832.00	0.00	days	TCGA-HT-A5RA	832.00	0.00	days	832.00	days	TCGA Lower Grade Glioma (LGG)	brain lower grade glioma	Brain					65.00	NO		NO	TCGA-HT-A5RA-F61263	TCGA-HT-A5RA	TCGA-HT-A5RA-01A			(23769.00)	385.00		0.00	832.00		244.00		NO	YES	NO				Seizures	31 - 90 Days	Scheduled Follow-up Submission	Partial Remission/Response	NO		41816.00	FEMALE	NO	NO	Astrocytoma
327	TCGA-HT-A5RB-01	0.00	TCGA-HT-A5RB-01	9.63	3.00	0.00	days	TCGA-HT-A5RB			days	3.00	days	TCGA Lower Grade Glioma (LGG)	brain lower grade glioma	Brain					24.00			NO	TCGA-HT-A5RB-F61266	TCGA-HT-A5RB	TCGA-HT-A5RB-01A			(8820.00)	597.00		0.00	3.00				NO	NO	NO				Seizures	0 - 30 Days	Scheduled Follow-up Submission				41816.00	MALE	NO	NO	Astrocytoma
328	TCGA-HT-A5RC-01	1.00	TCGA-HT-A5RC-01	11.67	162.00	1.00	days	TCGA-HT-A5RC	162.00	0.00	days	162.00	days	TCGA Lower Grade Glioma (LGG)	brain lower grade glioma	Brain					70.00					TCGA-HT-A5RC	TCGA-HT-A5RC-01A			(25772.00)	140.00	162.00	0.00						NO	NO				Mental Status Changes	0 - 30 Days					41484.00	FEMALE		NO	Astrocytoma
329	TCGA-HT-A614-01	0.00	TCGA-HT-A614-01	9.55	82.00	0.00	days	TCGA-HT-A614			days	82.00	days	TCGA Lower Grade Glioma (LGG)	brain lower grade glioma	Brain					47.00				TCGA-HT-A614-F61087	TCGA-HT-A614	TCGA-HT-A614-01A			(17392.00)	78.00		0.00	82.00										Headaches	> 181 Days	Scheduled Follow-up Submission	Partial Remission/Response			41813.00	MALE		YES	Oligoastrocytoma
330	TCGA-HT-A615-01	0.00	TCGA-HT-A615-01	10.53	515.00	0.00	days	TCGA-HT-A615	515.00	0.00	days	515.00	days	TCGA Lower Grade Glioma (LGG)	brain lower grade glioma	Brain					38.00			NO	TCGA-HT-A615-F66402	TCGA-HT-A615	TCGA-HT-A615-01A			(13892.00)	300.00		0.00	515.00		271.00		NO	YES	NO				Seizures	0 - 30 Days	Scheduled Follow-up Submission	Complete Remission/Response			41926.00	FEMALE		NO	Oligodendroglioma
331	TCGA-HT-A616-01	0.00	TCGA-HT-A616-01	8.60	55.00	0.00	days	TCGA-HT-A616			days	55.00	days	TCGA Lower Grade Glioma (LGG)	brain lower grade glioma	Brain					36.00	NO		NO	TCGA-HT-A616-F61270	TCGA-HT-A616	TCGA-HT-A616-01A			(13222.00)	70.00		0.00	55.00				NO	YES	NO				Seizures	31 - 90 Days	Scheduled Follow-up Submission				41816.00	FEMALE	NO	NO	Astrocytoma
332	TCGA-HT-A617-01	0.00	TCGA-HT-A617-01	10.71	491.00	0.00	days	TCGA-HT-A617			days	491.00	days	TCGA Lower Grade Glioma (LGG)	brain lower grade glioma	Brain					47.00			NO	TCGA-HT-A617-F61272	TCGA-HT-A617	TCGA-HT-A617-01A			(17331.00)	961.00		0.00	491.00		486.00		NO	NO	NO				Seizures	31 - 90 Days	Scheduled Follow-up Submission				41816.00	MALE	NO	NO	Oligodendroglioma
333	TCGA-HT-A618-01	0.00	TCGA-HT-A618-01	8.22	512.00	0.00	days	TCGA-HT-A618	512.00	0.00	days	512.00	days	TCGA Lower Grade Glioma (LGG)	brain lower grade glioma	Brain					37.00				TCGA-HT-A618-F61273	TCGA-HT-A618	TCGA-HT-A618-01A			(13595.00)	71.00		0.00	512.00		62.00			NO	NO				Mental Status Changes	0 - 30 Days	Scheduled Follow-up Submission	Complete Remission/Response			41816.00	FEMALE		NO	Astrocytoma
334	TCGA-HT-A619-01	0.00	TCGA-HT-A619-01	9.27	651.00	0.00	days	TCGA-HT-A619	651.00	0.00	days	651.00	days	TCGA Lower Grade Glioma (LGG)	brain lower grade glioma	Brain					51.00				TCGA-HT-A619-F66412	TCGA-HT-A619	TCGA-HT-A619-01A			(18857.00)	84.00		0.00	651.00										Motor/Movement Changes	0 - 30 Days	Scheduled Follow-up Submission	Complete Remission/Response			41926.00	FEMALE		NO	Oligodendroglioma
335	TCGA-HT-A61A-01	0.00	TCGA-HT-A61A-01	9.63	194.00	0.00	days	TCGA-HT-A61A			days	194.00	days	TCGA Lower Grade Glioma (LGG)	brain lower grade glioma	Brain					20.00					TCGA-HT-A61A	TCGA-HT-A61A-01A			(7664.00)	41.00		0.00	194.00						NO				Seizures	31 - 90 Days					41535.00	FEMALE		NO	Oligodendroglioma
336	TCGA-HT-A61B-01	0.00	TCGA-HT-A61B-01	8.46	533.00	0.00	days	TCGA-HT-A61B	533.00	0.00	days	533.00	days	TCGA Lower Grade Glioma (LGG)	brain lower grade glioma	Brain					22.00				TCGA-HT-A61B-F61305	TCGA-HT-A61B	TCGA-HT-A61B-01A			(8335.00)	101.00		0.00	533.00					NO	NO				Seizures	0 - 30 Days	Scheduled Follow-up Submission	Complete Remission/Response			41816.00	MALE		NO	Astrocytoma
337	TCGA-HT-A61C-01	1.00	TCGA-HT-A61C-01	11.18	537.00	1.00	days	TCGA-HT-A61C	476.00	1.00	days	537.00	days	TCGA Lower Grade Glioma (LGG)	brain lower grade glioma	Brain			YES		66.00	NO		NO	TCGA-HT-A61C-F61306	TCGA-HT-A61C	TCGA-HT-A61C-01A	501.00		(24276.00)	56.00	537.00	0.00	103.00	476.00	19.00		NO	YES					Motor/Movement Changes	0 - 30 Days	Scheduled Follow-up Submission	Progressive Disease	NO		41835.00	MALE	NO	NO	Oligodendroglioma
338	TCGA-HT-A74H-01	0.00	TCGA-HT-A74H-01	12.53	74.00	0.00	days	TCGA-HT-A74H			days	74.00	days	TCGA Lower Grade Glioma (LGG)	brain lower grade glioma	Brain					62.00	NO		NO	TCGA-HT-A74H-F65103	TCGA-HT-A74H	TCGA-HT-A74H-01A			(22850.00)	121.00		0.00	74.00				NO	NO	NO				Seizures	> 181 Days	Scheduled Follow-up Submission		NO		41900.00	MALE	NO	NO	Astrocytoma
339	TCGA-HT-A74J-01	0.00	TCGA-HT-A74J-01	8.28	320.00	0.00	days	TCGA-HT-A74J	320.00	0.00	days	320.00	days	TCGA Lower Grade Glioma (LGG)	brain lower grade glioma	Brain					33.00					TCGA-HT-A74J	TCGA-HT-A74J-01A			(12393.00)	91.00		0.00	320.00										Seizures	0 - 30 Days					41816.00	MALE		YES	Oligoastrocytoma
340	TCGA-HT-A74K-01	0.00	TCGA-HT-A74K-01	12.02	467.00	0.00	days	TCGA-HT-A74K	467.00	0.00	days	467.00	days	TCGA Lower Grade Glioma (LGG)	brain lower grade glioma	Brain					58.00					TCGA-HT-A74K	TCGA-HT-A74K-01A			(21189.00)	293.00		0.00	467.00										Seizures	> 181 Days					41816.00	FEMALE		NO	Oligodendroglioma
341	TCGA-HT-A74L-01	0.00	TCGA-HT-A74L-01	9.33	336.00	0.00	days	TCGA-HT-A74L	336.00	0.00	days	336.00	days	TCGA Lower Grade Glioma (LGG)	brain lower grade glioma	Brain					22.00	NO		NO	TCGA-HT-A74L-F65345	TCGA-HT-A74L	TCGA-HT-A74L-01A			(8240.00)	103.00		0.00	336.00				NO	YES	NO				Seizures	31 - 90 Days	Scheduled Follow-up Submission	Partial Remission/Response	NO		41904.00	FEMALE	NO	NO	Oligoastrocytoma
342	TCGA-HT-A74O-01	0.00	TCGA-HT-A74O-01	10.48	3.00	0.00	days	TCGA-HT-A74O			days	3.00	days	TCGA Lower Grade Glioma (LGG)	brain lower grade glioma	Brain					34.00	NO		NO	TCGA-HT-A74O-F63075	TCGA-HT-A74O	TCGA-HT-A74O-01A			(12460.00)	124.00		0.00	3.00				NO	NO	NO				Headaches	> 181 Days	Scheduled Follow-up Submission		NO		41850.00	MALE	NO	YES	Astrocytoma
343	TCGA-HW-7486-01	0.00	TCGA-HW-7486-01	11.19	1721.00	0.00	days	TCGA-HW-7486	1721.00	0.00	days	1721.00	days	TCGA Lower Grade Glioma (LGG)	brain lower grade glioma	Brain					37.00				TCGA-HW-7486-F71504	TCGA-HW-7486	TCGA-HW-7486-01A			(13546.00)			0.00	1721.00												Scheduled Follow-up Submission	Stable Disease			42094.00	MALE			Oligodendroglioma
344	TCGA-HW-7487-01	0.00	TCGA-HW-7487-01	9.34	1382.00	0.00	days	TCGA-HW-7487	1356.00	1.00	days	1382.00	days	TCGA Lower Grade Glioma (LGG)	brain lower grade glioma	Brain	YES	NO			39.00				TCGA-HW-7487-F63127	TCGA-HW-7487	TCGA-HW-7487-01A			(14535.00)			0.00	1382.00	1356.00											Additional New Tumor Event				41851.00	MALE			Oligodendroglioma
345	TCGA-HW-7489-01	1.00	TCGA-HW-7489-01	9.50	1262.00	1.00	days	TCGA-HW-7489	1262.00	0.00	days	1262.00	days	TCGA Lower Grade Glioma (LGG)	brain lower grade glioma	Brain					38.00				TCGA-HW-7489-F65183	TCGA-HW-7489	TCGA-HW-7489-01A			(14201.00)		1262.00	0.00	142.00												Scheduled Follow-up Submission				41901.00	MALE			Oligoastrocytoma
346	TCGA-HW-7490-01	0.00	TCGA-HW-7490-01	11.04	1387.00	0.00	days	TCGA-HW-7490	1387.00	0.00	days	1387.00	days	TCGA Lower Grade Glioma (LGG)	brain lower grade glioma	Brain					41.00				TCGA-HW-7490-F71515	TCGA-HW-7490	TCGA-HW-7490-01A			(15092.00)			0.00	1387.00												Scheduled Follow-up Submission	Progressive Disease			42094.00	MALE			Astrocytoma
347	TCGA-HW-7491-01	0.00	TCGA-HW-7491-01	11.15	2078.00	0.00	days	TCGA-HW-7491	2197.00	1.00	days	2078.00	days	TCGA Lower Grade Glioma (LGG)	brain lower grade glioma	Brain	NO	NO			35.00				TCGA-HW-7491-F71510	TCGA-HW-7491	TCGA-HW-7491-01A			(12874.00)			0.00	2078.00	2197.00											Scheduled Follow-up Submission				42094.00	MALE			Oligodendroglioma
348	TCGA-HW-7493-01	0.00	TCGA-HW-7493-01	9.93	2107.00	0.00	days	TCGA-HW-7493	2107.00	0.00	days	2107.00	days	TCGA Lower Grade Glioma (LGG)	brain lower grade glioma	Brain					40.00				TCGA-HW-7493-F71516	TCGA-HW-7493	TCGA-HW-7493-01A			(14767.00)			0.00	2107.00												Scheduled Follow-up Submission	Stable Disease			42094.00	FEMALE			Astrocytoma
349	TCGA-HW-7495-01	0.00	TCGA-HW-7495-01	9.55	1078.00	0.00	days	TCGA-HW-7495	1078.00	0.00	days	1078.00	days	TCGA Lower Grade Glioma (LGG)	brain lower grade glioma	Brain					45.00				TCGA-HW-7495-F66931	TCGA-HW-7495	TCGA-HW-7495-01A			(16460.00)			0.00	1078.00												Scheduled Follow-up Submission	Progressive Disease			41940.00	FEMALE			Oligodendroglioma
350	TCGA-HW-8319-01	1.00	TCGA-HW-8319-01	8.77	1209.00	1.00	days	TCGA-HW-8319	997.00	1.00	days	1209.00	days	TCGA Lower Grade Glioma (LGG)	brain lower grade glioma	Brain	YES	NO			34.00				TCGA-HW-8319-F71526	TCGA-HW-8319	TCGA-HW-8319-01A			(12557.00)		1209.00	0.00	262.00	997.00											Scheduled Follow-up Submission				42094.00	FEMALE			Astrocytoma
351	TCGA-HW-8320-01	0.00	TCGA-HW-8320-01	8.89	1217.00	0.00	days	TCGA-HW-8320	1217.00	0.00	days	1217.00	days	TCGA Lower Grade Glioma (LGG)	brain lower grade glioma	Brain					36.00				TCGA-HW-8320-F71522	TCGA-HW-8320	TCGA-HW-8320-01A			(13503.00)			0.00	1217.00												Scheduled Follow-up Submission	Stable Disease			42094.00	MALE			Astrocytoma
352	TCGA-HW-8321-01	0.00	TCGA-HW-8321-01	10.51	1294.00	0.00	days	TCGA-HW-8321	1294.00	0.00	days	1294.00	days	TCGA Lower Grade Glioma (LGG)	brain lower grade glioma	Brain					31.00				TCGA-HW-8321-F71520	TCGA-HW-8321	TCGA-HW-8321-01A			(11474.00)			0.00	1294.00												Scheduled Follow-up Submission	Stable Disease			42094.00	MALE			Astrocytoma
353	TCGA-HW-8322-01	0.00	TCGA-HW-8322-01	8.70	758.00	0.00	days	TCGA-HW-8322	758.00	0.00	days	758.00	days	TCGA Lower Grade Glioma (LGG)	brain lower grade glioma	Brain					39.00				TCGA-HW-8322-F71499	TCGA-HW-8322	TCGA-HW-8322-01A			(14548.00)			0.00	758.00												Scheduled Follow-up Submission	Progressive Disease			42094.00	MALE			Oligodendroglioma
354	TCGA-HW-A5KJ-01	1.00	TCGA-HW-A5KJ-01	8.26	962.00	1.00	days	TCGA-HW-A5KJ	962.00	0.00	days	962.00	days	TCGA Lower Grade Glioma (LGG)	brain lower grade glioma	Brain					68.00				TCGA-HW-A5KJ-F71524	TCGA-HW-A5KJ	TCGA-HW-A5KJ-01A			(24966.00)	286.00	962.00	0.00	321.00												Scheduled Follow-up Submission	Stable Disease			42094.00	MALE			Oligodendroglioma
355	TCGA-HW-A5KK-01	1.00	TCGA-HW-A5KK-01	10.59	388.00	1.00	days	TCGA-HW-A5KK	388.00	0.00	days	388.00	days	TCGA Lower Grade Glioma (LGG)	brain lower grade glioma	Brain					64.00				TCGA-HW-A5KK-F59869	TCGA-HW-A5KK	TCGA-HW-A5KK-01A			(23484.00)	171.00	388.00	0.00	240.00												Scheduled Follow-up Submission				41781.00	MALE			Astrocytoma
356	TCGA-HW-A5KL-01	0.00	TCGA-HW-A5KL-01	9.61	878.00	0.00	days	TCGA-HW-A5KL	878.00	0.00	days	878.00	days	TCGA Lower Grade Glioma (LGG)	brain lower grade glioma	Brain					42.00				TCGA-HW-A5KL-F71508	TCGA-HW-A5KL				(15513.00)			0.00	878.00												Scheduled Follow-up Submission	Stable Disease			42094.00	FEMALE			Astrocytoma
357	TCGA-HW-A5KM-01	0.00	TCGA-HW-A5KM-01	8.95	630.00	0.00	days	TCGA-HW-A5KM	630.00	0.00	days	630.00	days	TCGA Lower Grade Glioma (LGG)	brain lower grade glioma	Brain					35.00				TCGA-HW-A5KM-F59872	TCGA-HW-A5KM	TCGA-HW-A5KM-01A			(12845.00)	139.00		0.00	630.00												Scheduled Follow-up Submission	Progressive Disease			41781.00	MALE			Astrocytoma
358	TCGA-IK-7675-01	1.00	TCGA-IK-7675-01	10.10	578.00	1.00	days	TCGA-IK-7675	123.00	1.00	days	578.00	days	TCGA Lower Grade Glioma (LGG)	brain lower grade glioma	Brain	YES				43.00			NO	TCGA-IK-7675-F33064	TCGA-IK-7675	TCGA-IK-7675-01A			(15900.00)		578.00	0.00		123.00	48.00			NO	NO				Motor/Movement Changes	0 - 30 Days	Scheduled Follow-up Submission	Progressive Disease			41079.00	MALE		YES	Oligodendroglioma

359	TCGA-IK-8125-01	0.00	TCGA-IK-8125-01	9.73	1301.00	0.00	days	TCGA-IK-8125	1301.00	0.00	days	1301.00	days	TCGA Lower Grade Glioma (LGG)	brain lower grade glioma	Brain					62.00			NO	TCGA-IK-8125-F40395	TCGA-IK-8125	TCGA-IK-8125-01A			(22745.00)			0.00	1301.00		42.00	0.00	YES	YES	NO	> 20 Years			Seizures	0 - 30 Days	Scheduled Follow-up Submission	Complete Remission/Response	NO		41323.00	MALE		NO	Oligoastrocytoma
360	TCGA-KT-A74X-01	0.00	TCGA-KT-A74X-01	11.21	438.00	0.00	days	TCGA-KT-A74X	438.00	0.00	days	438.00	days	TCGA Lower Grade Glioma (LGG)	brain lower grade glioma	Brain					26.00	YES	Cat Dander		TCGA-KT-A74X-F69145	TCGA-KT-A74X	TCGA-KT-A74X-01A			(9590.00)	21.00		0.00	438.00					NO	NO				Seizures		Scheduled Follow-up Submission	Stable Disease			42003.00	MALE	NO	YES	Oligoastrocytoma
361	TCGA-KT-A7W1-01	0.00	TCGA-KT-A7W1-01	10.32	438.00	0.00	days	TCGA-KT-A7W1	438.00	0.00	days	438.00	days	TCGA Lower Grade Glioma (LGG)	brain lower grade glioma	Brain					45.00				TCGA-KT-A7W1-F69146	TCGA-KT-A7W1				(16560.00)			0.00	438.00		10.00			YES	NO				Seizures	0 - 30 Days	Scheduled Follow-up Submission	Stable Disease			42003.00	FEMALE			Astrocytoma
362	TCGA-P5-A5EV-01	0.00	TCGA-P5-A5EV-01	9.85	7.00	0.00	days	TCGA-P5-A5EV			days	7.00	days	TCGA Lower Grade Glioma (LGG)	brain lower grade glioma	Brain					39.00				TCGA-P5-A5EV-F69040	TCGA-P5-A5EV	TCGA-P5-A5EV-01A			(14310.00)	86.00		0.00	7.00										Seizures	31 - 90 Days	Scheduled Follow-up Submission				41997.00	MALE		YES	Astrocytoma
363	TCGA-P5-A5EW-01	0.00	TCGA-P5-A5EW-01	9.40	204.00	0.00	days	TCGA-P5-A5EW	204.00	0.00	days	204.00	days	TCGA Lower Grade Glioma (LGG)	brain lower grade glioma	Brain					20.00	YES	cats		TCGA-P5-A5EW-F69041	TCGA-P5-A5EW	TCGA-P5-A5EW-01A			(7612.00)	77.00		0.00	204.00		(2.00)						< 12 Years		Headaches	91 - 180 Days	Scheduled Follow-up Submission		NO		41997.00	FEMALE	NO	YES	Astrocytoma
364	TCGA-P5-A5EX-01	0.00	TCGA-P5-A5EX-01	11.49	243.00	0.00	days	TCGA-P5-A5EX	243.00	0.00	days	243.00	days	TCGA Lower Grade Glioma (LGG)	brain lower grade glioma	Brain					41.00			NO	TCGA-P5-A5EX-F69042	TCGA-P5-A5EX	TCGA-P5-A5EX-01A			(15091.00)	65.00		0.00	243.00				NO						Headaches	> 181 Days	Scheduled Follow-up Submission				41997.00	FEMALE	NO	YES	Oligodendroglioma
365	TCGA-P5-A5EY-01	0.00	TCGA-P5-A5EY-01	10.09	72.00	0.00	days	TCGA-P5-A5EY			days	72.00	days	TCGA Lower Grade Glioma (LGG)	brain lower grade glioma	Brain					21.00				TCGA-P5-A5EY-F69043	TCGA-P5-A5EY	TCGA-P5-A5EY-01A			(7716.00)	65.00		0.00	72.00										Seizures	0 - 30 Days	Scheduled Follow-up Submission				41997.00	MALE			Astrocytoma
366	TCGA-P5-A5EZ-01	0.00	TCGA-P5-A5EZ-01	10.67	70.00	0.00	days	TCGA-P5-A5EZ	70.00	0.00	days	70.00	days	TCGA Lower Grade Glioma (LGG)	brain lower grade glioma	Brain					39.00	YES	bee		TCGA-P5-A5EZ-F69044	TCGA-P5-A5EZ	TCGA-P5-A5EZ-01A			(14375.00)	65.00		0.00	70.00								> 20 Years		Seizures	31 - 90 Days	Scheduled Follow-up Submission				41997.00	MALE		YES	Astrocytoma
367	TCGA-P5-A5F0-01	0.00	TCGA-P5-A5F0-01	9.69	287.00	0.00	days	TCGA-P5-A5F0	287.00	0.00	days	287.00	days	TCGA Lower Grade Glioma (LGG)	brain lower grade glioma	Brain					33.00			NO	TCGA-P5-A5F0-F69045	TCGA-P5-A5F0	TCGA-P5-A5F0-01A			(12157.00)	62.00		0.00	287.00				NO						Seizures	> 181 Days	Scheduled Follow-up Submission				41997.00	MALE	NO	YES	Oligodendroglioma
368	TCGA-P5-A5F1-01	0.00	TCGA-P5-A5F1-01	11.48	179.00	0.00	days	TCGA-P5-A5F1	179.00	0.00	days	179.00	days	TCGA Lower Grade Glioma (LGG)	brain lower grade glioma	Brain					30.00				TCGA-P5-A5F1-F69046	TCGA-P5-A5F1	TCGA-P5-A5F1-01A			(11070.00)	55.00		0.00	179.00										Motor/Movement Changes	31 - 90 Days	Scheduled Follow-up Submission				41997.00	MALE			Astrocytoma
369	TCGA-P5-A5F2-01	0.00	TCGA-P5-A5F2-01	8.98	242.00	0.00	days	TCGA-P5-A5F2	242.00	0.00	days	242.00	days	TCGA Lower Grade Glioma (LGG)	brain lower grade glioma	Brain					36.00				TCGA-P5-A5F2-F69047	TCGA-P5-A5F2	TCGA-P5-A5F2-01A			(13396.00)	48.00		0.00	242.00										Seizures	91 - 180 Days	Scheduled Follow-up Submission				41997.00	FEMALE		YES	Astrocytoma
370	TCGA-P5-A5F4-01	0.00	TCGA-P5-A5F4-01	9.62	231.00	0.00	days	TCGA-P5-A5F4			days	231.00	days	TCGA Lower Grade Glioma (LGG)	brain lower grade glioma	Brain					35.00				TCGA-P5-A5F4-F69048	TCGA-P5-A5F4	TCGA-P5-A5F4-01A			(12851.00)	37.00		0.00	231.00										Headaches	31 - 90 Days	Scheduled Follow-up Submission				41997.00	FEMALE		YES	Oligodendroglioma
371	TCGA-P5-A5F6-01	0.00	TCGA-P5-A5F6-01	5.12	39.00	0.00	days	TCGA-P5-A5F6	39.00	0.00	days	39.00	days	TCGA Lower Grade Glioma (LGG)	brain lower grade glioma	Brain					26.00				TCGA-P5-A5F6-F69049	TCGA-P5-A5F6	TCGA-P5-A5F6-01A			(9575.00)	35.00		0.00	39.00										Headaches		Scheduled Follow-up Submission				41997.00	MALE		YES	Oligodendroglioma
372	TCGA-P5-A72U-01	0.00	TCGA-P5-A72U-01	11.49	7.00	0.00	days	TCGA-P5-A72U	7.00	0.00	days	7.00	days	TCGA Lower Grade Glioma (LGG)	brain lower grade glioma	Brain					71.00				TCGA-P5-A72U-F69015	TCGA-P5-A72U	TCGA-P5-A72U-01A			(26002.00)	196.00		0.00	7.00												Additional New Tumor Event				41997.00	FEMALE			Oligodendroglioma
373	TCGA-P5-A72W-01	0.00	TCGA-P5-A72W-01	9.88	317.00	0.00	days	TCGA-P5-A72W	317.00	0.00	days	317.00	days	TCGA Lower Grade Glioma (LGG)	brain lower grade glioma	Brain					35.00				TCGA-P5-A72W-F69016	TCGA-P5-A72W	TCGA-P5-A72W-01A			(12848.00)	185.00		0.00	317.00												Scheduled Follow-up Submission				41997.00	MALE			Astrocytoma
374	TCGA-P5-A72X-01	0.00	TCGA-P5-A72X-01	11.28	403.00	0.00	days	TCGA-P5-A72X			days	403.00	days	TCGA Lower Grade Glioma (LGG)	brain lower grade glioma	Brain					21.00				TCGA-P5-A72X-F69018	TCGA-P5-A72X	TCGA-P5-A72X-01A			(7913.00)	178.00		0.00	403.00												Scheduled Follow-up Submission				41997.00	MALE			Astrocytoma
375	TCGA-P5-A72Z-01	0.00	TCGA-P5-A72Z-01	9.98	354.00	0.00	days	TCGA-P5-A72Z			days	354.00	days	TCGA Lower Grade Glioma (LGG)	brain lower grade glioma	Brain					64.00				TCGA-P5-A72Z-F69020	TCGA-P5-A72Z	TCGA-P5-A72Z-01A			(23482.00)	178.00		0.00	354.00												Scheduled Follow-up Submission				41997.00	FEMALE			Oligodendroglioma
376	TCGA-P5-A730-01	0.00	TCGA-P5-A730-01	10.03	333.00	0.00	days	TCGA-P5-A730	333.00	0.00	days	333.00	days	TCGA Lower Grade Glioma (LGG)	brain lower grade glioma	Brain					22.00				TCGA-P5-A730-F69023	TCGA-P5-A730	TCGA-P5-A730-01A			(8043.00)	161.00		0.00	333.00												Scheduled Follow-up Submission				41997.00	MALE			Oligoastrocytoma
377	TCGA-P5-A731-01	0.00	TCGA-P5-A731-01	11.23	274.00	0.00	days	TCGA-P5-A731			days	274.00	days	TCGA Lower Grade Glioma (LGG)	brain lower grade glioma	Brain					59.00				TCGA-P5-A731-F69010	TCGA-P5-A731	TCGA-P5-A731-01A			(21715.00)	143.00		0.00	274.00												Scheduled Follow-up Submission				41997.00	FEMALE		YES	Oligoastrocytoma
378	TCGA-P5-A733-01	0.00	TCGA-P5-A733-01	10.37	414.00	0.00	days	TCGA-P5-A733			days	414.00	days	TCGA Lower Grade Glioma (LGG)	brain lower grade glioma	Brain					52.00				TCGA-P5-A733-F69012	TCGA-P5-A733	TCGA-P5-A733-01A			(19142.00)	134.00		0.00	414.00												Scheduled Follow-up Submission				41997.00	FEMALE			Astrocytoma
379	TCGA-P5-A735-01	0.00	TCGA-P5-A735-01	9.13	292.00	0.00	days	TCGA-P5-A735			days	292.00	days	TCGA Lower Grade Glioma (LGG)	brain lower grade glioma	Brain					38.00				TCGA-P5-A735-F69024	TCGA-P5-A735	TCGA-P5-A735-01A			(14152.00)	134.00		0.00	292.00												Scheduled Follow-up Submission	Complete Remission/Response			41997.00	FEMALE			Astrocytoma
380	TCGA-P5-A736-01	0.00	TCGA-P5-A736-01	11.45	230.00	0.00	days	TCGA-P5-A736			days	230.00	days	TCGA Lower Grade Glioma (LGG)	brain lower grade glioma	Brain					44.00				TCGA-P5-A736-F69025	TCGA-P5-A736	TCGA-P5-A736-01A			(16300.00)	126.00		0.00	230.00												Scheduled Follow-up Submission	Complete Remission/Response			41997.00	FEMALE			Astrocytoma
381	TCGA-P5-A737-01	0.00	TCGA-P5-A737-01	9.98	372.00	0.00	days	TCGA-P5-A737			days	372.00	days	TCGA Lower Grade Glioma (LGG)	brain lower grade glioma	Brain					47.00				TCGA-P5-A737-F69027	TCGA-P5-A737	TCGA-P5-A737-01A			(17390.00)	217.00		0.00	372.00												Scheduled Follow-up Submission				41997.00	MALE			Oligoastrocytoma
382	TCGA-P5-A77W-01	0.00	TCGA-P5-A77W-01	9.04	584.00	0.00	days	TCGA-P5-A77W			days	584.00	days	TCGA Lower Grade Glioma (LGG)	brain lower grade glioma	Brain					37.00				TCGA-P5-A77W-F69028	TCGA-P5-A77W	TCGA-P5-A77W-01A			(13545.00)	114.00		0.00	584.00												Scheduled Follow-up Submission				41997.00	FEMALE			Oligoastrocytoma
383	TCGA-P5-A77X-01	0.00	TCGA-P5-A77X-01	9.37	105.00	0.00	days	TCGA-P5-A77X			days	105.00	days	TCGA Lower Grade Glioma (LGG)	brain lower grade glioma	Brain					55.00				TCGA-P5-A77X-F69029	TCGA-P5-A77X	TCGA-P5-A77X-01A			(20128.00)	111.00		0.00	105.00												Scheduled Follow-up Submission	Complete Remission/Response			41997.00	FEMALE			Oligoastrocytoma
384	TCGA-P5-A780-01	0.00	TCGA-P5-A780-01	11.01	72.00	0.00	days	TCGA-P5-A780			days	72.00	days	TCGA Lower Grade Glioma (LGG)	brain lower grade glioma	Brain					44.00				TCGA-P5-A780-F69031	TCGA-P5-A780	TCGA-P5-A780-01A			(16161.00)	92.00		0.00	72.00												Scheduled Follow-up Submission	Complete Remission/Response			41997.00	FEMALE			Astrocytoma
385	TCGA-P5-A781-01	0.00	TCGA-P5-A781-01	10.01	134.00	0.00	days	TCGA-P5-A781			days	134.00	days	TCGA Lower Grade Glioma (LGG)	brain lower grade glioma	Brain					34.00				TCGA-P5-A781-F69033	TCGA-P5-A781	TCGA-P5-A781-01A			(12757.00)	86.00		0.00	134.00												Scheduled Follow-up Submission	Complete Remission/Response			41997.00	FEMALE			Astrocytoma
386	TCGA-QH-A65R-01	0.00	TCGA-QH-A65R-01	9.07	461.00	0.00	days	TCGA-QH-A65R	461.00	0.00	days	461.00	days	TCGA Lower Grade Glioma (LGG)	brain lower grade glioma	Brain					38.00	YES	CAT	NO	TCGA-QH-A65R-F59571	TCGA-QH-A65R				(14073.00)			0.00	461.00		(10.00)	0.00	NO	YES	NO	< 12 Years	> 20 Years				Scheduled Follow-up Submission	Stable Disease	NO		41800.00	FEMALE	YES	NO	Oligodendroglioma
387	TCGA-QH-A65S-01	0.00	TCGA-QH-A65S-01	10.86	77.00	0.00	days	TCGA-QH-A65S	77.00	0.00	days	77.00	days	TCGA Lower Grade Glioma (LGG)	brain lower grade glioma	Brain	NO	NO			32.00	NO		NO	TCGA-QH-A65S-F42106	TCGA-QH-A65S	TCGA-QH-A65S-01A			(11861.00)	56.00		0.00	77.00		(7.00)	1.00	NO	YES	NO				Seizures	0 - 30 Days	Scheduled Follow-up Submission	Partial Remission/Response	NO		41957.00	FEMALE	NO	NO	Oligoastrocytoma
388	TCGA-QH-A65V-01	0.00	TCGA-QH-A65V-01	10.58	384.00	0.00	days	TCGA-QH-A65V	384.00	0.00	days	384.00	days	TCGA Lower Grade Glioma (LGG)	brain lower grade glioma	Brain	NO	NO	NO		43.00	NO		NO	TCGA-QH-A65V-F59561	TCGA-QH-A65V	TCGA-QH-A65V-01A			(15850.00)	48.00		0.00	384.00		(9.00)	1.00	NO	NO	NO				Seizures	0 - 30 Days	Scheduled Follow-up Submission		NO		41774.00	FEMALE	NO	NO	Oligodendroglioma
389	TCGA-QH-A65X-01	0.00	TCGA-QH-A65X-01	7.90	482.00	0.00	days	TCGA-QH-A65X	482.00	0.00	days	482.00	days	TCGA Lower Grade Glioma (LGG)	brain lower grade glioma	Brain					28.00	NO		NO	TCGA-QH-A65X-F67754	TCGA-QH-A65X	TCGA-QH-A65X-01A			(10440.00)	39.00		0.00	482.00		237.00	1.00	NO	NO	NO				Seizures	0 - 30 Days	Scheduled Follow-up Submission	Stable Disease	NO		41954.00	FEMALE	NO	NO	Oligoastrocytoma
390	TCGA-QH-A65Z-01	0.00	TCGA-QH-A65Z-01	9.83	427.00	0.00	days	TCGA-QH-A65Z	427.00	0.00	days	427.00	days	TCGA Lower Grade Glioma (LGG)	brain lower grade glioma	Brain	NO	NO			54.00	NO		NO	TCGA-QH-A65Z-F59568	TCGA-QH-A65Z	TCGA-QH-A65Z-01A			(19921.00)	19.00		0.00	427.00		(10.00)	1.00	NO	YES	NO				Seizures	0 - 30 Days	Scheduled Follow-up Submission		NO		41800.00	MALE	NO	NO	Oligodendroglioma
391	TCGA-QH-A6CS-01	0.00	TCGA-QH-A6CS-01	10.40	588.00	0.00	days	TCGA-QH-A6CS	498.00	1.00	days	588.00	days	TCGA Lower Grade Glioma (LGG)	brain lower grade glioma	Brain	YES	NO	NO		41.00	NO		NO	TCGA-QH-A6CS-F67755	TCGA-QH-A6CS	TCGA-QH-A6CS-01A			(15092.00)	53.00		0.00	588.00	498.00	(25.00)	1.00	NO	YES	NO				Seizures	0 - 30 Days	Scheduled Follow-up Submission		NO		41956.00	MALE	NO	YES	Astrocytoma
392	TCGA-QH-A6CU-01	0.00	TCGA-QH-A6CU-01	10.59	451.00	0.00	days	TCGA-QH-A6CU	451.00	0.00	days	451.00	days	TCGA Lower Grade Glioma (LGG)	brain lower grade glioma	Brain	NO	NO	NO		52.00	NO		NO	TCGA-QH-A6CU-F60341	TCGA-QH-A6CU	TCGA-QH-A6CU-01A			(19149.00)	59.00		0.00	451.00		(13.00)	1.00	NO	YES	NO	> 20 Years			Seizures	0 - 30 Days	Scheduled Follow-up Submission	Stable Disease	NO		41796.00	FEMALE	YES	NO	Oligodendroglioma
393	TCGA-QH-A6CV-01	0.00	TCGA-QH-A6CV-01	10.21	442.00	0.00	days	TCGA-QH-A6CV	442.00	0.00	days	442.00	days	TCGA Lower Grade Glioma (LGG)	brain lower grade glioma	Brain	NO	NO	NO		51.00	NO		NO	TCGA-QH-A6CV-F60218	TCGA-QH-A6CV	TCGA-QH-A6CV-01A			(18955.00)	52.00		0.00	442.00		(42.00)	1.00	NO	NO	NO				Headaches	0 - 30 Days	Scheduled Follow-up Submission	Stable Disease	NO		41796.00	MALE	NO	YES	Oligoastrocytoma
394	TCGA-QH-A6CW-01	0.00	TCGA-QH-A6CW-01	9.20	414.00	0.00	days	TCGA-QH-A6CW	414.00	0.00	days	414.00	days	TCGA Lower Grade Glioma (LGG)	brain lower grade glioma	Brain	NO	NO	NO		43.00	NO		YES	TCGA-QH-A6CW-F60337	TCGA-QH-A6CW	TCGA-QH-A6CW-01A			(15847.00)	24.00		0.00	414.00		(8.00)	1.00	NO	NO	NO	> 20 Years			Seizures	31 - 90 Days	Scheduled Follow-up Submission	Partial Remission/Response	NO		41796.00	MALE	YES	NO	Oligoastrocytoma

395	TCGA-QH-A6CX-01	1.00	TCGA-QH-A6CX-01	13.67	372.00	1.00	days	TCGA-QH-A6CX	273.00	1.00	days	372.00	days	TCGA Lower Grade Glioma (LGG)	brain lower grade glioma	Brain	NO	NO	NO		66.00	NO		NO	TCGA-QH-A6CX-F60335	TCGA-QH-A6CX	TCGA-QH-A6CX-01A			(24252.00)	23.00	372.00	0.00	(1.00)	273.00	(7.00)	1.00	NO	NO	NO				Seizures	0 - 30 Days	Scheduled Follow-up Submission		NO		41796.00	MALE	NO	NO	Astrocytoma
396	TCGA-QH-A6CY-01	0.00	TCGA-QH-A6CY-01	9.87	408.00	0.00	days	TCGA-QH-A6CY	408.00	0.00	days	408.00	days	TCGA Lower Grade Glioma (LGG)	brain lower grade glioma	Brain					38.00	NO		NO	TCGA-QH-A6CY-F60215	TCGA-QH-A6CY	TCGA-QH-A6CY-01A			(14140.00)	18.00		0.00	408.00		(26.00)	1.00	NO	NO	NO				Headaches	0 - 30 Days	Scheduled Follow-up Submission	Stable Disease	NO		41796.00	MALE	NO	YES	Oligoastrocytoma
397	TCGA-QH-A6CZ-01	0.00	TCGA-QH-A6CZ-01	9.94	279.00	0.00	days	TCGA-QH-A6CZ	279.00	0.00	days	279.00	days	TCGA Lower Grade Glioma (LGG)	brain lower grade glioma	Brain	NO	NO	NO		38.00	NO		NO	TCGA-QH-A6CZ-F60216	TCGA-QH-A6CZ	TCGA-QH-A6CZ-01A			(14007.00)	16.00		0.00	279.00		(51.00)	1.00	NO	NO	NO				Seizures	0 - 30 Days	Scheduled Follow-up Submission	Stable Disease	NO		41793.00	MALE	NO	NO	Oligoastrocytoma
398	TCGA-QH-A6X3-01	0.00	TCGA-QH-A6X3-01	10.89	313.00	0.00	days	TCGA-QH-A6X3	313.00	0.00	days	313.00	days	TCGA Lower Grade Glioma (LGG)	brain lower grade glioma	Brain					27.00			NO	TCGA-QH-A6X3-F67748	TCGA-QH-A6X3				(10184.00)			0.00	313.00		16.00	1.00	NO	NO	NO				Seizures	0 - 30 Days	Scheduled Follow-up Submission	Complete Remission/Response			41954.00	MALE	NO	NO	Oligoastrocytoma
399	TCGA-QH-A6X4-01	0.00	TCGA-QH-A6X4-01	9.15	442.00	0.00	days	TCGA-QH-A6X4	442.00	0.00	days	442.00	days	TCGA Lower Grade Glioma (LGG)	brain lower grade glioma	Brain					47.00	NO		NO	TCGA-QH-A6X4-F67744	TCGA-QH-A6X4	TCGA-QH-A6X4-01A			(17258.00)	129.00		0.00	442.00		192.00	1.00	NO	NO	NO				Sensory Changes	0 - 30 Days	Scheduled Follow-up Submission	Complete Remission/Response			41954.00	MALE	NO	NO	Oligoastrocytoma
400	TCGA-QH-A6X5-01	0.00	TCGA-QH-A6X5-01	10.15	497.00	0.00	days	TCGA-QH-A6X5	497.00	0.00	days	497.00	days	TCGA Lower Grade Glioma (LGG)	brain lower grade glioma	Brain					58.00	NO		NO	TCGA-QH-A6X5-F67745	TCGA-QH-A6X5	TCGA-QH-A6X5-01A			(21239.00)	65.00		0.00	497.00		119.00	1.00	NO	NO	NO				Seizures	0 - 30 Days	Scheduled Follow-up Submission				41954.00	FEMALE	NO	NO	Oligoastrocytoma
401	TCGA-QH-A6X8-01	0.00	TCGA-QH-A6X8-01	10.27	509.00	0.00	days	TCGA-QH-A6X8	509.00	0.00	days	509.00	days	TCGA Lower Grade Glioma (LGG)	brain lower grade glioma	Brain					56.00	NO		NO	TCGA-QH-A6X8-F67753	TCGA-QH-A6X8	TCGA-QH-A6X8-01A			(20771.00)	54.00		0.00	509.00		144.00	0.00	NO	NO	NO				Mental Status Changes	0 - 30 Days	Scheduled Follow-up Submission	Stable Disease			41954.00	FEMALE	NO	NO	Oligodendroglioma
402	TCGA-QH-A6X9-01	0.00	TCGA-QH-A6X9-01	9.43	58.00	0.00	days	TCGA-QH-A6X9	58.00	0.00	days	58.00	days	TCGA Lower Grade Glioma (LGG)	brain lower grade glioma	Brain					73.00	NO		NO	TCGA-QH-A6X9-F67996	TCGA-QH-A6X9	TCGA-QH-A6X9-01A			(26992.00)	22.00		0.00	58.00		58.00	3.00	NO	YES	NO				Seizures	0 - 30 Days	Scheduled Follow-up Submission		NO		41956.00	FEMALE	NO	NO	Oligodendroglioma
403	TCGA-QH-A6XA-01	0.00	TCGA-QH-A6XA-01	9.61	502.00	0.00	days	TCGA-QH-A6XA	502.00	0.00	days	502.00	days	TCGA Lower Grade Glioma (LGG)	brain lower grade glioma	Brain					23.00	NO		NO	TCGA-QH-A6XA-F67837	TCGA-QH-A6XA	TCGA-QH-A6XA-01A			(8508.00)	19.00		0.00	502.00		7.00	0.00	NO	NO	NO						Scheduled Follow-up Submission	Complete Remission/Response	NO		41955.00	FEMALE	NO	NO	Oligoastrocytoma
404	TCGA-QH-A6XC-01	0.00	TCGA-QH-A6XC-01	10.13	508.00	0.00	days	TCGA-QH-A6XC	438.00	1.00	days	508.00	days	TCGA Lower Grade Glioma (LGG)	brain lower grade glioma	Brain	YES	YES	YES		48.00			YES	TCGA-QH-A6XC-F67756	TCGA-QH-A6XC	TCGA-QH-A6XC-01A	440.00		(17692.00)	27.00		0.00	508.00	438.00	153.00	1.00	NO	YES	NO				Seizures	0 - 30 Days	Scheduled Follow-up Submission		NO		41955.00	MALE	NO	NO	Astrocytoma
405	TCGA-QH-A86X-01	0.00	TCGA-QH-A86X-01	9.35	337.00	0.00	days	TCGA-QH-A86X	337.00	0.00	days	337.00	days	TCGA Lower Grade Glioma (LGG)	brain lower grade glioma	Brain					33.00	NO		NO	TCGA-QH-A86X-F67829	TCGA-QH-A86X	TCGA-QH-A86X-01A			(12385.00)	7.00		0.00	337.00		106.00	0.00	NO	YES					Seizures	91 - 180 Days	Scheduled Follow-up Submission	Complete Remission/Response	NO		41955.00	MALE	NO	NO	Oligodendroglioma
406	TCGA-QH-A870-01	0.00	TCGA-QH-A870-01	10.13	395.00	0.00	days	TCGA-QH-A870	335.00	1.00	days	395.00	days	TCGA Lower Grade Glioma (LGG)	brain lower grade glioma	Brain	YES	NO	NO		38.00	NO		NO	TCGA-QH-A870-F67994	TCGA-QH-A870	TCGA-QH-A870-01A			(14082.00)	1.00		0.00	395.00	335.00	2.00	1.00	NO	YES	NO				Headaches	> 181 Days	Scheduled Follow-up Submission	Progressive Disease			41957.00	FEMALE	NO	YES	Oligoastrocytoma
407	TCGA-R8-A6MK-01	0.00	TCGA-R8-A6MK-01	10.32	2702.00	0.00	days	TCGA-R8-A6MK			days	2702.00	days	TCGA Lower Grade Glioma (LGG)	brain lower grade glioma	Brain					40.00					TCGA-R8-A6MK	TCGA-R8-A6MK-01A			(14716.00)	2841.00		0.00	2702.00					YES	NO										41638.00	MALE			Oligodendroglioma
408	TCGA-R8-A6ML-01	0.00	TCGA-R8-A6ML-01	9.64	2860.00	0.00	days	TCGA-R8-A6ML			days	2860.00	days	TCGA Lower Grade Glioma (LGG)	brain lower grade glioma	Brain					52.00					TCGA-R8-A6ML	TCGA-R8-A6ML-01A			(19185.00)	2735.00		0.00	2860.00					NO	NO										41638.00	MALE			Oligodendroglioma
409	TCGA-R8-A6MO-01	0.00	TCGA-R8-A6MO-01	9.71	993.00	0.00	days	TCGA-R8-A6MO			days	993.00	days	TCGA Lower Grade Glioma (LGG)	brain lower grade glioma	Brain					53.00					TCGA-R8-A6MO	TCGA-R8-A6MO-01A			(19432.00)	1519.00		0.00	993.00					NO	NO										41638.00	FEMALE			Oligodendroglioma
410	TCGA-R8-A73M-01	0.00	TCGA-R8-A73M-01	8.89	1806.00	0.00	days	TCGA-R8-A73M	1806.00	0.00	days	1806.00	days	TCGA Lower Grade Glioma (LGG)	brain lower grade glioma	Brain					48.00					TCGA-R8-A73M				(17610.00)			0.00	1806.00					YES	NO										41638.00	FEMALE		YES	Oligodendroglioma
411	TCGA-RY-A83X-01	0.00	TCGA-RY-A83X-01	9.90	939.00	0.00	days	TCGA-RY-A83X	939.00	0.00	days	939.00	days	TCGA Lower Grade Glioma (LGG)	brain lower grade glioma	Brain					46.00			NO	TCGA-RY-A83X-F69371	TCGA-RY-A83X	TCGA-RY-A83X-01A			(16911.00)	486.00		0.00	939.00				NO	NO	NO				Mental Status Changes	> 181 Days	Scheduled Follow-up Submission	Stable Disease			42011.00	FEMALE	NO	NO	Oligodendroglioma
412	TCGA-RY-A83Y-01	0.00	TCGA-RY-A83Y-01	9.68	166.00	0.00	days	TCGA-RY-A83Y			days	166.00	days	TCGA Lower Grade Glioma (LGG)	brain lower grade glioma	Brain					45.00			NO	TCGA-RY-A83Y-F69684	TCGA-RY-A83Y	TCGA-RY-A83Y-01A			(16470.00)	485.00		0.00	166.00		2.00		NO	NO	NO				Seizures	0 - 30 Days	Scheduled Follow-up Submission				42019.00	MALE	NO	NO	Oligodendroglioma
413	TCGA-RY-A83Z-01	0.00	TCGA-RY-A83Z-01	7.90	301.00	0.00	days	TCGA-RY-A83Z	301.00	0.00	days	301.00	days	TCGA Lower Grade Glioma (LGG)	brain lower grade glioma	Brain					54.00			NO	TCGA-RY-A83Z-F69683	TCGA-RY-A83Z	TCGA-RY-A83Z-01A			(19903.00)	375.00		0.00	301.00				NO	NO	NO				Seizures	0 - 30 Days	Scheduled Follow-up Submission				42019.00	FEMALE	NO	NO	Astrocytoma
414	TCGA-RY-A840-01	0.00	TCGA-RY-A840-01	11.63	854.00	0.00	days	TCGA-RY-A840	854.00	0.00	days	854.00	days	TCGA Lower Grade Glioma (LGG)	brain lower grade glioma	Brain					47.00			NO	TCGA-RY-A840-F69714	TCGA-RY-A840	TCGA-RY-A840-01A			(17501.00)	444.00		0.00	854.00		22.00	0.00	NO	NO	NO	< 12 Years			Seizures	0 - 30 Days	Scheduled Follow-up Submission	Stable Disease	NO		42020.00	MALE	YES	YES	Oligodendroglioma
415	TCGA-RY-A843-01	0.00	TCGA-RY-A843-01	10.64	63.00	0.00	days	TCGA-RY-A843	63.00	0.00	days	63.00	days	TCGA Lower Grade Glioma (LGG)	brain lower grade glioma	Brain					30.00			NO	TCGA-RY-A843-F69739	TCGA-RY-A843	TCGA-RY-A843-01A			(11083.00)	312.00		0.00	63.00				NO	NO	NO				Seizures	> 181 Days	Scheduled Follow-up Submission	Stable Disease			42020.00	MALE	NO	NO	Astrocytoma
416	TCGA-RY-A845-01	0.00	TCGA-RY-A845-01	9.03	596.00	0.00	days	TCGA-RY-A845	596.00	0.00	days	596.00	days	TCGA Lower Grade Glioma (LGG)	brain lower grade glioma	Brain					40.00			NO	TCGA-RY-A845-F69753	TCGA-RY-A845	TCGA-RY-A845-01A			(14617.00)	193.00		0.00	596.00		1.00		NO	NO	NO	< 12 Years			Mental Status Changes	0 - 30 Days	Scheduled Follow-up Submission		NO		42020.00	FEMALE	YES	NO	Oligoastrocytoma
417	TCGA-RY-A847-01	0.00	TCGA-RY-A847-01	10.00	933.00	0.00	days	TCGA-RY-A847	933.00	0.00	days	933.00	days	TCGA Lower Grade Glioma (LGG)	brain lower grade glioma	Brain					45.00				TCGA-RY-A847-F69806	TCGA-RY-A847	TCGA-RY-A847-01A			(16732.00)	471.00		0.00	933.00		62.00			NO	NO						Scheduled Follow-up Submission	Stable Disease			42024.00	MALE		NO	Oligodendroglioma
418	TCGA-S9-A6TS-01	1.00	TCGA-S9-A6TS-01	9.29	1891.00	1.00	days	TCGA-S9-A6TS	1324.00	1.00	days	1891.00	days	TCGA Lower Grade Glioma (LGG)	brain lower grade glioma	Brain	YES	NO	YES		48.00	NO		NO	TCGA-S9-A6TS-F51945	TCGA-S9-A6TS	TCGA-S9-A6TS-01A	1324.00		(17857.00)	2402.00	1891.00	0.00		1324.00	(10.00)	0.00	NO								Scheduled Follow-up Submission	Progressive Disease	NO		41604.00	FEMALE	NO	NO	Astrocytoma
419	TCGA-S9-A6TU-01	0.00	TCGA-S9-A6TU-01	9.33	2650.00	0.00	days	TCGA-S9-A6TU	2650.00	1.00	days	2650.00	days	TCGA Lower Grade Glioma (LGG)	brain lower grade glioma	Brain	NO	NO	NO		38.00	NO		NO	TCGA-S9-A6TU-F63758	TCGA-S9-A6TU	TCGA-S9-A6TU-01A			(14108.00)	2301.00		0.00	2650.00	2650.00	(2.00)	0.00	NO						Sensory Changes	31 - 90 Days	Additional New Tumor Event	Progressive Disease	NO		41869.00	MALE	NO	NO	Astrocytoma
420	TCGA-S9-A6TV-01	0.00	TCGA-S9-A6TV-01	11.41	571.00	0.00	days	TCGA-S9-A6TV	571.00	0.00	days	571.00	days	TCGA Lower Grade Glioma (LGG)	brain lower grade glioma	Brain					50.00	NO		NO	TCGA-S9-A6TV-F67222	TCGA-S9-A6TV	TCGA-S9-A6TV-01A			(18321.00)	936.00		0.00	571.00		(14.00)	1.00	NO						Headaches	0 - 30 Days	Scheduled Follow-up Submission		NO		41946.00	MALE	NO	YES	Oligoastrocytoma
421	TCGA-S9-A6TW-01	0.00	TCGA-S9-A6TW-01	10.42	1250.00	0.00	days	TCGA-S9-A6TW	1250.00	0.00	days	1250.00	days	TCGA Lower Grade Glioma (LGG)	brain lower grade glioma	Brain					40.00	NO		NO	TCGA-S9-A6TW-F69523	TCGA-S9-A6TW				(14679.00)			0.00	1250.00		(8.00)	1.00	NO						Motor/Movement Changes	31 - 90 Days	Scheduled Follow-up Submission	Complete Remission/Response	NO		42017.00	MALE	YES	YES	Oligodendroglioma
422	TCGA-S9-A6TX-01	0.00	TCGA-S9-A6TX-01	9.97	1245.00	0.00	days	TCGA-S9-A6TX	1245.00	0.00	days	1245.00	days	TCGA Lower Grade Glioma (LGG)	brain lower grade glioma	Brain					46.00	NO		NO	TCGA-S9-A6TX-F69524	TCGA-S9-A6TX	TCGA-S9-A6TX-01A			(17025.00)	776.00		0.00	1245.00		(34.00)	0.00	NO						Headaches	0 - 30 Days	Scheduled Follow-up Submission	Partial Remission/Response	NO		42017.00	MALE	NO	YES	Oligodendroglioma
423	TCGA-S9-A6TY-01	0.00	TCGA-S9-A6TY-01	9.71	1076.00	0.00	days	TCGA-S9-A6TY	1076.00	0.00	days	1076.00	days	TCGA Lower Grade Glioma (LGG)	brain lower grade glioma	Brain					50.00	YES	hair of animals, not further specified	NO	TCGA-S9-A6TY-F69979	TCGA-S9-A6TY	TCGA-S9-A6TY-01A			(18564.00)	614.00		0.00	1076.00		(18.00)	0.00	NO						Seizures	0 - 30 Days	Scheduled Follow-up Submission	Stable Disease	NO		42030.00	MALE	NO	NO	Oligodendroglioma
424	TCGA-S9-A6TZ-01	0.00	TCGA-S9-A6TZ-01	9.64	1120.00	0.00	days	TCGA-S9-A6TZ	1120.00	0.00	days	1120.00	days	TCGA Lower Grade Glioma (LGG)	brain lower grade glioma	Brain					39.00	NO		NO	TCGA-S9-A6TZ-F69980	TCGA-S9-A6TZ				(14592.00)			0.00	1120.00		(15.00)	0.00	NO						Mental Status Changes	> 181 Days	Scheduled Follow-up Submission	Stable Disease			42030.00	FEMALE	NO	NO	Astrocytoma
425	TCGA-S9-A6U0-01	1.00	TCGA-S9-A6U0-01	12.88	742.00	1.00	days	TCGA-S9-A6U0	742.00	0.00	days	742.00	days	TCGA Lower Grade Glioma (LGG)	brain lower grade glioma	Brain					46.00	NO		NO	TCGA-S9-A6U0-F72340	TCGA-S9-A6U0	TCGA-S9-A6U0-01A			(16890.00)	495.00	742.00	0.00	498.00		(18.00)	0.00	NO						Seizures	0 - 30 Days	Scheduled Follow-up Submission	Progressive Disease	NO		42151.00	MALE	NO	NO	Astrocytoma
426	TCGA-S9-A6U1-01	0.00	TCGA-S9-A6U1-01	9.68	785.00	0.00	days	TCGA-S9-A6U1	785.00	0.00	days	785.00	days	TCGA Lower Grade Glioma (LGG)	brain lower grade glioma	Brain					22.00	NO		NO	TCGA-S9-A6U1-F69560	TCGA-S9-A6U1	TCGA-S9-A6U1-01A			(8271.00)	260.00		0.00	785.00		(13.00)	0.00	NO	NO	NO				Sensory Changes	0 - 30 Days	Scheduled Follow-up Submission	Complete Remission/Response	NO		42018.00	FEMALE	NO	NO	Astrocytoma
427	TCGA-S9-A6U2-01	0.00	TCGA-S9-A6U2-01	9.68	908.00	0.00	days	TCGA-S9-A6U2	908.00	0.00	days	908.00	days	TCGA Lower Grade Glioma (LGG)	brain lower grade glioma	Brain					48.00	NO		NO	TCGA-S9-A6U2-F69983	TCGA-S9-A6U2	TCGA-S9-A6U2-01A			(17800.00)	430.00		0.00	908.00		(6.00)	0.00	NO						Seizures	0 - 30 Days	Scheduled Follow-up Submission	Stable Disease	NO		42030.00	FEMALE	NO	NO	Oligodendroglioma
428	TCGA-S9-A6U5-01	0.00	TCGA-S9-A6U5-01	10.26	992.00	0.00	days	TCGA-S9-A6U5	992.00	0.00	days	992.00	days	TCGA Lower Grade Glioma (LGG)	brain lower grade glioma	Brain	NO	NO			33.00	NO		NO	TCGA-S9-A6U5-F62932	TCGA-S9-A6U5	TCGA-S9-A6U5-01A			(12417.00)	636.00		0.00	992.00		(6.00)	0.00	NO						Seizures	31 - 90 Days	Scheduled Follow-up Submission	Stable Disease	NO		41849.00	MALE	NO	NO	Astrocytoma
429	TCGA-S9-A6U6-01	0.00	TCGA-S9-A6U6-01	10.28	1069.00	0.00	days	TCGA-S9-A6U6	1069.00	0.00	days	1069.00	days	TCGA Lower Grade Glioma (LGG)	brain lower grade glioma	Brain					28.00	NO		NO	TCGA-S9-A6U6-F72342	TCGA-S9-A6U6	TCGA-S9-A6U6-01A			(10253.00)	382.00		0.00	1069.00		(3.00)	0.00	NO								Scheduled Follow-up Submission		NO		42151.00	MALE	NO	NO	Astrocytoma
430	TCGA-S9-A6U8-01	1.00	TCGA-S9-A6U8-01	10.17	2988.00	1.00	days	TCGA-S9-A6U8	367.00	1.00	days	2988.00	days	TCGA Lower Grade Glioma (LGG)	brain lower grade glioma	Brain	NO	NO			24.00	NO		NO	TCGA-S9-A6U8-F65495	TCGA-S9-A6U8	TCGA-S9-A6U8-01A			(9044.00)	2962.00	2988.00	0.00	2878.00	367.00	(31.00)	0.00	NO						Seizures	> 181 Days	Scheduled Follow-up Submission	Progressive Disease	NO		41906.00	MALE	NO	NO	Astrocytoma

431	TCGA-S9-A6U9-01	0.00	TCGA-S9-A6U9-01	10.30	2918.00	0.00	days	TCGA-S9-A6U9	2918.00	0.00	days	2918.00	days	TCGA Lower Grade Glioma (LGG)	brain lower grade glioma	Brain	NO	NO			36.00	NO		NO	TCGA-S9-A6U9-F62934	TCGA-S9-A6U9	TCGA-S9-A6U9-01A			(13460.00)	2706.00		0.00	2918.00				NO	NO	NO				Seizures	> 181 Days	Scheduled Follow-up Submission	Stable Disease	NO		41849.00	MALE	NO	NO	Astrocytoma
432	TCGA-S9-A6UA-01	1.00	TCGA-S9-A6UA-01	9.27	178.00	1.00	days	TCGA-S9-A6UA	161.00	1.00	days	178.00	days	TCGA Lower Grade Glioma (LGG)	brain lower grade glioma	Brain	NO	NO	NO		66.00	NO		NO	TCGA-S9-A6UA-F51947	TCGA-S9-A6UA	TCGA-S9-A6UA-01A			(24242.00)	1989.00	178.00	0.00		161.00	(6.00)	2.00	NO						Mental Status Changes	> 181 Days	Scheduled Follow-up Submission	Progressive Disease	NO		41604.00	MALE	NO	NO	Astrocytoma
433	TCGA-S9-A6UB-01	0.00	TCGA-S9-A6UB-01	9.11	3761.00	0.00	days	TCGA-S9-A6UB	3761.00	0.00	days	3761.00	days	TCGA Lower Grade Glioma (LGG)	brain lower grade glioma	Brain					52.00	NO		YES	TCGA-S9-A6UB-F72418	TCGA-S9-A6UB	TCGA-S9-A6UB-01A			(19223.00)	3073.00		0.00	3761.00		(1.00)	0.00	NO								Scheduled Follow-up Submission		NO		42170.00	MALE	NO	NO	Oligodendroglioma
434	TCGA-S9-A6WD-01	0.00	TCGA-S9-A6WD-01	10.06	2289.00	0.00	days	TCGA-S9-A6WD	2289.00	0.00	days	2289.00	days	TCGA Lower Grade Glioma (LGG)	brain lower grade glioma	Brain					58.00	NO		NO	TCGA-S9-A6WD-F69986	TCGA-S9-A6WD	TCGA-S9-A6WD-01A			(21231.00)	1922.00		0.00	2289.00		(2.00)	0.00	NO						Seizures	> 181 Days	Scheduled Follow-up Submission	Stable Disease	NO		42030.00	MALE	NO	NO	Oligodendroglioma
435	TCGA-S9-A6WE-01	0.00	TCGA-S9-A6WE-01	8.78	4113.00	0.00	days	TCGA-S9-A6WE	4113.00	0.00	days	4113.00	days	TCGA Lower Grade Glioma (LGG)	brain lower grade glioma	Brain					34.00	NO		NO	TCGA-S9-A6WE-F72356	TCGA-S9-A6WE	TCGA-S9-A6WE-01A			(12488.00)	3434.00		0.00	4113.00		(4.00)	0.00	NO						Sensory Changes	> 181 Days	Scheduled Follow-up Submission		NO		42157.00	MALE	NO	NO	Oligodendroglioma
436	TCGA-S9-A6WG-01	0.00	TCGA-S9-A6WG-01	9.54	2602.00	0.00	days	TCGA-S9-A6WG	2602.00	0.00	days	2602.00	days	TCGA Lower Grade Glioma (LGG)	brain lower grade glioma	Brain					31.00	NO		NO	TCGA-S9-A6WG-F69988	TCGA-S9-A6WG	TCGA-S9-A6WG-01A			(11509.00)	2098.00		0.00	2602.00		(12.00)	0.00	NO						Seizures	0 - 30 Days	Scheduled Follow-up Submission	Stable Disease	NO		42030.00	MALE	NO	NO	Astrocytoma
437	TCGA-S9-A6WH-01	0.00	TCGA-S9-A6WH-01	7.94	1173.00	0.00	days	TCGA-S9-A6WH	1173.00	0.00	days	1173.00	days	TCGA Lower Grade Glioma (LGG)	brain lower grade glioma	Brain					73.00	NO		NO	TCGA-S9-A6WH-F69989	TCGA-S9-A6WH	TCGA-S9-A6WH-01A			(26904.00)	697.00		0.00	1173.00		(14.00)	1.00	NO						Motor/Movement Changes		Scheduled Follow-up Submission	Stable Disease	NO		42030.00	FEMALE	NO	NO	Oligoastrocytoma
438	TCGA-S9-A6WI-01	0.00	TCGA-S9-A6WI-01	9.50	2585.00	0.00	days	TCGA-S9-A6WI	2585.00	0.00	days	2585.00	days	TCGA Lower Grade Glioma (LGG)	brain lower grade glioma	Brain	NO	NO			56.00	NO		NO	TCGA-S9-A6WI-F63762	TCGA-S9-A6WI				(20609.00)			0.00	2585.00		(1.00)	0.00	NO						Headaches		Scheduled Follow-up Submission	Stable Disease	NO		41869.00	FEMALE	YES	YES	Oligoastrocytoma
439	TCGA-S9-A6WL-01	0.00	TCGA-S9-A6WL-01	9.59	946.00	0.00	days	TCGA-S9-A6WL	946.00	0.00	days	946.00	days	TCGA Lower Grade Glioma (LGG)	brain lower grade glioma	Brain	NO	YES			52.00	NO		NO	TCGA-S9-A6WL-F67227	TCGA-S9-A6WL				(19234.00)			0.00	946.00		(2.00)	0.00	NO						Seizures	0 - 30 Days	Scheduled Follow-up Submission		NO		41946.00	MALE	NO	NO	Astrocytoma
440	TCGA-S9-A6WM-01	0.00	TCGA-S9-A6WM-01	10.83	604.00	0.00	days	TCGA-S9-A6WM	505.00	1.00	days	604.00	days	TCGA Lower Grade Glioma (LGG)	brain lower grade glioma	Brain	YES	NO	NO		59.00	NO		NO	TCGA-S9-A6WM-F63843	TCGA-S9-A6WM				(21761.00)			0.00	604.00	505.00	(9.00)	1.00	NO						Headaches	0 - 30 Days	Additional New Tumor Event	Progressive Disease	NO		41871.00	FEMALE	NO	YES	Astrocytoma
441	TCGA-S9-A6WN-01	0.00	TCGA-S9-A6WN-01	13.16	805.00	0.00	days	TCGA-S9-A6WN	805.00	1.00	days	805.00	days	TCGA Lower Grade Glioma (LGG)	brain lower grade glioma	Brain	YES	NO			38.00	NO		NO	TCGA-S9-A6WN-F72374	TCGA-S9-A6WN	TCGA-S9-A6WN-01A			(13961.00)	181.00		0.00	805.00	805.00	(14.00)	1.00	NO						Seizures	0 - 30 Days	Additional New Tumor Event	Progressive Disease	NO		42164.00	FEMALE	NO	NO	Astrocytoma
442	TCGA-S9-A6WO-01	0.00	TCGA-S9-A6WO-01	9.14	566.00	0.00	days	TCGA-S9-A6WO	566.00	0.00	days	566.00	days	TCGA Lower Grade Glioma (LGG)	brain lower grade glioma	Brain	NO	NO			29.00	NO		NO	TCGA-S9-A6WO-F65749	TCGA-S9-A6WO				(10933.00)			0.00	566.00		(4.00)	0.00	NO						Seizures	31 - 90 Days	Scheduled Follow-up Submission	Stable Disease	NO		41912.00	MALE	NO	NO	Astrocytoma
443	TCGA-S9-A6WP-01	0.00	TCGA-S9-A6WP-01	10.29	567.00	0.00	days	TCGA-S9-A6WP	567.00	0.00	days	567.00	days	TCGA Lower Grade Glioma (LGG)	brain lower grade glioma	Brain					42.00	NO		NO	TCGA-S9-A6WP-F69994	TCGA-S9-A6WP	TCGA-S9-A6WP-01A			(15658.00)	111.00		0.00	567.00		(3.00)	0.00	NO						Seizures	0 - 30 Days	Scheduled Follow-up Submission	Stable Disease	NO		42030.00	MALE	NO	NO	Oligoastrocytoma
444	TCGA-S9-A6WQ-01	0.00	TCGA-S9-A6WQ-01	10.85	428.00	0.00	days	TCGA-S9-A6WQ	428.00	0.00	days	428.00	days	TCGA Lower Grade Glioma (LGG)	brain lower grade glioma	Brain	NO	NO			57.00	NO		NO	TCGA-S9-A6WQ-F62938	TCGA-S9-A6WQ	TCGA-S9-A6WQ-01A			(21133.00)	104.00		0.00	428.00		(7.00)	0.00	NO						Seizures	91 - 180 Days	Scheduled Follow-up Submission	Stable Disease	NO		41849.00	FEMALE	NO	NO	Oligoastrocytoma
445	TCGA-S9-A7IQ-01	0.00	TCGA-S9-A7IQ-01	9.41	1099.00	0.00	days	TCGA-S9-A7IQ	1099.00	0.00	days	1099.00	days	TCGA Lower Grade Glioma (LGG)	brain lower grade glioma	Brain	NO	NO			45.00	NO		NO	TCGA-S9-A7IQ-F65750	TCGA-S9-A7IQ	TCGA-S9-A7IQ-01A			(16512.00)	735.00		0.00	1099.00		(14.00)	0.00	NO						Sensory Changes	0 - 30 Days	Scheduled Follow-up Submission	Stable Disease	NO		41912.00	FEMALE	NO	NO	Oligoastrocytoma
446	TCGA-S9-A7IS-01	1.00	TCGA-S9-A7IS-01	10.92	241.00	1.00	days	TCGA-S9-A7IS	241.00	0.00	days	241.00	days	TCGA Lower Grade Glioma (LGG)	brain lower grade glioma	Brain					33.00	NO		NO	TCGA-S9-A7IS-F51948	TCGA-S9-A7IS	TCGA-S9-A7IS-01A			(12402.00)	3506.00	241.00	0.00			(2.00)	2.00	NO						Mental Status Changes		Scheduled Follow-up Submission	Progressive Disease	NO		41604.00	FEMALE	NO	NO	Astrocytoma
447	TCGA-S9-A7IX-01	1.00	TCGA-S9-A7IX-01	9.73	819.00	1.00	days	TCGA-S9-A7IX			days	819.00	days	TCGA Lower Grade Glioma (LGG)	brain lower grade glioma	Brain					57.00	NO		NO	TCGA-S9-A7IX-F72344	TCGA-S9-A7IX	TCGA-S9-A7IX-01A			(21095.00)	540.00	819.00	0.00	597.00		(2.00)	0.00	NO						Motor/Movement Changes	31 - 90 Days	Scheduled Follow-up Submission		NO		42151.00	MALE	YES	NO	Astrocytoma
448	TCGA-S9-A7IY-01	0.00	TCGA-S9-A7IY-01	10.69	715.00	0.00	days	TCGA-S9-A7IY	715.00	0.00	days	715.00	days	TCGA Lower Grade Glioma (LGG)	brain lower grade glioma	Brain	NO	NO			39.00	NO		NO	TCGA-S9-A7IY-F65751	TCGA-S9-A7IY	TCGA-S9-A7IY-01A			(14422.00)	398.00		0.00	715.00		(6.00)	2.00	NO						Motor/Movement Changes	0 - 30 Days	Scheduled Follow-up Submission	Partial Remission/Response	NO		41912.00	MALE	NO	NO	Oligoastrocytoma
449	TCGA-S9-A7IZ-01	0.00	TCGA-S9-A7IZ-01	10.99	608.00	0.00	days	TCGA-S9-A7IZ	467.00	1.00	days	608.00	days	TCGA Lower Grade Glioma (LGG)	brain lower grade glioma	Brain	YES	NO			48.00	NO		NO	TCGA-S9-A7IZ-F69998	TCGA-S9-A7IZ	TCGA-S9-A7IZ-01A			(17874.00)	140.00		0.00	608.00	467.00	(5.00)	1.00	NO						Seizures	0 - 30 Days	Additional New Tumor Event	Stable Disease	NO		42030.00	FEMALE	NO	NO	Astrocytoma
450	TCGA-S9-A7J0-01	0.00	TCGA-S9-A7J0-01	7.61	609.00	0.00	days	TCGA-S9-A7J0	390.00	1.00	days	609.00	days	TCGA Lower Grade Glioma (LGG)	brain lower grade glioma	Brain	YES	NO			30.00	NO		NO	TCGA-S9-A7J0-F65752	TCGA-S9-A7J0	TCGA-S9-A7J0-01A			(10989.00)	229.00		0.00	609.00	390.00	(7.00)	0.00	NO						Seizures	0 - 30 Days	Additional New Tumor Event	Progressive Disease	YES	Citrus fruits	41912.00	FEMALE	NO	NO	Oligodendroglioma
451	TCGA-S9-A7J1-01	0.00	TCGA-S9-A7J1-01	11.20	249.00	0.00	days	TCGA-S9-A7J1	249.00	0.00	days	249.00	days	TCGA Lower Grade Glioma (LGG)	brain lower grade glioma	Brain					43.00	NO		NO	TCGA-S9-A7J1-F71265	TCGA-S9-A7J1	TCGA-S9-A7J1-01A			(15926.00)	184.00		0.00	249.00		(17.00)	3.00	NO						Seizures	31 - 90 Days	Scheduled Follow-up Submission		NO		42087.00	MALE	NO	NO	Oligodendroglioma
452	TCGA-S9-A7J2-01	0.00	TCGA-S9-A7J2-01	9.40	62.00	0.00	days	TCGA-S9-A7J2	62.00	0.00	days	62.00	days	TCGA Lower Grade Glioma (LGG)	brain lower grade glioma	Brain					25.00	NO		NO	TCGA-S9-A7J2-F71266	TCGA-S9-A7J2	TCGA-S9-A7J2-01A			(9345.00)	194.00		0.00	62.00		(4.00)	1.00	NO						Mental Status Changes	> 181 Days	Scheduled Follow-up Submission		NO		42087.00	MALE	NO	NO	Oligodendroglioma
453	TCGA-S9-A7J3-01	0.00	TCGA-S9-A7J3-01	10.02	629.00	0.00	days	TCGA-S9-A7J3	629.00	0.00	days	629.00	days	TCGA Lower Grade Glioma (LGG)	brain lower grade glioma	Brain					52.00	NO		NO	TCGA-S9-A7J3-F70008	TCGA-S9-A7J3	TCGA-S9-A7J3-01A			(19056.00)	182.00		0.00	629.00		(14.00)	0.00	NO						Seizures	31 - 90 Days	Scheduled Follow-up Submission	Stable Disease	NO		42030.00	FEMALE	NO	NO	Oligodendroglioma
454	TCGA-S9-A7QW-01	0.00	TCGA-S9-A7QW-01	7.55	458.00	0.00	days	TCGA-S9-A7QW	382.00	1.00	days	458.00	days	TCGA Lower Grade Glioma (LGG)	brain lower grade glioma	Brain	YES	NO			54.00	NO		NO	TCGA-S9-A7QW-F66223	TCGA-S9-A7QW				(19869.00)			0.00	458.00	382.00	(4.00)	0.00	NO								Additional New Tumor Event	Stable Disease	NO		41918.00	FEMALE	NO	NO	Astrocytoma
455	TCGA-S9-A7QX-01	0.00	TCGA-S9-A7QX-01	10.17	564.00	0.00	days	TCGA-S9-A7QX	564.00	0.00	days	564.00	days	TCGA Lower Grade Glioma (LGG)	brain lower grade glioma	Brain					36.00	NO		NO	TCGA-S9-A7QX-F70010	TCGA-S9-A7QX	TCGA-S9-A7QX-01A			(13332.00)	81.00		0.00	564.00		(3.00)	0.00	NO	NO	NO				Sensory Changes	> 181 Days	Scheduled Follow-up Submission	Stable Disease	NO		42030.00	FEMALE	NO	NO	Astrocytoma
456	TCGA-S9-A7QY-01	0.00	TCGA-S9-A7QY-01	10.43	863.00	0.00	days	TCGA-S9-A7QY	863.00	0.00	days	863.00	days	TCGA Lower Grade Glioma (LGG)	brain lower grade glioma	Brain	NO	NO			35.00	NO		NO	TCGA-S9-A7QY-F62949	TCGA-S9-A7QY	TCGA-S9-A7QY-01A			(12853.00)	560.00		0.00	863.00		(2.00)	0.00	NO						Seizures	> 181 Days	Scheduled Follow-up Submission	Stable Disease	NO		41849.00	FEMALE	NO	NO	Oligoastrocytoma
457	TCGA-S9-A7QZ-01	0.00	TCGA-S9-A7QZ-01	9.30	826.00	0.00	days	TCGA-S9-A7QZ	774.00	1.00	days	826.00	days	TCGA Lower Grade Glioma (LGG)	brain lower grade glioma	Brain	NO	NO	YES		41.00	NO		NO	TCGA-S9-A7QZ-F70012	TCGA-S9-A7QZ	TCGA-S9-A7QZ-01A	826.00		(15300.00)	368.00		0.00	826.00	774.00	(3.00)	0.00	NO						Seizures	> 181 Days	Additional New Tumor Event	Partial Remission/Response	NO		42030.00	MALE	YES	NO	Oligodendroglioma
458	TCGA-S9-A7R1-01	1.00	TCGA-S9-A7R1-01	9.06	5166.00	1.00	days	TCGA-S9-A7R1	5166.00	0.00	days	5166.00	days	TCGA Lower Grade Glioma (LGG)	brain lower grade glioma	Brain	NO	NO			35.00	NO		NO	TCGA-S9-A7R1-F67312	TCGA-S9-A7R1	TCGA-S9-A7R1-01A			(12828.00)	5213.00	5166.00	0.00	5000.00		(122.00)	1.00	NO						Seizures	> 181 Days	Scheduled Follow-up Submission		NO		41947.00	MALE	NO	NO	Oligodendroglioma
459	TCGA-S9-A7R2-01	1.00	TCGA-S9-A7R2-01	10.69	316.00	1.00	days	TCGA-S9-A7R2	316.00	0.00	days	316.00	days	TCGA Lower Grade Glioma (LGG)	brain lower grade glioma	Brain					69.00	NO		NO		TCGA-S9-A7R2	TCGA-S9-A7R2-01A			(25419.00)	2027.00	316.00	0.00			(2.00)	1.00	NO						Seizures	> 181 Days			NO		41620.00	MALE	NO	NO	Astrocytoma
460	TCGA-S9-A7R3-01	0.00	TCGA-S9-A7R3-01	10.80	3013.00	0.00	days	TCGA-S9-A7R3	3013.00	0.00	days	3013.00	days	TCGA Lower Grade Glioma (LGG)	brain lower grade glioma	Brain	NO	NO			28.00	NO		NO	TCGA-S9-A7R3-F62951	TCGA-S9-A7R3	TCGA-S9-A7R3-01A			(10342.00)	2706.00		0.00	3013.00		(2.00)	0.00	NO						Headaches	> 181 Days	Scheduled Follow-up Submission	Stable Disease	NO		41849.00	FEMALE	NO	YES	Astrocytoma
461	TCGA-S9-A7R4-01	0.00	TCGA-S9-A7R4-01	10.48	914.00	0.00	days	TCGA-S9-A7R4	914.00	0.00	days	914.00	days	TCGA Lower Grade Glioma (LGG)	brain lower grade glioma	Brain					46.00	NO		NO	TCGA-S9-A7R4-F67313	TCGA-S9-A7R4	TCGA-S9-A7R4-01A			(17134.00)	565.00		0.00	914.00		(8.00)	1.00	NO						Seizures	0 - 30 Days	Scheduled Follow-up Submission		NO		41947.00	MALE	NO	NO	Astrocytoma
462	TCGA-S9-A7R7-01	0.00	TCGA-S9-A7R7-01	12.18	3000.00	0.00	days	TCGA-S9-A7R7	2894.00	1.00	days	3000.00	days	TCGA Lower Grade Glioma (LGG)	brain lower grade glioma	Brain	YES	YES	YES		27.00	NO		NO	TCGA-S9-A7R7-F66258	TCGA-S9-A7R7	TCGA-S9-A7R7-01A	2976.00		(10108.00)	2647.00		0.00	3000.00	2894.00	(8.00)	1.00	NO						Seizures	0 - 30 Days	Additional New Tumor Event	Progressive Disease	NO		41919.00	MALE	NO	NO	Astrocytoma
463	TCGA-S9-A7R8-01	1.00	TCGA-S9-A7R8-01	9.77	961.00	1.00	days	TCGA-S9-A7R8			days	961.00	days	TCGA Lower Grade Glioma (LGG)	brain lower grade glioma	Brain					44.00	NO		NO		TCGA-S9-A7R8	TCGA-S9-A7R8-01A			(16275.00)	3282.00	961.00	0.00			(1.00)	1.00	NO						Seizures	31 - 90 Days			NO		41619.00	FEMALE	NO	NO	Astrocytoma
464	TCGA-S9-A89V-01	0.00	TCGA-S9-A89V-01	8.25	569.00	0.00	days	TCGA-S9-A89V	221.00	1.00	days	569.00	days	TCGA Lower Grade Glioma (LGG)	brain lower grade glioma	Brain					70.00	NO		NO	TCGA-S9-A89V-F67315	TCGA-S9-A89V				(25570.00)			0.00	569.00	221.00	(32.00)	1.00	NO						Motor/Movement Changes	> 181 Days	Additional New Tumor Event		NO		41947.00	MALE	NO	NO	Astrocytoma
465	TCGA-S9-A89Z-01	0.00	TCGA-S9-A89Z-01	12.83	623.00	0.00	days	TCGA-S9-A89Z	623.00	0.00	days	623.00	days	TCGA Lower Grade Glioma (LGG)	brain lower grade glioma	Brain					40.00	NO		NO	TCGA-S9-A89Z-F72341	TCGA-S9-A89Z	TCGA-S9-A89Z-01A			(14858.00)	63.00		0.00	623.00		(10.00)	1.00	NO	YES	NO				Seizures	31 - 90 Days	Scheduled Follow-up Submission	Stable Disease	NO		42151.00	MALE	NO	NO	Astrocytoma
466	TCGA-TM-A7C3-01	1.00	TCGA-TM-A7C3-01	10.79	1666.00	1.00	days	TCGA-TM-A7C3	1666.00	0.00	days	1666.00	days	TCGA Lower Grade Glioma (LGG)	brain lower grade glioma	Brain					43.00	NO		YES	TCGA-TM-A7C3-F63557	TCGA-TM-A7C3	TCGA-TM-A7C3-01A			(16021.00)	1409.00	1666.00	0.00	1504.00		0.00	0.00	YES	YES	NO	< 12 Years			Seizures	0 - 30 Days	Scheduled Follow-up Submission	Progressive Disease	NO		41863.00	FEMALE	YES	NO	Astrocytoma

467	TCGA-TM-A7C4-01	0.00	TCGA-TM-A7C4-01	10.03	1470.00	0.00	days	TCGA-TM-A7C4	1470.00	0.00	days	1470.00	days	TCGA Lower Grade Glioma (LGG)	brain lower grade glioma	Brain					39.00	NO		NO	TCGA-TM-A7C4-F63553	TCGA-TM-A7C4	TCGA-TM-A7C4-01A			(14603.00)	1215.00		0.00	1470.00		0.00	0.00	YES	NO	NO	> 20 Years			Seizures	0 - 30 Days	Scheduled Follow-up Submission	Complete Remission/Response	NO		41863.00	FEMALE	NO	NO	Astrocytoma
468	TCGA-TM-A7C5-01	0.00	TCGA-TM-A7C5-01	8.94	1500.00	0.00	days	TCGA-TM-A7C5	1500.00	0.00	days	1500.00	days	TCGA Lower Grade Glioma (LGG)	brain lower grade glioma	Brain					30.00	NO		YES	TCGA-TM-A7C5-F63554	TCGA-TM-A7C5	TCGA-TM-A7C5-01A			(11286.00)	1194.00		0.00	1500.00		0.00	0.00	NO	NO	NO	< 12 Years			Seizures	0 - 30 Days	Scheduled Follow-up Submission	Complete Remission/Response	NO		41863.00	MALE	NO	NO	Oligoastrocytoma
469	TCGA-TM-A7CA-01	0.00	TCGA-TM-A7CA-01	9.61	1058.00	0.00	days	TCGA-TM-A7CA	1058.00	0.00	days	1058.00	days	TCGA Lower Grade Glioma (LGG)	brain lower grade glioma	Brain					44.00	NO		YES	TCGA-TM-A7CA-F63555	TCGA-TM-A7CA	TCGA-TM-A7CA-01A			(16415.00)	830.00		0.00	1058.00		0.00	0.00	NO	YES	NO	> 20 Years			Seizures	0 - 30 Days	Scheduled Follow-up Submission	Complete Remission/Response	NO		41863.00	MALE	NO	NO	Astrocytoma
470	TCGA-TM-A7CF-01	0.00	TCGA-TM-A7CF-01	9.93	1989.00	0.00	days	TCGA-TM-A7CF	1989.00	0.00	days	1989.00	days	TCGA Lower Grade Glioma (LGG)	brain lower grade glioma	Brain					41.00	NO		YES	TCGA-TM-A7CF-F70345	TCGA-TM-A7CF	TCGA-TM-A7CF-01A			(15178.00)	1453.00		0.00	1989.00		0.00	0.00	NO	NO	NO	< 12 Years			Seizures	0 - 30 Days	Scheduled Follow-up Submission	Stable Disease	NO		42040.00	FEMALE	YES	NO	Astrocytoma
471	TCGA-TM-A7CF-02	0.00	TCGA-TM-A7CF-02	10.53	1989.00	0.00	days	TCGA-TM-A7CF	1989.00	0.00	days	1989.00	days	TCGA Lower Grade Glioma (LGG)	brain lower grade glioma	Brain					41.00	NO		YES	TCGA-TM-A7CF-F70345	TCGA-TM-A7CF	TCGA-TM-A7CF-02A			(15178.00)	1453.00		0.00	1989.00		0.00	0.00	NO	NO	NO	< 12 Years			Seizures	0 - 30 Days	Scheduled Follow-up Submission	Stable Disease	NO		42040.00	FEMALE	YES	NO	Astrocytoma
472	TCGA-TM-A84B-01	1.00	TCGA-TM-A84B-01	11.66	758.00	1.00	days	TCGA-TM-A84B	758.00	0.00	days	758.00	days	TCGA Lower Grade Glioma (LGG)	brain lower grade glioma	Brain					40.00	NO		NO	TCGA-TM-A84B-F65302	TCGA-TM-A84B				(14933.00)		758.00	0.00	365.00		0.00	0.00	NO	NO	NO				Seizures	0 - 30 Days	Scheduled Follow-up Submission	Progressive Disease	NO		41904.00	MALE	NO	NO	Astrocytoma
473	TCGA-TM-A84C-01	1.00	TCGA-TM-A84C-01	10.90	492.00	1.00	days	TCGA-TM-A84C	492.00	0.00	days	492.00	days	TCGA Lower Grade Glioma (LGG)	brain lower grade glioma	Brain					32.00	NO		YES	TCGA-TM-A84C-F64988	TCGA-TM-A84C	TCGA-TM-A84C-01A			(11849.00)	459.00	492.00	0.00	459.00		0.00	1.00	NO	YES	NO	< 12 Years			Visual Changes	31 - 90 Days	Scheduled Follow-up Submission	Progressive Disease	NO		41899.00	MALE	NO	NO	Astrocytoma
474	TCGA-TM-A84F-01	0.00	TCGA-TM-A84F-01	11.08	1796.00	0.00	days	TCGA-TM-A84F	1796.00	0.00	days	1796.00	days	TCGA Lower Grade Glioma (LGG)	brain lower grade glioma	Brain					48.00	NO		NO	TCGA-TM-A84F-F65393	TCGA-TM-A84F	TCGA-TM-A84F-01A			(17744.00)	1471.00		0.00	1796.00		0.00	0.00	NO	NO	NO				Visual Changes	0 - 30 Days	Scheduled Follow-up Submission	Complete Remission/Response	NO		41920.00	MALE	NO	YES	Astrocytoma
475	TCGA-TM-A84G-01	0.00	TCGA-TM-A84G-01	8.69	1236.00	0.00	days	TCGA-TM-A84G	1236.00	0.00	days	1236.00	days	TCGA Lower Grade Glioma (LGG)	brain lower grade glioma	Brain					54.00	NO		NO	TCGA-TM-A84G-F65394	TCGA-TM-A84G				(20014.00)			0.00	1236.00		0.00	0.00	NO	NO	NO				Headaches	0 - 30 Days	Scheduled Follow-up Submission	Complete Remission/Response	NO		41905.00	FEMALE	NO	YES	Oligodendroglioma
476	TCGA-TM-A84H-01	0.00	TCGA-TM-A84H-01	8.29	926.00	0.00	days	TCGA-TM-A84H	926.00	0.00	days	926.00	days	TCGA Lower Grade Glioma (LGG)	brain lower grade glioma	Brain					44.00	NO		NO	TCGA-TM-A84H-F65389	TCGA-TM-A84H	TCGA-TM-A84H-01A			(16277.00)	627.00		0.00	926.00		0.00	0.00	NO	YES	NO				Headaches	0 - 30 Days	Scheduled Follow-up Submission	Complete Remission/Response	NO		41904.00	FEMALE	NO	YES	Oligoastrocytoma
477	TCGA-TM-A84I-01	0.00	TCGA-TM-A84I-01	8.51	854.00	0.00	days	TCGA-TM-A84I	854.00	0.00	days	854.00	days	TCGA Lower Grade Glioma (LGG)	brain lower grade glioma	Brain					30.00	NO		NO	TCGA-TM-A84I-F65390	TCGA-TM-A84I				(11221.00)			0.00	854.00		0.00	0.00	NO	YES	NO				Motor/Movement Changes	0 - 30 Days	Scheduled Follow-up Submission	Complete Remission/Response	NO		41904.00	MALE	NO	NO	Astrocytoma
478	TCGA-TM-A84J-01	0.00	TCGA-TM-A84J-01	10.92	735.00	0.00	days	TCGA-TM-A84J	454.00	1.00	days	735.00	days	TCGA Lower Grade Glioma (LGG)	brain lower grade glioma	Brain	YES	NO			63.00	NO		NO	TCGA-TM-A84J-F65391	TCGA-TM-A84J	TCGA-TM-A84J-01A			(23091.00)	415.00		0.00	735.00	454.00	0.00	1.00	NO	NO	NO				Visual Changes	0 - 30 Days	Scheduled Follow-up Submission	Progressive Disease	NO		41912.00	MALE	NO	NO	Oligodendroglioma
479	TCGA-TM-A84L-01	1.00	TCGA-TM-A84L-01	11.33	1242.00	1.00	days	TCGA-TM-A84L	1045.00	1.00	days	1242.00	days	TCGA Lower Grade Glioma (LGG)	brain lower grade glioma	Brain	YES	YES	YES		31.00	NO		NO	TCGA-TM-A84L-F56505	TCGA-TM-A84L		1045.00		(11498.00)		1242.00	0.00		1045.00	0.00	0.00	NO	NO	NO				Seizures	31 - 90 Days	Scheduled Follow-up Submission	Progressive Disease	NO		41687.00	MALE	NO	NO	Oligoastrocytoma
480	TCGA-TM-A84M-01	0.00	TCGA-TM-A84M-01	9.96	754.00	0.00	days	TCGA-TM-A84M	754.00	0.00	days	754.00	days	TCGA Lower Grade Glioma (LGG)	brain lower grade glioma	Brain					40.00	NO		NO	TCGA-TM-A84M-F65392	TCGA-TM-A84M	TCGA-TM-A84M-01A			(14660.00)	455.00		0.00	754.00		0.00	0.00	NO	NO	NO				Motor/Movement Changes	31 - 90 Days	Scheduled Follow-up Submission	Complete Remission/Response	NO		41905.00	MALE	NO	NO	Oligodendroglioma
481	TCGA-TM-A84O-01	1.00	TCGA-TM-A84O-01	10.09	1011.00	1.00	days	TCGA-TM-A84O	1011.00	0.00	days	1011.00	days	TCGA Lower Grade Glioma (LGG)	brain lower grade glioma	Brain					61.00	NO		NO	TCGA-TM-A84O-F65303	TCGA-TM-A84O	TCGA-TM-A84O-01A			(22327.00)	920.00	1011.00	0.00			0.00	0.00	NO						Seizures	0 - 30 Days	Scheduled Follow-up Submission	Progressive Disease	NO		41904.00	FEMALE	NO	NO	Oligodendroglioma
482	TCGA-TM-A84Q-01	0.00	TCGA-TM-A84Q-01	9.81	772.00	0.00	days	TCGA-TM-A84Q	455.00	1.00	days	772.00	days	TCGA Lower Grade Glioma (LGG)	brain lower grade glioma	Brain	NO	NO			31.00	NO		YES	TCGA-TM-A84Q-F65305	TCGA-TM-A84Q	TCGA-TM-A84Q-01A			(11668.00)	592.00		0.00	772.00	455.00	0.00	1.00	NO	NO	NO	> 20 Years			Mental Status Changes	0 - 30 Days	Scheduled Follow-up Submission	Complete Remission/Response	NO		41904.00	MALE	NO	NO	Astrocytoma
483	TCGA-TM-A84R-01	0.00	TCGA-TM-A84R-01	9.53	566.00	0.00	days	TCGA-TM-A84R	566.00	0.00	days	566.00	days	TCGA Lower Grade Glioma (LGG)	brain lower grade glioma	Brain					46.00	NO		NO	TCGA-TM-A84R-F65388	TCGA-TM-A84R	TCGA-TM-A84R-01A			(16843.00)	346.00		0.00	566.00		0.00	1.00	NO	NO	NO				Seizures	0 - 30 Days	Scheduled Follow-up Submission	Complete Remission/Response	NO		41904.00	MALE	NO	YES	Oligodendroglioma
484	TCGA-TM-A84S-01	0.00	TCGA-TM-A84S-01	11.11	454.00	0.00	days	TCGA-TM-A84S	454.00	0.00	days	454.00	days	TCGA Lower Grade Glioma (LGG)	brain lower grade glioma	Brain					36.00	NO		NO	TCGA-TM-A84S-F65304	TCGA-TM-A84S	TCGA-TM-A84S-01A			(13424.00)	163.00		0.00	454.00		0.00	1.00	NO	YES	YES				Seizures	31 - 90 Days	Scheduled Follow-up Submission	Complete Remission/Response	NO		41904.00	MALE	NO	NO	Oligodendroglioma
485	TCGA-TM-A84T-01	0.00	TCGA-TM-A84T-01	9.41	724.00	0.00	days	TCGA-TM-A84T	724.00	0.00	days	724.00	days	TCGA Lower Grade Glioma (LGG)	brain lower grade glioma	Brain					19.00	NO		NO	TCGA-TM-A84T-F65306	TCGA-TM-A84T				(6994.00)			0.00	724.00		0.00	0.00	NO	NO	NO				Seizures	0 - 30 Days	Scheduled Follow-up Submission	Stable Disease	NO		41904.00	MALE	NO	NO	Oligoastrocytoma
486	TCGA-TQ-A7RF-01	0.00	TCGA-TQ-A7RF-01	10.55	650.00	0.00	days	TCGA-TQ-A7RF	650.00	0.00	days	650.00	days	TCGA Lower Grade Glioma (LGG)	brain lower grade glioma	Brain					27.00	NO		NO	TCGA-TQ-A7RF-F61331	TCGA-TQ-A7RF	TCGA-TQ-A7RF-01A			(9919.00)	373.00		0.00	650.00				NO	NO	NO				Headaches	> 181 Days			NO		42054.00	FEMALE	NO	YES	Oligodendroglioma
487	TCGA-TQ-A7RG-01	0.00	TCGA-TQ-A7RG-01	9.32	599.00	0.00	days	TCGA-TQ-A7RG	599.00	0.00	days	599.00	days	TCGA Lower Grade Glioma (LGG)	brain lower grade glioma	Brain					36.00	NO		NO	TCGA-TQ-A7RG-F65500	TCGA-TQ-A7RG	TCGA-TQ-A7RG-01A			(13227.00)	287.00		0.00	599.00		(32.00)		NO	NO	NO				Seizures	> 181 Days			NO		42054.00	MALE	NO		Oligoastrocytoma
488	TCGA-TQ-A7RH-01	0.00	TCGA-TQ-A7RH-01	11.09	486.00	0.00	days	TCGA-TQ-A7RH	486.00	0.00	days	486.00	days	TCGA Lower Grade Glioma (LGG)	brain lower grade glioma	Brain					39.00	NO		NO	TCGA-TQ-A7RH-F65502	TCGA-TQ-A7RH	TCGA-TQ-A7RH-01A			(14524.00)	167.00		0.00	486.00				NO	NO	NO				Seizures	> 181 Days			NO		41907.00	MALE	NO	YES	Oligoastrocytoma
489	TCGA-TQ-A7RI-01	0.00	TCGA-TQ-A7RI-01	9.57	62.00	0.00	days	TCGA-TQ-A7RI	62.00	0.00	days	62.00	days	TCGA Lower Grade Glioma (LGG)	brain lower grade glioma	Brain					37.00	NO		NO	TCGA-TQ-A7RI-F65497	TCGA-TQ-A7RI				(13799.00)			0.00	62.00				NO	NO	NO				Seizures	0 - 30 Days			NO		41907.00	FEMALE	NO	NO	Oligodendroglioma
490	TCGA-TQ-A7RJ-01	0.00	TCGA-TQ-A7RJ-01	9.79	1229.00	0.00	days	TCGA-TQ-A7RJ	1229.00	0.00	days	1229.00	days	TCGA Lower Grade Glioma (LGG)	brain lower grade glioma	Brain					25.00	NO		NO	TCGA-TQ-A7RJ-F65494	TCGA-TQ-A7RJ	TCGA-TQ-A7RJ-01A			(9209.00)	924.00		0.00	1229.00				NO	NO	NO				Sensory Changes	> 181 Days			NO		41907.00	FEMALE	NO	NO	Oligoastrocytoma
491	TCGA-TQ-A7RK-01	0.00	TCGA-TQ-A7RK-01	9.07	1341.00	0.00	days	TCGA-TQ-A7RK	858.00	1.00	days	1341.00	days	TCGA Lower Grade Glioma (LGG)	brain lower grade glioma	Brain	YES	YES	YES		29.00	NO		NO	TCGA-TQ-A7RK-F61519	TCGA-TQ-A7RK	TCGA-TQ-A7RK-01A	882.00		(10742.00)	1057.00		0.00	1341.00	858.00	(115.00)	0.00	NO	NO	NO				Mental Status Changes	0 - 30 Days	Additional New Tumor Event	Complete Remission/Response	NO		41820.00	MALE	NO	NO	Oligoastrocytoma
492	TCGA-TQ-A7RK-02	0.00	TCGA-TQ-A7RK-02	9.66	1341.00	0.00	days	TCGA-TQ-A7RK	858.00	1.00	days	1341.00	days	TCGA Lower Grade Glioma (LGG)	brain lower grade glioma	Brain	YES	YES	YES		29.00	NO		NO	TCGA-TQ-A7RK-F61519	TCGA-TQ-A7RK	TCGA-TQ-A7RK-02B	882.00		(10742.00)	1131.00		0.00	1341.00	858.00	(115.00)	0.00	NO	NO	NO				Mental Status Changes	0 - 30 Days	Additional New Tumor Event	Complete Remission/Response	NO		41820.00	MALE	NO	NO	Oligoastrocytoma
493	TCGA-TQ-A7RM-01	0.00	TCGA-TQ-A7RM-01	11.43	1116.00	0.00	days	TCGA-TQ-A7RM	1116.00	0.00	days	1116.00	days	TCGA Lower Grade Glioma (LGG)	brain lower grade glioma	Brain					41.00	NO		NO	TCGA-TQ-A7RM-F65552	TCGA-TQ-A7RM				(15154.00)			0.00	1116.00		45.00	0.00	NO	NO	NO				Seizures	> 181 Days	Scheduled Follow-up Submission	Partial Remission/Response	NO		42052.00	FEMALE	NO	NO	Oligoastrocytoma
494	TCGA-TQ-A7RN-01	0.00	TCGA-TQ-A7RN-01	10.44	1026.00	0.00	days	TCGA-TQ-A7RN	1026.00	0.00	days	1026.00	days	TCGA Lower Grade Glioma (LGG)	brain lower grade glioma	Brain					32.00	NO		NO	TCGA-TQ-A7RN-F65559	TCGA-TQ-A7RN	TCGA-TQ-A7RN-01A			(11823.00)	714.00		0.00	1026.00		11.00	0.00	NO	NO	NO				Headaches	> 181 Days	Scheduled Follow-up Submission	Partial Remission/Response	NO		42052.00	MALE	NO	YES	Oligodendroglioma
495	TCGA-TQ-A7RO-01	0.00	TCGA-TQ-A7RO-01	10.07	949.00	0.00	days	TCGA-TQ-A7RO	949.00	0.00	days	949.00	days	TCGA Lower Grade Glioma (LGG)	brain lower grade glioma	Brain					29.00	NO		NO	TCGA-TQ-A7RO-F65562	TCGA-TQ-A7RO	TCGA-TQ-A7RO-01A			(10890.00)	665.00		0.00	949.00		123.00	0.00	NO	YES	NO			12 - 20 Years	Seizures	31 - 90 Days	Scheduled Follow-up Submission	Partial Remission/Response	YES	Seafood	42052.00	MALE	NO	NO	Oligoastrocytoma
496	TCGA-TQ-A7RP-01	0.00	TCGA-TQ-A7RP-01	12.27	914.00	0.00	days	TCGA-TQ-A7RP	914.00	0.00	days	914.00	days	TCGA Lower Grade Glioma (LGG)	brain lower grade glioma	Brain					66.00	NO		NO	TCGA-TQ-A7RP-F65564	TCGA-TQ-A7RP	TCGA-TQ-A7RP-01A			(24109.00)	553.00		0.00	914.00		152.00	0.00	NO	NO	NO				Seizures	> 181 Days	Scheduled Follow-up Submission	Partial Remission/Response	NO		42052.00	MALE	NO	NO	Oligoastrocytoma
497	TCGA-TQ-A7RQ-01	0.00	TCGA-TQ-A7RQ-01	7.83	795.00	0.00	days	TCGA-TQ-A7RQ	795.00	0.00	days	795.00	days	TCGA Lower Grade Glioma (LGG)	brain lower grade glioma	Brain					38.00	NO		NO	TCGA-TQ-A7RQ-F65566	TCGA-TQ-A7RQ	TCGA-TQ-A7RQ-01A			(14187.00)	504.00		0.00	795.00		53.00	0.00	NO						Seizures	91 - 180 Days	Scheduled Follow-up Submission	Complete Remission/Response	NO		41942.00	FEMALE	NO	NO	Oligodendroglioma
498	TCGA-TQ-A7RR-01	0.00	TCGA-TQ-A7RR-01	9.73	787.00	0.00	days	TCGA-TQ-A7RR	787.00	0.00	days	787.00	days	TCGA Lower Grade Glioma (LGG)	brain lower grade glioma	Brain					38.00	NO		NO	TCGA-TQ-A7RR-F65569	TCGA-TQ-A7RR	TCGA-TQ-A7RR-01A			(14022.00)	454.00		0.00	787.00		31.00	0.00	NO	NO	NO				Seizures	> 181 Days	Scheduled Follow-up Submission	Partial Remission/Response	NO		41942.00	MALE	NO	NO	Oligoastrocytoma
499	TCGA-TQ-A7RU-01	0.00	TCGA-TQ-A7RU-01	10.52	1032.00	0.00	days	TCGA-TQ-A7RU	1032.00	0.00	days	1032.00	days	TCGA Lower Grade Glioma (LGG)	brain lower grade glioma	Brain					51.00	NO		NO	TCGA-TQ-A7RU-F65649	TCGA-TQ-A7RU				(18990.00)			0.00	1032.00		158.00	0.00	NO	NO	NO				Seizures	> 181 Days	Scheduled Follow-up Submission	Partial Remission/Response	NO		41942.00	MALE	NO	YES	Oligodendroglioma
500	TCGA-TQ-A7RV-01	0.00	TCGA-TQ-A7RV-01	10.36	1868.00	0.00	days	TCGA-TQ-A7RV	530.00	1.00	days	1868.00	days	TCGA Lower Grade Glioma (LGG)	brain lower grade glioma	Brain	YES	YES			27.00	NO		NO	TCGA-TQ-A7RV-F61501	TCGA-TQ-A7RV	TCGA-TQ-A7RV-01A		1141.00	(10167.00)	1470.00		0.00	1868.00	530.00	(42.00)	1.00	NO	NO	NO				Sensory Changes	> 181 Days	Scheduled Follow-up Submission	Progressive Disease	NO		41919.00	MALE	NO	YES	Astrocytoma
501	TCGA-TQ-A7RV-02	0.00	TCGA-TQ-A7RV-02	11.04	1868.00	0.00	days	TCGA-TQ-A7RV	530.00	1.00	days	1868.00	days	TCGA Lower Grade Glioma (LGG)	brain lower grade glioma	Brain	YES	YES			27.00	NO		NO	TCGA-TQ-A7RV-F61501	TCGA-TQ-A7RV	TCGA-TQ-A7RV-02A		1141.00	(10167.00)	1544.00		0.00	1868.00	530.00	(42.00)	1.00	NO	NO	NO				Sensory Changes	> 181 Days	Scheduled Follow-up Submission	Progressive Disease	NO		41919.00	MALE	NO	YES	Astrocytoma
502	TCGA-TQ-A7RW-01	1.00	TCGA-TQ-A7RW-01	9.66	821.00	1.00	days	TCGA-TQ-A7RW	511.00	1.00	days	821.00	days	TCGA Lower Grade Glioma (LGG)	brain lower grade glioma	Brain	YES	YES	YES		32.00	NO		NO	TCGA-TQ-A7RW-F65651	TCGA-TQ-A7RW	TCGA-TQ-A7RW-01A	[Discrepancy]		(11804.00)	1022.00	821.00	0.00		511.00	529.00	0.00	NO	NO	NO				Seizures	31 - 90 Days	Scheduled Follow-up Submission	Progressive Disease	NO		41908.00	MALE	NO	NO	Oligodendroglioma

503	TCGA-TQ-A8XE-01	1.00	TCGA-TQ-A8XE-01	8.77	954.00	1.00	days	TCGA-TQ-A8XE	455.00	1.00	days	954.00	days	TCGA Lower Grade Glioma (LGG)	brain lower grade glioma	Brain	YES	YES	NO		42.00	NO		NO	TCGA-TQ-A8XE-F61509	TCGA-TQ-A8XE	TCGA-TQ-A8XE-01A			(15629.00)	1308.00	954.00	0.00		455.00	0.00	1.00	NO	NO	NO				Headaches	91 - 180 Days	Additional New Tumor Event	Progressive Disease			41820.00	FEMALE	NO	YES	Oligodendroglioma
504	TCGA-TQ-A8XE-02	1.00	TCGA-TQ-A8XE-02	10.59	954.00	1.00	days	TCGA-TQ-A8XE	455.00	1.00	days	954.00	days	TCGA Lower Grade Glioma (LGG)	brain lower grade glioma	Brain	YES	YES	NO		42.00	NO		NO	TCGA-TQ-A8XE-F61509	TCGA-TQ-A8XE	TCGA-TQ-A8XE-02A			(15629.00)	1308.00	954.00	0.00		455.00	0.00	1.00	NO	NO	NO				Headaches	91 - 180 Days	Additional New Tumor Event	Progressive Disease			41820.00	FEMALE	NO	YES	Oligodendroglioma
505	TCGA-VM-A8C8-01	0.00	TCGA-VM-A8C8-01	8.72	1397.00	0.00	days	TCGA-VM-A8C8	1397.00	0.00	days	1397.00	days	TCGA Lower Grade Glioma (LGG)	brain lower grade glioma	Brain	NO	NO			50.00				TCGA-VM-A8C8-F69643	TCGA-VM-A8C8	TCGA-VM-A8C8-01A			(18494.00)	959.00		0.00	1397.00		19.00			YES	NO				Motor/Movement Changes	> 181 Days	Scheduled Follow-up Submission				42019.00	FEMALE	YES	NO	Oligodendroglioma
506	TCGA-VM-A8C9-01	0.00	TCGA-VM-A8C9-01	11.04	1314.00	0.00	days	TCGA-VM-A8C9	1314.00	0.00	days	1314.00	days	TCGA Lower Grade Glioma (LGG)	brain lower grade glioma	Brain	NO	NO			37.00				TCGA-VM-A8C9-F70437	TCGA-VM-A8C9	TCGA-VM-A8C9-01A			(13663.00)	847.00		0.00	1314.00					NO	NO				Seizures	> 181 Days	Scheduled Follow-up Submission	Complete Remission/Response			42051.00	FEMALE		YES	Astrocytoma
507	TCGA-VM-A8CA-01	0.00	TCGA-VM-A8CA-01	9.64	411.00	0.00	days	TCGA-VM-A8CA			days	411.00	days	TCGA Lower Grade Glioma (LGG)	brain lower grade glioma	Brain					54.00			NO	TCGA-VM-A8CA-F63647	TCGA-VM-A8CA	TCGA-VM-A8CA-01A			(19991.00)	798.00		0.00	411.00		19.00			NO	NO				Mental Status Changes	0 - 30 Days	Scheduled Follow-up Submission				41936.00	MALE		NO	Oligodendroglioma
508	TCGA-VM-A8CB-01	0.00	TCGA-VM-A8CB-01	9.11	3.00	0.00	days	TCGA-VM-A8CB			days	3.00	days	TCGA Lower Grade Glioma (LGG)	brain lower grade glioma	Brain					33.00				TCGA-VM-A8CB-F66847	TCGA-VM-A8CB	TCGA-VM-A8CB-01A			(12340.00)	738.00		0.00	3.00					NO	NO				Seizures	0 - 30 Days	Scheduled Follow-up Submission				41936.00	MALE		YES	Oligodendroglioma
509	TCGA-VM-A8CD-01	1.00	TCGA-VM-A8CD-01	8.25	240.00	1.00	days	TCGA-VM-A8CD	240.00	0.00	days	240.00	days	TCGA Lower Grade Glioma (LGG)	brain lower grade glioma	Brain	NO	NO			58.00			NO	TCGA-VM-A8CD-F66848	TCGA-VM-A8CD	TCGA-VM-A8CD-01A			(21200.00)	517.00	240.00	0.00			(43.00)			YES	NO				Seizures	> 181 Days	Scheduled Follow-up Submission				41936.00	MALE		YES	Astrocytoma
510	TCGA-VM-A8CE-01	0.00	TCGA-VM-A8CE-01	10.37	1191.00	0.00	days	TCGA-VM-A8CE	1191.00	0.00	days	1191.00	days	TCGA Lower Grade Glioma (LGG)	brain lower grade glioma	Brain	NO	NO			25.00			NO	TCGA-VM-A8CE-F69636	TCGA-VM-A8CE	TCGA-VM-A8CE-01A			(9363.00)	780.00		0.00	1191.00		15.00			NO	NO				Seizures	0 - 30 Days	Scheduled Follow-up Submission				42019.00	MALE		NO	Oligodendroglioma
511	TCGA-VM-A8CF-01	0.00	TCGA-VM-A8CF-01	9.63	609.00	0.00	days	TCGA-VM-A8CF	609.00	0.00	days	609.00	days	TCGA Lower Grade Glioma (LGG)	brain lower grade glioma	Brain					44.00				TCGA-VM-A8CF-F66857	TCGA-VM-A8CF	TCGA-VM-A8CF-01A			(16334.00)	280.00		0.00	609.00		26.00			NO	NO				Motor/Movement Changes	> 181 Days	Scheduled Follow-up Submission	Stable Disease			41936.00	FEMALE		NO	Astrocytoma
512	TCGA-VM-A8CH-01	0.00	TCGA-VM-A8CH-01	9.80	714.00	0.00	days	TCGA-VM-A8CH	714.00	0.00	days	714.00	days	TCGA Lower Grade Glioma (LGG)	brain lower grade glioma	Brain					24.00				TCGA-VM-A8CH-F69641	TCGA-VM-A8CH	TCGA-VM-A8CH-01A			(9005.00)	300.00		0.00	714.00		(38.00)			YES	NO				Seizures	> 181 Days	Scheduled Follow-up Submission				42019.00	FEMALE		YES	Astrocytoma
513	TCGA-VV-A829-01	0.00	TCGA-VV-A829-01	10.30	1127.00	0.00	days	TCGA-VV-A829	1127.00	0.00	days	1127.00	days	TCGA Lower Grade Glioma (LGG)	brain lower grade glioma	Brain					44.00	NO		NO	TCGA-VV-A829-F65521	TCGA-VV-A829	TCGA-VV-A829-01A			(16163.00)	720.00		0.00	1127.00					YES	NO				Motor/Movement Changes	0 - 30 Days	Scheduled Follow-up Submission	Complete Remission/Response	NO		41914.00	MALE	NO	NO	Oligoastrocytoma
514	TCGA-VV-A86M-01	0.00	TCGA-VV-A86M-01	9.42	487.00	0.00	days	TCGA-VV-A86M	487.00	0.00	days	487.00	days	TCGA Lower Grade Glioma (LGG)	brain lower grade glioma	Brain					36.00	NO		NO	TCGA-VV-A86M-F66128	TCGA-VV-A86M	TCGA-VV-A86M-01A			(13453.00)	111.00		0.00	487.00										Sensory Changes	> 181 Days	Scheduled Follow-up Submission	Complete Remission/Response	NO		41914.00	FEMALE	NO	NO	Astrocytoma
515	TCGA-VW-A7QS-01	0.00	TCGA-VW-A7QS-01	11.96	706.00	0.00	days	TCGA-VW-A7QS	706.00	0.00	days	706.00	days	TCGA Lower Grade Glioma (LGG)	brain lower grade glioma	Brain					35.00	NO		NO		TCGA-VW-A7QS	TCGA-VW-A7QS-01A			(12820.00)	805.00		0.00	706.00				NO	NO	NO				Headaches	31 - 90 Days			NO		41620.00	FEMALE	NO	YES	Oligodendroglioma
516	TCGA-VW-A8FI-01	1.00	TCGA-VW-A8FI-01	9.67	245.00	1.00	days	TCGA-VW-A8FI	245.00	0.00	days	245.00	days	TCGA Lower Grade Glioma (LGG)	brain lower grade glioma	Brain					66.00	NO		NO		TCGA-VW-A8FI	TCGA-VW-A8FI-01A			(24411.00)	154.00	245.00	0.00					NO	NO	NO				Mental Status Changes	31 - 90 Days			NO		41708.00	MALE	NO	NO	Astrocytoma
517	TCGA-W9-A837-01	0.00	TCGA-W9-A837-01	9.46	1553.00	0.00	days	TCGA-W9-A837	1553.00	0.00	days	1553.00	days	TCGA Lower Grade Glioma (LGG)	brain lower grade glioma	Brain					47.00			NO	TCGA-W9-A837-F66176	TCGA-W9-A837	TCGA-W9-A837-01A				1298.00		0.00	1553.00		610.00	1.00	NO	NO	NO				Seizures	> 181 Days	Scheduled Follow-up Submission	Stable Disease			41915.00	MALE	NO	NO	Oligodendroglioma
518	TCGA-WH-A86K-01	0.00	TCGA-WH-A86K-01	10.08	405.00	0.00	days	TCGA-WH-A86K	405.00	0.00	days	405.00	days	TCGA Lower Grade Glioma (LGG)	brain lower grade glioma	Brain					65.00	NO		NO	TCGA-WH-A86K-F67062	TCGA-WH-A86K	TCGA-WH-A86K-01A			(24055.00)	112.00		0.00	405.00		20.00	0.00	NO	NO	NO				Motor/Movement Changes	0 - 30 Days	Scheduled Follow-up Submission	Stable Disease	NO		41947.00	MALE	NO	NO	Astrocytoma
519	TCGA-WY-A858-01	0.00	TCGA-WY-A858-01	11.35	1337.00	0.00	days	TCGA-WY-A858	1337.00	0.00	days	1337.00	days	TCGA Lower Grade Glioma (LGG)	brain lower grade glioma	Brain					32.00			NO		TCGA-WY-A858				(11855.00)			0.00	1337.00		45.00	0.00		NO	NO				Headaches	0 - 30 Days			YES	fresh pitted fruit	41680.00	FEMALE	YES	YES	Astrocytoma
520	TCGA-WY-A859-01	0.00	TCGA-WY-A859-01	9.17	1213.00	0.00	days	TCGA-WY-A859	1213.00	0.00	days	1213.00	days	TCGA Lower Grade Glioma (LGG)	brain lower grade glioma	Brain					34.00	NO		NO		TCGA-WY-A859				(12631.00)			0.00	1213.00		293.00	0.00		NO	NO				Seizures	0 - 30 Days			NO		41690.00	FEMALE	NO	NO	Astrocytoma
521	TCGA-WY-A85A-01	0.00	TCGA-WY-A85A-01	9.77	1320.00	0.00	days	TCGA-WY-A85A	1320.00	0.00	days	1320.00	days	TCGA Lower Grade Glioma (LGG)	brain lower grade glioma	Brain					20.00	NO		NO		TCGA-WY-A85A	TCGA-WY-A85A-01A			(7380.00)	1203.00		0.00	1320.00		718.00	0.00	NO	NO	NO				Seizures	0 - 30 Days			NO		41697.00	MALE	NO	NO	Astrocytoma
522	TCGA-WY-A85B-01	0.00	TCGA-WY-A85B-01	9.18	1393.00	0.00	days	TCGA-WY-A85B	1393.00	0.00	days	1393.00	days	TCGA Lower Grade Glioma (LGG)	brain lower grade glioma	Brain					24.00	NO		NO	TCGA-WY-A85B-F65769	TCGA-WY-A85B	TCGA-WY-A85B-01A			(8817.00)	1073.00		0.00	1393.00			0.00		YES	NO				Seizures	0 - 30 Days	Scheduled Follow-up Submission		NO		42026.00	MALE	NO	YES	Astrocytoma
523	TCGA-WY-A85C-01	0.00	TCGA-WY-A85C-01	9.06	1426.00	0.00	days	TCGA-WY-A85C	1261.00	1.00	days	1426.00	days	TCGA Lower Grade Glioma (LGG)	brain lower grade glioma	Brain			YES		36.00	NO		NO	TCGA-WY-A85C-F65786	TCGA-WY-A85C	TCGA-WY-A85C-01A	1337.00		(13506.00)	1119.00		0.00	1426.00	1261.00		1.00		NO	NO				Headaches	0 - 30 Days	Scheduled Follow-up Submission	Progressive Disease	NO		42026.00	MALE	NO	YES	Astrocytoma
524	TCGA-WY-A85D-01	0.00	TCGA-WY-A85D-01	10.06	1147.00	0.00	days	TCGA-WY-A85D			days	1147.00	days	TCGA Lower Grade Glioma (LGG)	brain lower grade glioma	Brain					60.00	NO		NO		TCGA-WY-A85D	TCGA-WY-A85D-01A			(21979.00)	1035.00		0.00	1147.00			1.00	NO	YES	NO				Sensory Changes	> 181 Days			NO		41696.00	MALE	NO	NO	Oligoastrocytoma
525	TCGA-WY-A85E-01	0.00	TCGA-WY-A85E-01	8.74	633.00	0.00	days	TCGA-WY-A85E	532.00	1.00	days	633.00	days	TCGA Lower Grade Glioma (LGG)	brain lower grade glioma	Brain	YES	YES	YES		48.00	NO		NO	TCGA-WY-A85E-F65805	TCGA-WY-A85E	TCGA-WY-A85E-01A	536.00		(17878.00)	794.00		0.00	633.00	532.00	311.00	1.00	NO		NO				Seizures	31 - 90 Days	Scheduled Follow-up Submission	Progressive Disease	YES	seafood, nuts	42026.00	FEMALE	NO	NO	Oligoastrocytoma

No.	history_ionizing_rt_to_head	history_of_neoadjuvant_treatment	icd_10	icd_o_3_histology	icd_o_3_site	informed_consent_verified	inherited_genetic_syndrome_found	inherited_genetic_syndrome_result	initial_weight	intermediate_dimension	is_ffpe	karnofsky_performance_score	laterality	ldh1_mutation_found	ldh1_mutation_test_method	ldh1_mutation_tested	longest_dimension	lost_follow_up	mental_status_changes	mold_or_dust_allergy_history	motor_movement_changes	neoplasm_histologic_grade	new_tumor_event_after_initial_treatment	oct_embedded	other_dx	pathology_report_file_name	patient_id	performance_status_scale_timing
1	NO	No	C71.9	36586.00	C71.9	YES				0.60	NO	90.00	Right			NO	1.50	NO	NO	NO	NO	G2			No	TCGA-CS-4938.36632fe7-c93d-4cad-bd58-	4938.00	Preoperative
2	NO	No	C71.9	43891.00	C71.9	YES	NO					90.00	Right			NO				NO		G3	YES		No		4941.00	Post-Adjuvant Therapy
3	NO	No	C71.9	43891.00	C71.9	YES	NO			0.40	NO	70.00	Right			NO	0.50		YES	NO	YES	G3	YES		No	TCGA-CS-4942.052c9ff3-16ad-4219-8839-	4942.00	Preoperative
4	NO	No	C71.9	43891.00	C71.9	YES				0.50	NO	50.00	Left			NO	0.60	NO				G3			No	TCGA-CS-4943.c7cbbdb2-8ec4-4e6e-836e-	4943.00	Preoperative
5	NO	No	C71.9	36586.00	C71.9	YES	NO			0.70	NO	90.00	Right			NO	1.00	NO	YES		NO	G2	NO		No	TCGA-CS-4944.6845998b-b702-4f92-85bb-	4944.00	Preoperative
6	YES	No	C71.9	43891.00	C71.9	YES				1.00	NO	100.00	Left			NO	1.60		NO		NO	G3	NO		No	TCGA-CS-5393.760b381f-f65d-4a68-a1e6-	5393.00	Post-Adjuvant Therapy
7	NO	No	C71.9	43891.00	C71.9	YES				0.70	NO		Left				1.20	YES	NO	NO	NO	G3			No	TCGA-CS-5394.e2716cb5-e02c-4867-ba99-	5394.00	
8	NO	No	C71.9	30011.00	C71.9	YES				1.00	NO	80.00	Right			NO	1.50					G2	YES		No	TCGA-CS-5395.509c2da8-c6cf-4803-a643-	5395.00	Preoperative
9	NO	No	C71.9	18688.00	C71.9	YES	NO			0.90	NO	90.00	Right			NO	1.60	NO	NO		NO	G3			No	TCGA-CS-5396.7ae9d388-1145-4549-91fe-	5396.00	Preoperative
10	NO	No	C71.9	43891.00	C71.9	YES				0.60	NO	80.00	Left			NO	1.40		NO	NO	YES	G3	NO		No	TCGA-CS-5397.7082a52b-e688-4642-9bcc-	5397.00	Preoperative
11	NO	No	C71.9	30011.00	C71.9	YES	NO			0.70	NO	80.00	Right			NO	1.00		NO	NO	NO	G3	YES		No	TCGA-CS-6186.ae1d6c04-b80b-4da5-9c19-	6186.00	Post-Adjuvant Therapy
12	NO	No	C71.9	43891.00	C71.9	YES				0.80	NO	90.00	Right			NO	1.20	NO	NO	NO	NO	G3			No	TCGA-CS-6188.e85d2ddf-98e1-4a02-bde1-	6188.00	Preoperative
13	NO	No	C71.9	43891.00	C71.9	YES				0.40	NO	90.00	Left			NO	0.40	NO	NO	NO	NO	G3			No	TCGA-CS-6290.4d5bdf18-7799-463d-a33a-	6290.00	Preoperative
14	NO	No	C71.9	43891.00	C71.9	YES				0.80	NO	90.00	Right			NO	0.80	NO	NO	NO	NO	G3			Yes	TCGA-CS-6665.733dd970-ddcd-40d4-903d-	6665.00	Preoperative
15	NO	No	C71.9	43891.00	C71.9	YES				0.90	NO	90.00	Right			NO	1.80	NO	NO	NO	NO	G3	NO		No	TCGA-CS-6666.9bc50bcf-b22b-4516-b8a9-	6666.00	Preoperative
16	NO	No	C71.9	36586.00	C71.9	YES				0.60	NO	90.00	Left			NO	0.60	NO	NO	YES	YES	G2			No	TCGA-CS-6667.18c7ebd7-0bdc-42e2-97a7-	6667.00	Preoperative
17	NO	No	C71.9	18323.00	C71.9	YES				0.60	NO	90.00	Left			NO	1.00	NO	NO	NO	YES	G2			No	TCGA-CS-6668.d5604d22-f9c7-4eb3-af92-	6668.00	Preoperative
18	NO	No	C71.9	18323.00	C71.9	YES				0.40	NO	90.00	Right			NO	1.30	NO	NO	NO	NO	G2			No	TCGA-CS-6669.4d8abb1c-f9a3-4e9b-8140-	6669.00	Preoperative
19	NO	Yes	C71.9	18688.00	C71.9	YES				0.80	NO		Right			NO	1.00	NO	NO	NO	NO	G3			Yes	TCGA-CS-6670.925aeb6b-7ed0-40b5-b2bb-	6670.00	
20	NO	No	C71.0	30011.00	C71.0	YES	NO			1.00	NO		Left			NO	1.00	NO	NO	NO	NO	G3	NO		No	TCGA-DB-5270.dd42d675-2630-4ae6-9f3b-	5270.00	
21	NO	No	C71.0	43891.00	C71.0	YES	NO			1.00	NO		Right			NO	1.50	NO	NO	NO	YES	G3	NO		No	TCGA-DB-5273.a415e3dd-3b4f-4e69-b1c8-	5273.00	
22	NO	No	C71.0	30011.00	C71.0	YES	NO			1.60	NO	80.00	Left				1.70	NO	NO	NO	NO	G3	NO		No	TCGA-DB-5274.e287cee7-9711-450c-817e-	5274.00	Preoperative
23	NO	No	C71.0	30011.00	C71.0	YES	NO			1.00	NO		Right				1.70	YES	YES	NO	NO	G3			No	TCGA-DB-5275.a034b295-7764-4732-94ea-	5275.00	
24	NO	No	C71.0	30011.00	C71.0	YES	NO			1.30	NO		Left				1.40	NO	NO	NO	NO	G3	NO		No	TCGA-DB-5276.b1f076d6-2b5c-43b9-8240-	5276.00	
25	NO	No	C71.0	43891.00	C71.0	YES	NO			1.00	NO		Left				1.50	NO	NO	YES	NO	G3	YES		No	TCGA-DB-5277.5dd3f674-5ffa-45ce-98fd-	5277.00	
26	NO	No	C71.0	18323.00	C71.0	YES	NO			1.30	NO		Left				1.80	NO	YES	NO	YES	G2	NO		No	TCGA-DB-5278.b9380181-eb0c-458b-b0c4-	5278.00	
27	NO	No	C71.0	18323.00	C71.0	YES	NO			1.00	NO		Right				1.30	YES	NO	NO	NO	G2			No	TCGA-DB-5279.74052ac9-36de-45e7-8716-	5279.00	
28	NO	No	C71.0	30011.00	C71.0	YES	NO			1.50	NO		Right				1.60	NO	NO	NO	NO	G2	NO		No	TCGA-DB-5280.9592281f-2fae-4376-9f64-	5280.00	
29	NO	No	C71.0	30011.00	C71.0	YES	NO			1.00	NO		Left			NO	2.00	NO	YES	NO	NO	G3	NO		No	TCGA-DB-5281.40462852-7dc3-4a3f-a4f3-	5281.00	
30	NO	No	C71.2	30011.00	C71.2	YES	NO		460.00		NO		Left	YES	IHC	YES		NO	NO	NO	NO	G2	NO	TRUE	No	TCGA-DB-A4X9.8C541033-3F59-4F35-9BA8-	A4X9	
31	NO	No	C71.2	30011.00	C71.2	YES	NO		140.00		NO		Left	YES	IHC	YES		YES	NO	NO	NO	G2	NO	TRUE	No	TCGA-DB-A4XA.8DCE6400-9767-46DD-A965-	A4XA	
32	NO	No	C71.4	43891.00	C71.4	YES	NO		120.00		NO		Left	YES	IHC	YES		NO	NO		NO	G3	NO	TRUE	No	TCGA-DB-A4XB.EAFB98CA-3206-4193-9FA9-	A4XB	
33	NO	No	C71.2	30011.00	C71.2	YES	NO		230.00		NO		Left			NO		NO	NO		NO	G2	NO	TRUE	Yes, History of Synchronous/ Bilateral	TCGA-DB-A4XC.26BC05CE-7A4F-4E5C-84A5-	A4XC	
34	NO	No	C71.2	43891.00	C71.2	YES	NO		280.00		NO		Right			NO		NO	NO		NO	G3	NO	TRUE	No	TCGA-DB-A4XD.5D9BCE5F-0490-4504-B5A2-	A4XD	

No.	person_neoplasm_cancer_status	preoperative_antiseizure_meds	preoperative_corticosteroids	primary_therapy_outcome_success	radiation_therapy	sample_type	sample_type_id	seizure_history	sensory_changes	shortest_dimension	supratentorial_localization	targeted_molecular_therapy	tissue_prospective_collection_indicator	tissue_retrospective_collection_indicator	tissue_source_site	tumor_location	tumor_tissue_site	vial_number	visual_changes	vital_status	year_of_initial_pathologic_diagnosis
1	WITH TUMOR	NO	NO			Primary Tumor	1.00	YES	NO	0.30	Cerebral Cortex		NO	YES	CS	Supratentorial, Frontal Lobe	Central nervous system	B	NO	LIVING	2005.00
2	WITH TUMOR			Stable Disease	YES	Primary Tumor	1.00				Cerebral Cortex	YES	NO	YES	CS	Supratentorial, Frontal Lobe	Central nervous system			DECEASED	2005.00
3	WITH TUMOR	NO	NO	Progressive Disease	YES	Primary Tumor	1.00	YES	YES	0.40	White Matter	NO	NO	YES	CS	Supratentorial, Frontal Lobe	Central nervous system	A	NO	DECEASED	2006.00
4	WITH TUMOR	YES				Primary Tumor	1.00	YES		0.10	Cerebral Cortex		NO	YES	CS	Supratentorial, Frontal Lobe	Central nervous system	A		DECEASED	2009.00
5	WITH TUMOR					Primary Tumor	1.00		NO	0.30	Cerebral Cortex		NO	YES	CS	Supratentorial, Temporal Lobe	Central nervous system	A	NO	LIVING	2010.00
6	TUMOR FREE	NO		Complete Remission/Response	YES	Primary Tumor	1.00	NO	NO	0.30	Cerebral Cortex	YES	NO	YES	CS	Supratentorial, Temporal Lobe	Central nervous system	A	NO	LIVING	2008.00
7		YES	YES			Primary Tumor	1.00	YES	NO	0.60	Cerebral Cortex		NO	YES	CS	Supratentorial, Frontal Lobe	Central nervous system	A	NO	LIVING	2005.00
8	WITH TUMOR			Progressive Disease	YES	Primary Tumor	1.00			0.10	Cerebral Cortex	YES	NO	YES	CS	Supratentorial, Parietal Lobe	Central nervous system	A		DECEASED	2007.00
9	WITH TUMOR			[Discrepancy]		Primary Tumor	1.00	NO		0.80	Cerebral Cortex		NO	YES	CS	Supratentorial, Frontal Lobe	Central nervous system	A		LIVING	2010.00
10	WITH TUMOR	NO	NO	Progressive Disease	YES	Primary Tumor	1.00	NO	YES	0.50	Cerebral Cortex	YES	NO	YES	CS	Supratentorial, Temporal Lobe	Central nervous system	A	NO	DECEASED	2010.00
11	WITH TUMOR	NO	NO	Progressive Disease	YES	Primary Tumor	1.00	YES	NO	0.30	Cerebral Cortex	YES	NO	YES	CS	Supratentorial, Frontal Lobe	Central nervous system	A	NO	DECEASED	2009.00
12	WITH TUMOR	NO	NO			Primary Tumor	1.00	YES	NO	0.40	Cerebral Cortex		NO	YES	CS	Supratentorial, Temporal Lobe	Central nervous system	A	NO	DECEASED	2009.00
13		NO	NO			Primary Tumor	1.00	YES	NO	0.40	Cerebral Cortex		NO	YES	CS	Supratentorial, Temporal Lobe	Central nervous system	A	NO	DECEASED	2009.00
14	WITH TUMOR	NO	NO	Stable Disease		Primary Tumor	1.00	YES	NO	0.20	Cerebral Cortex		NO	YES	CS	Supratentorial, Temporal Lobe	Central nervous system	A	NO	LIVING	2010.00
15	TUMOR FREE	NO	NO	Complete Remission/Response		Primary Tumor	1.00	NO	NO	0.40	Cerebral Cortex		NO	YES	CS	Supratentorial, Parietal Lobe	Central nervous system	A	NO	LIVING	2010.00
16	WITH TUMOR	NO	NO	Stable Disease		Primary Tumor	1.00	NO	YES	0.20	Cerebral Cortex		NO	YES	CS	Supratentorial, Parietal Lobe	Central nervous system	A	NO	LIVING	2010.00
17	WITH TUMOR	NO	NO	Stable Disease		Primary Tumor	1.00	YES	NO	0.50	Cerebral Cortex		NO	YES	CS	Supratentorial, Frontal Lobe	Central nervous system	A	NO	LIVING	2011.00
18	WITH TUMOR	NO	NO	Stable Disease		Primary Tumor	1.00	YES	NO	0.20	Cerebral Cortex		NO	YES	CS	Supratentorial, Temporal Lobe	Central nervous system	A	NO	LIVING	2011.00
19	WITH TUMOR	NO	NO	[Discrepancy]		Primary Tumor	1.00	NO	NO	0.30	Cerebral Cortex				CS	Supratentorial, Temporal Lobe	Central nervous system	A	NO	LIVING	2011.00
20	TUMOR FREE	YES	NO	Complete Remission/Response	NO	Primary Tumor	1.00	YES	NO	0.70	Cerebral Cortex	NO	NO	YES	DB	Supratentorial, Frontal Lobe	Central nervous system	A	NO	LIVING	2002.00
21	TUMOR FREE	YES	NO	Complete Remission/Response	YES	Primary Tumor	1.00	YES	NO	0.30	Cerebral Cortex	YES	NO	YES	DB	Supratentorial, Frontal Lobe	Central nervous system	A	NO	LIVING	2006.00
22	TUMOR FREE	YES	YES	Complete Remission/Response	YES	Primary Tumor	1.00	NO	NO	0.70	Cerebral Cortex	YES	NO	YES	DB	Supratentorial, Frontal Lobe	Central nervous system	A	NO	LIVING	2006.00
23	TUMOR FREE	NO	NO	Complete Remission/Response	YES	Primary Tumor	1.00	NO	NO	0.30	Cerebral Cortex	YES	NO	YES	DB	Supratentorial, Temporal Lobe	Central nervous system	A	NO	LIVING	2006.00
24	TUMOR FREE	YES	YES	Complete Remission/Response	YES	Primary Tumor	1.00	YES	NO	0.40	Cerebral Cortex	YES	NO	YES	DB	Supratentorial, Frontal Lobe	Central nervous system	A	NO	LIVING	2007.00
25	WITH TUMOR	YES	NO	Complete Remission/Response	YES	Primary Tumor	1.00	YES	NO	0.10	Cerebral Cortex	YES	NO	YES	DB	Supratentorial, Frontal Lobe	Central nervous system	A	NO	DECEASED	2007.00
26	WITH TUMOR	YES	NO	Stable Disease	NO	Primary Tumor	1.00	YES	NO	0.40	Cerebral Cortex	NO	NO	YES	DB	Supratentorial, Frontal Lobe	Central nervous system	A	NO	LIVING	2007.00
27	TUMOR FREE	YES	YES	Complete Remission/Response	NO	Primary Tumor	1.00	YES	NO	0.40	Cerebral Cortex	NO	NO	YES	DB	Supratentorial, Frontal Lobe	Central nervous system	A	NO	LIVING	2007.00
28	TUMOR FREE	YES	NO	Complete Remission/Response	NO	Primary Tumor	1.00	YES	NO	0.30	Cerebral Cortex	NO	NO	YES	DB	Supratentorial, Frontal Lobe	Central nervous system	A	NO	LIVING	2007.00
29	WITH TUMOR	YES	NO	Stable Disease	YES	Primary Tumor	1.00	YES	NO	0.50	Deep Gray (e.g.basal ganglia, thalamus)	YES	NO	YES	DB	Supratentorial, Frontal Lobe	Central nervous system	A	NO	LIVING	2006.00
30	TUMOR FREE	YES	NO	Complete Remission/Response	NO	Primary Tumor	1.00	YES	NO		Cerebral Cortex	NO	YES	NO	DB	Supratentorial, Temporal Lobe	Central nervous system	A	NO	LIVING	2010.00
31	WITH TUMOR	YES	NO	Stable Disease	YES	Primary Tumor	1.00	YES	NO		Cerebral Cortex	NO	YES	NO	DB	Supratentorial, Parietal Lobe	Central nervous system	A	NO	LIVING	2012.00
32	TUMOR FREE	YES	NO	Stable Disease	YES	Primary Tumor	1.00	YES	NO		Cerebral Cortex	YES	YES	NO	DB	Supratentorial, Parietal Lobe	Central nervous system	A	NO	LIVING	2011.00
33	TUMOR FREE			Partial Remission/Response	YES	Primary Tumor	1.00	YES	NO		Cerebral Cortex	NO	YES	NO	DB	Supratentorial, Temporal Lobe	Central nervous system	A	NO	LIVING	2010.00
34	TUMOR FREE	NO	NO	Complete Remission/Response	YES	Primary Tumor	1.00	NO	NO		Cerebral Cortex	YES	YES	NO	DB	Supratentorial, Temporal Lobe	Central nervous system	A	NO	LIVING	2010.00

35	NO	No	C71.2	30011.00	C71.2	YES	NO		170.00		NO	90.00	Left			NO		NO	NO		NO	G3	NO	TRUE	No	TCGA-DB-A4XE.1D110C27-320E-4C5D-A8E1-	A4XE	Preoperative	TUMOR FREE	YES	NO	Complete Remission/Response	YES	Primary Tumor	1.00	YES	NO		Cerebral Cortex	NO	YES	NO	DB	Supratentorial, Temporal Lobe	Central nervous system	A	NO	LIVING	2011.00
36	NO	No	C71.1	43891.00	C71.1	YES	NO		300.00		NO		Right			NO		NO	NO		NO	G3	NO	TRUE	No	TCGA-DB-A4XF.E9D4CC67-D5FC-4E81-91EB-	A4XF		TUMOR FREE	YES	NO	Stable Disease	YES	Primary Tumor	1.00	YES	NO		Cerebral Cortex	YES	YES	NO	DB	Supratentorial, Frontal Lobe	Central nervous system	A	NO	LIVING	2011.00
37	NO	No	C71.1	18688.00	C71.1	YES	NO		210.00		NO		Right	NO	IHC	YES		YES	NO	NO	NO	G3	NO	TRUE	No	TCGA-DB-A4XG.A0776539-C1FE-4798-9F65-	A4XG		WITH TUMOR	YES		Stable Disease	NO	Primary Tumor	1.00	YES	NO		Cerebral Cortex	YES	YES	NO	DB	Supratentorial, Frontal Lobe	Central nervous system	A	NO	LIVING	2006.00
38	NO	No	C71.1	30011.00	C71.1	YES	NO		160.00		NO		Left			NO		NO	NO		NO	G2	NO	TRUE	No	TCGA-DB-A4XH.5AD2C9F3-9A4A-4974-98F8-	A4XH		TUMOR FREE	YES	YES	Stable Disease	NO	Primary Tumor	1.00	YES	NO		Cerebral Cortex	NO	YES	NO	DB	Supratentorial, Frontal Lobe	Central nervous system	A	NO	LIVING	2011.00
39	NO	No	C71.0	18323.00	C71.0	YES	YES		150.00		NO	70.00	Right	YES	IHC	YES		NO	NO	NO	NO	G2	YES	TRUE	No	TCGA-DB-A64L.B8075A84-8641-4085-9B51-	A64L	Preoperative	WITH TUMOR	YES		Complete Remission/Response	NO	Primary Tumor	1.00	YES	NO		Cerebral Cortex	NO	YES	NO	DB	Supratentorial, Frontal Lobe	Central nervous system	A	NO	LIVING	2012.00
40	NO	No	C71.0	30011.00	C71.0	YES	NO		290.00		NO		Left	NO	IHC	YES		YES	NO		YES	G2	YES	TRUE	No	TCGA-DB-A64O.67251C0F-7DD0-4736-A614-	A64O		WITH TUMOR	YES	YES	Stable Disease	YES	Primary Tumor	1.00	NO	NO		Cerebral Cortex	NO	YES	NO	DB	Supratentorial, Temporal Lobe	Central nervous system	A	NO	DECEASED	2011.00
41	NO	No	C71.0	18688.00	C71.0	YES	NO		280.00		NO		Left	YES	IHC	YES		NO	NO		NO	G3	NO	TRUE	No	TCGA-DB-A64P.3D2EEE2E-B4EB-4603-B4D2-	A64P		TUMOR FREE	YES	YES	Complete Remission/Response	NO	Primary Tumor	1.00	YES	NO		Cerebral Cortex	YES	YES	NO	DB	Supratentorial, Frontal Lobe	Central nervous system	A	NO	LIVING	2012.00
42	NO	No	C71.0	30011.00	C71.0	YES	NO		200.00		NO	90.00	Left	YES	IHC	YES		YES	NO		NO	G2	NO	TRUE	No	TCGA-DB-A64Q.1650DAE4-352B-4BDD-A48E-	A64Q	Preoperative	TUMOR FREE	YES	NO	Stable Disease	NO	Primary Tumor	1.00	YES	NO		Cerebral Cortex	YES	YES	NO	DB	Supratentorial, Frontal Lobe	Central nervous system	A	NO	LIVING	2012.00
43	NO	No	C71.0	18323.00	C71.0	YES	NO		90.00		NO	90.00	Right	YES	IHC	YES		NO	NO		NO	G2	NO	TRUE	No	TCGA-DB-A64R.A9F5EDE4-1FC5-4E6D-BEA1-	A64R	Preoperative	TUMOR FREE	YES	NO	Complete Remission/Response	NO	Primary Tumor	1.00	YES	NO		Cerebral Cortex	NO	YES	NO	DB	Supratentorial, Frontal Lobe	Central nervous system	A	NO	LIVING	2012.00
44	NO	No	C71.0	30011.00	C71.0	YES	NO		220.00		NO	100.00				NO		NO	NO		NO	G2	NO	TRUE	No	TCGA-DB-A64S.31AB0B8C-9300-4508-B653-	A64S	Preoperative	TUMOR FREE	NO	NO	Complete Remission/Response	NO	Primary Tumor	1.00	NO	NO		Cerebral Cortex	NO	YES	NO	DB	Supratentorial, Frontal Lobe	Central nervous system	A	NO	LIVING	2012.00
45	NO	No	C71.0	30011.00	C71.0	YES	NO		340.00		NO	90.00	Right			NO		YES	NO	NO	NO	G2	NO	TRUE	No	TCGA-DB-A64U.7BE25474-4E80-403D-A999-	A64U	Preoperative	TUMOR FREE	YES	NO	Complete Remission/Response	NO	Primary Tumor	1.00	NO	NO		Cerebral Cortex	NO	YES	NO	DB	Supratentorial, Frontal Lobe	Central nervous system	A	NO	LIVING	2011.00
46	NO	No	C71.0	18323.00	C71.0	YES	NO		200.00		NO	90.00	Left			NO		NO	NO		NO	G2	NO	TRUE	No	TCGA-DB-A64V.9675813D-E747-4768-835C-	A64V	Preoperative	TUMOR FREE	YES	NO	Stable Disease	NO	Primary Tumor	1.00	YES	NO		Cerebral Cortex	YES	YES	NO	DB	Supratentorial, Frontal Lobe	Central nervous system	A	NO	LIVING	2012.00
47	NO	No	C71.0	30011.00	C71.0	YES	NO		100.00		NO	90.00	Right	YES	IHC	YES		NO	NO		NO	G3	NO	TRUE	No	TCGA-DB-A64W.9F52D767-A4C4-4D70-BB04-	A64W	Preoperative	TUMOR FREE	YES	YES	Stable Disease	YES	Primary Tumor	1.00	YES	NO		Cerebral Cortex	YES	YES	NO	DB	Supratentorial, Frontal Lobe	Central nervous system	A	NO	DECEASED	2012.00
48	NO	No	C71.0	43891.00	C71.0	YES	NO		280.00		NO	90.00	Right	YES	IHC	YES		NO	NO		YES	G3	NO	TRUE	No	TCGA-DB-A64X.2B5E76F9-FEC7-4238-AF98-	A64X	Preoperative	TUMOR FREE	NO	NO	Stable Disease	YES	Primary Tumor	1.00	NO	NO		Cerebral Cortex	YES	YES	NO	DB	Supratentorial, Frontal Lobe	Central nervous system	A	NO	LIVING	2012.00
49	NO	No	C71.0	30011.00	C71.0	YES	NO		330.00		NO		Right	YES	IHC	YES		NO	NO	NO	NO	G3	NO	TRUE	No	TCGA-DB-A75K.9ED9A0B1-8854-4E44-BD86-	A75K		TUMOR FREE	YES		Complete Remission/Response	YES	Primary Tumor	1.00	YES	NO		Cerebral Cortex	YES	YES	NO	DB	Supratentorial, Frontal Lobe	Central nervous system	A	NO	LIVING	2013.00
50	NO	No	C71.0	43891.00	C71.0	YES	NO		100.00		NO	90.00	Left	YES	IHC	YES		NO	NO	NO	NO	G3	NO	TRUE	No	TCGA-DB-A75L.A725AA31-18ED-47A2-9393-	A75L	Preoperative	TUMOR FREE	YES	NO	Complete Remission/Response	YES	Primary Tumor	1.00	YES	NO		Cerebral Cortex	YES	YES	NO	DB	Supratentorial, Frontal Lobe	Central nervous system	A	NO	LIVING	2013.00
51	NO	No	C71.0	36586.00	C71.0	YES	NO		250.00		NO		Right	YES	Sequence Analysis	YES		NO	NO	NO	NO	G2	NO	TRUE	No	TCGA-DB-A75M.C19F2C06-B479-44A0-8BE2-	A75M		TUMOR FREE	YES	NO	Complete Remission/Response	YES	Primary Tumor	1.00	YES	NO		Cerebral Cortex	YES	YES	NO	DB	Supratentorial, Parietal Lobe	Central nervous system	A	NO	LIVING	2013.00
52	NO	No	C71.0	43891.00	C71.0	YES	NO		240.00		NO	90.00	Left			NO		YES	NO	NO	NO	G3		TRUE	No	TCGA-DB-A75O.CADF06CC-B405-4CFF-AB8D-	A75O	Preoperative		YES	NO	Partial Remission/Response		Primary Tumor	1.00	YES	NO		Cerebral Cortex		YES	NO	DB	Supratentorial, Frontal Lobe	Central nervous system	A	NO	LIVING	2010.00
53	NO	No	C71.0	36586.00	C71.0	YES	NO		190.00		NO		Right	NO	IHC	YES		NO	NO	NO	NO	G2	NO	TRUE	No	TCGA-DB-A75P.6CD48114-C318-4450-AADD-	A75P		TUMOR FREE	YES	NO	Complete Remission/Response	NO	Primary Tumor	1.00	YES	NO		Cerebral Cortex	NO	YES	NO	DB	Supratentorial, Temporal Lobe	Central nervous system	A	NO	LIVING	2013.00
54	NO	No	C71.0	30011.00	C71.0	YES				0.50	NO	90.00	Left			NO	0.60		NO	NO	NO	G3	NO		No	TCGA-DH-5140.4f8b82c9-5185-4994-ac3d-	5140.00	Other	WITH TUMOR	YES	YES	Progressive Disease	YES	Primary Tumor	1.00	NO	NO	0.20	Cerebral Cortex	YES	NO	YES	DH	Supratentorial, Temporal Lobe	Central nervous system	A	NO	DECEASED	2007.00
55	NO	No	C71.0	18688.00	C71.0	YES				0.30	NO		Left			NO	0.60	YES	YES	NO	NO	G3			No	TCGA-DH-5141.15a0f3ec-2bdc-4fb9-ae65-	5141.00			YES	YES	Stable Disease	YES	Primary Tumor	1.00	NO	NO	0.20	Cerebral Cortex	YES	NO	YES	DH	Supratentorial, Frontal Lobe	Central nervous system	A	NO	LIVING	2005.00
56	NO	No	C71.0	43891.00	C71.0	YES				0.40	NO	90.00	Left			NO	0.40	YES	NO	NO	NO	G3	YES		No	TCGA-DH-5142.d6bad55b-31f4-416a-8775-	5142.00	Pre-Adjuvant Therapy	WITH TUMOR	NO	NO	Stable Disease	YES	Primary Tumor	1.00	YES	NO	0.30	Cerebral Cortex	YES	NO	YES	DH	Supratentorial, Frontal Lobe	Central nervous system	A	NO	LIVING	2008.00
57	NO	No	C71.0	30011.00	C71.0	YES				0.70	NO	80.00	Left			NO	1.00	YES	YES			G3	NO		No	TCGA-DH-5143.5cec11b4-9ba5-4a49-8c7c-	5143.00	Pre-Adjuvant Therapy	WITH TUMOR	YES	YES	Stable Disease	YES	Primary Tumor	1.00	YES		0.70	Cerebral Cortex	YES	NO	YES	DH	Supratentorial, Temporal Lobe	Central nervous system	A	NO	LIVING	2009.00
58	NO	No	C71.0	18688.00	C71.0	YES				0.60	NO	100.00	Right			NO	1.10	NO	NO	NO	NO	G3	NO		No	TCGA-DH-5144.95ca5a19-cf38-49f1-afac-	5144.00	Pre-Adjuvant Therapy	TUMOR FREE	YES	NO	Complete Remission/Response	YES	Primary Tumor	1.00	YES	NO	0.30	Cerebral Cortex	NO	NO	YES	DH	Supratentorial, Frontal Lobe	Central nervous system	A	NO	LIVING	2009.00
59	NO	No	C71.0	18688.00	C71.0	YES	NO						Left			NO			YES	NO	YES	G3	YES		No		A669		TUMOR FREE	NO	YES	Stable Disease	NO	Primary Tumor	1.00	NO	NO		Not listed in Medical Record	NO	NO	YES	DH	Supratentorial, Parietal Lobe	Central nervous system		NO	DECEASED	2007.00
60	NO	No	C71.0	18688.00	C71.0	YES	NO		50.00		NO		Left			NO			YES	NO	YES	G3	YES	FALSE	No	TCGA-DH-A669.B34CDA94-75EB-42E4-AAF8-	A669		TUMOR FREE	NO	YES	Stable Disease	NO	Recurrent Tumor	2.00	NO	NO		Not listed in Medical Record	NO	NO	YES	DH	Supratentorial, Parietal Lobe	Central nervous system	A	NO	DECEASED	2007.00
61	NO	No	C71.0	43891.00	C71.0	YES	NO		100.00		NO	90.00	Left			NO		NO	YES	NO	NO	G3	NO	FALSE	Yes, History of Prior Malignancy	TCGA-DH-A66B.7D6D3FE4-FA4C-4827-B141-	A66B	Post-Adjuvant Therapy	WITH TUMOR	YES	NO	Stable Disease	YES	Primary Tumor	1.00	NO	NO		Not listed in Medical Record	YES	NO	YES	DH	Supratentorial, Temporal Lobe	Central nervous system	A	NO	LIVING	2010.00
62	NO	No	C71.0	43891.00	C71.0	YES	NO		380.00		NO	80.00	Right	YES	IHC	YES		NO	YES	NO	NO	G3	NO	FALSE	No	TCGA-DH-A66D.44D22FE9-2B47-4638-A97F-	A66D	Post-Adjuvant Therapy	WITH TUMOR	NO	NO	Stable Disease	YES	Primary Tumor	1.00	YES	NO		Not listed in Medical Record	YES	NO	YES	DH	Supratentorial, Frontal Lobe	Central nervous system	A	NO	LIVING	2012.00
63	NO	No	C71.0	18323.00	C71.0	YES	NO		90.00		NO	90.00	Left	YES	IHC	YES		YES	NO		NO	G2	NO	FALSE	No	TCGA-DH-A66F.8D9C9718-7904-4C45-8217-	A66F	Other	TUMOR FREE	YES	YES	Complete Remission/Response	NO	Primary Tumor	1.00	YES	NO		Not listed in Medical Record	NO	YES	NO	DH	Supratentorial, Frontal Lobe	Central nervous system	A	NO	LIVING	2012.00
64	NO	No	C71.0	18688.00	C71.0	YES	NO		320.00		NO	80.00	Left	YES	IHC	YES		NO	NO	NO	NO	G3	NO	FALSE	No	TCGA-DH-A66G.6A01BF6C-E171-4D0A-977B-	A66G	Pre-Adjuvant Therapy	WITH TUMOR	YES	YES	Stable Disease	YES	Primary Tumor	1.00	YES	NO		Not listed in Medical Record	YES	YES	NO	DH	Supratentorial, Frontal Lobe	Central nervous system	A	NO	LIVING	2013.00
65	NO	No	C71.0	18688.00	C71.0	YES	NO					90.00	Right			NO		YES	YES	NO	NO	G3	NO		No		A7UR	Post-Adjuvant Therapy	TUMOR FREE	YES	NO	Complete Remission/Response	YES	Primary Tumor	1.00	YES	NO		Not listed in Medical Record	YES	NO	YES	DH	Supratentorial, Frontal Lobe	Central nervous system		NO	LIVING	2008.00
66	NO	No	C71.0	18323.00	C71.0	YES	NO		160.00		NO		Right	YES	IHC	YES		NO	NO	NO	NO	G2	NO	FALSE	No	TCGA-DH-A7US.1078C5C3-CA98-48D7-B810-	A7US		WITH TUMOR	YES	NO	Stable Disease	NO	Primary Tumor	1.00	YES	NO		Not listed in Medical Record	NO	YES	NO	DH	Supratentorial, Temporal Lobe	Central nervous system	A	NO	LIVING	2013.00
67	NO	No	C71.0	43891.00	C71.0	YES	NO		70.00		NO		Left	YES	IHC	YES		NO	NO	NO	NO	G3	YES	FALSE	No	TCGA-DH-A7UT.D737A708-EBF5-41F9-9245-	A7UT		WITH TUMOR	YES	YES	Stable Disease	YES	Primary Tumor	1.00	YES	NO		Not listed in Medical Record	YES	YES	NO	DH	Supratentorial, Frontal Lobe	Central nervous system	A	NO	DECEASED	2013.00
68	NO	No	C71.0	43891.00	C71.0	YES	NO					90.00	Right	YES	IHC	YES		NO	YES	YES	NO	G3	NO		No		A7UU	Pre-Adjuvant Therapy	WITH TUMOR	NO	NO	Stable Disease	YES	Primary Tumor	1.00	NO	YES		Not listed in Medical Record	YES	YES	NO	DH	Supratentorial, Frontal Lobe	Central nervous system		YES	LIVING	2013.00
69	NO	No	C71.0	43891.00	C71.0	YES	NO					90.00	Right	NO	IHC	YES		NO	YES		YES	G3	NO		No		A7UV	Pre-Adjuvant Therapy	WITH TUMOR	YES	NO	Stable Disease	YES	Primary Tumor	1.00	NO	NO		Not listed in Medical Record	YES	YES	NO	DH	Supratentorial, Temporal Lobe	Central nervous system		NO	LIVING	2013.00
70	NO	No	C71.9	43891.00	C71.9	YES				0.80	NO		Right			NO	1.00		NO	NO	NO	G3	NO		No	TCGA-DU-5847.d9f2d994-5d17-46c6-a6c9-	5847.00		WITH TUMOR	YES	NO	Stable Disease	YES	Primary Tumor	1.00	YES	NO	0.40	White Matter	YES	YES	NO	DU	Supratentorial, Frontal Lobe	Central nervous system	A	NO	LIVING	2009.00

71	NO	No	C71.9	18323.00	C71.9	YES				0.90	NO		Left			NO	0.90		NO		NO	G2	NO		No	TCGA-DU-5849.7ec64754-8800-44dd-a5cc-	5849.00		TUMOR FREE	YES	YES	Stable Disease	NO	Primary Tumor	1.00	YES	NO	0.40	White Matter	YES	YES	NO	DU	Supratentorial, Frontal Lobe	Central nervous system	A	NO	LIVING	2010.00
72	NO	No	C71.9	30011.00	C71.9	YES				1.00	NO	90.00	Right			NO	1.00		NO		NO	G3	NO		No	TCGA-DU-5851.beb751ed-b79d-43de-93b6-	5851.00	Pre-Adjuvant Therapy	WITH TUMOR	YES	YES	Stable Disease	YES	Primary Tumor	1.00	YES	YES	1.00	Cerebral Cortex	YES	YES	NO	DU	Supratentorial, Temporal Lobe	Central nervous system	A	NO	LIVING	2010.00
73	NO	No	C71.9	30011.00	C71.9	YES				0.90	NO	50.00	Left			NO	1.00		NO		YES	G3	YES		No	TCGA-DU-5852.a6c1f30a-8350-4fbc-9af2-	5852.00	Pre-Adjuvant Therapy	WITH TUMOR	YES	NO	Progressive Disease	NO	Primary Tumor	1.00	YES	NO	0.90	White Matter	NO	NO	YES	DU	Supratentorial, Temporal Lobe	Central nervous system	A	NO	DECEASED	2010.00
74	NO	No	C71.9	30011.00	C71.9	YES				0.90	NO	100.00	Left			NO	0.90		NO		NO	G2	NO		No	TCGA-DU-5853.d4bda6a8-21ef-4395-9091-	5853.00	Pre-Adjuvant Therapy	WITH TUMOR	YES	NO	Stable Disease	YES	Primary Tumor	1.00	YES	NO	0.80	Not listed in Medical Record	YES	YES	NO	DU	Supratentorial, Frontal Lobe	Central nervous system	A	NO	LIVING	2010.00
75	NO	No	C71.9	43891.00	C71.9	YES				0.90	NO	100.00	Right			NO	1.00		NO		YES	G3	YES		No	TCGA-DU-5854.e057679f-9741-4a4e-bb60-	5854.00	Pre-Adjuvant Therapy	WITH TUMOR	YES	YES	Progressive Disease	YES	Primary Tumor	1.00	NO	YES	0.70	Not listed in Medical Record	YES	YES	NO	DU	Supratentorial, Frontal Lobe	Central nervous system	A	NO	LIVING	2010.00
76	NO	No	C71.9	30011.00	C71.9	YES				0.70	NO	100.00	Right			NO	1.10		YES		NO	G3	NO		No	TCGA-DU-5855.ba07cd68-b0ac-4e98-bc4b-	5855.00	Pre-Adjuvant Therapy	WITH TUMOR	YES	YES	Stable Disease	YES	Primary Tumor	1.00	NO	NO	0.70	Not listed in Medical Record	YES	YES	NO	DU	Supratentorial, Frontal Lobe	Central nervous system	A	NO	LIVING	2010.00
77	NO	No	C71.9	18323.00	C71.9	YES				0.80	NO	70.00	Right			NO	0.90		YES		NO	G2	YES		No	TCGA-DU-5870.5A7D5219-AFF1-4DC0-9A8B-	5870.00	Post-Adjuvant Therapy	WITH TUMOR			Progressive Disease	YES	Primary Tumor	1.00	NO	YES	0.70	Not listed in Medical Record	NO	NO	YES	DU	Supratentorial, Temporal Lobe	Central nervous system	A	NO	LIVING	1995.00
78	NO	No	C71.9	18323.00	C71.9	YES			390.00		NO	70.00	Right			NO			YES		NO	G2	YES	FALSE	No	TCGA-DU-5870.5A7D5219-AFF1-4DC0-9A8B-	5870.00	Post-Adjuvant Therapy	WITH TUMOR			Progressive Disease	YES	Recurrent Tumor	2.00	NO	YES		Not listed in Medical Record	NO	NO	YES	DU	Supratentorial, Temporal Lobe	Central nervous system	A	NO	LIVING	1995.00
79	NO	No	C71.9	30011.00	C71.9	YES				1.00	NO	100.00	Left			NO	1.70		NO		NO	G2	NO		No	TCGA-DU-5871.fc11347a-6c78-46ed-a07d-	5871.00	Preoperative	TUMOR FREE	YES	YES	Stable Disease	YES	Primary Tumor	1.00	YES	NO	0.90	White Matter	YES	YES	NO	DU	Supratentorial, Frontal Lobe	Central nervous system	A	NO	LIVING	2009.00
80	NO	No	C71.9	30011.00	C71.9	YES				1.00	NO		Left			NO	1.20		NO		NO	G2	YES		No	TCGA-DU-5872.86EEC915-5F91-4C9E-B243-	5872.00		WITH TUMOR	YES	YES	Progressive Disease	YES	Primary Tumor	1.00	NO	NO	1.00	Not listed in Medical Record	YES	YES	NO	DU	Supratentorial, Frontal Lobe	Central nervous system	A	NO	LIVING	2009.00
81	NO	No	C71.9	30011.00	C71.9	YES			430.00		NO		Left			NO			NO		NO	G2	YES	FALSE	No	TCGA-DU-5872.86EEC915-5F91-4C9E-B243-	5872.00		WITH TUMOR	YES	YES	Progressive Disease	YES	Recurrent Tumor	2.00	NO	NO		Not listed in Medical Record	YES	YES	NO	DU	Supratentorial, Frontal Lobe	Central nervous system	A	NO	LIVING	2009.00
82	NO	No	C71.9	18323.00	C71.9	YES				1.00	NO	100.00	Left			NO	1.00		NO		NO	G2	NO		No	TCGA-DU-5874.edd1c152-5b40-4f2f-a7bc-	5874.00	Pre-Adjuvant Therapy	TUMOR FREE	YES	NO	Stable Disease	NO	Primary Tumor	1.00	YES	NO	0.70	Not listed in Medical Record	YES	YES	NO	DU	Supratentorial, Frontal Lobe	Central nervous system	A	NO	LIVING	2010.00
83	NO	No	C71.0	43891.00	C71.0	YES				1.00	NO		Right			NO	1.00					G3	YES		No	TCGA-DU-6392.ebd2fe7e-1d87-4831-b605-	6392.00		TUMOR FREE			Progressive Disease	YES	Primary Tumor	1.00	YES		0.60	White Matter	YES	NO	YES	DU	Supratentorial, Parietal Lobe	Central nervous system	A		LIVING	1993.00
84	NO	No	C71.0	18688.00	C71.0	YES				0.70	NO		Left			NO	0.70					G3			No	TCGA-DU-6393.13d53e5b-0ff2-4903-91a4-	6393.00						YES	Primary Tumor	1.00			0.50	White Matter	YES	NO	YES	DU	Supratentorial, Occipital Lobe	Central nervous system	A		DECEASED	1993.00
85	NO	No	C71.0	18688.00	C71.0	YES				1.00	NO		Midline				1.00					G3	YES		No	TCGA-DU-6394.300d6b45-ff77-493d-9b07-	6394.00		WITH TUMOR			Progressive Disease	YES	Primary Tumor	1.00			1.00	White Matter	YES	NO	YES	DU	Supratentorial, Frontal Lobe	Central nervous system	A		DECEASED	1994.00
86	NO	No	C71.0	30011.00	C71.0	YES				0.90	NO		Left			NO	1.00		NO		NO	G2	YES		No	TCGA-DU-6395.06DC35CF-0317-47F4-9F85-	6395.00		WITH TUMOR			Progressive Disease	YES	Primary Tumor	1.00	YES	NO	0.40	Not listed in Medical Record	NO	NO	YES	DU	Supratentorial, Frontal Lobe	Central nervous system	A	NO	DECEASED	1994.00
87	NO	No	C71.0	30011.00	C71.0	YES				1.00	NO	90.00	Right			NO	1.00		NO		YES	G3	YES		No	TCGA-DU-6396.23ea2bb8-e87f-4956-8a97-	6396.00	Post-Adjuvant Therapy	WITH TUMOR			Progressive Disease	YES	Primary Tumor	1.00	NO	YES	1.00	White Matter	YES	NO	YES	DU	Supratentorial, Frontal Lobe	Central nervous system	A	NO	DECEASED	1996.00
88	NO	No	C71.0	18688.00	C71.0	YES				1.00	NO		Right			NO	1.30		YES			G3	YES		No	TCGA-DU-6397.54C5D9F8-8737-49DA-81CF-	6397.00		WITH TUMOR			Progressive Disease	YES	Primary Tumor	1.00	NO		0.80	Not listed in Medical Record	NO	NO	YES	DU	Supratentorial, Temporal Lobe	Central nervous system	A	YES	DECEASED	1997.00
89	NO	No	C71.0	18688.00	C71.0	YES			200.00		NO		Right			NO			YES			G3	YES	FALSE	No	TCGA-DU-6397.54C5D9F8-8737-49DA-81CF-	6397.00		WITH TUMOR			Progressive Disease	YES	Recurrent Tumor	2.00	NO			Not listed in Medical Record	NO	NO	YES	DU	Supratentorial, Temporal Lobe	Central nervous system	A	YES	DECEASED	1997.00
90	NO	No	C71.0	18323.00	C71.0	YES				1.00	NO		Right			NO	2.00		NO		NO	G2	YES		No	TCGA-DU-6399.e0803e30-ae37-4c26-8d3b-	6399.00		WITH TUMOR			Progressive Disease	YES	Primary Tumor	1.00	NO	NO	1.00	White Matter	NO	NO	YES	DU	Supratentorial, Temporal Lobe	Central nervous system	A	NO	DECEASED	1998.00
91	NO	No	C71.0	18323.00	C71.0	YES				0.60	NO		Left			NO	1.00		YES		YES	G2	NO		No	TCGA-DU-6400.a61be291-6258-47f2-8404-	6400.00		WITH TUMOR	YES	YES		NO	Primary Tumor	1.00	NO	NO	0.50	Not listed in Medical Record	NO	NO	YES	DU	Supratentorial, Temporal Lobe	Central nervous system	A	NO	DECEASED	1998.00
92	NO	No	C71.0	18323.00	C71.0	YES				0.80	NO	100.00	Left			NO	1.50		NO		NO	G2	YES		No	TCGA-DU-6401.8B65EAA0-2BE6-4648-9CF0-	6401.00	Preoperative	WITH TUMOR	YES	YES	Progressive Disease	YES	Primary Tumor	1.00	NO	NO	0.50	Not listed in Medical Record	NO	NO	YES	DU	Supratentorial, Frontal Lobe	Central nervous system	A	YES	DECEASED	1998.00
93	NO	No	C71.0	43891.00	C71.0	YES				1.00	NO		Right			NO	1.50					G3	YES		No	TCGA-DU-6402.cf00fc85-c4be-4d4a-987a-	6402.00		WITH TUMOR			Progressive Disease	YES	Primary Tumor	1.00	YES		0.50	Not listed in Medical Record	YES	NO	YES	DU	Supratentorial, Frontal Lobe	Central nervous system	A		DECEASED	1998.00
94	NO	No	C71.0	30011.00	C71.0	YES				0.60	NO	90.00	Right			NO	0.90		NO		NO	G3			No	TCGA-DU-6403.86c8b229-d75e-48ce-849e-	6403.00	Post-Adjuvant Therapy	WITH TUMOR		YES		YES	Primary Tumor	1.00	NO	NO	0.50	Not listed in Medical Record	YES	NO	YES	DU	Supratentorial, Temporal Lobe	Central nervous system	A	NO	DECEASED	1999.00
95	NO	No	C71.0	18688.00	C71.0	YES				0.60	NO	100.00	Right			NO	1.00		NO	NO	NO	G3	YES		No		6404.00	Post-Adjuvant Therapy	WITH TUMOR	YES	NO	Progressive Disease	YES	Primary Tumor	1.00	YES	NO	0.30	Not listed in Medical Record	YES	NO	YES	DU	Supratentorial, Frontal Lobe	Central nervous system	A	NO	DECEASED	2000.00
96	NO	No	C71.0	18688.00	C71.0	YES			200.00		NO	100.00	Right			NO			NO	NO	NO	G3	YES	FALSE	No	TCGA-DU-6404.C13F0838-3BA3-4C58-BDCC-	6404.00	Post-Adjuvant Therapy	WITH TUMOR	YES	NO	Progressive Disease	YES	Recurrent Tumor	2.00	YES	NO		Not listed in Medical Record	YES	NO	YES	DU	Supratentorial, Frontal Lobe	Central nervous system	A	NO	DECEASED	2000.00
97	NO	No	C71.0	43891.00	C71.0	YES				1.00	NO	60.00	Right			NO	1.00					G3	YES		No	TCGA-DU-6405.5a26ce1c-d97c-4123-a573-	6405.00	Post-Adjuvant Therapy	WITH TUMOR	YES	NO	Progressive Disease	YES	Primary Tumor	1.00	YES		0.60	Not listed in Medical Record	YES	NO	YES	DU	Supratentorial, Frontal Lobe	Central nervous system	A		DECEASED	2000.00
98	NO	No	C71.0	30011.00	C71.0	YES				0.50	NO		Left			NO	1.00		NO		YES	G3			No	TCGA-DU-6406.84775a3e-6deb-477c-bd7b-	6406.00						YES	Primary Tumor	1.00	NO	YES	0.50	Not listed in Medical Record	YES	NO	YES	DU	Supratentorial, Frontal Lobe	Central nervous system	A	YES	DECEASED	2001.00
99	NO	No	C71.0	18323.00	C71.0	YES				0.80	NO	80.00	Right			NO	0.80		NO		NO	G2	YES		No		6407.00	Pre-Adjuvant Therapy	WITH TUMOR	YES	NO	Progressive Disease	YES	Primary Tumor	1.00	YES	NO	0.50	Not listed in Medical Record	NO	NO	YES	DU	[Discrepancy]	Central nervous system	A	NO	DECEASED	2001.00
100	NO	No	C71.0	18323.00	C71.0	YES			390.00		NO	80.00	Right			NO			NO		NO	G2	YES	FALSE	No	TCGA-DU-6407.49BAF246-AE0A-4627-AF91-	6407.00	Pre-Adjuvant Therapy	WITH TUMOR	YES	NO	Progressive Disease	YES	Recurrent Tumor	2.00	YES	NO		Not listed in Medical Record	NO	NO	YES	DU	[Discrepancy]	Central nervous system	B	NO	DECEASED	2001.00
101	NO	No	C71.0	18688.00	C71.0	YES				1.00	NO	90.00	Right			NO	1.00		YES		YES	G3	YES		No	TCGA-DU-6408.83c5c547-8f79-4b30-9883-	6408.00	Post-Adjuvant Therapy		YES	YES	Progressive Disease	YES	Primary Tumor	1.00	YES	NO	0.50	Not listed in Medical Record	YES	NO	YES	DU	Supratentorial, Frontal Lobe	Central nervous system	A	NO	DECEASED	1998.00
102	NO	No	C71.0	18688.00	C71.0	YES				0.80	NO	50.00	Midline			NO	0.80		YES			G3	NO		No	TCGA-DU-6410.d7745e3b-3ccd-40ff-8634-	6410.00	Pre-Adjuvant Therapy	WITH TUMOR				NO	Primary Tumor	1.00			0.60	Not listed in Medical Record	YES	YES	NO	DU	Supratentorial, Frontal Lobe	Central nervous system	A		LIVING	2010.00
103	NO	No	C71.0	30011.00	C71.0	YES				0.90	NO		Right			NO	1.00	YES	NO		NO	G3			No	TCGA-DU-6542.bd0d6d1b-bdcd-4c33-9adc-	6542.00		WITH TUMOR	YES	YES		YES	Primary Tumor	1.00	NO	NO	0.90	Not listed in Medical Record	YES	YES	NO	DU	Supratentorial, Temporal Lobe	Central nervous system	A	YES	LIVING	2011.00
104	NO	No	C71.0	43891.00	C71.0	YES				0.80	NO		Left			NO	1.00		NO		NO	G3			No	TCGA-DU-7006.1341bbae-b2bf-43fb-be7e-	7006.00		WITH TUMOR			Progressive Disease	YES	Primary Tumor	1.00	YES	NO	0.40	Cerebral Cortex	YES	NO	YES	DU	Supratentorial, Temporal Lobe	Central nervous system	A	NO	DECEASED	1995.00
105	NO	No	C71.0	36586.00	C71.0	YES				1.00	NO		Right				1.00		NO		NO	G2	NO		No	TCGA-DU-7007.ffbbe732-9a5a-4269-b0a7-	7007.00							Primary Tumor	1.00	NO	YES	0.60	Not listed in Medical Record		NO	YES	DU	Supratentorial, Frontal Lobe	Central nervous system	A	NO	DECEASED	1997.00
106	NO	No	C71.0	18323.00	C71.0	YES				0.60	NO		Right			NO	0.80		NO		NO	G2	YES		No	TCGA-DU-7008.70aff058-6c24-488c-89ae-	7008.00		TUMOR FREE	YES	YES	Progressive Disease	NO	Primary Tumor	1.00	YES	NO	0.50	Not listed in Medical Record	YES	NO	YES	DU	Supratentorial, Frontal Lobe	Central nervous system	A	NO	LIVING	1998.00

107	NO	No	C71.0	18323.00	C71.0	YES				1.00	NO		Left			NO	1.00	NO	NO		NO	G2	YES		No	TCGA-DU-7009.bc350788-3a70-47d5-b2ef-	7009.00		WITH TUMOR			Progressive Disease	YES	Primary Tumor	1.00	NO	YES	0.50	Cerebral Cortex	YES	NO	YES	DU	Supratentorial, Frontal Lobe	Central nervous system	A	NO	DECEASED	2000.00
108	NO	No	C71.0	43891.00	C71.0	YES				1.00	NO		Left			NO	1.10		NO		NO	G3	YES		No	TCGA-DU-7010.B62E7173-FC3E-4D4C-B9C0-	7010.00		WITH TUMOR	YES	YES	Progressive Disease	YES	Primary Tumor	1.00	YES	NO	0.50	Not listed in Medical Record	YES	NO	YES	DU	Supratentorial, Frontal Lobe	Central nervous system	A	NO	DECEASED	2001.00
109	NO	No	C71.0	30011.00	C71.0	YES				1.00	NO	80.00	Right			NO	1.00		NO		NO	G2			No	TCGA-DU-7011.4f0cbf0f-f9f0-44f4-b498-	7011.00	Pre-Adjuvant Therapy	WITH TUMOR	YES	YES	Stable Disease	YES	Primary Tumor	1.00	YES	NO	0.30	Cerebral Cortex		NO	YES	DU	Supratentorial, Frontal Lobe	Central nervous system	A	NO	DECEASED	2000.00
110	NO	No	C71.0	43891.00	C71.0	YES				1.00	NO		Left			NO	1.10		YES		NO	G3	NO		No	TCGA-DU-7012.097539a1-a885-478d-9a11-	7012.00		WITH TUMOR			Progressive Disease	YES	Primary Tumor	1.00	YES	NO	0.90	Not listed in Medical Record	NO	NO	YES	DU	Supratentorial, Temporal Lobe	Central nervous system	A	NO	DECEASED	2001.00
111	NO	No	C71.0	43891.00	C71.0	YES				1.00	NO	70.00	Left			NO	1.00		NO		NO	G3	YES		No	TCGA-DU-7013.a39cad6f-249d-4bc5-8d67-	7013.00	Post-Adjuvant Therapy	WITH TUMOR	YES	YES	Progressive Disease	YES	Primary Tumor	1.00	YES	NO	0.50	White Matter	YES	NO	YES	DU	Supratentorial, Temporal Lobe	Central nervous system	A	NO	DECEASED	2001.00
112	NO	No	C71.0	18323.00	C71.0	YES				1.00	NO	50.00	Right			YES	1.00		NO		NO	G2	YES		No	TCGA-DU-7014.a848a22c-c92d-42cc-8e0b-	7014.00	Pre-Adjuvant Therapy	WITH TUMOR	YES	YES	Progressive Disease	YES	Primary Tumor	1.00	YES	NO	0.60	White Matter	NO	NO	YES	DU	Supratentorial, Frontal Lobe	Central nervous system	A	NO	DECEASED	2001.00
113	NO	No	C71.0	18323.00	C71.0	YES				1.00	NO	90.00	Right			NO	1.00		NO		NO	G2	YES		No	TCGA-DU-7015.15dbad7b-e2ae-42b0-92a1-	7015.00	Pre-Adjuvant Therapy	WITH TUMOR	YES	YES	Progressive Disease	NO	Primary Tumor	1.00	YES	NO	0.90	Cerebral Cortex	NO	NO	YES	DU	Supratentorial, Temporal Lobe	Central nervous system	A	NO	LIVING	2004.00
114	NO	No	C71.0	18688.00	C71.0	YES				1.00	NO	90.00	Right			YES	1.00		YES		NO	G3	YES		No	TCGA-DU-7018.caa41707-cb2e-4c3f-bae9-	7018.00	Pre-Adjuvant Therapy	WITH TUMOR	YES		Progressive Disease	NO	Primary Tumor	1.00	YES	NO	0.50	Not listed in Medical Record	YES	NO	YES	DU	Supratentorial, Frontal Lobe	Central nervous system	A	NO	DECEASED	2006.00
115	NO	No	C71.0	30011.00	C71.0	YES				1.00	NO	100.00	Right			NO	1.00		NO		NO	[Discrepancy]	NO		No	TCGA-DU-7019.a389c9c9-e639-49c6-a8d8-	7019.00	Pre-Adjuvant Therapy	TUMOR FREE	YES	YES	Stable Disease	YES	Primary Tumor	1.00	NO	NO	1.00	Cerebral Cortex	YES	YES	NO	DU	Supratentorial, Temporal Lobe	Central nervous system	A	YES	LIVING	2009.00
116	NO	No	C71.0	43891.00	C71.0	YES				0.80	NO		Right			NO	1.10		NO		NO	G3			No	TCGA-DU-7290.06977359-42d7-453c-9ed3-	7290.00		WITH TUMOR				NO	Primary Tumor	1.00	NO	YES	0.60	Cerebral Cortex	NO	NO	YES	DU	Supratentorial, Temporal Lobe	Central nervous system	A	YES	DECEASED	1995.00
117	NO	No	C71.0	43891.00	C71.0	YES	NO			1.00	NO		Left			NO	1.20		NO		NO	G3	YES		No	TCGA-DU-7292.13d45e58-4eb6-405e-916f-	7292.00		WITH TUMOR			Progressive Disease	YES	Primary Tumor	1.00	YES	NO	0.40	Not listed in Medical Record	NO	NO	YES	DU	Supratentorial, Temporal Lobe	Central nervous system	A	NO	DECEASED	1997.00
118	NO	No	C71.0	18323.00	C71.0	YES				1.00	NO	100.00	Right			NO	1.10		NO		NO	G2	NO		No	TCGA-DU-7294.3f73eaa4-67da-4c34-98a1-	7294.00	Pre-Adjuvant Therapy	TUMOR FREE	NO	NO	Stable Disease	NO	Primary Tumor	1.00	NO	NO	0.90	Not listed in Medical Record	NO	NO	YES	DU	Supratentorial, Frontal Lobe	Central nervous system	A	NO	LIVING	2003.00
119	NO	No	C71.0	43891.00	C71.0	YES				0.70	NO	80.00	Left			NO	1.00		NO		NO	G3	YES		No	TCGA-DU-7298.14bf3d59-284e-40d0-ba0b-	7298.00	Preoperative	WITH TUMOR	YES	YES	Progressive Disease	YES	Primary Tumor	1.00	NO	NO	0.50	Cerebral Cortex	YES	NO	YES	DU	Supratentorial, Temporal Lobe	Central nervous system	A	YES	DECEASED	2006.00
120	NO	No	C71.0	43891.00	C71.0	YES				1.00	NO	90.00	Left			NO	1.00		NO		NO	G3	YES		No	TCGA-DU-7299.36ca34b7-04ee-4cbb-aba8-	7299.00	Post-Adjuvant Therapy	WITH TUMOR	YES	YES	Progressive Disease	YES	Primary Tumor	1.00	YES	NO	0.30	Not listed in Medical Record	YES	NO	YES	DU	Supratentorial, Parietal Lobe	Central nervous system	A	NO	DECEASED	2006.00
121	NO	No	C71.0	18688.00	C71.0	YES				1.00	NO	100.00	Right			NO	1.00		NO		NO	G3	YES		Yes	TCGA-DU-7300.6a6a701f-4276-4ee8-9d47-	7300.00	Post-Adjuvant Therapy	WITH TUMOR	YES	NO	Progressive Disease	NO	Primary Tumor	1.00	YES	NO	0.40	Not listed in Medical Record	YES	NO	YES	DU	Supratentorial, Frontal Lobe	Central nervous system	A	NO	DECEASED	2006.00
122	NO	No	C71.0	18323.00	C71.0	YES				1.00	NO	60.00	Left			NO	1.00		NO		NO	G2	YES		No	TCGA-DU-7301.8ff62d9f-003f-4194-8cc6-	7301.00	Post-Adjuvant Therapy	WITH TUMOR	YES	NO	Progressive Disease	YES	Primary Tumor	1.00	YES	YES	0.50	Not listed in Medical Record	YES	NO	YES	DU	Supratentorial, Temporal Lobe	Central nervous system	A	NO	DECEASED	2006.00
123	NO	No	C71.0	18688.00	C71.0	YES				0.90	NO	70.00	Left			NO	1.00		NO		NO	G3	YES		No	TCGA-DU-7302.ad715b7b-52a2-493a-99d2-	7302.00	Post-Adjuvant Therapy	WITH TUMOR	YES	YES	Progressive Disease	NO	Primary Tumor	1.00	YES	NO	0.70	Not listed in Medical Record	YES	NO	YES	DU	Supratentorial, Frontal Lobe	Central nervous system	A	NO	LIVING	2006.00
124	NO	No	C71.0	30011.00	C71.0	YES				0.80	NO	80.00	Left			NO	1.00		NO		NO	G3	YES		No	TCGA-DU-7304.EFF2E68D-4795-413F-B5B1-	7304.00	Post-Adjuvant Therapy	WITH TUMOR	YES	YES	Progressive Disease	YES	Primary Tumor	1.00	YES	NO	0.60	Not listed in Medical Record	YES	NO	YES	DU	Supratentorial, Frontal Lobe	Central nervous system	A	NO	DECEASED	2008.00
125	NO	No	C71.0	30011.00	C71.0	YES			280.00		NO	80.00	Left			NO			NO		NO	G3	YES	FALSE	No	TCGA-DU-7304.EFF2E68D-4795-413F-B5B1-	7304.00	Post-Adjuvant Therapy	WITH TUMOR	YES	YES	Progressive Disease	YES	Recurrent Tumor	2.00	YES	NO		Not listed in Medical Record	YES	NO	YES	DU	Supratentorial, Frontal Lobe	Central nervous system	A	NO	DECEASED	2008.00
126	NO	No	C71.0	30011.00	C71.0	YES				1.10	NO	100.00	Right			NO	1.10		NO		NO	G2	YES		No	TCGA-DU-7306.dfe1d2e3-a9c7-4d49-984d-	7306.00	Preoperative	WITH TUMOR	YES	YES	Progressive Disease	YES	Primary Tumor	1.00	YES	NO	0.70	Not listed in Medical Record	YES	NO	YES	DU	Supratentorial, Temporal Lobe	Central nervous system	A	NO	LIVING	2008.00
127	NO	No	C71.0	18688.00	C71.0	YES				0.90	NO	90.00	Left			NO	1.00		NO		YES	G3	NO		No	TCGA-DU-7309.0b731266-908e-477b-8228-	7309.00	Pre-Adjuvant Therapy	WITH TUMOR	YES	NO	Stable Disease	YES	Primary Tumor	1.00	YES	YES	0.60	Cerebral Cortex	YES	YES	NO	DU	Supratentorial, Frontal Lobe	Central nervous system	A	YES	LIVING	2011.00
128	NO	No	C71.0	43891.00	C71.0	YES				0.90	NO	80.00	Left			NO	0.90		YES			G3			No	TCGA-DU-8158.83e22d80-b93f-48cc-9f4f-	8158.00	Pre-Adjuvant Therapy	WITH TUMOR					Primary Tumor	1.00			0.50			NO	YES	DU	Supratentorial, Frontal Lobe	Central nervous system	A		DECEASED	2001.00
129	NO	No	C71.0	30011.00	C71.0	YES				1.00	NO	70.00	Left			NO	1.00	NO	NO		NO	G3	YES		No	TCGA-DU-8161.867daa2d-0b54-4ac8-9203-	8161.00	Pre-Adjuvant Therapy	WITH TUMOR	YES		Progressive Disease	YES	Primary Tumor	1.00	NO	YES	0.70	Not listed in Medical Record	YES	NO	YES	DU	Supratentorial, Not Otherwise Specified	Central nervous system	A	NO	DECEASED	2009.00
130	NO	No	C71.0	30011.00	C71.0	YES				0.90	NO	80.00	Right			NO	1.20	NO	NO		NO	G3	YES		No	TCGA-DU-8162.a9f8f064-c21c-4d7e-ae38-	8162.00	Pre-Adjuvant Therapy	WITH TUMOR			Progressive Disease	YES	Primary Tumor	1.00	NO	NO	0.90	Not listed in Medical Record	YES	YES	NO	DU	Supratentorial, Temporal Lobe	Central nervous system	A	NO	DECEASED	2011.00
131	NO	No	C71.0	30011.00	C71.0	YES				1.00	NO	90.00	Left			NO	1.00	NO	NO		NO	G3	YES		No	TCGA-DU-8163.7c9b671f-e8f9-4b12-b497-	8163.00		WITH TUMOR	YES	NO	Progressive Disease	YES	Primary Tumor	1.00	YES	NO	0.60	Not listed in Medical Record	YES	YES	NO	DU	Supratentorial, Temporal Lobe	Central nervous system	A	NO	LIVING	2011.00
132	NO	No	C71.0	18323.00	C71.0	YES	NO			0.80	NO		Right	YES	IHC	YES	0.80	NO	NO		NO	G2	NO		No	TCGA-DU-8164.452cb553-ee9b-44e3-bbc4-	8164.00		TUMOR FREE	YES	NO	Stable Disease	NO	Primary Tumor	1.00	YES	NO	0.60	Cerebral Cortex	YES	YES	NO	DU	Supratentorial, Frontal Lobe	Central nervous system	A	NO	LIVING	2011.00
133	NO	No	C71.0	18323.00	C71.0	YES				1.00	NO	90.00	Left			NO	1.10	NO	NO		NO	G3	NO		No	TCGA-DU-8165.611a4975-123d-470e-afa7-	8165.00	Pre-Adjuvant Therapy	TUMOR FREE	YES	NO	Stable Disease	YES	Primary Tumor	1.00	YES	NO	1.00	Not listed in Medical Record	YES	YES	NO	DU	Supratentorial, Temporal Lobe	Central nervous system	A	NO	LIVING	2011.00
134	NO	No	C71.0	30011.00	C71.0	YES				0.90	NO		Left			NO	0.90	NO	NO		NO	G2	YES		No	TCGA-DU-8166.d4140f71-f42d-4b23-bff6-	8166.00		WITH TUMOR	YES	YES	Progressive Disease	YES	Primary Tumor	1.00	YES	NO	0.50	Cerebral Cortex	YES	YES	NO	DU	Supratentorial, Frontal Lobe	Central nervous system	A	NO	LIVING	2012.00
135	NO	No	C71.0	30011.00	C71.0	YES				1.20	NO	100.00	Right			NO	1.20	NO	NO		NO	G2	NO		No	TCGA-DU-8167.8c93cc66-38b1-451f-b941-	8167.00	Preoperative	TUMOR FREE	YES	NO	Stable Disease	YES	Primary Tumor	1.00	YES	NO	0.40	Not listed in Medical Record	NO	YES	NO	DU	Supratentorial, Frontal Lobe	Central nervous system	A	NO	LIVING	2012.00
136	NO	No	C71.0	18688.00	C71.0	YES				0.90	NO	70.00	Right			NO	0.90	NO	NO		NO	G3	NO		No	TCGA-DU-8168.e96918fc-f639-46eb-b87a-	8168.00	Pre-Adjuvant Therapy	TUMOR FREE	NO	YES	Stable Disease	YES	Primary Tumor	1.00	NO	NO	0.80	Not listed in Medical Record	YES	YES	NO	DU	Supratentorial, Frontal Lobe	Central nervous system	A	NO	LIVING	2012.00
137	YES	No	C71.0	43891.00	C71.0	YES			280.00		NO	70.00	Right			NO		NO	NO	NO	NO	G3	YES	FALSE	No	TCGA-DU-A5TP.CD515D5F-80C5-4571-B431-	A5TP	Pre-Adjuvant Therapy	TUMOR FREE	YES	NO	[Discrepancy]	YES	Primary Tumor	1.00	YES	NO		Not listed in Medical Record	YES	YES	NO	DU	Supratentorial, Parietal Lobe	Central nervous system	A	NO	LIVING	2012.00
138	NO	No	C71.0	30011.00	C71.0	YES			280.00		NO	90.00	Left			NO		NO	YES	NO	NO	G2	YES	FALSE	No	TCGA-DU-A5TR.7BF16354-F6FD-428D-ACB3-	A5TR	Pre-Adjuvant Therapy	TUMOR FREE	YES	YES	[Discrepancy]	NO	Primary Tumor	1.00	YES	NO		Not listed in Medical Record	YES	YES	NO	DU	Supratentorial, Temporal Lobe	Central nervous system	A	NO	LIVING	2012.00
139	NO	No	C71.0	18323.00	C71.0	YES			360.00		NO	100.00	Right			NO		NO	NO		NO	G2	YES	FALSE	No	TCGA-DU-A5TS.1D94A4FE-F273-4DD4-AB30-	A5TS	Pre-Adjuvant Therapy	TUMOR FREE	NO	YES	Stable Disease	YES	Primary Tumor	1.00	NO	NO		Not listed in Medical Record	YES	YES	NO	DU	Supratentorial, Frontal Lobe	Central nervous system	A	NO	LIVING	2012.00
140	NO	No	C71.0	18688.00	C71.0	YES			420.00		NO		Left	NO	IHC	YES		NO	NO	NO	NO	G3	YES	FALSE	No	TCGA-DU-A5TT.6E55B698-D0BA-4834-91EE-	A5TT		WITH TUMOR	YES	NO	[Discrepancy]	YES	Primary Tumor	1.00	YES	NO		Not listed in Medical Record	YES	YES	NO	DU	Supratentorial, Frontal Lobe	Central nervous system	A	NO	LIVING	2013.00
141	NO	No	C71.0	36586.00	C71.0	YES	NO		300.00		NO	50.00	Left			NO		NO	YES	NO	NO	G2	YES	FALSE	No	TCGA-DU-A5TU.6F62570D-D7DE-45F2-8025-	A5TU	Other	WITH TUMOR	NO	YES	Progressive Disease	YES	Primary Tumor	1.00	NO	NO		Cerebral Cortex	NO	YES	NO	DU	Supratentorial, Frontal Lobe	Central nervous system	A	NO	LIVING	2013.00
142	NO	No	C71.0	43891.00	C71.0	YES			320.00		NO	100.00	Right	YES	IHC	YES		NO	YES		NO	G3	NO	FALSE	No	TCGA-DU-A5TW.CE930C5F-0F10-4CC9-AF72-	A5TW	Other	WITH TUMOR	YES	YES	Progressive Disease	NO	Primary Tumor	1.00	YES	NO		Not listed in Medical Record	NO	YES	NO	DU	Supratentorial, Frontal Lobe	Central nervous system	A	NO	LIVING	2012.00

143	NO	No	C71.0	43891.00	C71.0	YES			130.00		NO		Left	NO	IHC	YES		NO	YES	NO	NO	G3	YES	FALSE	No	TCGA-DU-A5TY.70236BAA-BD1E-4AA3-A8D8-	A5TY		WITH TUMOR	YES	YES	[Discrepancy]	YES	Primary Tumor	1.00	YES	NO		Not listed in Medical Record	YES	YES	NO	DU	Supratentorial, Parietal Lobe	Central nervous system	A	NO	DECEASED	2012.00
144	NO	No	C71.0	18323.00	C71.0	YES	NO		110.00		NO	70.00	Right	YES	IHC	YES		NO	NO	YES	NO	G2	NO	FALSE	No	TCGA-DU-A6S2.163325D0-4CEA-4AC4-	A6S2	Pre-Adjuvant Therapy	TUMOR FREE	NO	YES	Stable Disease	NO	Primary Tumor	1.00	NO	NO		Cerebral Cortex	NO	YES	NO	DU	Supratentorial, Frontal Lobe	Central nervous system	A	NO	LIVING	2013.00
145	NO	No	C71.0	18323.00	C71.0	YES	NO		270.00		NO		Right			NO		NO	NO		YES	G2	NO	FALSE	No	TCGA-DU-A6S3.913A3754-B550-431A-B9B7-	A6S3		TUMOR FREE	NO	NO	Stable Disease	NO	Primary Tumor	1.00	NO	NO		Not listed in Medical Record	YES	YES	NO	DU	Supratentorial, Frontal Lobe	Central nervous system	A	NO	LIVING	2013.00
146	NO	No	C71.0	30011.00	C71.0	YES	NO					90.00	Right			NO		NO	NO	NO	NO	G2	YES		No		A6S6	Other	TUMOR FREE	NO	NO	[Discrepancy]	NO	Primary Tumor	1.00	NO	NO		White Matter	YES	NO	YES	DU	Supratentorial, Frontal Lobe	Central nervous system		NO	LIVING	2007.00
147	NO	No	C71.0	43891.00	C71.0	YES	NO					90.00	Left	YES	IHC	YES		NO	NO	NO	NO	G3	NO		No		A6S7	Preoperative	TUMOR FREE	YES	NO	Stable Disease	YES	Primary Tumor	1.00	YES	NO		Not listed in Medical Record	YES	YES	NO	DU	Supratentorial, Frontal Lobe	Central nervous system		NO	LIVING	2013.00
148	NO	No	C71.0	18688.00	C71.0	YES	NO		390.00		NO	90.00	Right	YES	IHC	YES		NO	YES	NO	NO	G3	YES	FALSE	No	TCGA-DU-A6S8.CCEA4518-483F-48A1-92C5-	A6S8	Pre-Adjuvant Therapy	WITH TUMOR	NO	NO	Progressive Disease	NO	Primary Tumor	1.00	NO	NO		Not listed in Medical Record	YES	YES	NO	DU	Supratentorial, Frontal Lobe	Central nervous system	A	NO	LIVING	2013.00
149	NO	No	C71.0	18323.00	C71.0	YES			340.00		NO		Right			NO			NO		NO	G2	YES	FALSE	No	TCGA-DU-A76K.E30A6271-8007-4457-A48E-	A76K		WITH TUMOR			Progressive Disease	YES	Primary Tumor	1.00	NO	NO		Not listed in Medical Record	NO	NO	YES	DU	Supratentorial, Parietal Lobe	Central nervous system	A	NO	DECEASED	1999.00
150	NO	No	C71.0	18688.00	C71.0	YES			290.00		NO		Right			NO			NO		NO	G3	YES	FALSE	No	TCGA-DU-A76L.E66BD7A0-E6D5-4773-9718-	A76L		WITH TUMOR			Progressive Disease	YES	Primary Tumor	1.00	NO	NO		Not listed in Medical Record	YES	NO	YES	DU	Supratentorial, Temporal Lobe	Central nervous system	A	NO	DECEASED	2000.00
151	NO	No	C71.0	36586.00	C71.0	YES	NO		150.00		NO		Left	NO	IHC	YES		YES	NO	NO	NO	G2	NO	FALSE	Yes, History of Prior Malignancy	TCGA-DU-A76O.64ED6980-61B9-4F1C-B7EE-	A76O		WITH TUMOR	YES	NO		YES	Primary Tumor	1.00	YES	NO		Not listed in Medical Record	YES	YES	NO	DU	Supratentorial, Frontal Lobe	Central nervous system	A	NO	LIVING	2013.00
152	NO	No	C71.0	18688.00	C71.0	YES			250.00		NO	50.00	Right			NO			YES		YES	G3	YES	FALSE	No	TCGA-DU-A76R.7D334156-3FCB-4C90-AD99-	A76R	Pre-Adjuvant Therapy	WITH TUMOR	YES	YES	Progressive Disease	YES	Primary Tumor	1.00	NO	NO		Not listed in Medical Record	NO	NO	YES	DU	Supratentorial, Frontal Lobe	Central nervous system	A	NO	DECEASED	2006.00
153	NO	No	C71.0	18688.00	C71.0	YES			250.00		NO	60.00	Right			NO			NO		NO	G3		FALSE	No	TCGA-DU-A7T6.B14319BC-DB5D-41E4-9954-	A7T6	Pre-Adjuvant Therapy	WITH TUMOR					Primary Tumor	1.00	NO	NO		Not listed in Medical Record		NO	YES	DU	Supratentorial, Frontal Lobe	Central nervous system	A	NO	DECEASED	2002.00
154	NO	No	C71.0	30011.00	C71.0	YES			280.00		NO	100.00	Right			NO		NO	YES	NO	YES	G3	YES	FALSE	No	TCGA-DU-A7T8.342A92BA-BEF9-40C6-85FF-	A7T8	Other	WITH TUMOR			Progressive Disease	YES	Primary Tumor	1.00	NO	YES		Not listed in Medical Record	YES	NO	YES	DU	Supratentorial, Frontal Lobe	Central nervous system	A	NO	DECEASED	2003.00
155	NO	No	C71.0	18323.00	C71.0	YES			380.00		NO	80.00	Right			NO		NO	NO	NO	NO	G2	NO	FALSE	No	TCGA-DU-A7TA.4E9DD63D-7C18-4922-93B5-	A7TA	Pre-Adjuvant Therapy	TUMOR FREE	NO	NO	Stable Disease	YES	Primary Tumor	1.00	NO	NO		Not listed in Medical Record	YES	NO	YES	DU	Supratentorial, Occipital Lobe	Central nervous system	A	YES	LIVING	2007.00
156	NO	No	C71.0	18323.00	C71.0	YES			250.00		NO	100.00	Right			NO		NO	NO		NO	G2	YES	FALSE	No	TCGA-DU-A7TB.3D167AD9-276F-47F4-9C77-	A7TB	Post-Adjuvant Therapy	TUMOR FREE	YES	YES	Progressive Disease	NO	Primary Tumor	1.00	YES	NO		Not listed in Medical Record	YES	YES	NO	DU	Supratentorial, Temporal Lobe	Central nervous system	A	NO	LIVING	2010.00
157		No	C71.0	36586.00	C71.0	YES						90.00	Right			NO		NO	YES	NO	NO	G2	YES		No		A7TC	[Discrepancy]	WITH TUMOR	NO	NO	[Discrepancy]	NO	Primary Tumor	1.00	NO	NO		Not listed in Medical Record	NO	YES	NO	DU	Supratentorial, Frontal Lobe	Central nervous system		NO	LIVING	2011.00
158	NO	No	C71.0	30011.00	C71.0	YES			530.00		NO	60.00	Right			NO			NO		YES	G3	YES	FALSE	No	TCGA-DU-A7TD.0DDAB8E8-54DA-495A-9A4B-	A7TD	Post-Adjuvant Therapy	WITH TUMOR	YES	YES	Progressive Disease	YES	Primary Tumor	1.00	NO	NO			YES	YES	NO	DU	Supratentorial, Temporal Lobe	Central nervous system	A	NO	DECEASED	2012.00
159	NO	No	C71.0	18323.00	C71.0	YES			300.00		NO	70.00	Right			NO			YES		NO	G2		FALSE	No	TCGA-DU-A7TG.B8C5F2FE-1618-4AB8-924E-	A7TG	Post-Adjuvant Therapy					NO	Primary Tumor	1.00	NO	NO		Not listed in Medical Record	YES	NO	YES	DU	Supratentorial, Frontal Lobe	Central nervous system	A	NO	DECEASED	2008.00
160		No	C71.0	43891.00	C71.0	YES			180.00		NO		Left			NO			YES		NO	G3	YES	FALSE	No	TCGA-DU-A7TI.F365952B-D839-4B07-953A-	A7TI		WITH TUMOR	YES		Progressive Disease	YES	Primary Tumor	1.00	YES	NO		Cerebral Cortex	YES	NO	YES	DU	Supratentorial, Frontal Lobe	Central nervous system	A	NO	DECEASED	1998.00
161	NO	No	C71.0	43891.00	C71.0	YES	NO		470.00		NO		Right	YES	IHC	YES		YES				G3		FALSE	No	TCGA-DU-A7TJ.039F34B6-70E4-42E2-A93C-	A7TJ		WITH TUMOR	YES	YES			Primary Tumor	1.00	YES			Not listed in Medical Record		YES	NO	DU	Supratentorial, Temporal Lobe	Central nervous system	A		LIVING	2013.00
162	NO	No	C71.9	43891.00	C71.9	YES				0.70	NO	100.00	Left			NO	1.10		NO		NO	G3	YES		No	TCGA-E1-5302.cd1ef6bc-41bf-4b2c-80dc-	5302.00	Post-Adjuvant Therapy	WITH TUMOR	YES	YES	Stable Disease	YES	Primary Tumor	1.00	YES	NO	0.30	Not listed in Medical Record	YES	NO	YES	E1	Supratentorial, Frontal Lobe	Central nervous system	A	NO	DECEASED	1998.00
163	NO	No	C71.9	43891.00	C71.9	YES	NO			0.60	NO	80.00	Left				0.80		NO		NO	G3	YES		No	TCGA-E1-5303.20e5b4f2-6555-4bf9-80c3-	5303.00	Post-Adjuvant Therapy	WITH TUMOR	YES	YES	Progressive Disease	YES	Primary Tumor	1.00	YES	NO	0.60	Not listed in Medical Record	[Discrepancy]	NO	YES	E1	Supratentorial, Frontal Lobe	Central nervous system	A	NO	DECEASED	1998.00
164	NO	No	C71.9	43891.00	C71.9	YES				0.60	NO	90.00	Right				0.80		NO	NO	NO	G3	YES		No	TCGA-E1-5304.4180deb2-569a-49d5-9b0c-	5304.00	Post-Adjuvant Therapy	WITH TUMOR	YES	YES	Stable Disease	YES	Primary Tumor	1.00	NO	YES	0.60	Not listed in Medical Record	YES	NO	YES	E1	Supratentorial, Frontal Lobe	Central nervous system	A	NO	DECEASED	1999.00
165	NO	No	C71.9	43891.00	C71.9	YES				0.80	NO	90.00	Right				1.00		NO	YES	NO	G3	YES		No	TCGA-E1-5305.7c854147-edeb-47ca-9b46-	5305.00	Post-Adjuvant Therapy	WITH TUMOR	YES	YES	Stable Disease	YES	Primary Tumor	1.00	YES	NO	0.40	Not listed in Medical Record	[Discrepancy]	NO	YES	E1	Supratentorial, Frontal Lobe	Central nervous system	A	NO	DECEASED	2000.00
166	NO	No	C71.9	43891.00	C71.9	YES				1.00	NO	80.00	Left				1.30		YES		NO	G3	YES		No	TCGA-E1-5307.60b202db-3c7c-4c86-be48-	5307.00	Pre-Adjuvant Therapy	WITH TUMOR	YES	YES	Stable Disease	YES	Primary Tumor	1.00	NO	NO	0.60	Not listed in Medical Record	YES	NO	YES	E1	Supratentorial, Temporal Lobe	Central nervous system	A	NO	DECEASED	2000.00
167	NO	No	C71.9	18688.00	C71.9	YES				0.60	NO	100.00	Right				1.10		NO	NO	NO	G3	YES		No	TCGA-E1-5311.f4d55755-105e-4740-b13e-	5311.00	Pre-Adjuvant Therapy	WITH TUMOR	YES	YES	Stable Disease	YES	Primary Tumor	1.00	NO	NO	0.20	Not listed in Medical Record	YES	NO	YES	E1	Supratentorial, Temporal Lobe	Central nervous system	A	NO	DECEASED	1994.00
168	NO	No	C71.9	18323.00	C71.9	YES				0.50	NO	100.00	Left				0.70		NO	NO	NO	G2	YES		No	TCGA-E1-5318.9a5db4fd-190b-448c-a22a-	5318.00	Pre-Adjuvant Therapy	WITH TUMOR	YES	YES	Progressive Disease	YES	Primary Tumor	1.00	NO	NO	0.20	Not listed in Medical Record	YES	NO	YES	E1	Supratentorial, Frontal Lobe	Central nervous system	A	NO	DECEASED	1997.00
169	NO	No	C71.9	18323.00	C71.9	YES				0.80	NO	100.00	Right				1.00		NO		NO	G2	YES		No	TCGA-E1-5319.31d5346f-9f29-4eee-86e4-	5319.00	Pre-Adjuvant Therapy	WITH TUMOR	YES	YES	Stable Disease	NO	Primary Tumor	1.00	NO	NO	0.50	Not listed in Medical Record	NO	NO	YES	E1	Supratentorial, Frontal Lobe	Central nervous system	A	YES	DECEASED	1998.00
170	NO	No	C71.9	30011.00	C71.9	YES				0.40	NO	90.00	Right				0.70					G2	YES		No	TCGA-E1-5322.68735214-8bff-497f-8157-	5322.00	Post-Adjuvant Therapy		YES	YES	Stable Disease	[Discrepancy]	Primary Tumor	1.00	YES		0.20	Not listed in Medical Record	[Discrepancy]	NO	YES	E1	Supratentorial, Temporal Lobe	Central nervous system	A		DECEASED	1999.00
171	NO	No	C71.0	43891.00	C71.0	YES	NO		80.00		NO		Left			NO			NO		NO	G3	YES	TRUE	No	TCGA-E1-A7YD.B371FF08-C874-4A6E-9CF9-	A7YD		WITH TUMOR	YES		Stable Disease	YES	Primary Tumor	1.00	YES	NO		Not listed in Medical Record	YES	NO	YES	E1	Supratentorial, Temporal Lobe	Central nervous system	A	NO	DECEASED	1998.00
172	NO	No	C71.0	43891.00	C71.0	YES						90.00	Left			NO		NO	NO	NO	NO	G3	YES		No		A7YE	Post-Adjuvant Therapy	WITH TUMOR	YES	NO	Stable Disease	YES	Primary Tumor	1.00	YES	YES		Not listed in Medical Record	YES	NO	YES	E1	Supratentorial, Frontal Lobe	Central nervous system		NO	DECEASED	2001.00
173	NO	No	C71.0	43891.00	C71.0	YES	NO		130.00		NO	80.00	Right			NO		NO	NO		NO	G3	YES	TRUE	No	TCGA-E1-A7YH.4E589046-F21D-4822-8243-	A7YH	Pre-Adjuvant Therapy	WITH TUMOR	YES	NO	Stable Disease	YES	Primary Tumor	1.00	YES	NO		White Matter	YES	NO	YES	E1	Supratentorial, Temporal Lobe	Central nervous system	A	NO	DECEASED	2005.00
174	NO	No	C71.0	43891.00	C71.0	YES	NO		330.00		NO	80.00	Right			NO			NO		YES	G3	YES	TRUE	No	TCGA-E1-A7YI.17841D2A-99A8-4F1D-900B-	A7YI	Pre-Adjuvant Therapy	WITH TUMOR	YES			NO	Primary Tumor	1.00	NO	YES		Not listed in Medical Record	NO	NO	YES	E1	Supratentorial, Occipital Lobe	Central nervous system	A	YES	DECEASED	2008.00
175	NO	No	C71.0	43891.00	C71.0	YES	NO		190.00		NO	80.00	Right			NO		NO	NO		YES	G3	YES	TRUE	No	TCGA-E1-A7YJ.CF66C60F-354C-4BB5-9A07-	A7YJ	Pre-Adjuvant Therapy	WITH TUMOR	YES	YES	Stable Disease	YES	Primary Tumor	1.00	NO	NO		White Matter	YES	NO	YES	E1	Supratentorial, Frontal Lobe	Central nervous system	A	NO	DECEASED	2008.00
176	NO	No	C71.0	43891.00	C71.0	YES						90.00	Left			NO			NO		NO	G3	NO		No		A7YK	Pre-Adjuvant Therapy		YES	YES	Stable Disease	YES	Primary Tumor	1.00	YES	NO		Cerebral Cortex	YES	NO	YES	E1	Supratentorial, Temporal Lobe	Central nervous system		NO	DECEASED	2009.00
177	NO	No	C71.0	43891.00	C71.0	YES	NO		150.00		NO	80.00	Right	NO	IHC	YES		NO	NO	NO	NO	G3	YES	TRUE	No	TCGA-E1-A7YL.4965D7EE-815C-40D9-8071-	A7YL	Pre-Adjuvant Therapy	WITH TUMOR	YES	YES	Progressive Disease	YES	Primary Tumor	1.00	NO	YES		Not listed in Medical Record	YES	NO	YES	E1	Supratentorial, Frontal Lobe	Central nervous system	A	YES	DECEASED	2009.00
178	NO	No	C71.0	43891.00	C71.0	YES	NO		180.00		NO	80.00	Left	YES	IHC	YES		NO	YES	NO	NO	G3	YES	TRUE	No	TCGA-E1-A7YM.FC2E7F8E-0C21-457D-92A1-	A7YM	Pre-Adjuvant Therapy	WITH TUMOR	NO	YES	Progressive Disease	YES	Primary Tumor	1.00	YES	NO		Not listed in Medical Record	YES	NO	YES	E1	Supratentorial, Temporal Lobe	Central nervous system	A	NO	DECEASED	2010.00

179	NO	No	C71.0	43891.00	C71.0	YES	NO		140.00		NO	80.00	Right	NO	IHC	YES		NO	NO	NO	NO	G3	YES	TRUE	Yes, History of Prior Malignancy	TCGA-E1-A7YN.2C9A41BE-767B-4929-9186-	A7YN	Pre-Adjuvant Therapy	WITH TUMOR	YES	YES	Stable Disease	YES	Primary Tumor	1.00	NO	YES		Not listed in Medical Record	YES	NO	YES	E1	Supratentorial, Temporal Lobe	Central nervous system	A	YES	DECEASED	2010.00
180	NO	No	C71.0	18688.00	C71.0	YES	NO		50.00		NO	100.00	Right			NO		NO	NO	NO	NO	G3	YES	TRUE	No	TCGA-E1-A7YO.3A005836-F7CF-4423-8548-	A7YO	Post-Adjuvant Therapy	WITH TUMOR	YES		Stable Disease	NO	Primary Tumor	1.00	YES	NO		Not listed in Medical Record	YES	NO	YES	E1	Supratentorial, Frontal Lobe	Central nervous system	A	NO	DECEASED	1995.00
181	NO	No	C71.0	18688.00	C71.0	YES	NO		180.00		NO	60.00	Right			NO		NO	NO	NO	NO	G3	YES	TRUE	No	TCGA-E1-A7YQ.FECABDF2-3EBF-4C3C-	A7YQ	Post-Adjuvant Therapy	WITH TUMOR	YES	YES	Stable Disease	YES	Primary Tumor	1.00	NO	NO		White Matter	YES	NO	YES	E1	Supratentorial, Temporal Lobe	Central nervous system	A	YES	DECEASED	2000.00
182	NO	No	C71.0	18688.00	C71.0	YES	NO		180.00		NO	80.00	Left			NO		NO	YES	NO		G3	NO	TRUE	No	TCGA-E1-A7YS.8C44E399-41A8-455E-86EA-	A7YS	Pre-Adjuvant Therapy	WITH TUMOR	YES	YES	Stable Disease	YES	Primary Tumor	1.00	NO	NO		Not listed in Medical Record	YES	NO	YES	E1	Supratentorial, Temporal Lobe	Central nervous system	A	NO	DECEASED	2007.00
183	NO	No	C71.0	30011.00	C71.0	YES	NO									NO			NO		NO	G3			No		A7YU		WITH TUMOR	YES			NO	Primary Tumor	1.00		YES		Not listed in Medical Record	YES	NO	YES	E1	Supratentorial, Frontal Lobe	Central nervous system		NO	DECEASED	1996.00
184	NO	No	C71.0	30011.00	C71.0	YES	YES	3 X chromosomes (super female)	230.00		NO	90.00	Left	YES	IHC	YES		NO	NO	NO	NO	G3	YES	TRUE	No	TCGA-E1-A7YV.96974491-20AD-491B-A475-	A7YV	Pre-Adjuvant Therapy	WITH TUMOR	NO	YES	Progressive Disease	YES	Primary Tumor	1.00	NO	NO		Not listed in Medical Record	YES	NO	YES	E1	Supratentorial, Frontal Lobe	Central nervous system	A	NO	DECEASED	2010.00
185	NO	No	C71.0	30011.00	C71.0	YES	NO		100.00		NO	90.00	Left			NO		NO	NO	NO	NO	G2	YES	TRUE	No	TCGA-E1-A7YW.821A037A-B968-4A91-9D3F-	A7YW	Pre-Adjuvant Therapy	WITH TUMOR	YES		Progressive Disease	YES	Primary Tumor	1.00	YES	NO		Cerebral Cortex	YES	NO	YES	E1	Supratentorial, Parietal Lobe	Central nervous system	A	NO	DECEASED	2002.00
186	NO	No	C71.0	18323.00	C71.0	YES	NO		140.00		NO	100.00	Right			NO		NO	NO	NO	NO	G2	YES	TRUE	No	TCGA-E1-A7YY.EBCF38D8-E69D-4F12-B7F5-	A7YY	Pre-Adjuvant Therapy	WITH TUMOR	YES	YES	Stable Disease	YES	Primary Tumor	1.00	YES	NO		Not listed in Medical Record	YES	NO	YES	E1	Supratentorial, Frontal Lobe	Central nervous system	A	NO	DECEASED	1998.00
187	NO	No	C71.0	18323.00	C71.0	YES	NO		160.00		NO	80.00	Left	NO	IHC	YES		NO	NO	NO	NO	G2	YES	TRUE	No	TCGA-E1-A7Z2.2729C641-D25B-4C92-94C8-	A7Z2	Pre-Adjuvant Therapy	WITH TUMOR	YES	YES	Progressive Disease	NO	Primary Tumor	1.00	YES	NO		Cerebral Cortex	YES	NO	YES	E1	Supratentorial, Frontal Lobe	Central nervous system	A	NO	DECEASED	2012.00
188	NO	No	C71.0	36586.00	C71.0	YES	NO		260.00		NO		Right			NO		NO	NO		NO	G2	YES	TRUE	No	TCGA-E1-A7Z3.A522B02B-1349-44FD-9AEA-	A7Z3		WITH TUMOR	YES		Progressive Disease	YES	Primary Tumor	1.00	YES	NO		Cerebral Cortex	YES	NO	YES	E1	Supratentorial, Parietal Lobe	Central nervous system	A	NO	DECEASED	1992.00
189	NO	No	C71.0	36586.00	C71.0	YES	NO					90.00	Left	YES	IHC	YES		NO	NO	NO	NO	G2	YES		No		A7Z4	Post-Adjuvant Therapy	WITH TUMOR	YES	YES	Progressive Disease	YES	Primary Tumor	1.00	NO	NO		Not listed in Medical Record	YES	NO	YES	E1	Supratentorial, Frontal Lobe	Central nervous system		YES	DECEASED	1999.00
190	NO	No	C71.0	36586.00	C71.0	YES	NO		160.00		NO	90.00	Right	YES	IHC	YES		NO	NO	NO	NO	G2	YES	TRUE	No	TCGA-E1-A7Z6.096B03E5-82A9-41E6-BC6D-	A7Z6	Pre-Adjuvant Therapy	WITH TUMOR	NO	YES	Progressive Disease	YES	Primary Tumor	1.00	NO	NO		Not listed in Medical Record	YES	NO	YES	E1	Supratentorial, Frontal Lobe	Central nervous system	A	NO	DECEASED	2009.00
191	NO	No	C71.0	18323.00	C71.0	YES				0.90	NO		Right				1.00	NO	YES	NO	NO	G2	NO		No	TCGA-EZ-7264.1a18641e-75f5-4601-8d9b-	7264.00		WITH TUMOR	YES	YES	Stable Disease	NO	Primary Tumor	1.00	YES	NO	0.50	White Matter	NO	YES	NO	EZ	Supratentorial, Frontal Lobe	Central nervous system	A	NO	LIVING	2011.00
192	NO	No	C71.0	18323.00	C71.0	YES	NO		550.00		NO					NO		YES	NO	NO	NO	G2	NO	TRUE	No	TCGA-F6-A8O3.79D96BBD-E399-4928-A86A-	A8O3		TUMOR FREE	NO	NO	Complete Remission/Response		Primary Tumor	1.00	NO	NO		Not listed in Medical Record		YES	NO	F6	Supratentorial, Frontal Lobe	Central nervous system	A	NO	LIVING	2013.00
193	NO	No	C71.9	36586.00	C71.9	YES	NO		970.00		NO		Left			NO		YES	NO	NO	NO	G2	NO	TRUE	No	TCGA-F6-A8O4.4CDE1CF9-1B5E-4838-8417-	A8O4		TUMOR FREE	NO	NO	Complete Remission/Response		Primary Tumor	1.00	NO	NO		Not listed in Medical Record		YES	NO	F6	Supratentorial, Frontal Lobe	Central nervous system	A	NO	LIVING	2013.00
194	NO	No	C71.0	18688.00	C71.0	YES	NO			1.00	NO		Left			NO	1.00	NO	NO	NO	NO	G3	NO		No	TCGA-FG-5962.1ed7f628-f222-41e4-a83c-	5962.00		WITH TUMOR	NO	NO	Stable Disease	YES	Primary Tumor	1.00	NO	NO	0.50	White Matter	YES	NO	YES	FG	Supratentorial, Occipital Lobe	Central nervous system	B	YES	LIVING	2009.00
195	NO	No	C71.0	43891.00	C71.0	YES	NO			0.90	NO	90.00	Right			NO	0.90	NO	YES	YES	NO	G3	YES		No	TCGA-FG-5963.6BDCE0AC-CDC9-45A2-B796-	5963.00	Post-Adjuvant Therapy	WITH TUMOR	YES		Progressive Disease	YES	Primary Tumor	1.00	YES	NO	0.70	White Matter	YES	NO	YES	FG	Supratentorial, Frontal Lobe	Central nervous system	A	NO	DECEASED	2009.00
196	NO	No	C71.0	43891.00	C71.0	YES	NO		130.00		NO	90.00	Right			NO		NO	YES	YES	NO	G3	YES	FALSE	No	TCGA-FG-5963.6BDCE0AC-CDC9-45A2-B796-	5963.00	Post-Adjuvant Therapy	WITH TUMOR	YES		Progressive Disease	YES	Recurrent Tumor	2.00	YES	NO		White Matter	YES	NO	YES	FG	Supratentorial, Frontal Lobe	Central nervous system	A	NO	DECEASED	2009.00
197	NO	No	C71.0	18323.00	C71.0	YES	NO			0.70	NO		Left			NO	0.70	NO	NO	YES	NO	G2	NO		No	TCGA-FG-5964.bcaff494-0ef3-42b0-8d93-	5964.00		WITH TUMOR	YES	YES	Stable Disease	YES	Primary Tumor	1.00	YES	NO	0.60	White Matter	NO	NO	YES	FG	Supratentorial, Parietal Lobe	Central nervous system	A	NO	LIVING	2010.00
198	NO	No	C71.0	30011.00	C71.0	YES	NO			1.00	NO	90.00	Right			NO	1.10		NO	NO	NO	G2	YES		No		5965.00	Post-Adjuvant Therapy	WITH TUMOR	NO	NO	Progressive Disease	NO	Primary Tumor	1.00	YES	NO	0.80	White Matter	YES	NO	YES	FG	Supratentorial, Frontal Lobe	Central nervous system	B	YES	DECEASED	2008.00
199	NO	No	C71.0	30011.00	C71.0	YES	NO		120.00		NO	90.00	Right			NO			NO	NO	NO	G2	YES	FALSE	No	TCGA-FG-5965.DD2349DD-E128-4C2D-962B-	5965.00	Post-Adjuvant Therapy	WITH TUMOR	NO	NO	Progressive Disease	NO	Recurrent Tumor	2.00	YES	NO		White Matter	YES	NO	YES	FG	Supratentorial, Frontal Lobe	Central nervous system	B	YES	DECEASED	2008.00
200	NO	No	C71.0	43891.00	C71.0	YES	NO			1.00	NO	50.00	Left			NO	1.00		NO	NO	NO	G3	YES		No	TCGA-FG-6688.b889306a-615a-4c0e-8acd-	6688.00	Post-Adjuvant Therapy	WITH TUMOR	YES	YES	Progressive Disease	YES	Primary Tumor	1.00	YES	YES	0.80	Not listed in Medical Record	YES	YES	NO	FG	Supratentorial, Parietal Lobe	Central nervous system	A	YES	LIVING	2010.00
201	NO	No	C71.0	36586.00	C71.0	YES	NO			1.00	NO	70.00	Right			NO	1.00	YES	YES	YES	NO	G2	YES		No	TCGA-FG-6689.cc32ee60-c475-48dd-acd3-	6689.00	Preoperative	WITH TUMOR	NO	YES	Progressive Disease	YES	Primary Tumor	1.00	NO	NO	1.00	Not listed in Medical Record	NO	YES	NO	FG	Supratentorial, Frontal Lobe	Central nervous system	A	YES	LIVING	2011.00
202	NO	No	C71.0	18323.00	C71.0	YES	NO			0.60	NO	90.00	Left			NO	1.70	NO	NO	NO	NO	G2	NO		No	TCGA-FG-6690.d4c3d026-59a3-4b34-9d49-	6690.00	Preoperative	WITH TUMOR	YES	YES	Stable Disease	YES	Primary Tumor	1.00	YES	NO	0.50	Not listed in Medical Record	NO	YES	NO	FG	Supratentorial, Frontal Lobe	Central nervous system	A	NO	LIVING	2011.00
203	NO	No	C71.0	36586.00	C71.0	YES	NO			0.90	NO	100.00	Right			NO	1.10	NO	NO	NO	NO	G2	YES		No	TCGA-FG-6691.799a7cc3-b435-4208-9996-	6691.00	Preoperative	WITH TUMOR	NO	NO	Complete Remission/Response	NO	Primary Tumor	1.00	NO	NO	0.90	White Matter	NO	YES	NO	FG	Supratentorial, Temporal Lobe	Central nervous system	A	NO	LIVING	2011.00
204	NO	No	C71.0	18688.00	C71.0	YES	NO						Right			NO		NO	YES		NO	G3			No		6692.00	Pre-Adjuvant Therapy	WITH TUMOR	YES	NO	Stable Disease	YES	Primary Tumor	1.00	YES	NO		Not listed in Medical Record	YES	YES	NO	FG	Supratentorial, Frontal Lobe	Central nervous system		NO	DECEASED	2011.00
205	NO	No	C71.0	18323.00	C71.0	YES	NO			0.90	NO		Left			NO	1.00	YES	NO	NO	NO	G2			No	TCGA-FG-7634.b6184ded-a667-44ea-a0d3-	7634.00		WITH TUMOR	YES	NO	Stable Disease		Primary Tumor	1.00	YES	NO	0.60	Not listed in Medical Record		YES	NO	FG	Supratentorial, Frontal Lobe	Central nervous system	A	NO	LIVING	2008.00
206	NO	No	C71.0	43891.00	C71.0	YES	NO			0.60	NO	90.00	Left			NO	1.10	NO	YES	NO	YES	G3	NO		No	TCGA-FG-7636.f257e22a-45d4-4c1d-b18e-	7636.00	Preoperative	TUMOR FREE	YES	YES	Complete Remission/Response	YES	Primary Tumor	1.00	YES	YES	0.40	Not listed in Medical Record	YES	YES	NO	FG	Supratentorial, Frontal Lobe	Central nervous system	A	NO	LIVING	2011.00
207	NO	No	C71.0	30011.00	C71.0	YES	NO			0.60	NO		Right			NO	1.00	NO	YES	NO	NO	G2	NO		No	TCGA-FG-7637.9fdc723f-1cba-4660-8b6e-	7637.00		WITH TUMOR	YES	NO	Stable Disease	NO	Primary Tumor	1.00	YES	NO	0.50	Not listed in Medical Record	NO	YES	NO	FG	Supratentorial, Temporal Lobe	Central nervous system	A	NO	LIVING	2009.00
208	NO	No	C71.0	18688.00	C71.0	YES	NO			1.00	NO	100.00	Left			NO	1.30	NO	NO	NO	YES	G3	YES		No	TCGA-FG-7638.2a94afdc-2504-4b73-8b1e-	7638.00	Preoperative	WITH TUMOR	YES	YES	Stable Disease	YES	Primary Tumor	1.00	YES	NO	0.60	Not listed in Medical Record	YES	YES	NO	FG	Supratentorial, Frontal Lobe	Central nervous system	B	NO	LIVING	2011.00
209	NO	No	C71.0	18323.00	C71.0	YES	NO			0.90	NO		Left			NO	1.00	NO	NO	NO	NO	G2	NO		No	TCGA-FG-7641.0f5772cf-b2c8-4872-a05d-	7641.00		TUMOR FREE	NO	YES	Complete Remission/Response	NO	Primary Tumor	1.00	NO	NO	0.60	Not listed in Medical Record	NO	YES	NO	FG	Supratentorial, Frontal Lobe	Central nervous system	B	YES	LIVING	2011.00
210	NO	No	C71.0	30011.00	C71.0	YES	NO			0.80	NO		Right			NO	1.30	NO	NO	YES	NO	G2	YES		No	TCGA-FG-7643.e6f58f35-8608-4620-b170-	7643.00		WITH TUMOR	YES	NO	Progressive Disease	NO	Primary Tumor	1.00	YES	NO	0.60	Cerebral Cortex	NO	YES	NO	FG	Supratentorial, Frontal Lobe	Central nervous system	A	NO	LIVING	2011.00
211	NO	No	C71.0	30011.00	C71.0	YES	NO			1.70	NO	90.00	Left			NO	1.90	NO				G3	NO		No	TCGA-FG-8181.6b2e8993-f8a8-400a-bc49-	8181.00	Preoperative	WITH TUMOR			Stable Disease	YES	Primary Tumor	1.00			0.70	Not listed in Medical Record	YES	YES	NO	FG	Supratentorial, Frontal Lobe	Central nervous system	A		LIVING	2012.00
212	NO	No	C71.0	18323.00	C71.0	YES	NO			1.10	NO		Left			NO	1.40	NO	NO	NO	NO	G2	NO		No	TCGA-FG-8182.e5be5e58-2293-4328-a626-	8182.00		WITH TUMOR			Stable Disease	NO	Primary Tumor	1.00	YES	NO	0.30	Not listed in Medical Record	NO	YES	NO	FG	Supratentorial, Frontal Lobe	Central nervous system	A	NO	LIVING	2012.00
213	NO	No	C71.0	43891.00	C71.0	YES	NO			0.90	NO	90.00	Right				1.30	NO	NO	NO	NO	G3	NO		No	TCGA-FG-8185.74a335ae-a23d-4cc8-abb1-	8185.00	Pre-Adjuvant Therapy	TUMOR FREE			Complete Remission/Response	YES	Primary Tumor	1.00	NO	NO	0.30	White Matter	YES	YES	NO	FG	Supratentorial, Frontal Lobe	Central nervous system	A	YES	LIVING	2012.00
214	NO	No	C71.0	30011.00	C71.0	YES	NO			1.00	NO	70.00	Right			NO	1.50	NO	NO	NO	NO	G3	NO		No	TCGA-FG-8186.7e694f9c-b221-40a9-a63a-	8186.00	Pre-Adjuvant Therapy	WITH TUMOR	YES	NO	Stable Disease	YES	Primary Tumor	1.00	YES	NO	0.90	Not listed in Medical Record	NO	YES	NO	FG	Supratentorial, Frontal Lobe	Central nervous system	A	NO	LIVING	2011.00

215	NO	No	C71.0	30011.00	C71.0	YES	NO			1.20	NO	90.00	Right			NO	1.40	NO				G2	NO		Yes	TCGA-FG-8187.7fee5178-b1ff-4d7c-beed-	8187.00	Other	TUMOR FREE			Complete Remission/Response	NO	Primary Tumor	1.00			0.70	Not listed in Medical Record	NO	YES	NO	FG	Supratentorial, Frontal Lobe	Central nervous system	A		LIVING	2011.00
216	NO	No	C71.0	30011.00	C71.0	YES	NO			0.60	NO	90.00	Left			NO	1.10	NO	YES	NO	NO	G2	YES		No	TCGA-FG-8188.7f0c279e-aecb-4bb2-925b-	8188.00	Preoperative	WITH TUMOR	YES		Stable Disease	NO	Primary Tumor	1.00	NO	NO	0.60	Not listed in Medical Record	NO	YES	NO	FG	Supratentorial, Temporal Lobe	Central nervous system	A	NO	LIVING	2012.00
217	NO	No	C71.0	18323.00	C71.0	YES	NO		370.00		NO	70.00	Right			NO		NO	NO	NO	NO	G2	NO	FALSE	No	TCGA-FG-8189.0645C7FA-38CE-48A5-B873-	8189.00	Pre-Adjuvant Therapy	WITH TUMOR	YES	NO	Stable Disease	YES	Primary Tumor	1.00	YES	NO		Not listed in Medical Record	YES	YES	NO	FG	Supratentorial, Temporal Lobe	Central nervous system	B	NO	LIVING	2012.00
218	NO	No	C71.0	18688.00	C71.0	YES	NO			0.70	NO		Right			NO	1.30	NO	NO	NO	NO	G3	NO		No	TCGA-FG-8191.33d30363-b7fb-479f-a985-	8191.00		WITH TUMOR			Stable Disease	YES	Primary Tumor	1.00	NO	YES	0.70	Not listed in Medical Record	YES	YES	NO	FG	Supratentorial, Frontal Lobe	Central nervous system	A	NO	LIVING	2011.00
219	NO	No	C71.2	18323.00	C71.2	YES	NO		520.00		NO	100.00	Left			NO		NO	NO	NO	NO	G2	YES	FALSE	No	TCGA-FG-A4MT.5FBDAD09-6FC1-41FF-9361-	A4MT	Pre-Adjuvant Therapy	TUMOR FREE	YES	YES	Complete Remission/Response	NO	Primary Tumor	1.00	YES	NO		Not listed in Medical Record	NO	YES	NO	FG	Supratentorial, Temporal Lobe	Central nervous system	A	NO	LIVING	2010.00
220	NO	No	C71.2	18323.00	C71.2	YES	NO		250.00		NO	100.00	Left			NO		NO	NO	NO	NO	G2	YES	FALSE	No	TCGA-FG-A4MT.592AF347-5CB0-43E7-8950-	A4MT	Pre-Adjuvant Therapy	TUMOR FREE	YES	YES	Complete Remission/Response	NO	Recurrent Tumor	2.00	YES	NO		Not listed in Medical Record	NO	YES	NO	FG	Supratentorial, Temporal Lobe	Central nervous system	A	NO	LIVING	2010.00
221	NO	No	C71.0	30011.00	C71.0	YES	NO		700.00		NO		Right	NO	IHC	YES		NO	YES	NO	NO	G3		FALSE	No	TCGA-FG-A4MU.25B6CCE5-DF7C-483D-AA5D-	A4MU		WITH TUMOR	NO	YES			Primary Tumor	1.00	NO	NO		Not listed in Medical Record		YES	NO	FG	Supratentorial, Temporal Lobe	Central nervous system	B	YES	LIVING	2012.00
222	NO	No	C71.1	30011.00	C71.1	YES	NO		220.00		NO	80.00	Right	NO	IHC	YES		NO	NO	NO	NO	G3	YES	FALSE	No	TCGA-FG-A4MW.E9D4D450-3905-4D11-A814-	A4MW	Post-Adjuvant Therapy	WITH TUMOR	YES	YES	Stable Disease	YES	Primary Tumor	1.00	YES	NO		Not listed in Medical Record	YES	YES	NO	FG	Supratentorial, Frontal Lobe	Central nervous system	A	NO	DECEASED	2012.00
223	NO	No	C71.1	36586.00	C71.1	YES	NO		170.00		NO	100.00	Right	NO	IHC	YES		NO	NO	NO	YES	G2	NO	FALSE	No	TCGA-FG-A4MX.E88FF565-EFD8-4E5D-87BC-	A4MX	Preoperative	TUMOR FREE	NO	NO	Complete Remission/Response	YES	Primary Tumor	1.00	NO	NO		Not listed in Medical Record	NO	YES	NO	FG	Supratentorial, Frontal Lobe	Central nervous system	A	NO	LIVING	2012.00
224	NO	No	C71.3	30011.00	C71.3	YES	NO		480.00		NO		Right			NO		NO	YES	NO	NO	G2	NO	FALSE	No	TCGA-FG-A4MY.485F752A-74F7-4E4B-B921-	A4MY		WITH TUMOR			Stable Disease	YES	Primary Tumor	1.00	YES	NO		Not listed in Medical Record	NO	YES	NO	FG	Supratentorial, Parietal Lobe	Central nervous system	A	NO	LIVING	2012.00
225	NO	No	C71.0	30011.00	C71.0	YES	NO		240.00		NO	90.00	Left	YES	IHC	YES		YES	NO	NO	NO	G2	NO	FALSE	No	TCGA-FG-A60J.0EE45912-9058-4883-922E-	A60J	Preoperative	TUMOR FREE	YES	NO	Complete Remission/Response	NO	Primary Tumor	1.00	YES	NO		Not listed in Medical Record	NO	YES	NO	FG	Supratentorial, Frontal Lobe	Central nervous system	A	NO	LIVING	2012.00
226	NO	No	C71.0	30011.00	C71.0	YES	NO						Right			NO		NO	YES	NO	NO	G2	NO		Yes, History of Prior Malignancy		A60K		WITH TUMOR	NO	NO	Stable Disease	NO	Primary Tumor	1.00	NO	NO		Not listed in Medical Record	NO	YES	NO	FG	Supratentorial, Frontal Lobe	Central nervous system		NO	LIVING	2012.00
227	NO	No	C71.0	36586.00	C71.0	YES	NO		220.00		NO	100.00	Left			NO		NO	NO		NO	G2	NO	FALSE	No	TCGA-FG-A60L.E6DA1846-7274-44A3-99DE-	A60L	Other	TUMOR FREE	YES	NO	Complete Remission/Response	NO	Primary Tumor	1.00	YES	NO		Not listed in Medical Record	NO	YES	NO	FG	Supratentorial, Temporal Lobe	Central nervous system	A	NO	LIVING	2012.00
228	NO	No	C71.0	18323.00	C71.0	YES	NO		240.00		NO		Left	YES	IHC	YES		NO	NO	NO	YES	G2	NO	TRUE	No	TCGA-FG-A6IZ.999D85F3-3DE0-4A08-BCB7-	A6IZ		TUMOR FREE	YES	NO	Complete Remission/Response	YES	Primary Tumor	1.00	YES	NO		Cerebral Cortex	NO	YES	NO	FG	Supratentorial, Not Otherwise Specified	Central nervous system	A	NO	LIVING	2013.00
229	NO	No	C71.0	18323.00	C71.0	YES	NO		60.00		NO	70.00	Left	YES	IHC	YES		NO	NO	NO	NO	G2	NO	TRUE	No	TCGA-FG-A6J1.9421C492-5E69-4641-B4CD-	A6J1	Pre-Adjuvant Therapy	WITH TUMOR	NO	NO	Stable Disease	YES	Primary Tumor	1.00	YES	NO		Not listed in Medical Record	NO	YES	NO	FG	Supratentorial, Not Otherwise Specified	Central nervous system	A	NO	LIVING	2013.00
230	NO	No	C71.0	43891.00	C71.0	YES			170.00		NO	90.00	Right	YES	IHC	YES		NO	NO	NO	NO	G3	NO	TRUE	No	TCGA-FG-A6J3.480EE630-15DD-4346-84FF-	A6J3	Post-Adjuvant Therapy	WITH TUMOR	YES	NO	Progressive Disease	YES	Primary Tumor	1.00	NO	YES		Cerebral Cortex	YES	YES	NO	FG	Supratentorial, Frontal Lobe	Central nervous system	A	NO	LIVING	2012.00
231	NO	No	C71.0	18323.00	C71.0	YES	NO		170.00		NO		Left			NO		YES	NO		YES	G2	YES	FALSE	No	TCGA-FG-A70Y.C736A839-8853-478E-BB5E-	A70Y		WITH TUMOR	NO	NO	Complete Remission/Response	NO	Primary Tumor	1.00	NO	NO		Not listed in Medical Record	NO	YES	NO	FG	Supratentorial, Temporal Lobe	Central nervous system	A	YES	LIVING	2011.00
232	NO	No	C71.0	30011.00	C71.0	YES	NO		300.00		NO		Left			NO		YES	NO		NO	G3		FALSE	No	TCGA-FG-A70Z.E2908E5D-A017-41AE-AF5F-	A70Z		WITH TUMOR	NO	YES	Stable Disease	YES	Primary Tumor	1.00	NO	YES		Not listed in Medical Record	YES	YES	NO	FG	Supratentorial, Temporal Lobe	Central nervous system	A	YES	LIVING	2011.00
233	NO	No	C71.0	18323.00	C71.0	YES	NO		460.00		NO		Left			NO		NO	NO		YES	G2	NO	FALSE	No	TCGA-FG-A710.0D50F154-43CC-4916-9D8E-	A710		WITH TUMOR	YES	NO	Stable Disease	NO	Primary Tumor	1.00	YES	NO		Not listed in Medical Record	YES	YES	NO	FG	Supratentorial, Frontal Lobe	Central nervous system	A	NO	LIVING	2011.00
234	NO	No	C71.0	18323.00	C71.0	YES	NO		410.00		NO	90.00	Left			NO		NO	YES	NO	NO	G2	YES	FALSE	No	TCGA-FG-A711.5E5F767F-755E-4361-AA92-	A711	Post-Adjuvant Therapy	WITH TUMOR	YES	YES	Progressive Disease	NO	Primary Tumor	1.00	YES	YES		Not listed in Medical Record	NO	YES	NO	FG	Supratentorial, Frontal Lobe	Central nervous system	A	NO	DECEASED	2010.00
235	NO	No	C71.0	30011.00	C71.0	YES	NO		200.00		NO	60.00	Right			NO		NO	YES	YES	NO	G2	NO	FALSE	Yes, History of Prior Malignancy	TCGA-FG-A713.B44387B9-C120-4F55-87B5-	A713	Pre-Adjuvant Therapy	TUMOR FREE	NO	NO	Complete Remission/Response	YES	Primary Tumor	1.00	NO	NO		Not listed in Medical Record	NO	YES	NO	FG	Supratentorial, Frontal Lobe	Central nervous system	A	NO	LIVING	2013.00
236	NO	No	C71.0	43891.00	C71.0	YES	NO		130.00		NO		Left	YES	IHC	YES		NO		NO	YES	G3	NO	TRUE	Yes, History of Prior Malignancy	TCGA-FG-A87N.061C4D2D-340B-4F59-9B6F-	A87N		WITH TUMOR			Stable Disease	YES	Primary Tumor	1.00	YES			Not listed in Medical Record	YES	YES	NO	FG	Supratentorial, Frontal Lobe	Central nervous system	A	YES	LIVING	2013.00
237	NO	No	C71.0	43891.00	C71.0	YES	NO		250.00		NO	80.00	Right	NO	IHC	YES		YES	NO	NO	NO	G3	NO	TRUE	No	TCGA-FG-A87Q.072A2905-3E7F-4C11-87A2-	A87Q	Post-Adjuvant Therapy	TUMOR FREE	YES	YES	Complete Remission/Response	YES	Primary Tumor	1.00	YES	NO		White Matter	YES	YES	NO	FG	Supratentorial, Parietal Lobe	Central nervous system	A	NO	LIVING	2013.00
238	NO	No	C71.0	30011.00	C71.0	YES				0.30	NO		Midline				0.50	NO	YES		YES	G3	NO		No	TCGA-FN-7833.a6b649b5-cf85-4f4e-bf5f-	7833.00		TUMOR FREE			Complete Remission/Response	YES	Primary Tumor	1.00	YES	NO	0.20	Not listed in Medical Record	YES	YES	NO	FN	Supratentorial, Frontal Lobe	Central nervous system	A	NO	LIVING	2011.00
239	NO	No	C71.9	18323.00	C71.9	YES	NO			0.80	NO		Right			NO	3.00		NO	NO	YES	G2			No	TCGA-HT-7467.440fce7d-5d39-4e81-8b2d-	7467.00		TUMOR FREE	YES	NO	Complete Remission/Response		Primary Tumor	1.00	NO	YES	0.40	White Matter		NO	YES	HT	Supratentorial, Parietal Lobe	Central nervous system	A	YES	LIVING	2011.00
240	NO	No	C71.0	18688.00	C71.0	YES	NO	1p and 19Q deleted		0.70	NO	80.00	Right			NO	1.30		NO	NO	NO	G3	NO		No	TCGA-HT-7468.f56fcc2c-db3d-4ae5-b8dc-	7468.00	Post-Adjuvant Therapy	TUMOR FREE	YES	NO	Stable Disease	NO	Primary Tumor	1.00	YES	NO	0.70	White Matter	YES	NO	YES	HT	Supratentorial, Parietal Lobe	Central nervous system	A	NO	LIVING	2011.00
241	NO	No	C71.0	18688.00	C71.0	YES				1.00	NO		Left			NO	1.50			NO	YES	G3	YES		No	TCGA-HT-7469.d79ea6e7-ce40-4e9a-9e82-	7469.00		WITH TUMOR	NO	NO	Progressive Disease	YES	Primary Tumor	1.00			1.00	Cerebral Cortex	YES	NO	YES	HT	Supratentorial, Temporal Lobe	Central nervous system	A		DECEASED	2011.00
242	NO	No	C71.9	18688.00	C71.9	YES				1.00	NO	90.00	Right			NO	1.30	NO	NO	NO	NO	G3	NO		No	TCGA-HT-7470.55af0dfb-afa8-4333-a0be-	7470.00	Post-Adjuvant Therapy	TUMOR FREE	NO	NO	Progressive Disease	YES	Primary Tumor	1.00	YES	YES	0.80	White Matter	YES	NO	YES	HT	Supratentorial, Temporal Lobe	Central nervous system	A	YES	DECEASED	2011.00
243	NO	No	C71.9	18688.00	C71.9	YES				0.90	NO		Left				1.50	YES	NO	NO	YES	G3			No	TCGA-HT-7471.424d1ea1-6333-45bb-b04e-	7471.00		TUMOR FREE	YES	NO			Primary Tumor	1.00	YES	NO	0.40	Not listed in Medical Record		NO	YES	HT	Supratentorial, Temporal Lobe	Central nervous system	A	NO	LIVING	2010.00
244	NO	No	C71.0	18323.00	C71.0	YES	NO			1.00	NO		Right			NO	2.70		NO	NO	NO	G2	NO		No	TCGA-HT-7472.5842f023-208b-48dd-9c9d-	7472.00		TUMOR FREE	YES	NO	Stable Disease	NO	Primary Tumor	1.00	YES	NO	1.00	Cerebral Cortex	NO	NO	YES	HT	Supratentorial, Frontal Lobe	Central nervous system	A	NO	LIVING	2010.00
245	NO	No	C71.9	30011.00	C71.9	YES				1.20	NO		Right			NO	1.50		NO	NO	YES	G2	NO		No	TCGA-HT-7473.6f87a2ee-cb07-4d62-9f16-	7473.00		TUMOR FREE	YES	YES	Complete Remission/Response	YES	Primary Tumor	1.00	NO	NO	0.50	White Matter	NO	NO	YES	HT	Supratentorial, Temporal Lobe	Central nervous system	A	NO	LIVING	2010.00
246	NO	No	C71.0	30011.00	C71.0	YES	NO			0.90	NO		Right			NO	1.40		NO	NO	YES	G2	NO		No	TCGA-HT-7474.cef17048-e651-4025-b26e-	7474.00		TUMOR FREE	YES	NO	Stable Disease	NO	Primary Tumor	1.00	YES	NO	0.80	Not listed in Medical Record	NO	NO	YES	HT	Supratentorial, Frontal Lobe	Central nervous system	A	NO	LIVING	2011.00
247	NO	No	C71.9	30011.00	C71.9	YES	NO			1.70	NO	70.00	Right			NO	1.70		NO	NO	NO	G3	NO		No	TCGA-HT-7475.5662d09d-0abc-4365-a532-	7475.00	Post-Adjuvant Therapy	TUMOR FREE	NO	NO	Complete Remission/Response	YES	Primary Tumor	1.00	YES	NO	0.90	White Matter	YES	NO	YES	HT	Supratentorial, Frontal Lobe	Central nervous system	A	NO	LIVING	2010.00
248	NO	No	C71.0	36586.00	C71.0	YES	NO			0.70	NO		Left			NO	1.30		YES	NO	NO	G2	NO		No	TCGA-HT-7476.8b010189-b14a-458f-98b9-	7476.00		TUMOR FREE	YES	NO	Stable Disease	YES	Primary Tumor	1.00	YES	YES	0.20	Cerebral Cortex	YES	NO	YES	HT	Supratentorial, Temporal Lobe	Central nervous system	A	NO	LIVING	2011.00
249	NO	No	C71.0	43891.00	C71.0	YES			280.00		NO	80.00	Left					NO	NO	NO	YES	G3	NO	TRUE	No	TCGA-HT-7477.0BCE1BFA-C7D1-4A3E-A9DE-	7477.00	Post-Adjuvant Therapy	TUMOR FREE			Complete Remission/Response	YES	Primary Tumor	1.00	YES	NO		Not listed in Medical Record	YES	NO	YES	HT	Supratentorial, Frontal Lobe	Central nervous system	B	NO	LIVING	2011.00
250	NO	No	C71.0	36586.00	C71.0	YES	NO			0.80	NO		Left			NO	0.80		YES	NO	YES	G2	NO		No	TCGA-HT-7478.9be4b6b7-f32a-413d-adb9-	7478.00		TUMOR FREE	YES	NO	Complete Remission/Response	YES	Primary Tumor	1.00	YES	YES	0.50	White Matter	NO	NO	YES	HT	Supratentorial, Frontal Lobe	Central nervous system	A	NO	LIVING	2011.00

251	NO	No	C71.9	43891.00	C71.9	YES	NO			0.80	NO		Right			NO	1.00	NO	NO	NO	NO	G3	YES		No	TCGA-HT-7479.9bdf94b6-7e28-4c01-a392-	7479.00		TUMOR FREE	NO	NO	Complete Remission/Response	YES	Primary Tumor	1.00	NO	NO	0.60	White Matter	YES	NO	YES	HT	Supratentorial, Frontal Lobe	Central nervous system	A	NO	LIVING	2011.00
252	NO	No	C71.9	18323.00	C71.9	YES	NO			1.20	NO		Right			NO	1.80		NO	NO	YES	G2	YES		No	TCGA-HT-7480.289b59d0-0a44-4d34-a991-	7480.00		WITH TUMOR	YES	NO	Stable Disease	YES	Primary Tumor	1.00	YES	NO	0.30	Cerebral Cortex	YES	NO	YES	HT	Supratentorial, Frontal Lobe	Central nervous system	A	NO	LIVING	2005.00
253	NO	No	C71.9	18323.00	C71.9	YES	NO			1.00	NO		Right			NO	2.00	NO	NO	NO	YES	G2	YES		No	TCGA-HT-7481.879a7f04-519f-44c0-bad3-	7481.00		WITH TUMOR	YES	YES	Partial Remission/Response	YES	Primary Tumor	1.00	YES	NO	0.90	White Matter	NO	NO	YES	HT	Supratentorial, Frontal Lobe	Central nervous system	A	NO	LIVING	2006.00
254	NO	No	C71.9	30011.00	C71.9	YES	NO			1.00	NO		Left			NO	1.70	NO	NO	NO	NO	G2	YES		No	TCGA-HT-7482.79a1bd03-dd03-4d45-9bd6-	7482.00		TUMOR FREE	YES	NO	Partial Remission/Response	YES	Primary Tumor	1.00	YES	NO	1.00	Not listed in Medical Record	YES	NO	YES	HT	Supratentorial, Frontal Lobe	Central nervous system	A	NO	LIVING	2005.00
255	NO	No	C71.0	30011.00	C71.0	YES				1.00	NO		Right			NO	1.30	NO	YES	NO	YES	G2	NO		No	TCGA-HT-7483.a5c5d547-2d3d-41df-a490-	7483.00		TUMOR FREE			Complete Remission/Response	YES	Primary Tumor	1.00	YES	NO	0.50	White Matter	YES	NO	YES	HT	Supratentorial, Frontal Lobe	Central nervous system	A	NO	LIVING	1998.00
256	NO	No	C71.9	36586.00	C71.9	YES	NO			1.10	NO	100.00	Right			NO	1.60		NO	NO	YES	G2	NO		No	TCGA-HT-7485.5af016f0-9c44-4b57-8477-	7485.00	Post-Adjuvant Therapy	TUMOR FREE	NO	NO	Complete Remission/Response	NO	Primary Tumor	1.00	NO	YES	0.70	White Matter	NO	NO	YES	HT	Supratentorial, Parietal Lobe	Central nervous system	A	NO	LIVING	2006.00
257	NO	No	C71.9	43891.00	C71.9	YES	NO			1.00	NO		Left			NO	2.70		NO	NO	YES	G3	NO		No	TCGA-HT-7601.2a8341ad-9d93-4a5f-b3f8-	7601.00		TUMOR FREE	YES	NO	Partial Remission/Response	YES	Primary Tumor	1.00	YES	NO	0.90	White Matter	YES	NO	YES	HT	Supratentorial, Frontal Lobe	Central nervous system	A	NO	LIVING	2008.00
258	NO	No	C71.9	18323.00	C71.9	YES	NO			1.00	NO		Right			NO	2.80		NO	NO	NO	G2			No	TCGA-HT-7602.0ae8d27a-943e-48ac-8c70-	7602.00		TUMOR FREE				NO	Primary Tumor	1.00	NO	NO	1.00	White Matter	NO	NO	YES	HT	Supratentorial, Frontal Lobe	Central nervous system	A	NO	LIVING	2008.00
259	NO	No	C71.9	18323.00	C71.9	YES	NO			1.20	NO					NO	1.40		NO	NO	NO	G2	NO		No	TCGA-HT-7603.1bae23f1-0f8d-4dcc-b47d-	7603.00		TUMOR FREE	NO	YES	Complete Remission/Response	YES	Primary Tumor	1.00	NO	NO	0.40	White Matter	NO	NO	YES	HT	Supratentorial, Frontal Lobe	Central nervous system	A	YES	LIVING	2008.00
260	NO	No	C71.0	36586.00	C71.0	YES				1.20	NO		Left			NO	1.70	NO	YES	NO	YES	G2	NO		No	TCGA-HT-7604.ecac7160-5404-42f2-90d3-	7604.00		TUMOR FREE	YES	YES	Partial Remission/Response	YES	Primary Tumor	1.00	YES	NO	0.40	Not listed in Medical Record	YES	NO	YES	HT	Supratentorial, Frontal Lobe	Central nervous system	A	NO	LIVING	2003.00
261	NO	No	C71.9	18323.00	C71.9	YES				0.90	NO		Right			NO	2.00		NO	YES	YES	G2	NO		No	TCGA-HT-7605.820c0259-c976-48cc-af5a-	7605.00						NO	Primary Tumor	1.00	NO	NO	0.80	White Matter	NO	NO	YES	HT	Supratentorial, Frontal Lobe	Central nervous system	A	NO	LIVING	2008.00
262	NO	No	C71.9	36586.00	C71.9	YES				0.80	NO		Left				1.20		NO	NO	NO	G2	NO		No	TCGA-HT-7606.4f988b76-fc06-45af-a537-	7606.00		TUMOR FREE			Complete Remission/Response	YES	Primary Tumor	1.00	NO	NO	0.70	Not listed in Medical Record	YES	NO	YES	HT	Supratentorial, Frontal Lobe	Central nervous system	A	NO	LIVING	2008.00
263	NO	No	C71.0	36586.00	C71.0	YES	NO	1p19q codeleted		1.00	NO	70.00	Left			NO	2.20	NO	YES	NO	NO	G2	NO		No	TCGA-HT-7607.564d0fdc-517d-4cc7-817b-	7607.00	Pre-Adjuvant Therapy	WITH TUMOR	YES	NO	Progressive Disease	NO	Primary Tumor	1.00	YES	NO	0.80	White Matter	NO	NO	YES	HT	Supratentorial, Frontal Lobe	Central nervous system	A	NO	DECEASED	2008.00
264	NO	No	C71.0	30011.00	C71.0	YES	NO	1p19q deletion		0.90	NO		Right			NO	2.20		NO	NO	YES	G2	NO		No	TCGA-HT-7608.3e9306b1-c5bc-48e4-9bc2-	7608.00		TUMOR FREE	NO	NO	Stable Disease	NO	Primary Tumor	1.00	YES	NO	0.50	White Matter	NO	NO	YES	HT	Supratentorial, Temporal Lobe	Central nervous system	A	NO	LIVING	2007.00
265	NO	No	C71.0	30011.00	C71.0	YES	NO			1.00	NO	90.00	Right			NO	3.50		YES	NO	NO	G3	NO		No	TCGA-HT-7609.d6e4b412-5a31-4cac-be39-	7609.00	Pre-Adjuvant Therapy	TUMOR FREE	NO	NO	Stable Disease	YES	Primary Tumor	1.00	NO	NO	1.00	White Matter	YES	NO	YES	HT	Supratentorial, Frontal Lobe	Central nervous system	A	NO	LIVING	2007.00
266	NO	No	C71.0	30011.00	C71.0	YES	NO			0.90	NO		Left			NO	1.50		NO	NO	NO	G2	YES		No	TCGA-HT-7610.f33a23ff-73c6-4aea-a9c0-	7610.00		TUMOR FREE	YES	NO	Stable Disease	NO	Primary Tumor	1.00	YES	NO	0.60	White Matter	NO	NO	YES	HT	Supratentorial, Temporal Lobe	Central nervous system	A	NO	LIVING	2007.00
267	NO	No	C71.0	30011.00	C71.0	YES				0.90	NO	90.00	Right				2.00	YES	NO	NO	NO	G2	NO		No	TCGA-HT-7611.3010cc40-0d85-403e-b6fa-	7611.00	Other	TUMOR FREE			Complete Remission/Response	NO	Primary Tumor	1.00	NO	NO	0.80	Not listed in Medical Record	NO	NO	YES	HT	Supratentorial, Frontal Lobe	Central nervous system	A	NO	LIVING	2007.00
268	NO	No	C71.9	18688.00	C71.9	YES	NO			0.90	NO		Right			NO	1.30		YES	NO	YES	G3	NO		No	TCGA-HT-7616.d8e58500-52b1-4fad-8a36-	7616.00		WITH TUMOR	NO	NO	Progressive Disease	NO	Primary Tumor	1.00	YES	NO	0.30	Not listed in Medical Record	NO	NO	YES	HT	Supratentorial, Frontal Lobe	Central nervous system	A	NO	DECEASED	2007.00
269	NO	No	C71.9	18688.00	C71.9	YES	NO			1.10	NO		Left			NO	3.70		NO	NO	YES	G3	YES		No	TCGA-HT-7620.4115a114-29b2-4f53-a7df-	7620.00		TUMOR FREE	YES	YES	Complete Remission/Response	YES	Primary Tumor	1.00	NO	YES	0.60	White Matter	YES	NO	YES	HT	Supratentorial, Frontal Lobe	Central nervous system	A	YES	LIVING	2007.00
270	NO	No	C71.0	18323.00	C71.0	YES				1.20	NO		Left				1.90	YES	NO		NO	G2			No	TCGA-HT-7676.275566dc-510f-46f1-8e3d-	7676.00		TUMOR FREE					Primary Tumor	1.00	YES	NO	0.40	Not listed in Medical Record		NO	YES	HT	Supratentorial, Frontal Lobe	Central nervous system	A	NO	LIVING	2009.00
271	NO	No	C71.0	18688.00	C71.0	YES	NO			1.10	NO		Left			NO	2.10		YES	NO	YES	G3	NO		No	TCGA-HT-7677.816ea52a-dca4-48f4-8fbb-	7677.00		WITH TUMOR	NO	NO	Stable Disease	YES	Primary Tumor	1.00	NO	NO	0.70	White Matter	YES	NO	YES	HT	Supratentorial, Frontal Lobe	Central nervous system	A	NO	LIVING	2009.00
272	NO	No	C71.9	36586.00	C71.9	YES	NO			1.00	NO		Midline			NO	1.60		NO	NO	YES	G2	NO		No	TCGA-HT-7680.156c9439-4f38-45cb-ab67-	7680.00		TUMOR FREE	NO	NO	Stable Disease	NO	Primary Tumor	1.00	NO	NO	0.50	White Matter	NO	NO	YES	HT	Posterior Fossa, Cerebellum	Central nervous system	A	NO	LIVING	2010.00
273	NO	No	C71.0	30011.00	C71.0	YES				0.90	NO		Right				2.80	NO				G2	NO		No	TCGA-HT-7681.d6ca1fd7-edd7-432c-a296-	7681.00		TUMOR FREE			Complete Remission/Response	NO	Primary Tumor	1.00			0.60	Not listed in Medical Record	NO	NO	YES	HT	Supratentorial, Frontal Lobe	Central nervous system	A		LIVING	2009.00
274	NO	No	C71.0	30011.00	C71.0	YES	NO			0.90	NO		Left			NO	1.70		YES	NO	NO	G3	NO		No	TCGA-HT-7684.02500cb1-2f28-4624-b001-	7684.00		TUMOR FREE	NO	NO	Complete Remission/Response	NO	Primary Tumor	1.00	NO	YES	0.90	White Matter	NO	NO	YES	HT	Supratentorial, Frontal Lobe	Central nervous system	A	NO	LIVING	2008.00
275	NO	No	C71.0	43891.00	C71.0	YES	NO			0.90	NO	90.00	Left			NO	2.00		NO	NO	NO	G3	NO		No	TCGA-HT-7686.1be5b4db-9b53-4b2f-b44b-	7686.00	Post-Adjuvant Therapy	TUMOR FREE	YES	YES	Complete Remission/Response	YES	Primary Tumor	1.00	NO	NO	0.60	Not listed in Medical Record	YES	NO	YES	HT	Supratentorial, Parietal Lobe	Central nervous system	A	YES	LIVING	2008.00
276	NO	No	C71.0	18688.00	C71.0	YES	NO			1.10	NO		Right			NO	1.90		NO		NO	G3			No	TCGA-HT-7687.f1bff6bc-cba4-4986-b4a1-	7687.00		TUMOR FREE	NO	NO	Stable Disease		Primary Tumor	1.00	NO	YES	0.40	Not listed in Medical Record		NO	YES	HT	Supratentorial, Frontal Lobe	Central nervous system	A	YES	LIVING	2010.00
277	NO	No	C71.0	18688.00	C71.0	YES	NO			0.90	NO		Left			NO	2.30		NO	NO	YES	G3	NO		No	TCGA-HT-7688.35d09648-037f-4e5e-b70b-	7688.00		TUMOR FREE	YES	YES	Complete Remission/Response	YES	Primary Tumor	1.00	YES	NO	0.60	White Matter	YES	NO	YES	HT	Supratentorial, Frontal Lobe	Central nervous system	A	YES	LIVING	2009.00
278	NO	No	C71.0	18323.00	C71.0	YES				1.00	NO	70.00	Left				1.20		YES	NO	YES	G2	NO		No	TCGA-HT-7689.21e316fb-fd2b-4890-a34a-	7689.00	Post-Adjuvant Therapy	TUMOR FREE			Complete Remission/Response	YES	Primary Tumor	1.00	NO	NO	0.70	Not listed in Medical Record	YES	NO	YES	HT	Supratentorial, Frontal Lobe	Central nervous system	A	NO	LIVING	2009.00
279	NO	No	C71.0	30011.00	C71.0	YES				0.90	NO		Right			NO	1.50		YES	NO	NO	G3	NO		No	TCGA-HT-7690.cc74dde4-33c1-4a77-a773-	7690.00		TUMOR FREE				NO	Primary Tumor	1.00	NO	NO	0.70	White Matter	NO	NO	YES	HT	Supratentorial, Frontal Lobe	Central nervous system	A	NO	LIVING	2009.00
280	NO	No	C71.0	36586.00	C71.0	YES				1.00	NO		Right			NO	1.40		NO	NO	NO	G2	NO		No	TCGA-HT-7691.f6b38096-6209-4c37-a545-	7691.00		TUMOR FREE	NO	NO		NO	Primary Tumor	1.00	NO	NO	0.80	Not listed in Medical Record	NO	NO	YES	HT	Supratentorial, Not Otherwise Specified	Central nervous system	A	YES	LIVING	2008.00
281	NO	No	C71.0	30011.00	C71.0	YES				0.80	NO	100.00	Left				1.10		YES	NO	NO	G2	NO		No	TCGA-HT-7692.c5f0a360-5011-4ede-b4b5-	7692.00	Pre-Adjuvant Therapy	TUMOR FREE	YES	YES	Complete Remission/Response	YES	Primary Tumor	1.00	YES	NO	0.50		YES	NO	YES	HT	Supratentorial, Frontal Lobe	Central nervous system	A	NO	LIVING	2009.00
282	NO	No	C71.0	18323.00	C71.0	YES				1.00	NO	90.00	Right				1.10		NO	NO	YES	G2	NO		No	TCGA-HT-7693.7ad7aac7-df39-4f13-9038-	7693.00	Post-Adjuvant Therapy	TUMOR FREE			Partial Remission/Response	YES	Primary Tumor	1.00	YES	NO	0.70		YES	NO	YES	HT	Supratentorial, Frontal Lobe	Central nervous system	A	NO	LIVING	2008.00
283	NO	No	C71.0	18688.00	C71.0	YES				0.90	NO	90.00	Right			NO	2.50		YES	NO	YES	G3	NO		No	TCGA-HT-7694.09cb8fc6-43e0-484f-9897-	7694.00	Post-Adjuvant Therapy	TUMOR FREE	NO	YES	Complete Remission/Response	YES	Primary Tumor	1.00	NO	NO	0.50	White Matter	YES	NO	YES	HT	Supratentorial, Frontal Lobe	Central nervous system	A	NO	LIVING	2008.00
284	NO	No	C71.0	18323.00	C71.0	YES				0.90	NO		Left			NO	1.80		NO	NO	NO	G2	NO		No	TCGA-HT-7695.351859ed-9826-4414-958d-	7695.00		TUMOR FREE			Complete Remission/Response	YES	Primary Tumor	1.00	YES	NO	0.70	White Matter	YES	NO	YES	HT	Supratentorial, Frontal Lobe	Central nervous system	A	NO	LIVING	2009.00
285	NO	No	C71.0	36586.00	C71.0	YES	NO			1.10	NO	100.00	Left			NO	1.50	NO	YES	NO	NO	G2	YES		No	TCGA-HT-7854.abff42e8-3d5d-438c-91a8-	7854.00	Post-Adjuvant Therapy	TUMOR FREE	YES	YES		YES	Primary Tumor	1.00	NO	NO	0.60	White Matter	NO	NO	YES	HT	Supratentorial, Temporal Lobe	Central nervous system	A	YES	LIVING	2010.00
286	NO	No	C71.0	43891.00	C71.0	YES	NO			1.50	NO	80.00	Left			NO	1.60		YES	NO	YES	G3	NO		No	TCGA-HT-7855.77a245ba-4e68-4215-86d4-	7855.00	Post-Adjuvant Therapy	TUMOR FREE	NO	YES	Complete Remission/Response	YES	Primary Tumor	1.00	NO	NO	0.60	Cerebral Cortex	YES	NO	YES	HT	Supratentorial, Parietal Lobe	Central nervous system	A	NO	LIVING	2008.00

287	NO	No	C71.0	18688.00	C71.0	YES	NO			1.00	NO	80.00	Left			NO	3.10		YES	NO	YES	G3	NO		No	TCGA-HT-7856.0c4f151b-9d73-46a3-94ee-	7856.00	Adjuvant therapy	TUMOR FREE	YES	YES	Complete Remission/Response	YES	Primary Tumor	1.00	NO	NO	0.80	White Matter	YES	NO	YES	HT	Supratentorial, Frontal Lobe	Central nervous system	A	NO	LIVING	2008.00
288	NO	No	C71.0	43891.00	C71.0	YES				1.00	NO		Left				2.30		NO	NO	YES	G3	NO		No	TCGA-HT-7857.3779c43d-34b9-426a-a59e-	7857.00		TUMOR FREE	YES	YES		NO	Primary Tumor	1.00	NO	NO	0.30	Not listed in Medical Record	NO			HT	Supratentorial, Not Otherwise Specified	Central nervous system	A	YES	LIVING	2006.00
289	NO	No	C71.0	36586.00	C71.0	YES	NO			1.80	NO	90.00	Right			NO	2.30		NO	NO	NO	G2	NO		No	TCGA-HT-7858.203f03ef-f7ed-46ce-9f5f-	7858.00	Post-Adjuvant Therapy	TUMOR FREE			Complete Remission/Response	YES	Primary Tumor	1.00	NO	NO	0.60	White Matter	NO	NO	YES	HT	Supratentorial, Frontal Lobe	Central nervous system	A	NO	LIVING	2007.00
290	NO	No	C71.0	43891.00	C71.0	YES	NO			1.10	NO		Left			NO	1.10		NO	NO	YES	G3			No	TCGA-HT-7860.281dc713-88c3-485f-96a9-	7860.00		TUMOR FREE	YES	YES			Primary Tumor	1.00	YES	NO	0.60	White Matter		NO	YES	HT	Supratentorial, Frontal Lobe	Central nervous system	A	NO	LIVING	2009.00
291	NO	No	C71.0	30011.00	C71.0	YES	NO			0.60	NO		Right			NO	1.00	NO	YES	NO	YES	G2	NO		No	TCGA-HT-7873.0efa1cbb-8b95-4b63-94bd-	7873.00		WITH TUMOR	NO	NO	Stable Disease	YES	Primary Tumor	1.00	NO	NO	0.30	White Matter	NO	NO	YES	HT	Supratentorial, Frontal Lobe	Central nervous system	B	YES	LIVING	2011.00
292	NO	No	C71.0	18688.00	C71.0	YES	NO			1.00	NO		Right			NO	2.00		YES	NO	NO	G3	NO		No	TCGA-HT-7874.dee7aaf7-0e9a-454b-a9aa-	7874.00		TUMOR FREE	YES	YES	Complete Remission/Response	YES	Primary Tumor	1.00	YES	NO	0.60	Not listed in Medical Record	YES	NO	YES	HT	Supratentorial, Frontal Lobe	Central nervous system	A	NO	LIVING	2008.00
293	NO	No	C71.0	18323.00	C71.0	YES	NO			1.10	NO	80.00	Left			NO	2.40		NO	NO	NO	G2	NO		No	TCGA-HT-7875.f247c5ba-6d4d-4d31-b3c5-	7875.00	Pre-Adjuvant Therapy	TUMOR FREE	YES	NO	Stable Disease	YES	Primary Tumor	1.00	YES	NO	0.60	White Matter	NO	NO	YES	HT	Supratentorial, Frontal Lobe	Central nervous system	A	NO	LIVING	2008.00
294	NO	No	C71.0	18323.00	C71.0	YES				0.80	NO		Left				1.20		NO	NO	YES	G2	NO		No	TCGA-HT-7877.c9bdae23-9523-46e1-8f14-	7877.00		TUMOR FREE	NO	NO		NO	Primary Tumor	1.00	NO	NO	0.60	White Matter	NO	NO	YES	HT	Supratentorial, Frontal Lobe	Central nervous system	A	NO	LIVING	2011.00
295	NO	No	C71.0	30011.00	C71.0	YES				0.80	NO		Right				1.00		NO	NO	NO	G3	NO		No	TCGA-HT-7879.632e55f9-34bc-455e-98b8-	7879.00		TUMOR FREE	YES	NO	Complete Remission/Response	YES	Primary Tumor	1.00	YES	NO	0.50		YES	NO	YES	HT	Supratentorial, Temporal Lobe	Central nervous system	A	YES	LIVING	2011.00
296	NO	No	C71.0	30011.00	C71.0	YES				0.80	NO		Right				1.10		NO	NO	NO	G2	NO		No	TCGA-HT-7880.1c00de9e-c2c1-4b57-b0a1-	7880.00		TUMOR FREE	YES	YES		NO	Primary Tumor	1.00	YES	YES	0.70	White Matter	NO	NO	YES	HT	Supratentorial, Parietal Lobe	Central nervous system	A	NO	LIVING	2011.00
297	NO	No	C71.0	18323.00	C71.0	YES				0.80	NO		Left				1.40	NO	NO	NO	NO	G2	NO		No	TCGA-HT-7881.8c9a3353-4d77-4d26-8522-	7881.00		TUMOR FREE	YES	YES	Complete Remission/Response	YES	Primary Tumor	1.00	YES	NO	0.30	Not listed in Medical Record	YES	NO	YES	HT	Supratentorial, Temporal Lobe	Central nervous system	A	NO	LIVING	2011.00
298	NO	No	C71.0	18688.00	C71.0	YES	NO			1.00	NO		Left			NO	2.40		YES	NO	YES	G3	NO		No	TCGA-HT-7882.6ef48ba9-cdeb-4279-bd24-	7882.00		WITH TUMOR	YES	NO	Progressive Disease	NO	Primary Tumor	1.00	YES	NO	0.30	Not listed in Medical Record	NO	NO	YES	HT	Supratentorial, Frontal Lobe	Central nervous system	A	NO	DECEASED	2010.00
299	NO	No	C71.0	36586.00	C71.0	YES	NO			0.80	NO	80.00	Left			NO	1.30	NO	YES	NO	NO	G2	NO		No	TCGA-HT-7884.9294057d-1aba-44d6-a756-	7884.00	Post-Adjuvant Therapy	TUMOR FREE	YES	NO	Stable Disease	YES	Primary Tumor	1.00	YES	NO	0.30	White Matter	YES	NO	YES	HT	Supratentorial, Frontal Lobe	Central nervous system	B	YES	LIVING	2011.00
300	NO	No	C71.0	30011.00	C71.0	YES	NO			0.80	NO		Right			NO	1.50		NO	NO	NO	G2	NO		No	TCGA-HT-7902.bb55771d-e5dc-4625-8c3c-	7902.00		TUMOR FREE	NO	NO	Stable Disease	NO	Primary Tumor	1.00	NO	NO	0.80	Cerebral Cortex	NO	NO	YES	HT	Supratentorial, Frontal Lobe	Central nervous system	A	YES	LIVING	2009.00
301	NO	No	C71.0	18323.00	C71.0	YES	NO			0.40	NO		Right			NO	1.30		NO	NO	YES	G2	NO		No	TCGA-HT-8010.ec4dae3c-9d46-48cb-9201-	8010.00		TUMOR FREE	NO	NO	Stable Disease	NO	Primary Tumor	1.00	NO	NO	0.30	Not listed in Medical Record	NO	NO	YES	HT	Supratentorial, Frontal Lobe	Central nervous system	A	NO	LIVING	2011.00
302	NO	No	C71.0	43891.00	C71.0	YES	NO			1.00	NO		Right			NO	1.30		NO	NO	NO	G3	YES		No	TCGA-HT-8011.67c30fd5-6ff5-49b7-b0e6-	8011.00		WITH TUMOR	YES	NO	Stable Disease	YES	Primary Tumor	1.00	YES	NO	0.60	Not listed in Medical Record	YES	NO	YES	HT	Supratentorial, Temporal Lobe	Central nervous system	A	NO	LIVING	2009.00
303	NO	No	C71.0	18323.00	C71.0	YES	NO			0.80	NO		Right			NO	1.30		NO	NO	NO	G2	YES		No	TCGA-HT-8012.d968744b-5830-4320-9143-	8012.00		WITH TUMOR	YES	NO	Stable Disease	NO	Primary Tumor	1.00	YES	NO	0.60	Not listed in Medical Record	NO	NO	YES	HT	Supratentorial, Temporal Lobe	Central nervous system	A	NO	LIVING	2010.00
304	NO	No	C71.0	30011.00	C71.0	YES	NO			0.80	NO	70.00	Left			NO	0.80		YES	NO	NO	G2	YES		No	TCGA-HT-8013.9f364b5b-ead4-42a4-a802-	8013.00	Post-Adjuvant Therapy	WITH TUMOR	YES	NO	Stable Disease	YES	Primary Tumor	1.00	YES	NO	0.80	Cerebral Cortex	YES	NO	YES	HT	Supratentorial, Frontal Lobe	Central nervous system	A	NO	DECEASED	2005.00
305	NO	No	C71.0	36586.00	C71.0	YES			660.00		NO		Left									G2		TRUE	No	TCGA-HT-8015.555DEE9A-4BA2-4E0A-A76E-	8015.00		TUMOR FREE			Complete Remission/Response		Primary Tumor	1.00				Deep Gray (e.g.basal ganglia, thalamus)		NO	YES	HT	Supratentorial, Temporal Lobe	Central nervous system	B		LIVING	2008.00
306	NO	No	C71.0	30011.00	C71.0	YES	NO			0.90	NO		Left			NO	1.30	NO	NO	NO	YES	G2	NO		No	TCGA-HT-8018.3c1686b7-7a13-43cd-9d42-	8018.00		TUMOR FREE			Partial Remission/Response	YES	Primary Tumor	1.00	YES	NO	0.90	White Matter	YES	NO	YES	HT	Supratentorial, Temporal Lobe	Central nervous system	A	NO	DECEASED	2010.00
307	NO	No	C71.0	18688.00	C71.0	YES	NO			1.00	NO		Right			NO	1.60	NO	NO	NO	NO	G3	NO		No	TCGA-HT-8019.18eba3ff-f941-4a98-8603-	8019.00		TUMOR FREE			Complete Remission/Response	YES	Primary Tumor	1.00	YES	NO	0.80	White Matter	YES	NO	YES	HT	Supratentorial, Frontal Lobe	Central nervous system	A	NO	LIVING	2009.00
308	NO	No	C71.0	43891.00	C71.0	YES	NO			0.70	NO		Left			NO	1.30	YES	NO	NO	YES	G3			No	TCGA-HT-8104.be808788-d11d-4e91-8b52-	8104.00		TUMOR FREE	NO	NO			Primary Tumor	1.00	NO	NO	0.70	White Matter		NO	YES	HT	Supratentorial, Frontal Lobe	Central nervous system	A	NO	LIVING	2010.00
309	NO	No	C71.0	18688.00	C71.0	YES	NO			1.00	NO	70.00	Right			NO	1.60		YES	NO	YES	G3	NO		No	TCGA-HT-8105.d7c51467-5baa-4858-86bd-	8105.00		TUMOR FREE	NO	NO	Stable Disease	YES	Primary Tumor	1.00	YES	NO	1.00	Not listed in Medical Record	NO	NO	YES	HT	Supratentorial, Frontal Lobe	Central nervous system	A	NO	LIVING	2011.00
310	NO	No	C71.0	43891.00	C71.0	YES	NO			0.90	NO		Midline			NO	1.20		NO	NO	NO	G3	NO		No	TCGA-HT-8106.aebc4408-92f6-404c-b9e6-	8106.00		TUMOR FREE	NO	NO	Stable Disease	NO	Primary Tumor	1.00	YES	NO	0.50	Not listed in Medical Record	NO	NO	YES	HT	Supratentorial, Frontal Lobe	Central nervous system	A	NO	LIVING	2010.00
311	NO	No	C71.0	18323.00	C71.0	YES	NO			0.60	NO		Left			NO	1.00		YES	NO	NO	G2	NO		No	TCGA-HT-8107.4e4564f4-1ebf-4d0d-a461-	8107.00		TUMOR FREE	YES	NO	Stable Disease	NO	Primary Tumor	1.00	YES	NO	0.40	Not listed in Medical Record	NO	NO	YES	HT	Supratentorial, Parietal Lobe	Central nervous system	A	NO	LIVING	2011.00
312	NO	No	C71.0	18323.00	C71.0	YES	NO			0.60	NO		Left			NO	0.70		NO	NO	NO	G2	NO		No	TCGA-HT-8108.8e2e0a3b-afd5-4077-b95f-	8108.00		TUMOR FREE	NO	NO	Stable Disease	NO	Primary Tumor	1.00	NO	NO	0.30	White Matter	NO	NO	YES	HT	Supratentorial, Parietal Lobe	Central nervous system	A	YES	LIVING	2011.00
313	NO	No	C71.0	18688.00	C71.0	YES	NO			0.80	NO		Right			NO	1.10	YES	YES	NO	NO	G3			No	TCGA-HT-8109.ae84e661-5ec1-46e2-ac49-	8109.00		TUMOR FREE	YES	YES		YES	Primary Tumor	1.00	NO	NO	0.60	White Matter	YES	NO	YES	HT	Supratentorial, Frontal Lobe	Central nervous system	A	NO	LIVING	2011.00
314	NO	No	C71.0	43891.00	C71.0	YES	NO			0.90	NO		Right			NO	1.60	NO	YES	NO	NO	G3	NO		No	TCGA-HT-8110.c423008b-608e-4260-b36c-	8110.00		TUMOR FREE			Complete Remission/Response	YES	Primary Tumor	1.00	NO	NO	0.90	White Matter		NO	YES	HT	Supratentorial, Parietal Lobe	Central nervous system	A	YES	LIVING	2011.00
315	NO	No	C71.0	30011.00	C71.0	YES				0.90	NO		Left			NO	2.20	YES	YES	NO	NO	G3			No	TCGA-HT-8111.9de92ba0-f9d6-4f7b-8a92-	8111.00			YES	NO			Primary Tumor	1.00	YES	NO	0.90	White Matter		NO	YES	HT	Supratentorial, Frontal Lobe	Central nervous system	A	NO	LIVING	2011.00
316	NO	No	C71.0	18323.00	C71.0	YES				0.90	NO	90.00	Left				2.20		NO	NO	NO	G2	NO		No	TCGA-HT-8113.93ef4c6d-e0a1-48f0-b6af-	8113.00		TUMOR FREE	YES	NO	Complete Remission/Response	YES	Primary Tumor	1.00	YES	NO	0.60	White Matter	YES	NO	YES	HT	Supratentorial, Frontal Lobe	Central nervous system	A	NO	LIVING	2006.00
317	NO	No	C71.0	30011.00	C71.0	YES				0.80	NO		Left			NO	2.00	NO	YES	NO	YES	G3	NO		No	TCGA-HT-8114.8aa9d827-a738-4750-af50-	8114.00		TUMOR FREE	NO	NO	Complete Remission/Response	YES	Primary Tumor	1.00	NO	NO	0.60	White Matter	YES	NO	YES	HT	Supratentorial, Parietal Lobe	Central nervous system	A	YES	LIVING	2011.00
318	NO	No	C71.0	18323.00	C71.0	YES				0.80	NO		Left				1.20	YES	NO	NO	NO	G2	NO		No	TCGA-HT-8558.3F364926-441B-435D-BDF5-	8558.00		TUMOR FREE	YES	YES	Complete Remission/Response	NO	Primary Tumor	1.00	YES	NO	0.50	Not listed in Medical Record	NO	NO	YES	HT	Supratentorial, Temporal Lobe	Central nervous system	A	NO	LIVING	2011.00
319	NO	No	C71.0	43891.00	C71.0	YES				0.60	NO		Right				0.70	NO	NO		NO	G3	NO		No	TCGA-HT-8563.5a5ea09f-fbc2-43a7-a135-	8563.00		TUMOR FREE			Complete Remission/Response	YES	Primary Tumor	1.00	NO	NO	0.20	Cerebral Cortex	NO	NO	YES	HT	Supratentorial, Frontal Lobe	Central nervous system	A	YES	LIVING	2011.00
320	NO	No	C71.0	43891.00	C71.0	YES				0.40	NO	80.00	Left				0.70	NO	YES	NO	NO	G3	YES		No	TCGA-HT-8564.2809249b-fed4-4b7a-b221-	8564.00	Post-Adjuvant Therapy	WITH TUMOR			Progressive Disease	NO	Primary Tumor	1.00	NO	NO	0.30	Not listed in Medical Record	YES	NO	YES	HT	Supratentorial, Temporal Lobe	Central nervous system	A	NO	LIVING	2012.00
321	NO	No	C71.0	43891.00	C71.0	YES			80.00		NO		Right			NO		YES	NO		NO	G3		TRUE	No	TCGA-HT-A4DS.3E2FDB9A-0295-493E-8F8D-	A4DS		TUMOR FREE	YES	YES			Primary Tumor	1.00	YES	YES		Not listed in Medical Record		NO	YES	HT	Supratentorial, Temporal Lobe	Central nervous system	A	NO	LIVING	2012.00
322	NO	No	C71.0	18688.00	C71.0	YES			360.00		NO	90.00	Left			NO		NO	NO		NO	G3	NO	TRUE	No	TCGA-HT-A4DV.A5D9C1B2-9F81-42BD-927B-	A4DV	Post-Adjuvant Therapy	TUMOR FREE	YES	YES	Partial Remission/Response	YES	Primary Tumor	1.00	YES	NO		White Matter	YES	NO	YES	HT	Supratentorial, Frontal Lobe	Central nervous system	A	YES	LIVING	2012.00

323	NO	No	C71.0	18323.00	C71.0	YES			260.00		NO		Left					YES	NO		YES	G2		TRUE	No	TCGA-HT-A5R5.9A308F04-2E34-40CB-97FC-	A5R5		TUMOR FREE			Complete Remission/Response		Primary Tumor	1.00	YES	NO		Not listed in Medical Record		NO	YES	HT	Supratentorial, Temporal Lobe	Central nervous system	A	NO	LIVING	2012.00
324	NO	No	C71.0	43891.00	C71.0	YES			310.00		NO	90.00	Right			YES		NO	NO		YES	G3	NO	TRUE	No	TCGA-HT-A5R7.0C8ED6F2-0B6A-4316-A53C-	A5R7	Post-Adjuvant Therapy	TUMOR FREE	YES		Complete Remission/Response	YES	Primary Tumor	1.00	YES	NO		Not listed in Medical Record	YES	NO	YES	HT	Supratentorial, Parietal Lobe	Central nervous system	A	NO	LIVING	2012.00
325	NO	No	C71.0	18688.00	C71.0	YES			390.00		NO		Right			NO		YES	NO	NO	NO	G3		TRUE	No	TCGA-HT-A5R9.D35BB9FB-030C-4B30-AE08-	A5R9							Primary Tumor	1.00	YES	NO		Not listed in Medical Record		NO	YES	HT	Supratentorial, Frontal Lobe	Central nervous system	A	NO	LIVING	2012.00
326	NO	No	C71.0	43891.00	C71.0	YES			70.00		NO	70.00	Right					NO	NO	NO	NO	G3	NO	TRUE	No	TCGA-HT-A5RA.4FF0C3AD-41E6-4814-9ED9-	A5RA	Post-Adjuvant Therapy	WITH TUMOR			Partial Remission/Response	YES	Primary Tumor	1.00	YES	NO		Not listed in Medical Record	YES	NO	YES	HT	Supratentorial, Temporal Lobe	Central nervous system	A	NO	LIVING	2012.00
327	NO	No	C71.0	36586.00	C71.0	YES			320.00		NO		Right					YES	NO	NO	NO	G2		TRUE	No	TCGA-HT-A5RB.3DDDF9B9-4EB3-4098-8898-	A5RB			YES	NO			Primary Tumor	1.00	YES	NO		Not listed in Medical Record		NO	YES	HT	Supratentorial, Frontal Lobe	Central nervous system	A	NO	LIVING	2011.00
328	NO	No	C71.0	43891.00	C71.0	YES			60.00		NO	40.00	Left						YES		YES	G3	NO	TRUE	No	TCGA-HT-A5RC.A17D7270-D512-4F3F-B40C-	A5RC	Post-Adjuvant Therapy	WITH TUMOR			Progressive Disease	YES	Primary Tumor	1.00	NO	NO		Not listed in Medical Record	YES	NO	YES	HT	Supratentorial, Frontal Lobe	Central nervous system	A	NO	DECEASED	2012.00
329	NO	No	C71.0	30011.00	C71.0	YES			130.00		NO		Right					YES	YES		YES	G2		FALSE	No	TCGA-HT-A614.9E38FBA2-A9D8-4428-B586-	A614		WITH TUMOR	YES		Partial Remission/Response		Primary Tumor	1.00	YES	YES		Not listed in Medical Record		NO	YES	HT	Supratentorial, Not Otherwise Specified	Central nervous system	A	NO	LIVING	2013.00
330	NO	No	C71.0	18323.00	C71.0	YES			140.00		NO	90.00	Left					NO	NO		NO	G2	NO	FALSE	No	TCGA-HT-A615.34BD1967-CF9D-4B21-866A-	A615	Post-Adjuvant Therapy	TUMOR FREE			Complete Remission/Response	YES	Primary Tumor	1.00	YES	NO		Not listed in Medical Record	NO	NO	YES	HT	Supratentorial, Temporal Lobe	Central nervous system	A	NO	LIVING	2012.00
331	NO	No	C71.0	36586.00	C71.0	YES			290.00		NO		Left					YES	NO	NO	NO	G2		FALSE	No	TCGA-HT-A616.65CCB3E5-56CE-4D6F-A6F5-	A616						NO	Primary Tumor	1.00	YES	NO		Not listed in Medical Record	NO	NO	YES	HT	Supratentorial, Frontal Lobe	Central nervous system	A	NO	LIVING	2013.00
332	NO	No	C71.0	18323.00	C71.0	YES			230.00		NO	60.00	Right			NO		YES	NO	NO	NO	G2	YES	FALSE	No	TCGA-HT-A617.0DF6A8BE-1AE0-40CE-98D1-	A617	Post-Adjuvant Therapy		YES	NO	Progressive Disease		Primary Tumor	1.00	YES	NO		Not listed in Medical Record		NO	YES	HT	Supratentorial, Not Otherwise Specified	Central nervous system	A	NO	LIVING	2010.00
333	NO	No	C71.0	43891.00	C71.0	YES			130.00		NO	80.00						NO	YES		YES	G3	NO	FALSE	No	TCGA-HT-A618.1E45E711-0DF2-43BD-8E84-	A618	Pre-Adjuvant Therapy	TUMOR FREE			Complete Remission/Response	NO	Primary Tumor	1.00		YES		Not listed in Medical Record	NO	NO	YES	HT	Supratentorial, Temporal Lobe	Central nervous system	A	NO	LIVING	2013.00
334	YES	Yes, Radiation Prior to Resection	C71.0	18688.00	C71.0	YES			130.00		NO		Left					NO	NO		YES	G3	NO	FALSE	Yes, History of Prior Malignancy	TCGA-HT-A619.1425BF47-215A-486A-BA84-	A619	Preoperative	TUMOR FREE			Complete Remission/Response	NO	Primary Tumor	1.00	NO	NO		Not listed in Medical Record	YES	NO	YES	HT	Supratentorial, Frontal Lobe	Central nervous system	A	NO	LIVING	2012.00
335	NO	No	C71.0	18323.00	C71.0	YES			110.00		NO	80.00	Left						NO		NO	G2		FALSE	No	TCGA-HT-A61A.52DE6BC7-B83B-4C8A-	A61A	Other	TUMOR FREE	YES		Complete Remission/Response	YES	Primary Tumor	1.00	YES	NO		Not listed in Medical Record	YES	NO	YES	HT	Supratentorial, Temporal Lobe	Central nervous system	A	NO	LIVING	2013.00
336	NO	No	C71.0	43891.00	C71.0	YES			260.00		NO	80.00	Left					NO	YES		NO	G3	NO	FALSE	No	TCGA-HT-A61B.24042BE1-BDC3-41A3-93EE-	A61B	Post-Adjuvant Therapy	TUMOR FREE	YES		Complete Remission/Response	YES	Primary Tumor	1.00	YES	NO		Not listed in Medical Record	YES	NO	YES	HT	Supratentorial, Temporal Lobe	Central nervous system	A	NO	LIVING	2012.00
337	NO	No	C71.0	18688.00	C71.0	YES			40.00		NO	100.00	Left					NO	NO	NO	YES	G3	YES	FALSE	No	TCGA-HT-A61C.D3039C78-0727-4215-8EE9-	A61C	Post-Adjuvant Therapy	WITH TUMOR	YES	YES	Complete Remission/Response	YES	Primary Tumor	1.00	YES	NO		Not listed in Medical Record	YES	NO	YES	HT	Supratentorial, Temporal Lobe	Central nervous system	A	NO	DECEASED	2013.00
338	NO	No	C71.0	43891.00	C71.0	YES			220.00		NO		Left			NO		YES	YES	NO	NO	G3		TRUE	No	TCGA-HT-A74H.436222D6-7870-4C4A-8F3A-	A74H			YES	NO	Stable Disease	YES	Primary Tumor	1.00	YES	NO		Not listed in Medical Record	YES	NO	YES	HT	Supratentorial, Temporal Lobe	Central nervous system	A	NO	LIVING	2013.00
339	NO	No	C71.0	30011.00	C71.0	YES			160.00		NO		Left	YES	IHC	YES			NO		YES	G2	NO	TRUE	No	TCGA-HT-A74J.458CC740-C271-4F99-99CE-	A74J			YES		Partial Remission/Response	NO	Primary Tumor	1.00	YES	NO		Cerebral Cortex	NO	NO	YES	HT	Supratentorial, Temporal Lobe	Central nervous system	A	NO	LIVING	2013.00
340	NO	No	C71.0	18688.00	C71.0	YES			80.00		NO		Right						NO		NO	G3	NO	TRUE	No	TCGA-HT-A74K.CB30199B-FBD0-494A-8A61-	A74K		TUMOR FREE			Complete Remission/Response	YES	Primary Tumor	1.00	YES	NO		Cerebral Cortex	YES	NO	YES	HT	Supratentorial, Frontal Lobe	Central nervous system	A	NO	LIVING	2012.00
341	NO	No	C71.0	30011.00	C71.0	YES			230.00		NO	90.00	Right	YES	IHC	YES		NO	NO	NO	YES	G2	NO	TRUE	No	TCGA-HT-A74L.FFB289B9-552E-4232-B2F7-	A74L	Pre-Adjuvant Therapy	WITH TUMOR			Partial Remission/Response		Primary Tumor	1.00	YES	NO		Cerebral Cortex		NO	YES	HT	Supratentorial, Parietal Lobe	Central nervous system	A	NO	LIVING	2013.00
342	NO	No	C71.0	43891.00	C71.0	YES			140.00		NO		Left			NO		YES	NO	NO	YES	G3		TRUE	No	TCGA-HT-A74O.0EA28E1A-D342-4149-95BB-	A74O			YES	NO			Primary Tumor	1.00	NO	YES		Not listed in Medical Record		NO	YES	HT	Supratentorial, Temporal Lobe	Central nervous system	A	YES	LIVING	2013.00
343	NO	No	C71.0	18323.00	C71.0	YES				0.90	NO		Right			NO	1.00	NO				G2	NO		No	TCGA-HW-7486.d98d840c-4acf-4312-98b5-	7486.00		TUMOR FREE		NO	Stable Disease	NO	Primary Tumor	1.00	YES		0.80	White Matter	YES			HW	Supratentorial, Frontal Lobe	Central nervous system	A		LIVING	2010.00
344	NO	No	C71.0	18323.00	C71.0	YES				0.90	NO		Left			NO	0.90	NO				G2	YES		No	TCGA-HW-7487.1aa8b50f-bce6-4ce5-9922-	7487.00		WITH TUMOR		YES	Progressive Disease	NO	Primary Tumor	1.00	YES		0.20	White Matter	YES			HW	Supratentorial, Frontal Lobe	Central nervous system	A		LIVING	2010.00
345	NO	No	C71.0	30011.00	C71.0	YES				0.80	NO		Right	YES	Sequence Analysis	YES	1.20	NO				G2	NO		No	TCGA-HW-7489.e79ae11e-5076-4e1e-a077-	7489.00		WITH TUMOR		YES	Progressive Disease	NO	Primary Tumor	1.00	YES		0.30	White Matter	NO			HW	Supratentorial, Frontal Lobe	Central nervous system	A		DECEASED	2011.00
346	NO	No	C71.0	36586.00	C71.0	YES				1.10	NO		Left	YES		YES	1.30	NO				G2	NO		Yes	TCGA-HW-7490.d3358d25-968e-4814-8b2f-	7490.00		WITH TUMOR		NO	Progressive Disease	NO	Primary Tumor	1.00	NO		0.70	White Matter	NO			HW	Supratentorial, Occipital Lobe	Central nervous system	A		LIVING	2011.00
347	NO	No	C71.0	18323.00	C71.0	YES				0.70	NO		Right			NO	1.10	NO				G2	YES		No	TCGA-HW-7491.8e93a347-6300-4a66-b5f2-	7491.00		WITH TUMOR		NO	Progressive Disease	NO	Primary Tumor	1.00	NO		0.50	White Matter	YES			HW	Supratentorial, Frontal Lobe	Central nervous system	A		LIVING	2009.00
348	NO	No	C71.0	36586.00	C71.0	YES				0.80	NO		Right			NO	1.10	NO				G2	NO		No	TCGA-HW-7493.19b2a34c-33d1-49a2-ac5b-	7493.00		WITH TUMOR		YES	Stable Disease	YES	Primary Tumor	1.00	YES		0.70	White Matter	NO			HW	Supratentorial, Temporal Lobe	Central nervous system	A		LIVING	2009.00
349	NO	No	C71.0	18323.00	C71.0	YES				1.10	NO		Right			NO	1.70	NO				G2	NO		No	TCGA-HW-7495.c5ad0f30-8b83-4d3b-b695-	7495.00		WITH TUMOR		NO	Progressive Disease	NO	Primary Tumor	1.00	YES		0.90	White Matter	NO			HW	Supratentorial, Frontal Lobe	Central nervous system	A		LIVING	2011.00
350	NO	No	C71.0	43891.00	C71.0	YES				1.20	NO		Left				1.30	NO				G3	YES		No	TCGA-HW-8319.c90e82a8-f877-40d8-b75f-	8319.00		WITH TUMOR		NO	Progressive Disease	YES	Primary Tumor	1.00	NO		0.60		YES	YES	NO	HW	Supratentorial, Frontal Lobe	Central nervous system	A		DECEASED	2011.00
351	NO	No	C71.0	43891.00	C71.0	YES				1.00	NO		Right		Sequence Analysis	YES	1.70	NO				G3	NO		No	TCGA-HW-8320.75564dac-fb13-4214-b588-	8320.00		WITH TUMOR		YES	Stable Disease	YES	Primary Tumor	1.00	YES		1.00		YES	YES	NO	HW	Supratentorial, Parietal Lobe	Central nervous system	A		LIVING	2011.00
352	NO	No	C71.0	36586.00	C71.0	YES				1.00	NO		Left				1.60	NO				G3	NO		No	TCGA-HW-8321.985f7f28-3f83-4cda-bfc8-	8321.00		WITH TUMOR		YES	Stable Disease	YES	Primary Tumor	1.00	NO		0.50		YES	YES	NO	HW	Supratentorial, Frontal Lobe	Central nervous system	A		LIVING	2011.00
353	NO	No	C71.0	18323.00	C71.0	YES				0.90	NO		Left				1.50	NO				G2	NO		No	TCGA-HW-8322.43aea5c8-29ec-4f1e-b948-	8322.00		WITH TUMOR		NO	Stable Disease	NO	Primary Tumor	1.00	YES		0.50		YES	YES	NO	HW	Supratentorial, Frontal Lobe	Central nervous system	A		LIVING	2012.00
354	NO	No	C71.0	18688.00	C71.0	YES			640.00		NO		Right					NO				G3	NO	TRUE	No	TCGA-HW-A5KJ.5A607989-F1E0-45AD-A669-	A5KJ		WITH TUMOR		NO	Progressive Disease	YES	Primary Tumor	1.00	NO			White Matter	YES	YES	NO	HW	Supratentorial, Frontal Lobe	Central nervous system	A		DECEASED	2012.00
355	NO	No	C71.0	43891.00	C71.0	YES			360.00		NO		Right					NO				G3	NO	TRUE	No	TCGA-HW-A5KK.3F8E4F24-CEB2-4B96-B251-	A5KK		WITH TUMOR		YES	Progressive Disease	YES	Primary Tumor	1.00	YES			White Matter	YES	YES	NO	HW	Supratentorial, Temporal Lobe	Central nervous system	A		DECEASED	2012.00
356	NO	No	C71.0	36586.00	C71.0	YES							Right					NO				G2	NO		No		A5KL		WITH TUMOR		NO	Stable Disease	YES	Primary Tumor	1.00	YES			White Matter	YES	YES	NO	HW	Supratentorial, Temporal Lobe	Central nervous system			LIVING	2012.00
357	NO	No	C71.0	36586.00	C71.0	YES			160.00		NO		Left					NO				G2	NO	TRUE	No	TCGA-HW-A5KM.1DA8A802-EC7A-4452-91AD-	A5KM		WITH TUMOR		NO	Progressive Disease	NO	Primary Tumor	1.00	YES			White Matter	NO	YES	NO	HW	Supratentorial, Frontal Lobe	Central nervous system	A		LIVING	2012.00
358	NO	No	C71.0	18323.00	C71.0	YES				0.80	NO	90.00	Right				1.00		NO		YES	G2	YES		No	TCGA-IK-7675.bb5fc5df-bc8d-4603-a924-	7675.00	Other	WITH TUMOR	YES	YES	Progressive Disease	YES	Primary Tumor	1.00	NO	NO	0.40	White Matter	NO	YES	NO	IK	Supratentorial, Frontal Lobe	Central nervous system	A	NO	DECEASED	2009.00

359	NO	No	C71.0	30011.00	C71.0	YES				0.80	NO		Left				0.90	NO	NO		NO	G3	NO		Yes	TCGA-IK-8125.77a2c310-8f8b-4e45-92d8-	8125.00	Pre-Adjuvant Therapy	TUMOR FREE	YES	YES	Complete Remission/Response	YES	Primary Tumor	1.00	YES	NO	0.60		YES	YES	NO	IK	Supratentorial, Frontal Lobe	Central nervous system	A	NO	LIVING	2009.00
360	NO	No	C71.0	30011.00	C71.0	YES			210.00		NO	90.00	Left	YES	IHC	YES		NO	YES	NO	YES	G3	NO	TRUE	No	TCGA-KT-A74X.70559888-99AB-44B6-8553-	A74X		WITH TUMOR			Stable Disease	YES	Primary Tumor	1.00	YES			Not listed in Medical Record	YES	YES	NO	KT	Supratentorial, Parietal Lobe	Central nervous system	A	NO	LIVING	2013.00
361	NO	No	C71.0	43891.00	C71.0	YES	NO					80.00	Right	NO	IHC	YES		NO	NO		YES	G3	NO		No		A7W1	Pre-Adjuvant Therapy	TUMOR FREE	YES	YES	Progressive Disease	YES	Primary Tumor	1.00	YES	NO		White Matter	YES	YES	NO	KT	Supratentorial, Frontal Lobe	Central nervous system		NO	LIVING	2013.00
362	NO	No	C71.0	36586.00	C71.0	YES			130.00		NO	60.00	Left					YES	YES		YES	G2		TRUE	No	TCGA-P5-A5EV.FDEF19AA-E140-405F-8BF4-	A5EV		WITH TUMOR	YES	YES			Primary Tumor	1.00	YES	YES		Not listed in Medical Record	NO	YES	NO	P5	Supratentorial, Temporal Lobe	Central nervous system	A	YES	LIVING	2012.00
363	NO	No	C71.0	36586.00	C71.0	YES			240.00		NO	80.00	Right			NO		YES		NO		G2	NO	TRUE	No	TCGA-P5-A5EW.604A50C0-CFE0-4D48-87ED-	A5EW	Preoperative	TUMOR FREE	YES	NO			Primary Tumor	1.00				Not listed in Medical Record		YES	NO	P5	Supratentorial, Frontal Lobe	Central nervous system	A		LIVING	2012.00
364	NO	No	C71.0	18688.00	C71.0	YES	NO		280.00		NO	80.00	Right			NO		NO	NO	NO	NO	G3	NO	TRUE	No	TCGA-P5-A5EX.C2C64F70-4E69-4A52-97AE-	A5EX		TUMOR FREE	NO	NO	Partial Remission/Response	YES	Primary Tumor	1.00	NO	YES		Not listed in Medical Record	YES	YES	NO	P5	Supratentorial, Frontal Lobe	Central nervous system	A	NO	LIVING	2012.00
365		No	C71.0	36586.00	C71.0	YES			550.00		NO		Right					YES				G2		TRUE	No	TCGA-P5-A5EY.13BDC5C7-92EB-4FCD-	A5EY			YES				Primary Tumor	1.00	YES			Not listed in Medical Record		YES	NO	P5	Supratentorial, Temporal Lobe	Central nervous system	A		LIVING	2012.00
366	NO	No	C71.0	43891.00	C71.0	YES			200.00		NO		Left			NO		YES				G3	NO	TRUE	No	TCGA-P5-A5EZ.F12771D2-103F-423A-A619-	A5EZ		TUMOR FREE					Primary Tumor	1.00	YES			Not listed in Medical Record		YES	NO	P5	Supratentorial, Frontal Lobe	Central nervous system	A		LIVING	2012.00
367	NO	No	C71.9	18323.00	C71.9	YES	NO		110.00		NO	80.00	Right			NO		NO	NO	NO	YES	G2	NO	TRUE	No	TCGA-P5-A5F0.6E1C12F5-85C4-4A43-8F05-	A5F0		TUMOR FREE	NO	NO	Partial Remission/Response	YES	Primary Tumor	1.00	YES	YES		Not listed in Medical Record	YES	YES	NO	P5	Supratentorial, Frontal Lobe	Central nervous system	A	YES	LIVING	2012.00
368	NO	No	C71.0	36586.00	C71.0	YES			500.00		NO		Left			NO		YES			YES	G2	NO	TRUE	No	TCGA-P5-A5F1.FA13A2CB-015F-4302-88ED-	A5F1		TUMOR FREE					Primary Tumor	1.00				Not listed in Medical Record		YES	NO	P5	Supratentorial, Temporal Lobe	Central nervous system	A		LIVING	2012.00
369	NO	No	C71.0	36586.00	C71.0	YES			610.00		NO	80.00	Right					NO	NO		NO	G2	NO	TRUE	No	TCGA-P5-A5F2.0DC839C0-415F-48FB-92A8-	A5F2		WITH TUMOR			Partial Remission/Response	NO	Primary Tumor	1.00	YES	NO		Not listed in Medical Record	YES	YES	NO	P5	Supratentorial, Frontal Lobe	Central nervous system	A	NO	LIVING	2012.00
370	NO	No	C71.0	18688.00	C71.0	YES			700.00		NO	70.00	Right					NO				G3		TRUE	No	TCGA-P5-A5F4.52CF242F-7062-4258-A991-	A5F4		TUMOR FREE			Partial Remission/Response	YES	Primary Tumor	1.00		YES		Not listed in Medical Record	YES	YES	NO	P5	Supratentorial, Temporal Lobe	Central nervous system	A	YES	LIVING	2012.00
371	NO	No	C71.0	18323.00	C71.0	YES			140.00		NO	70.00	Left					YES				G2	NO	TRUE	No	TCGA-P5-A5F6.1CAB6A23-C768-4790-9597-	A5F6		TUMOR FREE					Primary Tumor	1.00				Not listed in Medical Record		YES	NO	P5	Supratentorial, Frontal Lobe	Central nervous system	A		LIVING	2012.00
372		No	C71.0	18688.00	C71.0	YES			240.00		NO		Right					NO				G3	NO	TRUE	No	TCGA-P5-A72U.5C661E0D-6AC0-47DD-851F-	A72U		TUMOR FREE			Complete Remission/Response	NO	Primary Tumor	1.00					NO	YES	NO	P5	Supratentorial, Parietal Lobe	Central nervous system	A		LIVING	2013.00
373		No	C71.0	43891.00	C71.0	YES			500.00		NO		Left					NO				G3	NO	TRUE	No	TCGA-P5-A72W.5BBDAFDC-37F6-4750-9F67-	A72W		TUMOR FREE			Complete Remission/Response	YES	Primary Tumor	1.00						YES	NO	P5	Supratentorial, Frontal Lobe	Central nervous system	A		LIVING	2013.00
374		No	C71.0	43891.00	C71.0	YES			1150.00		NO		Left					NO				G3		TRUE	No	TCGA-P5-A72X.6553F280-EF4A-4FAA-9FA8-	A72X		TUMOR FREE			Complete Remission/Response	YES	Primary Tumor	1.00						YES	NO	P5	Supratentorial, Temporal Lobe	Central nervous system	A		LIVING	2013.00
375		No	C71.0	18688.00	C71.0	YES			160.00		NO		Left					NO				G3		TRUE	No	TCGA-P5-A72Z.99B1BF7C-E68F-4546-B13B-	A72Z		TUMOR FREE			Complete Remission/Response	YES	Primary Tumor	1.00					YES	YES	NO	P5	Supratentorial, Temporal Lobe	Central nervous system	A		LIVING	2013.00
376		No	C71.0	30011.00	C71.0	YES			510.00		NO		Right					NO				G3	NO	TRUE	No	TCGA-P5-A730.D03B2072-0B67-431A-9C5E-	A730		TUMOR FREE			Complete Remission/Response	NO	Primary Tumor	1.00					NO	YES	NO	P5	Supratentorial, Occipital Lobe	Central nervous system	A		LIVING	2013.00
377		No	C71.0	30011.00	C71.0	YES			220.00		NO	90.00	Right					NO	YES			G2		TRUE	No	TCGA-P5-A731.F22D0DFD-A361-4735-B391-	A731		TUMOR FREE				YES	Primary Tumor	1.00					NO	YES	NO	P5	Supratentorial, Frontal Lobe	Central nervous system	A		LIVING	2013.00
378		No	C71.0	36586.00	C71.0	YES			300.00		NO		Left					NO				G2		TRUE	No	TCGA-P5-A733.4F732BEF-ADE0-4381-8D60-	A733		TUMOR FREE			Complete Remission/Response	YES	Primary Tumor	1.00					YES	YES	NO	P5	Supratentorial, Frontal Lobe	Central nervous system	A		LIVING	2013.00
379		No	C71.0	36586.00	C71.0	YES			510.00		NO		Left					NO				G2		TRUE	No	TCGA-P5-A735.7950CF04-F8C0-4855-A108-	A735		TUMOR FREE			Complete Remission/Response	NO	Primary Tumor	1.00					NO	YES	NO	P5	Supratentorial, Temporal Lobe	Central nervous system	A		LIVING	2013.00
380		No	C71.0	43891.00	C71.0	YES			1110.00		NO		Right					NO				G3		TRUE	No	TCGA-P5-A736.46EF7549-943C-45E2-98AA-	A736		TUMOR FREE			Complete Remission/Response	YES	Primary Tumor	1.00					NO	YES	NO	P5	Supratentorial, Parietal Lobe	Central nervous system	A		LIVING	2013.00
381		No	C71.0	36586.00	C71.0	YES			310.00		NO		Right					NO				G2		TRUE	No	TCGA-P5-A737.3DD50AE3-0357-4D02-9AAD-	A737		TUMOR FREE			Complete Remission/Response	NO	Primary Tumor	1.00					NO	YES	NO	P5	Supratentorial, Frontal Lobe	Central nervous system	A		LIVING	2012.00
382		No	C71.0	30011.00	C71.0	YES			300.00		NO		Left					NO				G3		TRUE	No	TCGA-P5-A77W.E3E86605-648A-4153-A364-	A77W		TUMOR FREE			Complete Remission/Response	NO	Primary Tumor	1.00					NO	YES	NO	P5	Supratentorial, Frontal Lobe	Central nervous system	A		LIVING	2013.00
383		No	C71.0	30011.00	C71.0	YES			1480.00		NO		Right					NO				G2		TRUE	No	TCGA-P5-A77X.E1A92E19-6BC7-4AA0-	A77X		TUMOR FREE			Complete Remission/Response	YES	Primary Tumor	1.00					NO	YES	NO	P5	Supratentorial, Frontal Lobe	Central nervous system	A		LIVING	2013.00
384		No	C71.0	43891.00	C71.0	YES			230.00		NO		Right					NO				G3		TRUE	No	TCGA-P5-A780.6EBF635A-6875-4843-815A-	A780		TUMOR FREE			Complete Remission/Response	YES	Primary Tumor	1.00						YES	NO	P5	Supratentorial, Temporal Lobe	Central nervous system	A		LIVING	2013.00
385		No	C71.0	43891.00	C71.0	YES			2980.00		NO		Right					NO				G3		TRUE	No	TCGA-P5-A781.32064991-35D2-489F-836E-	A781		TUMOR FREE			Complete Remission/Response	YES	Primary Tumor	1.00					NO	YES	NO	P5	Supratentorial, Temporal Lobe	Central nervous system	A		LIVING	2013.00
386	NO	No	C71.9	18688.00	C71.9	YES	NO					100.00	Left	YES	IHC	YES		NO	NO	YES	NO	G3	NO		No		A65R	Preoperative	WITH TUMOR	NO	NO	Partial Remission/Response	YES	Primary Tumor	1.00	NO	NO		White Matter	YES	NO	YES	QH	Supratentorial, Frontal Lobe	Central nervous system		NO	LIVING	2013.00
387	NO	No	C71.9	30011.00	C71.9	YES	NO		100.00		NO	90.00	Left	YES	IHC	YES		NO	YES	NO	NO	G2	NO	FALSE	No	TCGA-QH-A65S.D970F3D5-D401-4BC0-A2FF-	A65S	Preoperative	WITH TUMOR	YES	YES	Partial Remission/Response	NO	Primary Tumor	1.00	YES	NO		White Matter	NO	NO	YES	QH	Supratentorial, Frontal Lobe	Central nervous system	A	NO	LIVING	2013.00
388	NO	No	C71.0	18323.00	C71.0	YES	NO		380.00		NO	90.00	Right	YES	IHC	YES		NO	NO	NO	NO	G2	NO	FALSE	No	TCGA-QH-A65V.32E7C9B3-CC0B-4D62-9798-	A65V	Preoperative	WITH TUMOR	YES	YES	Stable Disease	NO	Primary Tumor	1.00	YES	NO		White Matter	YES	YES	NO	QH	Supratentorial, Temporal Lobe	Central nervous system	A	NO	LIVING	2013.00
389	NO	No	C71.0	30011.00	C71.0	YES	NO		230.00		NO	90.00	Right	NO	Sequence Analysis	YES		NO	NO	NO	NO	G3	NO	FALSE	No	TCGA-QH-A65X.D7B970B7-6910-4926-AD60-	A65X	Post-Adjuvant Therapy	TUMOR FREE	YES	NO	Stable Disease	YES	Primary Tumor	1.00	YES	NO		White Matter	YES	YES	NO	QH	Supratentorial, Frontal Lobe	Central nervous system	A	NO	LIVING	2013.00
390	NO	No	C71.0	18323.00	C71.0	YES	NO		210.00		NO	90.00	Right	YES	IHC	YES		NO	NO	NO	YES	G2	NO	FALSE	No	TCGA-QH-A65Z.BA60EA12-509F-4699-AADC-	A65Z	Preoperative	TUMOR FREE	YES	YES	Stable Disease	YES	Primary Tumor	1.00	YES	YES		White Matter	YES	YES	NO	QH	Supratentorial, Frontal Lobe	Central nervous system	A	NO	LIVING	2013.00
391	NO	No	C71.0	43891.00	C71.0	YES	NO		490.00		NO	90.00	Right	NO	IHC	YES		NO	NO	NO	YES	G3	YES	FALSE	No	TCGA-QH-A6CS.0C4238FC-0A3C-46B5-8FFF-	A6CS	Preoperative	WITH TUMOR	YES	YES	Stable Disease	YES	Primary Tumor	1.00	YES	NO		White Matter	YES	YES	NO	QH	Supratentorial, Frontal Lobe	Central nervous system	A	NO	LIVING	2013.00
392	NO	No	C71.0	18688.00	C71.0	YES	NO		470.00		NO	90.00	Left	YES	IHC	YES		NO	YES	YES	NO	G3	NO	FALSE	No	TCGA-QH-A6CU.F87EBF56-A3DC-4790-98AB-	A6CU	Preoperative	TUMOR FREE	YES	YES	Stable Disease	YES	Primary Tumor	1.00	YES	NO		White Matter	YES	YES	NO	QH	Supratentorial, Frontal Lobe	Central nervous system	A	NO	LIVING	2013.00
393	NO	No	C71.0	30011.00	C71.0	YES			190.00		NO	90.00	Right	YES	Sequence Analysis	YES		NO	NO	NO	NO	G3	NO	FALSE	No	TCGA-QH-A6CV.DC5685CF-74DF-45C6-B530-	A6CV	Preoperative	WITH TUMOR	YES	NO	Stable Disease	YES	Primary Tumor	1.00	YES	NO		White Matter	NO	YES	NO	QH	Supratentorial, Frontal Lobe	Central nervous system	A	NO	LIVING	2013.00
394	NO	No	C71.0	30011.00	C71.0	YES			270.00		NO	90.00	Right	YES	IHC	YES		NO	NO	YES	NO	G3	NO	FALSE	No	TCGA-QH-A6CW.0D917D08-E23D-4158-8049-	A6CW	Preoperative	TUMOR FREE	YES	NO	Partial Remission/Response	NO	Primary Tumor	1.00	YES	NO		White Matter	NO	YES	NO	QH	Supratentorial, Temporal Lobe	Central nervous system	A	NO	LIVING	2013.00

395	NO	No	C71.0	36586.00	C71.0	YES	NO		270.00		NO	90.00	Left	NO	IHC	YES		NO	NO	NO	NO	G2	YES	FALSE	No	TCGA-QH-A6CX.FD9CC0AD-0EDC-4B6F-	A6CX	Preoperative	WITH TUMOR	YES	YES	Progressive Disease	YES	Primary Tumor	1.00	YES	NO		White Matter	NO	YES	NO	QH	Supratentorial, Temporal Lobe	Central nervous system	A	NO	DECEASED	2013.00
396	NO	No	C71.0	30011.00	C71.0	YES			360.00		NO	90.00	Right	YES	IHC	YES		NO	NO	NO	NO	G3	NO	FALSE	No	TCGA-QH-A6CY.F47D9AFD-E511-450E-9F15-	A6CY	Preoperative	WITH TUMOR	YES	YES	Stable Disease	YES	Primary Tumor	1.00	YES	NO		White Matter	YES	YES	NO	QH	Supratentorial, Frontal Lobe	Central nervous system	A	NO	LIVING	2013.00
397	NO	No	C71.0	30011.00	C71.0	YES			360.00		NO	90.00	Right	YES	IHC	YES		NO	NO	NO	NO	G2	NO	FALSE	No	TCGA-QH-A6CZ.B346A105-9790-4B51-B83E-	A6CZ	Preoperative	TUMOR FREE	YES	YES	Stable Disease	NO	Primary Tumor	1.00	YES	NO		White Matter	NO	YES	NO	QH	Supratentorial, Frontal Lobe	Central nervous system	A	NO	LIVING	2013.00
398	NO	No	C71.0	30011.00	C71.0	YES	NO					90.00	Right	YES	IHC	YES		NO	NO	NO	NO	G2	NO		No		A6X3	Other	TUMOR FREE	NO	NO	Complete Remission/Response	NO	Primary Tumor	1.00	YES	NO		White Matter	NO	YES	NO	QH	Supratentorial, Parietal Lobe	Central nervous system		NO	LIVING	2013.00
399	NO	No	C71.0	30011.00	C71.0	YES	NO		550.00		NO	90.00	Left	YES	Sequence Analysis	YES		NO	NO	NO	YES	G3	NO	FALSE	No	TCGA-QH-A6X4.638A5F85-FA2A-4E1D-B1BC-	A6X4	Pre-Adjuvant Therapy	TUMOR FREE	YES	YES	Complete Remission/Response	YES	Primary Tumor	1.00	YES	YES		Cerebral Cortex	YES	YES	NO	QH	Supratentorial, Temporal Lobe	Central nervous system	A	NO	LIVING	2013.00
400	NO	No	C71.0	30011.00	C71.0	YES	NO		320.00		NO	90.00	Left	YES	Sequence Analysis	YES		NO	NO	NO	YES	G2	NO	FALSE	No	TCGA-QH-A6X5.5125411F-F380-4367-8A21-	A6X5	Other		NO	NO		NO	Primary Tumor	1.00	YES	NO		White Matter	NO	YES	NO	QH	Supratentorial, Frontal Lobe	Central nervous system	A	NO	LIVING	2013.00
401	NO	No	C71.0	18688.00	C71.0	YES	NO		330.00		NO	100.00	Left	YES	IHC	YES		NO	YES	NO	YES	G3	NO	FALSE	No	TCGA-QH-A6X8.01982953-8F6F-4BDE-B645-	A6X8	Post-Adjuvant Therapy	WITH TUMOR	YES	NO	Stable Disease	YES	Primary Tumor	1.00	YES	NO		White Matter	YES	YES	NO	QH	Supratentorial, Frontal Lobe	Central nervous system	A	NO	LIVING	2013.00
402	NO	No	C71.0	18323.00	C71.0	YES	NO		190.00		NO	40.00	Right	YES	Sequence Analysis	YES		NO	YES	NO	NO	G2	NO	FALSE	No	TCGA-QH-A6X9.62469927-D0A5-4C27-9ABA-	A6X9	Pre-Adjuvant Therapy		YES	NO		NO	Primary Tumor	1.00	YES	NO		White Matter	NO	YES	NO	QH	Supratentorial, Temporal Lobe	Central nervous system	A	NO	LIVING	2013.00
403	NO	No	C71.0	30011.00	C71.0	YES	NO		230.00		NO	100.00	Left	YES	Sequence Analysis	YES		NO	NO	YES	NO	G2	NO	FALSE	No	TCGA-QH-A6XA.9B2B37D4-EF00-4EC5-A549-	A6XA	Pre-Adjuvant Therapy	TUMOR FREE	NO	NO	Complete Remission/Response	NO	Primary Tumor	1.00	NO	NO		White Matter	NO	YES	NO	QH	Supratentorial, Frontal Lobe	Central nervous system	A	NO	LIVING	2013.00
404	NO	No	C71.0	43891.00	C71.0	YES	NO		310.00		NO	70.00	Right	NO	Sequence Analysis	YES		NO	NO		NO	G3	YES	FALSE	No	TCGA-QH-A6XC.27080340-615E-489D-932B-	A6XC	Post-Adjuvant Therapy	WITH TUMOR	YES	NO	[Discrepancy]	YES	Primary Tumor	1.00	YES	NO		White Matter	YES	YES	NO	QH	Supratentorial, Frontal Lobe	Central nervous system	A	NO	LIVING	2013.00
405	NO	No	C71.0	18323.00	C71.0	YES	NO		240.00		NO	100.00	Left	YES	IHC	YES		NO	NO	NO	NO	G2	NO	FALSE	No	TCGA-QH-A86X.CC0201F5-5747-4A46-A364-	A86X	Other	TUMOR FREE	YES	NO	Complete Remission/Response	NO	Primary Tumor	1.00	YES	NO		Cerebral Cortex	NO	YES	NO	QH	Supratentorial, Frontal Lobe	Central nervous system	A	NO	LIVING	2013.00
406	NO	No	C71.0	30011.00	C71.0	YES	NO		450.00		NO	80.00	Right	YES	IHC	YES		NO	NO	NO	NO	G3	YES	FALSE	No	TCGA-QH-A870.C84F6D0C-3879-4D63-8CE8-	A870	Other	WITH TUMOR	YES	YES	Complete Remission/Response	NO	Primary Tumor	1.00	NO	NO		White Matter	NO	YES	NO	QH	Supratentorial, Occipital Lobe	Central nervous system	A	YES	LIVING	2013.00
407	NO	No	C71.0	18323.00	C71.0	YES			260.00		NO		Right									G2		TRUE	No	TCGA-R8-A6MK.0E083D5C-1EE1-4BDA-9D6E-	A6MK						NO	Primary Tumor	1.00	YES				YES	NO	YES	R8	Supratentorial, Frontal Lobe	Central nervous system	A		LIVING	2005.00
408		Yes, Pharmaceutical Treatment Prior to	C71.0	18688.00	C71.0	YES			130.00		NO		Left									G3		TRUE	No	TCGA-R8-A6ML.E87BD3AD-C060-4DF5-8396-	A6ML						YES	Primary Tumor	1.00	YES				YES	NO	YES	R8	Supratentorial, Frontal Lobe	Central nervous system	A		LIVING	2005.00
409		No	C71.0	18323.00	C71.0	YES			380.00		NO		Right									G2		TRUE	No	TCGA-R8-A6MO.333C073F-08C4-475A-B622-	A6MO						NO	Primary Tumor	1.00	YES				NO	NO	YES	R8	Supratentorial, Frontal Lobe	Central nervous system	A		LIVING	2009.00
410		No	C71.0	18323.00	C71.0	YES							Left									G2	NO		Yes, History of Prior Malignancy		A73M						NO	Primary Tumor	1.00					YES	NO	YES	R8	Supratentorial, Parietal Lobe	Central nervous system		YES	LIVING	2008.00
411	NO	No	C71.0	18323.00	C71.0	YES	NO		130.00		NO		Right	YES	IHC	YES		NO	YES	NO	NO	G2	NO	FALSE	No	TCGA-RY-A83X.0964A173-152D-4C42-BBBC-	A83X		TUMOR FREE				NO	Primary Tumor	1.00	NO	NO		Cerebral Cortex	NO	YES	NO	RY	Supratentorial, Frontal Lobe	Central nervous system	A	NO	LIVING	2012.00
412	NO	No	C71.0	18323.00	C71.0	YES	NO		150.00		NO	80.00	Left	YES	IHC	YES		YES	YES	NO	NO	G2		FALSE	No	TCGA-RY-A83Y.EEC58176-5007-4A8F-9BBB-	A83Y	Other	TUMOR FREE				NO	Primary Tumor	1.00	YES	NO		Cerebral Cortex		YES	NO	RY	Supratentorial, Parietal Lobe	Central nervous system	A	NO	LIVING	2012.00
413	NO	No	C71.9	43891.00	C71.9	YES	NO		160.00		NO		Left	YES	IHC	NO		YES	NO	NO	NO	G3	NO	FALSE	No	TCGA-RY-A83Z.923A22A4-A05E-4B5E-B682-	A83Z		WITH TUMOR			Stable Disease	YES	Primary Tumor	1.00	YES	NO		Cerebral Cortex	YES	YES	NO	RY	Supratentorial, Frontal Lobe	Central nervous system	A	NO	LIVING	2012.00
414	NO	No	C71.0	18688.00	C71.0	YES	NO		60.00		NO	90.00	Left	YES	IHC	YES		NO	NO	NO	NO	G3	NO	FALSE	No	TCGA-RY-A840.D0ABD0C6-7F14-4CC3-9B44-	A840	Pre-Adjuvant Therapy	WITH TUMOR			Stable Disease	NO	Primary Tumor	1.00	YES	NO		Cerebral Cortex	YES	YES	NO	RY	Supratentorial, Temporal Lobe	Central nervous system	A	NO	LIVING	2012.00
415	NO	No	C71.0	43891.00	C71.0	YES	NO		300.00		NO		Left	NO	IHC	YES		YES	NO	NO	NO	G3	NO	FALSE	No	TCGA-RY-A843.2C380241-843C-4301-8891-	A843		WITH TUMOR				NO	Primary Tumor	1.00	YES	NO		Cerebral Cortex	YES	YES	NO	RY	Supratentorial, Temporal Lobe	Central nervous system	A	NO	LIVING	2012.00
416	NO	No	C71.0	30011.00	C71.0	YES	NO		250.00		NO	80.00	Left	NO	IHC	YES		NO	YES	NO	NO	G2	NO	FALSE	No	TCGA-RY-A845.E4FBBEC7-8717-4D7D-B390-	A845		TUMOR FREE				NO	Primary Tumor	1.00	YES	NO		Cerebral Cortex	NO	YES	NO	RY	Supratentorial, Parietal Lobe	Central nervous system	A	NO	LIVING	2013.00
417	NO	No	C71.0	18323.00	C71.0	YES	NO		60.00		NO	90.00	Right	YES	IHC	YES		NO	NO		NO	G2	NO	FALSE	No	TCGA-RY-A847.E1F9B39F-DE08-45CD-	A847	Other	TUMOR FREE				NO	Primary Tumor	1.00	NO	NO		Cerebral Cortex	NO	YES	NO	RY	Supratentorial, Frontal Lobe	Central nervous system	A	NO	LIVING	2012.00
418	NO	No	C71.0	43891.00	C71.0	YES	NO		150.00		NO	100.00	Right			NO		NO	NO	NO	NO	G3	YES	FALSE	No	TCGA-S9-A6TS.A6DF65CD-E78C-4CF8-ACC7-	A6TS	Preoperative	WITH TUMOR	NO	NO	Progressive Disease	YES	Primary Tumor	1.00	NO	NO		Cerebral Cortex	NO	NO	YES	S9	Supratentorial, Frontal Lobe	Central nervous system	A	NO	DECEASED	2006.00
419	NO	No	C71.0	36586.00	C71.0	YES			170.00		NO	100.00	Left	YES	IHC	YES		NO	NO	NO	NO	G2	YES	FALSE	No	TCGA-S9-A6TU.8CD1578E-88C2-4328-97B8-	A6TU	Preoperative	WITH TUMOR	NO	NO	Partial Remission/Response	NO	Primary Tumor	1.00	YES	YES		Cerebral Cortex	NO	NO	YES	S9	Supratentorial, Temporal Lobe	Central nervous system	A	NO	LIVING	2007.00
420	NO	No	C71.0	30011.00	C71.0	YES			140.00		NO	90.00	Right	YES	IHC	YES		YES	NO	NO	YES	G3	NO	FALSE	No	TCGA-S9-A6TV.0D9D6080-A0B2-4198-ACDF-	A6TV	Preoperative	TUMOR FREE	NO	YES	Complete Remission/Response	YES	Primary Tumor	1.00	NO	NO		Cerebral Cortex	YES	NO	YES	S9	Supratentorial, Frontal Lobe	Central nervous system	A	YES	LIVING	2010.00
421	NO	No	C71.0	18688.00	C71.0	YES						90.00	Left	YES	IHC	YES		NO	NO	NO	YES	G3	NO		No		A6TW	Preoperative	TUMOR FREE	NO	YES	Partial Remission/Response	NO	Primary Tumor	1.00	NO	YES		Cerebral Cortex	YES	NO	YES	S9	Supratentorial, Frontal Lobe	Central nervous system		NO	LIVING	2011.00
422	NO	No	C71.0	18688.00	C71.0	YES			150.00		NO	100.00	Left	YES	IHC	YES		NO	NO	NO	NO	G3	NO	FALSE	No	TCGA-S9-A6TX.B2F18877-6F79-40BE-8F35-	A6TX	Preoperative	WITH TUMOR	NO	NO	Complete Remission/Response	NO	Primary Tumor	1.00	NO	YES		Cerebral Cortex	YES	NO	YES	S9	Supratentorial, Temporal Lobe	Central nervous system	A	NO	LIVING	2011.00
423	NO	No	C71.0	18323.00	C71.0	YES			130.00		NO	100.00	Left	NO	IHC	YES		NO	NO	YES	NO	G2	NO	FALSE	No	TCGA-S9-A6TY.13C1027F-56A6-4710-946D-	A6TY	Preoperative	WITH TUMOR	YES	NO	Partial Remission/Response	NO	Primary Tumor	1.00	YES	NO		Cerebral Cortex	YES	NO	YES	S9	Supratentorial, Temporal Lobe	Central nervous system	A	NO	LIVING	2011.00
424	NO	No	C71.0	36586.00	C71.0	YES						100.00	Left	YES	IHC	YES		NO	YES	NO	NO	G2	NO		No		A6TZ	Preoperative	WITH TUMOR	NO	YES	Partial Remission/Response	NO	Primary Tumor	1.00	NO	NO		Cerebral Cortex	NO	NO	YES	S9	Supratentorial, Frontal Lobe	Central nervous system		NO	LIVING	2011.00
425	NO	No	C71.0	43891.00	C71.0	YES			140.00		NO	100.00	Right	NO	IHC	YES		NO	NO	NO	NO	G3	NO	FALSE	No	TCGA-S9-A6U0.D07453BE-76A4-42D0-B075-	A6U0	Preoperative	WITH TUMOR	NO	NO	Partial Remission/Response	YES	Primary Tumor	1.00	YES	NO		Cerebral Cortex	YES	NO	YES	S9	Supratentorial, Temporal Lobe	Central nervous system	A	NO	DECEASED	2012.00
426	NO	No	C71.0	43891.00	C71.0	YES			230.00		NO	100.00	Right	NO	IHC	YES		NO	NO	NO	NO	G3	NO	FALSE	No	TCGA-S9-A6U1.400A5278-9C45-4FF9-AEF8-	A6U1	Preoperative	TUMOR FREE	NO	NO	Complete Remission/Response	NO	Primary Tumor	1.00	NO	YES		Cerebral Cortex	YES	NO	YES	S9	Supratentorial, Frontal Lobe	Central nervous system	A	NO	LIVING	2012.00
427	NO	No	C71.0	18323.00	C71.0	YES			140.00		NO	100.00	Right	YES	IHC	YES		NO	NO	NO	NO	G2	NO	FALSE	No	TCGA-S9-A6U2.C9E09216-EB85-433E-897C-	A6U2	Preoperative	WITH TUMOR	YES	NO	Partial Remission/Response	YES	Primary Tumor	1.00	YES	NO		Cerebral Cortex	NO	NO	YES	S9	Supratentorial, Frontal Lobe	Central nervous system	A	NO	LIVING	2012.00
428	NO	No	C71.0	36586.00	C71.0	YES			130.00		NO	100.00	Right	YES	IHC	YES		NO	NO	NO	NO	G2	NO	FALSE	No	TCGA-S9-A6U5.0A1B0FCA-6B68-4BA3-946D-	A6U5	Preoperative	TUMOR FREE	YES	NO	Complete Remission/Response	NO	Primary Tumor	1.00	YES	NO		Cerebral Cortex	NO	NO	YES	S9	Supratentorial, Temporal Lobe	Central nervous system	A	NO	LIVING	2011.00
429	NO	No	C71.0	43891.00	C71.0	YES	NO		170.00		NO	100.00	Left	YES	IHC	YES		NO	NO	NO	NO	G3	NO	FALSE	No	TCGA-S9-A6U6.741221A4-D7C5-45F8-9D69-	A6U6	Preoperative	TUMOR FREE	NO	NO	Complete Remission/Response	YES	Primary Tumor	1.00	NO	NO		Cerebral Cortex	NO	NO	YES	S9	Supratentorial, Temporal Lobe	Central nervous system	A	NO	LIVING	2012.00
430	NO	No	C71.0	36586.00	C71.0	YES			100.00		NO	100.00	Left			NO		NO	NO	NO	NO	G2	YES	FALSE	No	TCGA-S9-A6U8.6999D0D0-E961-478C-8935-	A6U8	Preoperative	WITH TUMOR	NO	NO	Partial Remission/Response	NO	Primary Tumor	1.00	YES	NO		White Matter	NO	NO	YES	S9	Supratentorial, Temporal Lobe	Central nervous system	A	NO	DECEASED	2005.00

431	NO	No	C71.0	43891.00	C71.0	YES			160.00		NO		Right			NO		NO	YES	NO	NO	G3	NO	FALSE	No	TCGA-S9-A6U9.3F2EBA7C-1491-4995-9AE4-	A6U9		WITH TUMOR	YES	NO	Partial Remission/Response	YES	Primary Tumor	1.00	YES	NO		White Matter	NO	NO	YES	S9	Supratentorial, Frontal Lobe	Central nervous system	A	NO	LIVING	2006.00
432	NO	No	C71.0	43891.00	C71.0	YES			80.00		NO	60.00	Right			NO		NO	YES	NO	NO	G3	YES	FALSE	No	TCGA-S9-A6UA.7F0ED679-C54A-4B51-ACD8-	A6UA	Preoperative	WITH TUMOR	NO	NO	Progressive Disease	NO	Primary Tumor	1.00	NO	NO		Deep Gray (e.g.basal ganglia, thalamus)	NO	NO	YES	S9	Supratentorial, Temporal Lobe	Central nervous system	A	NO	DECEASED	2008.00
433	NO	No	C71.0	18323.00	C71.0	YES			120.00		NO	100.00	Right			NO		NO	NO	NO	NO	G2	NO	FALSE	No	TCGA-S9-A6UB.F71CEABA-8472-49C7-B618-	A6UB	Preoperative	TUMOR FREE	NO	NO	Complete Remission/Response	NO	Primary Tumor	1.00	NO	NO		Cerebral Cortex	NO	NO	YES	S9	Supratentorial, Frontal Lobe	Central nervous system	A	NO	LIVING	2005.00
434	NO	No	C71.0	18688.00	C71.0	YES			160.00		NO	100.00	Left			NO		NO	NO	NO	NO	G3	NO	FALSE	No	TCGA-S9-A6WD.5F24A61F-39AA-426A-89D2-	A6WD	Preoperative	WITH TUMOR	YES	NO	Partial Remission/Response	NO	Primary Tumor	1.00	YES	NO		Cerebral Cortex	YES	NO	YES	S9	Supratentorial, Parietal Lobe	Central nervous system	A	NO	LIVING	2008.00
435	NO	No	C71.0	18323.00	C71.0	YES			140.00		NO	100.00	Right			NO		NO	NO	NO	NO	G2	NO	FALSE	No	TCGA-S9-A6WE.F17B7D4A-F22C-472A-A84F-	A6WE	Preoperative	WITH TUMOR	YES	YES	Partial Remission/Response	NO	Primary Tumor	1.00	NO	YES		Cerebral Cortex	YES	NO	YES	S9	Supratentorial, Frontal Lobe	Central nervous system	A	NO	LIVING	2004.00
436	NO	No	C71.0	43891.00	C71.0	YES			90.00		NO	100.00	Right			NO		NO	NO	NO	NO	G3	NO	FALSE	No	TCGA-S9-A6WG.82E91E98-4371-46DC-912C-	A6WG	Preoperative	WITH TUMOR	YES	YES	Progressive Disease	YES	Primary Tumor	1.00	YES	NO		Cerebral Cortex	YES	NO	YES	S9	Supratentorial, Frontal Lobe	Central nervous system	A	NO	LIVING	2007.00
437	NO	No	C71.0	30011.00	C71.0	YES			180.00		NO	80.00	Right	YES	IHC	YES		NO	YES	NO	YES	G2	NO	FALSE	No	TCGA-S9-A6WH.ED9DEB9C-4DC6-4743-8402-	A6WH	Preoperative	WITH TUMOR	NO	NO	Partial Remission/Response	YES	Primary Tumor	1.00	NO	YES		Cerebral Cortex	NO	NO	YES	S9	Supratentorial, Temporal Lobe	Central nervous system	A	NO	LIVING	2011.00
438	NO	No	C71.0	30011.00	C71.0	YES						100.00	Right			NO		NO	YES	NO	NO	G2	NO		No		A6WI	Preoperative	WITH TUMOR	NO	NO	Partial Remission/Response	YES	Primary Tumor	1.00	NO	NO		White Matter	NO	NO	YES	S9	Supratentorial, Frontal Lobe	Central nervous system		NO	LIVING	2006.00
439	NO	No	C71.0	43891.00	C71.0	YES						100.00	Right	YES	IHC	YES		NO	NO	NO	NO	G3	NO		No		A6WL	Preoperative	WITH TUMOR	YES	NO	Progressive Disease	NO	Primary Tumor	1.00	YES	NO		Cerebral Cortex	NO	NO	YES	S9	Supratentorial, Frontal Lobe	Central nervous system		NO	LIVING	2012.00
440	NO	No	C71.0	43891.00	C71.0	YES						90.00	Left	NO	IHC	YES		NO	NO	NO	NO	G3	YES		No		A6WM	Preoperative	WITH TUMOR	NO	NO	Partial Remission/Response	YES	Primary Tumor	1.00	NO	NO		Cerebral Cortex	YES	NO	YES	S9	Supratentorial, Temporal Lobe	Central nervous system		NO	LIVING	2012.00
441	NO	No	C71.0	43891.00	C71.0	YES			140.00		NO	90.00	Left	NO	IHC	YES		NO	NO	NO	NO	G3	YES	FALSE	No	TCGA-S9-A6WN.DF6F3A7F-700B-4AB7-8E0A-	A6WN	Preoperative	WITH TUMOR	YES	YES	Progressive Disease	YES	Primary Tumor	1.00	YES	YES		Cerebral Cortex	YES	NO	YES	S9	Supratentorial, Frontal Lobe	Central nervous system	A	NO	LIVING	2013.00
442	NO	No	C71.0	36586.00	C71.0	YES						100.00	Left	YES	IHC	YES		NO	NO	NO	NO	G2	NO		No		A6WO	Preoperative	WITH TUMOR	YES	NO	Progressive Disease	NO	Primary Tumor	1.00	YES	NO		White Matter	NO	NO	YES	S9	Supratentorial, Frontal Lobe	Central nervous system		NO	LIVING	2013.00
443	NO	No	C71.0	30011.00	C71.0	YES			180.00		NO	100.00	Right	YES	IHC	YES		NO	NO	NO	NO	G3	NO	FALSE	No	TCGA-S9-A6WP.55030C3A-6D25-4EB0-9B6C-	A6WP	Preoperative	WITH TUMOR	NO	NO	Partial Remission/Response	NO	Primary Tumor	1.00	YES	NO		Cerebral Cortex	YES	NO	YES	S9	Supratentorial, Frontal Lobe	Central nervous system	A	NO	LIVING	2013.00
444	NO	No	C71.0	30011.00	C71.0	YES	NO		150.00		NO	100.00	Left	YES	IHC	YES		NO	YES	YES	NO	G2	NO	FALSE	No	TCGA-S9-A6WQ.E2BDCD9B-B785-46F6-A55C-	A6WQ	Preoperative	WITH TUMOR	NO	NO	Partial Remission/Response	NO	Primary Tumor	1.00	YES	YES		Cerebral Cortex	NO	NO	YES	S9	Supratentorial, Frontal Lobe	Central nervous system	A	NO	LIVING	2013.00
445	NO	No	C71.0	30011.00	C71.0	YES			150.00		NO	100.00	Left	YES	IHC	YES		NO	NO	NO	NO	G2	NO	FALSE	No	TCGA-S9-A7IQ.2E181A31-FB43-4A8C-87E2-	A7IQ	Preoperative	WITH TUMOR	NO	NO	Partial Remission/Response	NO	Primary Tumor	1.00	NO	YES		Cerebral Cortex	NO	NO	YES	S9	Supratentorial, Frontal Lobe	Central nervous system	A	NO	LIVING	2011.00
446	NO	No	C71.0	43891.00	C71.0	YES			160.00		NO	60.00	Right			NO		NO	YES	NO	NO	G3	NO	FALSE	No	TCGA-S9-A7IS.B8C99516-595F-4A10-B400-	A7IS	Preoperative	WITH TUMOR	NO	NO	Progressive Disease	YES	Primary Tumor	1.00	NO	NO		Not listed in Medical Record	NO	NO	YES	S9	Supratentorial, Frontal Lobe	Central nervous system	A	NO	DECEASED	2004.00
447	NO	No	C71.0	43891.00	C71.0	YES			80.00		NO	100.00	Right			NO		NO	NO	NO	YES	G3	YES	FALSE	No	TCGA-S9-A7IX.BCA5F1A6-7BAB-416F-81A1-	A7IX	Preoperative	WITH TUMOR	YES	NO	Progressive Disease	YES	Primary Tumor	1.00	NO	NO		White Matter	NO	NO	YES	S9	Supratentorial, Temporal Lobe	Central nervous system	A	NO	DECEASED	2012.00
448	NO	No	C71.0	30011.00	C71.0	YES			100.00		NO	80.00	Right	YES	IHC	YES		NO	YES	NO	YES	G3	NO	FALSE	No	TCGA-S9-A7IY.9223C347-C93C-4B31-BEEE-	A7IY	Preoperative	WITH TUMOR	NO	YES	Partial Remission/Response	YES	Primary Tumor	1.00	NO	NO		White Matter	YES	NO	YES	S9	Supratentorial, Frontal Lobe	Central nervous system	A	NO	LIVING	2012.00
449	NO	No	C71.0	43891.00	C71.0	YES			130.00		NO	90.00	Left	YES	IHC	YES		NO	NO	NO	NO	G3	YES	FALSE	No	TCGA-S9-A7IZ.82CF208A-A8F2-4BE1-85DF-	A7IZ	Preoperative	WITH TUMOR	YES	NO	Partial Remission/Response	YES	Primary Tumor	1.00	YES	NO		Cerebral Cortex	NO	NO	YES	S9	Supratentorial, Frontal Lobe	Central nervous system	A	NO	LIVING	2013.00
450	NO	No	C71.0	18688.00	C71.0	YES			140.00		NO	100.00	Right	NO	IHC	YES		NO	NO	NO	NO	G3	YES	FALSE	No	TCGA-S9-A7J0.B1D1504E-B9FB-4974-A8B6-	A7J0	Preoperative	WITH TUMOR	YES	NO	Partial Remission/Response	YES	Primary Tumor	1.00	YES	NO		Cerebral Cortex	YES	NO	YES	S9	Supratentorial, Frontal Lobe	Central nervous system	A	NO	LIVING	2013.00
451	NO	No	C71.0	18323.00	C71.0	YES			250.00		NO	60.00	Right	YES	IHC	YES		YES	NO	NO	NO	G2	NO	FALSE	No	TCGA-S9-A7J1.60CE3E08-7253-47C7-B7FF-	A7J1	Preoperative	WITH TUMOR	YES	NO	Partial Remission/Response	NO	Primary Tumor	1.00	YES	NO		Cerebral Cortex	NO	NO	YES	S9	Supratentorial, Temporal Lobe	Central nervous system	A	NO	LIVING	2013.00
452	NO	No	C71.0	18688.00	C71.0	YES			200.00		NO	80.00	Left	YES	IHC	YES		YES	YES	NO	NO	G3	NO	FALSE	No	TCGA-S9-A7J2.F7F8D6E7-0C27-4FAC-96E0-	A7J2	Preoperative	WITH TUMOR	YES	NO	Partial Remission/Response	NO	Primary Tumor	1.00	YES	NO		Cerebral Cortex	YES	NO	YES	S9	Supratentorial, Frontal Lobe	Central nervous system	A	NO	LIVING	2013.00
453	NO	No	C71.0	18688.00	C71.0	YES			120.00		NO	100.00	Left	YES	IHC	YES		NO	NO	NO	NO	G3	NO	FALSE	No	TCGA-S9-A7J3.EC6B158C-2D75-43CF-8D80-	A7J3	Preoperative	WITH TUMOR	YES	YES	Partial Remission/Response	YES	Primary Tumor	1.00	YES	NO		White Matter	YES	NO	YES	S9	Supratentorial, Parietal Lobe	Central nervous system	A	NO	LIVING	2013.00
454	NO	No	C71.0	43891.00	C71.0	YES						100.00	Left	YES	IHC	YES		NO	NO	NO	NO	G3	YES		No		A7QW	Preoperative	WITH TUMOR	NO	NO	Complete Remission/Response	YES	Primary Tumor	1.00	NO	NO		Cerebral Cortex	NO	YES	NO	S9	Supratentorial, Frontal Lobe	Central nervous system		NO	LIVING	2013.00
455	NO	No	C71.0	43891.00	C71.0	YES			150.00		NO	100.00	Right	YES	IHC	YES		NO	NO	NO	NO	G3	NO	FALSE	No	TCGA-S9-A7QX.C0F30532-18DA-4E77-A2CC-	A7QX	Preoperative	WITH TUMOR	YES	NO	Partial Remission/Response	YES	Primary Tumor	1.00	YES	YES		Cerebral Cortex	YES	YES	NO	S9	Supratentorial, Temporal Lobe	Central nervous system	A	NO	LIVING	2013.00
456	NO	No	C71.0	30011.00	C71.0	YES			60.00		NO	100.00	Left	NO	IHC	YES		NO	NO	NO	NO	G2	NO	FALSE	No	TCGA-S9-A7QY.7833FCD6-2858-4428-9E9C-	A7QY	Preoperative	WITH TUMOR	YES	YES	Partial Remission/Response	NO	Primary Tumor	1.00	YES	NO		Cerebral Cortex	YES	NO	YES	S9	Supratentorial, Frontal Lobe	Central nervous system	A	NO	LIVING	2012.00
457	NO	No	C71.0	18323.00	C71.0	YES			120.00		NO	100.00	Left	YES	IHC	YES		NO	NO	NO	NO	G2	YES	FALSE	No	TCGA-S9-A7QZ.2DBE4A35-E160-47C2-B000-	A7QZ	Preoperative	WITH TUMOR	YES	NO	Partial Remission/Response	NO	Primary Tumor	1.00	YES	NO		Cerebral Cortex	NO	NO	YES	S9	Supratentorial, Frontal Lobe	Central nervous system	A	NO	LIVING	2012.00
458	NO	No	C71.0	18323.00	C71.0	YES			200.00		NO	90.00	Midline			NO		NO	NO	NO	NO	G2	NO	FALSE	No	TCGA-S9-A7R1.7E6FECAF-5861-4BE0-B867-	A7R1	Preoperative	WITH TUMOR	NO	NO	Partial Remission/Response	YES	Primary Tumor	1.00	YES	NO		Not listed in Medical Record	NO	NO	YES	S9	Supratentorial, Frontal Lobe	Central nervous system	A	YES	DECEASED	1999.00
459	NO	No	C71.0	43891.00	C71.0	YES			120.00		NO	90.00	Left			NO			NO	NO	NO	G3	NO	FALSE	No	TCGA-S9-A7R2.2907EB1D-32CC-4BB7-B485-	A7R2	Preoperative	WITH TUMOR	YES	NO	Progressive Disease	YES	Primary Tumor	1.00	YES	NO		White Matter	NO	NO	YES	S9	Supratentorial, Temporal Lobe	Central nervous system	A	NO	DECEASED	2008.00
460	NO	No	C71.0	36586.00	C71.0	YES			80.00		NO	100.00	Left			NO		NO	NO	NO	NO	G2	NO	FALSE	No	TCGA-S9-A7R3.769B080E-C2D2-492E-9B82-	A7R3	Preoperative	TUMOR FREE	NO	NO	Partial Remission/Response	NO	Primary Tumor	1.00	NO	NO		Cerebral Cortex	NO	NO	YES	S9	Supratentorial, Frontal Lobe	Central nervous system	A	NO	LIVING	2006.00
461	NO	No	C71.0	43891.00	C71.0	YES			120.00		NO	90.00	Left	YES	IHC	YES		NO	NO	NO	NO	G3	NO	FALSE	No	TCGA-S9-A7R4.6AE37879-1BB7-4C4A-B318-	A7R4	Preoperative	WITH TUMOR	YES	NO	Partial Remission/Response	YES	Primary Tumor	1.00	YES	NO		Cerebral Cortex	YES	NO	YES	S9	Supratentorial, Frontal Lobe	Central nervous system	A	NO	LIVING	2012.00
462	NO	No	C71.0	36586.00	C71.0	YES			70.00		NO	80.00	Left	YES	IHC	YES		NO	NO	NO	NO	G2	YES	FALSE	No	TCGA-S9-A7R7.0C30BB62-F9E0-4702-B488-	A7R7	Preoperative	WITH TUMOR	YES	YES	Progressive Disease	YES	Primary Tumor	1.00	YES	NO		Cerebral Cortex	NO	NO	YES	S9	Supratentorial, Frontal Lobe	Central nervous system	A	NO	LIVING	2006.00
463	NO	No	C71.0	43891.00	C71.0	YES			110.00		NO	90.00	Right			NO			NO	NO	NO	G3	YES	FALSE	No	TCGA-S9-A7R8.7287E887-2783-4416-8C61-	A7R8	Preoperative	WITH TUMOR	YES	YES	Progressive Disease	YES	Primary Tumor	1.00	YES	NO		Not listed in Medical Record	NO	NO	YES	S9	Supratentorial, Frontal Lobe	Central nervous system	A	NO	DECEASED	2004.00
464	NO	No	C71.0	43891.00	C71.0	YES						80.00	Right	NO	IHC	YES		NO	NO	NO	YES	G3	YES		No		A89V	Preoperative	WITH TUMOR	NO	NO	Progressive Disease	YES	Primary Tumor	1.00	NO	YES		Cerebral Cortex	YES	NO	YES	S9	Posterior Fossa, Cerebellum	Central nervous system		NO	LIVING	2012.00
465	NO	No	C71.0	43891.00	C71.0	YES	NO		70.00		NO	70.00	Right	YES	IHC	YES		NO	NO	NO	NO	G3	NO	FALSE	No	TCGA-S9-A89Z.DAAF016F-5C66-4E31-B57A-	A89Z	Preoperative	WITH TUMOR	YES	YES	Partial Remission/Response	YES	Primary Tumor	1.00	YES	NO		White Matter	YES	YES	NO	S9	Supratentorial, Frontal Lobe	Central nervous system	A	NO	LIVING	2013.00
466	NO	No	C71.0	43891.00	C71.0	YES	NO		80.00		NO	90.00	Left	NO				NO	NO	NO	NO	G3	NO	FALSE	No	TCGA-TM-A7C3.8716B17F-6119-4999-8F12-	A7C3	Preoperative	WITH TUMOR			Complete Remission/Response	NO	Primary Tumor	1.00	YES	NO		Not listed in Medical Record	NO	NO	YES	TM	Supratentorial, Temporal Lobe	Central nervous system	A	NO	DECEASED	2009.00

467	NO	No	C71.0	36586.00	C71.0	YES	NO		80.00		NO	90.00	Right			NO		NO	NO		NO	G2	NO	FALSE	No	TCGA-TM-A7C4.4097D689-A950-486D-9CDA-	A7C4	Preoperative	TUMOR FREE			Complete Remission/Response	NO	Primary Tumor	1.00	YES	NO		Not listed in Medical Record	NO	NO	YES	TM	Supratentorial, Temporal Lobe	Central nervous system	A	NO	LIVING	2010.00
468	NO	No	C71.0	30011.00	C71.0	YES	NO		140.00		NO	100.00	Left			NO		NO	NO	NO	NO	G2	NO	FALSE	No	TCGA-TM-A7C5.CEA3E51F-5E16-4778-9684-	A7C5	Preoperative	TUMOR FREE			Complete Remission/Response	NO	Primary Tumor	1.00	YES	NO		Not listed in Medical Record	NO	NO	YES	TM	Supratentorial, Parietal Lobe	Central nervous system	A	NO	LIVING	2010.00
469	NO	No	C71.0	36586.00	C71.0	YES	NO		60.00		NO	100.00	Left			NO		NO	NO	NO	NO	G2	NO	FALSE	No	TCGA-TM-A7CA.FA7FFEBC-9D1C-42E6-BD8E-	A7CA	Preoperative	TUMOR FREE			Complete Remission/Response	NO	Primary Tumor	1.00	YES	NO		Not listed in Medical Record	NO	NO	YES	TM	Supratentorial, Frontal Lobe	Central nervous system	A	NO	LIVING	2011.00
470	NO	No	C71.0	36586.00	C71.0	YES			80.00		NO	100.00	Right			NO		NO	NO	NO	NO	G2	NO	FALSE	No	TCGA-TM-A7CF.CC5699A6-8FC6-438D-ACC4-	A7CF	Preoperative	WITH TUMOR			Complete Remission/Response	YES	Primary Tumor	1.00	YES	NO		Not listed in Medical Record	YES	NO	YES	TM	Supratentorial, Frontal Lobe	Central nervous system	A	NO	LIVING	2009.00
471	NO	No	C71.0	36586.00	C71.0	YES			100.00		NO	100.00	Right			NO		NO	NO	NO	NO	G2	NO	FALSE	No	TCGA-TM-A7CF.E7D22CFE-C4C9-4E2F-AB45-	A7CF	Preoperative	WITH TUMOR			Complete Remission/Response	YES	Recurrent Tumor	2.00	YES	NO		Not listed in Medical Record	YES	NO	YES	TM	Supratentorial, Frontal Lobe	Central nervous system	A	NO	LIVING	2009.00
472	NO	No	C71.0	43891.00	C71.0	YES						90.00	Left	NO	IHC	YES		NO	NO	NO	YES	G3	NO		No		A84B	Preoperative	WITH TUMOR			Progressive Disease	NO	Primary Tumor	1.00	YES	YES		Not listed in Medical Record	YES	NO	YES	TM	Supratentorial, Parietal Lobe	Central nervous system		NO	DECEASED	2012.00
473	NO	No	C71.0	36586.00	C71.0	YES			80.00		NO	80.00	Midline	NO	IHC	YES		NO	YES	NO	YES	G2	NO	FALSE	No	TCGA-TM-A84C.506E57F2-58E8-44D0-BEBC-	A84C	Preoperative	WITH TUMOR			Progressive Disease	NO	Primary Tumor	1.00	NO	YES		Not listed in Medical Record	YES	NO	YES	TM	Posterior Fossa, Brain Stem	Central nervous system	A	YES	DECEASED	2012.00
474	NO	No	C71.0	43891.00	C71.0	YES			60.00		NO	100.00	Right			NO		NO	NO	NO	NO	G3	NO	FALSE	No	TCGA-TM-A84F.5142804B-6A45-44E9-99A1-	A84F	Preoperative	TUMOR FREE			Complete Remission/Response	YES	Primary Tumor	1.00	NO	NO		Not listed in Medical Record	NO	NO	YES	TM	Supratentorial, Frontal Lobe	Central nervous system	A	YES	LIVING	2009.00
475	NO	No	C71.0	18688.00	C71.0	YES						100.00	Right			NO		NO	NO	NO	NO	G3	NO		No		A84G	Preoperative	TUMOR FREE			Complete Remission/Response	YES	Primary Tumor	1.00	NO	NO		Not listed in Medical Record	NO	NO	YES	TM	Supratentorial, Frontal Lobe	Central nervous system		YES	LIVING	2010.00
476	NO	No	C71.0	30011.00	C71.0	YES			60.00		NO	90.00	Right			NO		NO	YES	NO	NO	G3	NO	FALSE	No	TCGA-TM-A84H.654A67F7-8755-4BF6-9674-	A84H	Preoperative	TUMOR FREE			Complete Remission/Response	YES	Primary Tumor	1.00	NO	NO		Not listed in Medical Record	NO	NO	YES	TM	Supratentorial, Frontal Lobe	Central nervous system	A	NO	LIVING	2012.00
477	NO	No	C71.0	43891.00	C71.0	YES						90.00	Left			NO		NO	NO	NO	YES	G3	NO		No		A84I	Preoperative	TUMOR FREE			Complete Remission/Response	YES	Primary Tumor	1.00	NO	NO		Not listed in Medical Record	NO	NO	YES	TM	Supratentorial, Parietal Lobe	Central nervous system		NO	LIVING	2012.00
478	NO	No	C71.0	18688.00	C71.0	YES			50.00		NO	80.00	Left			NO		NO	YES	NO	YES	G3	YES	FALSE	No	TCGA-TM-A84J.D3563493-4083-479F-A172-	A84J	Preoperative	WITH TUMOR			Progressive Disease	YES	Primary Tumor	1.00	YES	NO		Not listed in Medical Record	YES	NO	YES	TM	Supratentorial, Occipital Lobe	Central nervous system	A	YES	LIVING	2012.00
479	NO	No	C71.0	30011.00	C71.0	YES						90.00	Left			NO		NO	NO	NO	NO	G2	YES		No		A84L	Preoperative	WITH TUMOR			Complete Remission/Response	NO	Primary Tumor	1.00	YES	NO		Not listed in Medical Record	NO	NO	YES	TM	Supratentorial, Temporal Lobe	Central nervous system		NO	DECEASED	2010.00
480	NO	No	C71.0	18688.00	C71.0	YES			10.00		NO	100.00	Right			NO		NO	NO	NO	YES	G3	NO	FALSE	No	TCGA-TM-A84M.A9063552-B6AE-4CD9-9A0C-	A84M	Preoperative	TUMOR FREE			Complete Remission/Response	YES	Primary Tumor	1.00	NO	NO		Not listed in Medical Record	YES	NO	YES	TM	Supratentorial, Frontal Lobe	Central nervous system	A	NO	LIVING	2012.00
481	NO	No	C71.0	18688.00	C71.0	YES			60.00		NO	90.00	Right			NO		NO	NO	NO	NO	G3	NO	FALSE	No	TCGA-TM-A84O.6E28D951-BB27-4E32-A681-	A84O	Preoperative	WITH TUMOR			Complete Remission/Response	YES	Primary Tumor	1.00	YES	NO		Not listed in Medical Record	NO	NO	YES	TM	Supratentorial, Not Otherwise Specified	Central nervous system	A	NO	DECEASED	2011.00
482	NO	No	C71.0	36586.00	C71.0	YES			50.00		NO	80.00	Left			NO		NO	YES	NO	NO	G2	YES	FALSE	No	TCGA-TM-A84Q.CE780D35-9DBE-4596-812C-	A84Q	Preoperative	TUMOR FREE			Complete Remission/Response	YES	Primary Tumor	1.00	NO	NO		Not listed in Medical Record	NO	NO	YES	TM	Supratentorial, Temporal Lobe	Central nervous system	A	NO	LIVING	2012.00
483	NO	No	C71.0	18323.00	C71.0	YES			140.00		NO	80.00	Left	NO	Sequence Analysis	YES		NO	NO	NO	NO	G2	NO	FALSE	No	TCGA-TM-A84R.3182D6DB-4B2D-49B0-A907-	A84R	Preoperative	TUMOR FREE			Complete Remission/Response	NO	Primary Tumor	1.00	YES	NO		Not listed in Medical Record	NO	NO	YES	TM	Supratentorial, Frontal Lobe	Central nervous system	A	NO	LIVING	2012.00
484	NO	No	C71.0	18688.00	C71.0	YES			110.00		NO	80.00	Left	YES	IHC	YES		NO	NO	NO	YES	G3	NO	FALSE	No	TCGA-TM-A84S.286FC4C6-07CD-4056-B6EE-	A84S	Preoperative	TUMOR FREE			Complete Remission/Response	NO	Primary Tumor	1.00	YES	NO		Not listed in Medical Record	NO	NO	YES	TM	Supratentorial, Frontal Lobe	Central nervous system	A	YES	LIVING	2013.00
485	NO	No	C71.0	30011.00	C71.0	YES						90.00	Left	YES	IHC	YES		NO	NO	NO	NO	G2	NO		No		A84T	Preoperative	WITH TUMOR			Stable Disease	NO	Primary Tumor	1.00	YES	YES		Not listed in Medical Record	NO	NO	YES	TM	Supratentorial, Frontal Lobe	Central nervous system		NO	LIVING	2012.00
486	NO	No	C71.0	18688.00	C71.0	YES			130.00		NO		Right			NO		NO	YES	NO	NO	G3	NO	FALSE	No	TCGA-TQ-A7RF.F4103666-DB5D-4286-8769-	A7RF	Preoperative	WITH TUMOR	YES	YES	Partial Remission/Response	YES	Primary Tumor	1.00	YES	YES		White Matter	YES	NO	YES	TQ	Supratentorial, Temporal Lobe	Central nervous system	A	YES	LIVING	2012.00
487	NO	No	C71.0	30011.00	C71.0	YES	NO		530.00		NO	100.00	Left					NO	NO	NO	NO	G2	NO	FALSE	No	TCGA-TQ-A7RG.396F6EAB-2B8F-4054-AACA-	A7RG	Preoperative	WITH TUMOR	YES	YES	Partial Remission/Response	NO	Primary Tumor	1.00	YES	NO		White Matter	YES	NO	YES	TQ	Supratentorial, Temporal Lobe	Central nervous system	A	YES	LIVING	2012.00
488	NO	No	C71.0	30011.00	C71.0	YES	NO		130.00		NO		Right			NO		NO	YES	NO	NO	G2	NO	FALSE	No	TCGA-TQ-A7RH.489D1069-B12F-4250-9FD0-	A7RH		WITH TUMOR	YES	YES	Partial Remission/Response	YES	Primary Tumor	1.00	YES	NO		White Matter	YES	NO	YES	TQ	Supratentorial, Temporal Lobe	Central nervous system	A	NO	LIVING	2013.00
489	NO	No	C71.0	18323.00	C71.0	YES	NO						Left			NO		YES	NO	NO	NO	G2	NO		No		A7RI	Preoperative	TUMOR FREE	YES	YES		NO	Primary Tumor	1.00	YES	NO		White Matter	NO	NO	YES	TQ	Supratentorial, Parietal Lobe	Central nervous system		NO	LIVING	2012.00
490	NO	No	C71.0	30011.00	C71.0	YES	NO		50.00		NO		Left			NO		NO	NO	NO	YES	G2	NO	FALSE	No	TCGA-TQ-A7RJ.9C4E4467-F9B6-456F-8138-	A7RJ	Preoperative	WITH TUMOR	NO	NO	Stable Disease	YES	Primary Tumor	1.00	NO	YES		White Matter	YES	NO	YES	TQ	Supratentorial, Parietal Lobe	Central nervous system	A	NO	LIVING	2011.00
491	NO	No	C71.0	18323.00	C71.0	YES	NO		330.00		NO	90.00	Left			NO		NO	YES	NO	NO	G2	YES	FALSE	No	TCGA-TQ-A7RK.740E07A6-7579-4967-827C-	A7RK	Preoperative	TUMOR FREE	YES	NO	Complete Remission/Response	NO	Primary Tumor	1.00	NO	NO		White Matter	NO	NO	YES	TQ	Supratentorial, Frontal Lobe	Central nervous system	A	NO	LIVING	2010.00
492	NO	No	C71.0	18323.00	C71.0	YES	NO		60.00		NO	90.00	Left			NO		NO	YES	NO	NO	G2	YES	TRUE	No	TCGA-TQ-A7RK.5452FDC5-EB15-49DE-A0CC-	A7RK	Preoperative	TUMOR FREE	YES	NO	Complete Remission/Response	NO	Recurrent Tumor	2.00	NO	NO		White Matter	NO	NO	YES	TQ	Supratentorial, Frontal Lobe	Central nervous system	B	NO	LIVING	2010.00
493	NO	No	C71.0	30011.00	C71.0	YES	NO					90.00	Left			NO		NO	YES	NO	NO	G3	NO		No		A7RM	Pre-Adjuvant Therapy	WITH TUMOR	YES	NO	Partial Remission/Response	YES	Primary Tumor	1.00	YES	NO		Cerebral Cortex	YES	NO	YES	TQ	Supratentorial, Temporal Lobe	Central nervous system		NO	LIVING	2011.00
494	NO	No	C71.0	18323.00	C71.0	YES	NO		150.00		NO	100.00	Left			NO		NO	NO	NO	NO	G2	NO	FALSE	No	TCGA-TQ-A7RN.87A66ECE-00EE-4D45-83BF-	A7RN	Pre-Adjuvant Therapy	WITH TUMOR	YES	NO	Partial Remission/Response	YES	Primary Tumor	1.00	YES	NO		Cerebral Cortex	YES	NO	YES	TQ	Supratentorial, Temporal Lobe	Central nervous system	A	NO	LIVING	2011.00
495	YES	No	C71.0	30011.00	C71.0	YES	NO		80.00		NO	100.00	Left			NO		NO	NO	NO	NO	G2	NO	FALSE	No	TCGA-TQ-A7RO.41735167-9814-4D95-A3D4-	A7RO	Other	WITH TUMOR	YES	YES	Partial Remission/Response	NO	Primary Tumor	1.00	YES	NO		Cerebral Cortex	NO	NO	YES	TQ	Supratentorial, Temporal Lobe	Central nervous system	A	NO	LIVING	2011.00
496	NO	No	C71.0	30011.00	C71.0	YES	NO		400.00		NO	90.00	Left			NO		YES	NO	NO	NO	G2	NO	FALSE	No	TCGA-TQ-A7RP.27A97401-6DB1-4439-A06D-	A7RP	Pre-Adjuvant Therapy	WITH TUMOR	YES	NO	Partial Remission/Response	YES	Primary Tumor	1.00	YES	NO		Cerebral Cortex	YES	NO	YES	TQ	Supratentorial, Temporal Lobe	Central nervous system	A	NO	LIVING	2012.00
497	NO	No	C71.0	18323.00	C71.0	YES	NO		140.00		NO	90.00	Left			NO		NO	NO	NO	NO	G2	NO	FALSE	No	TCGA-TQ-A7RQ.627A2B25-6FC6-4CF3-9EA8-	A7RQ	Pre-Adjuvant Therapy	TUMOR FREE	YES	YES	Complete Remission/Response	NO	Primary Tumor	1.00	YES	NO		Cerebral Cortex	YES	NO	YES	TQ	Supratentorial, Temporal Lobe	Central nervous system	A	NO	LIVING	2012.00
498	NO	No	C71.0	30011.00	C71.0	YES	NO		220.00		NO	100.00	Right			NO		NO	NO	NO	NO	G2	NO	FALSE	No	TCGA-TQ-A7RR.34C9D741-8075-4FE1-8144-	A7RR	Pre-Adjuvant Therapy	TUMOR FREE	NO	NO	Partial Remission/Response	YES	Primary Tumor	1.00	YES	NO		Cerebral Cortex	YES	NO	YES	TQ	Supratentorial, Parietal Lobe	Central nervous system	A	NO	LIVING	2012.00
499	NO	No	C71.0	18323.00	C71.0	YES	NO					80.00	Left			NO		NO	NO	NO	NO	G2	NO		No		A7RU	Preoperative	WITH TUMOR	YES	NO	Partial Remission/Response	NO	Primary Tumor	1.00	YES	NO		Cerebral Cortex	NO	NO	YES	TQ	Supratentorial, Temporal Lobe	Central nervous system		NO	LIVING	2011.00
500	NO	No	C71.0	30011.00	C71.0	YES	NO		120.00		NO	80.00	Left			NO		NO	YES	NO	YES	G2	YES	FALSE	No	TCGA-TQ-A7RV.5316A44B-CF13-4A3B-AE9D-	A7RV	Preoperative	WITH TUMOR	YES	NO	Partial Remission/Response	NO	Primary Tumor	1.00	YES	YES		White Matter	NO	NO	YES	TQ	Supratentorial, Temporal Lobe	Central nervous system	A	NO	LIVING	2009.00
501	NO	No	C71.0	30011.00	C71.0	YES	NO		200.00		NO	80.00	Left			NO		NO	YES	NO	YES	G2	YES	TRUE	No	TCGA-TQ-A7RV.98CC2EDE-4503-4D22-8F18-	A7RV	Preoperative	WITH TUMOR	YES	NO	Partial Remission/Response	NO	Recurrent Tumor	2.00	YES	YES		White Matter	NO	NO	YES	TQ	Supratentorial, Temporal Lobe	Central nervous system	A	NO	LIVING	2009.00
502	NO	No	C71.0	30011.00	C71.0	YES	NO		90.00		NO	90.00	Left			NO		NO	NO	NO	NO	G2	YES	FALSE	No	TCGA-TQ-A7RW.CD826C0A-8057-4BD6-A79A-	A7RW	Pre-Adjuvant Therapy	WITH TUMOR	YES	NO	Progressive Disease	YES	Primary Tumor	1.00	YES	NO		Cerebral Cortex	YES	NO	YES	TQ	Supratentorial, Temporal Lobe	Central nervous system	A	NO	DECEASED	2010.00

503	NO	No	C71.0	18323.00	C71.0	YES	NO		130.00		NO	70.00	Left					NO	NO	NO	YES	G2	YES	TRUE	No	TCGA-TQ-A8XE.431037B9-36D3-4978-AEED-	A8XE	Preoperative	WITH TUMOR	NO	NO	Partial Remission/Response	NO	Primary Tumor	1.00	NO	YES		White Matter	NO	NO	NO	TQ	Supratentorial, Temporal Lobe	Central nervous system	A	NO	DECEASED	2010.00
504	NO	No	C71.0	18323.00	C71.0	YES	NO		200.00		NO	70.00	Left					NO	NO	NO	YES	G2	YES	TRUE	No	TCGA-TQ-A8XE.845B196D-DB8C-4AA6-A506-	A8XE	Preoperative	WITH TUMOR	NO	NO	Partial Remission/Response	NO	Recurrent Tumor	2.00	NO	YES		White Matter	NO	NO	NO	TQ	Supratentorial, Temporal Lobe	Central nervous system	A	NO	DECEASED	2010.00
505	NO	No	C71.0	18323.00	C71.0	YES					NO	90.00	Right	YES	IHC	YES		NO	NO		YES	G2	NO	FALSE	No	TCGA-VM-A8C8.11BD5D8D-FB5B-4D96-A421-	A8C8	Pre-Adjuvant Therapy					YES	Primary Tumor	1.00	NO	NO		Not listed in Medical Record	YES	NO	YES	VM	Supratentorial, Temporal Lobe	Central nervous system	A	NO	LIVING	2011.00
506	NO	No	C71.0	36586.00	C71.0	YES			120.00		NO		Left			NO		NO	NO		NO	G2	NO	FALSE	No	TCGA-VM-A8C9.572BF80A-ECDD-4EAF-A28D-	A8C9		TUMOR FREE			Complete Remission/Response	NO	Primary Tumor	1.00	YES	NO		Not listed in Medical Record	NO	NO	YES	VM	Supratentorial, Temporal Lobe	Central nervous system	A	NO	LIVING	2011.00
507	NO	No	C71.0	18323.00	C71.0	YES	NO		160.00		NO	70.00	Left			NO		YES	YES		NO	G2		FALSE	No	TCGA-VM-A8CA.44E81259-80C6-4665-95DE-	A8CA	Pre-Adjuvant Therapy	WITH TUMOR				NO	Primary Tumor	1.00	NO	NO		Not listed in Medical Record	YES	NO	YES	VM	Supratentorial, Frontal Lobe	Central nervous system	A	NO	LIVING	2011.00
508	NO	No	C71.0	18688.00	C71.0	YES	NO		100.00		NO		Left			NO		YES	NO		NO	G3		FALSE	No	TCGA-VM-A8CB.581F6861-4408-48DF-	A8CB		TUMOR FREE	YES	YES			Primary Tumor	1.00	YES	NO		Not listed in Medical Record		NO	YES	VM	Supratentorial, Frontal Lobe	Central nervous system	A	NO	LIVING	2011.00
509	NO	No	C71.0	43891.00	C71.0	YES			110.00		NO	100.00	Right	NO	IHC	YES		NO	YES		NO	G3	NO	FALSE	No	TCGA-VM-A8CD.CB64BE55-522F-4634-A7B4-	A8CD	Pre-Adjuvant Therapy	WITH TUMOR	YES	NO		YES	Primary Tumor	1.00	YES	NO		Not listed in Medical Record	NO	NO	YES	VM	Supratentorial, Temporal Lobe	Central nervous system	A	YES	DECEASED	2012.00
510	NO	No	C71.0	18323.00	C71.0	YES	NO		100.00		NO	90.00	Left			NO		NO	NO		NO	G2	NO	FALSE	No	TCGA-VM-A8CE.E89B7ED7-D8CA-4112-B0D9-	A8CE	Pre-Adjuvant Therapy	WITH TUMOR	YES	NO		NO	Primary Tumor	1.00	YES	NO		Not listed in Medical Record	YES	NO	YES	VM	Supratentorial, Frontal Lobe	Central nervous system	A	NO	LIVING	2011.00
511	NO	No	C71.0	43891.00	C71.0	YES	NO		90.00		NO	60.00	Left	YES	IHC	YES		NO	NO		YES	G3	NO	FALSE	No	TCGA-VM-A8CF.3D01CE9E-83E3-4D46-A3C2-	A8CF	Pre-Adjuvant Therapy	TUMOR FREE	NO	NO		YES	Primary Tumor	1.00	NO	NO		Not listed in Medical Record	YES	NO	YES	VM	Supratentorial, Frontal Lobe	Central nervous system	A	NO	LIVING	2013.00
512	NO	No	C71.0	36586.00	C71.0	YES	NO		110.00		NO	90.00	Right	YES	IHC	YES		NO	NO		YES	G2	NO	FALSE	No	TCGA-VM-A8CH.A289AB8D-8051-48E9-91FE-	A8CH	Preoperative	TUMOR FREE	YES	NO	Complete Remission/Response	NO	Primary Tumor	1.00	YES	YES		Not listed in Medical Record	NO	NO	YES	VM	Supratentorial, Frontal Lobe	Central nervous system	A	YES	LIVING	2013.00
513	NO	No	C71.0	30011.00	C71.0	YES			140.00		NO		Left			NO		NO	NO	NO	YES	G3	NO	TRUE	No	TCGA-VV-A829.CD914F13-6DA0-47EE-90AD-	A829		TUMOR FREE			Stable Disease	YES	Primary Tumor	1.00	NO	NO		Not listed in Medical Record	YES	NO	YES	VV	Supratentorial, Frontal Lobe	Central nervous system	A	NO	LIVING	2011.00
514	NO	No	C71.0	43891.00	C71.0	YES			270.00		NO		Left					NO	YES	NO	NO	G3	NO	TRUE	No	TCGA-VV-A86M.866E82BA-87CA-408F-A0E5-	A86M		TUMOR FREE				YES	Primary Tumor	1.00	YES	YES		Not listed in Medical Record	YES	NO	YES	VV	Supratentorial, Frontal Lobe	Central nervous system	A	NO	LIVING	2013.00
515	NO	No	C71.0	18688.00	C71.0	YES	NO		260.00		NO		Right			NO			YES	NO	NO	G3	NO	TRUE	No	TCGA-VW-A7QS.47DD727D-85CE-4CBC-8FE6-	A7QS		WITH TUMOR	YES	YES	Stable Disease	NO	Primary Tumor	1.00	NO	NO		Not listed in Medical Record	YES	NO	YES	VW	Supratentorial, Frontal Lobe	Central nervous system	A	NO	LIVING	2011.00
516	NO	No	C71.0	43891.00	C71.0	YES	NO		40.00		NO		Left			NO			YES	NO	NO	G3	NO	TRUE	No	TCGA-VW-A8FI.B073A7FE-1986-4EF7-B186-	A8FI		WITH TUMOR	NO	NO	Stable Disease	YES	Primary Tumor	1.00	NO	NO		White Matter	YES	YES	NO	VW	Supratentorial, Temporal Lobe	Central nervous system	A	NO	DECEASED	2013.00
517	NO	No	C71.0	18323.00	C71.0	YES			530.00		NO		Right			NO		NO	NO	NO	NO	G2	NO	FALSE	No	TCGA-W9-A837.278CE30B-6EAA-47C3-9D21-	A837		WITH TUMOR	YES		Stable Disease	NO	Primary Tumor	1.00	YES	NO		Not listed in Medical Record	NO	NO	YES	W9	Supratentorial, Frontal Lobe	Central nervous system	A	NO	LIVING	2010.00
518	NO	No	C71.0	36586.00	C71.0	YES			30.00		NO		Right	YES	IHC	YES		NO	NO	NO	YES	G2	NO	FALSE	No	TCGA-WH-A86K.BA54C0C4-01A6-4764-ABEC-	A86K	Pre-Adjuvant Therapy	WITH TUMOR			Stable Disease	NO	Primary Tumor	1.00	YES	NO		Not listed in Medical Record	NO	NO	YES	WH	Supratentorial, Frontal Lobe	Central nervous system	A	NO	LIVING	2013.00
519	NO	No	C71.0	43891.00	C71.0	YES	NO					100.00	Left						NO		NO	G3	NO		No		A858	Pre-Adjuvant Therapy	TUMOR FREE	NO	NO	Complete Remission/Response	YES	Primary Tumor	1.00	NO	NO		Not listed in Medical Record	YES	NO	YES	WY	Supratentorial, Frontal Lobe	Central nervous system		NO	LIVING	2010.00
520	NO	No	C71.0	36586.00	C71.0	YES	NO					100.00	Left						NO	NO	NO	G2	NO		No		A859	Pre-Adjuvant Therapy	WITH TUMOR	YES		Stable Disease	YES	Primary Tumor	1.00	YES	NO		Not listed in Medical Record	NO	NO	YES	WY	Supratentorial, Frontal Lobe	Central nervous system		NO	LIVING	2010.00
521	NO	No	C71.0	36586.00	C71.0	YES	NO		500.00		NO		Right			NO			NO	NO	NO	G2	NO	TRUE	No	TCGA-WY-A85A.AEC260E5-995F-439A-8042-	A85A		TUMOR FREE	YES	YES	Partial Remission/Response	NO	Primary Tumor	1.00	YES	NO		Not listed in Medical Record	NO	NO	YES	WY	Supratentorial, Frontal Lobe	Central nervous system	A	NO	LIVING	2010.00
522	NO	No	C71.0	36586.00	C71.0	YES	NO		420.00		NO		Right					NO	NO	NO	NO	G2	NO	TRUE	No	TCGA-WY-A85B.3BB27604-CFD2-4C1D-A53A-	A85B		TUMOR FREE	YES		Complete Remission/Response	NO	Primary Tumor	1.00	YES	YES		Not listed in Medical Record	NO	NO	YES	WY	Supratentorial, Temporal Lobe	Central nervous system	A	NO	LIVING	2010.00
523	NO	No	C71.0	36586.00	C71.0	YES	NO		300.00		NO		Right			NO		NO	NO	NO	YES	G2	YES	TRUE	No	TCGA-WY-A85C.2CB63EDD-F6C4-4CE9-AB8D-	A85C		WITH TUMOR	YES	YES	Progressive Disease	YES	Primary Tumor	1.00	NO	YES		Not listed in Medical Record	YES	NO	YES	WY	Supratentorial, Frontal Lobe	Central nervous system	A	NO	LIVING	2010.00
524	NO	No	C71.0	30011.00	C71.0	YES	NO		120.00		NO		Right			NO			NO	NO	NO	G2	YES	TRUE	No	TCGA-WY-A85D.82FFEB3D-5C1C-4FF0-A824-	A85D		WITH TUMOR	NO	YES	Partial Remission/Response	NO	Primary Tumor	1.00	NO	YES		Not listed in Medical Record	NO	NO	YES	WY	Supratentorial, Frontal Lobe	Central nervous system	A	YES	LIVING	2010.00
525	NO	No	C71.0	30011.00	C71.0	YES	NO		60.00		NO		Left			NO		NO	NO	NO	NO	G2	YES	TRUE	No	TCGA-WY-A85E.BD5E04AC-1E0D-400E-B5A8-	A85E		WITH TUMOR	YES	NO	Partial Remission/Response	NO	Primary Tumor	1.00	YES	NO			NO	NO	YES	WY	Supratentorial, Frontal Lobe	Central nervous system	A	YES	LIVING	2011.00
