# Supplementary material for: High APLN Expression Predicts Poor Prognosis for Glioma Patients
Source: Oxid Med Cell Longev. 2022 Sep 22;2022:8393336. doi: 10.1155/2022/8393336 (PMC9526648; doi:10.1155/2022/8393336)
Supplement: Supplementary Materials — Figure S1. Kaplan-Meier curves of recurrence free survival (RFS) in LGG patients with seizure history (A), primary tumor (B), targeted molecular therapy (C), and oligodendroglioma (D) from TCGA data. Figure S2. Kaplan-Meier curves of overall survival (OS) in LGG patients without seizure history (A), with recurrent tumor (B), with astrocytoma (C), and with oligoastrocytoma (D) from TCGA data. Figure S3. Kaplan-Meier curves of recurrence free survival (RFS) in LGG patients without seizure history (A), with recurrent tumor (B), without targeted molecular therapy (C), with astrocytoma (D), and with oligoastrocytoma (E) from TCGA data. Figure S4. Kaplan-Meier curves of progression free survival (PFS) in LGG patients with biopsy (A), partial resection (B), total resection (C), temozolomide (TMZ, D), and radiation therapy (RT, E) from GSE107850 dataset. Figure S5. Kaplan-Meier curves of PFS in LGG patients with different IDH status (mutated vs. normal) in high APLN expression group (A) and low APLN expression group (B). Kaplan-Meier curves of PFS in LGG patients with different treatment therapies (RT vs. TMZ, B) in high APLN expression group (C) and low APLN expression group (D). The high APLN expression was defined by upper 50% expression, and low APLN expression group was defined by lower 50% expression in GSE107850 dataset. RT, radiation therapy; TMZ, temozolomide. Table S1. Information of LGG clinical samples from TCGA. Table S2. Information of LGG clinical samples from GSE107850. [file 8393336.f1.zip › Table S2.pdf]

Table S2. Information of LGG clinical samples from GSE107850

No.	!Sample_title	!Sample_geo_accession	APLN expression-ILMN_1689353	!Sample_status	!Sample_submission_date	!Sample_last_update_date	!Sample_type	!Sample_channel_count	!Sample_source_name_ch1	!Sample_organism_ch1	!Sample_characteristics_ch1	!Sample_characteristics_ch1	!Sample_characteristics_ch1	!Sample_characteristics_ch1	!Sample_characteristics_ch1	!Sample_characteristics_ch1	!Sample_characteristics_ch1	!Sample_characteristics_ch1	!Sample_characteristics_ch1	!Sample_characteristics_ch1	!Sample_molecule_ch1	!Sample_extract_protocol_ch1	!Sample_label_ch1	!Sample_label_protocol_ch1	!Sample_taxid_ch1	!Sample_hyb_protocol	!Sample_scan_protocol	!Sample_description	!Sample_data_processing	!Sample_platform_id	!Sample_contact_name	!Sample_contact_email	!Sample_contact_phone	!Sample_contact_laboratory	!Sample_contact_department	!Sample_contact_institute	!Sample_contact_address	!Sample_contact_city	!Sample_contact_zip/postal_code	!Sample_contact_country	!Sample_supplementary_file	!Sample_data_row_count	!series_matrix_table_begin	ID_REF
1	LGG_22033_X102	GSM2881630	9.964616893	Public on Mar 19 2019	Dec 08 2017	Mar 19 2019	RNA	1	low grade glioma	Homo sapiens	gender: Female	age: 46.8966	treatment therapy: RT	igs: IGS-9	type.of.sugery: Partial removal	histology: AOD GrII	performance: 0	idh.status: mutated	progression free survival (pfs) event: Yes	pfs (days): 1876	total RNA	Total RNA was extracted from resection material of	Biotin	As per manufacturer (Illumina)	9606	RNA was hybridized to each array according to the	The arrays were scanned using standard Illumina	raw data file and column: DASL.1; X102012.073	The data was quantile normalized using	GPL14951	Pim,,French	p.french@erasmusmc.nl	+31 10 7044333	Neurooncology	Neurology	Erasmus MC	Dr Molewaterplein 50	Rotterdam	2040CA	Netherlands	NONE	29377		GSM2881630
2	LGG_22033_X105	GSM2881631	9.882570652	Public on Mar 19 2019	Dec 08 2017	Mar 19 2019	RNA	1	low grade glioma	Homo sapiens	gender: Male	age: 28.1944	treatment therapy: TMZ	igs: IGS-17	type.of.sugery: Partial removal	histology: AOA GrII	performance: 0	idh.status: mutated	progression free survival (pfs) event: Yes	pfs (days): 1603	total RNA	Total RNA was extracted from resection material of	Biotin	As per manufacturer (Illumina)	9606	RNA was hybridized to each array according to the	The arrays were scanned using standard Illumina	raw data file and column: DASL.2; 102217-96	The data was quantile normalized using	GPL14951	Pim,,French	p.french@erasmusmc.nl	+31 10 7044333	Neurooncology	Neurology	Erasmus MC	Dr Molewaterplein 50	Rotterdam	2040CA	Netherlands	NONE	29377		GSM2881631
3	LGG_22033_X107	GSM2881632	9.98834864	Public on Mar 19 2019	Dec 08 2017	Mar 19 2019	RNA	1	low grade glioma	Homo sapiens	gender: Female	age: 51.2608	treatment therapy: RT	igs: IGS-17	type.of.sugery: Biopsy	histology: AA GrII	performance: 0	idh.status: mutated	progression free survival (pfs) event: No	pfs (days): 2056	total RNA	Total RNA was extracted from resection material of	Biotin	As per manufacturer (Illumina)	9606	RNA was hybridized to each array according to the	The arrays were scanned using standard Illumina	raw data file and column: DASL.1; X102012.151	The data was quantile normalized using	GPL14951	Pim,,French	p.french@erasmusmc.nl	+31 10 7044333	Neurooncology	Neurology	Erasmus MC	Dr Molewaterplein 50	Rotterdam	2040CA	Netherlands	NONE	29377		GSM2881632
4	LGG_22033_X113	GSM2881633	10.43887692	Public on Mar 19 2019	Dec 08 2017	Mar 19 2019	RNA	1	low grade glioma	Homo sapiens	gender: Male	age: 26.5325	treatment therapy: TMZ	igs: IGS-17	type.of.sugery: Partial removal	histology: AA GrII	performance: 0	idh.status: mutated	progression free survival (pfs) event: Yes	pfs (days): 781	total RNA	Total RNA was extracted from resection material of	Biotin	As per manufacturer (Illumina)	9606	RNA was hybridized to each array according to the	The arrays were scanned using standard Illumina	raw data file and column: DASL.2; 102217-33	The data was quantile normalized using	GPL14951	Pim,,French	p.french@erasmusmc.nl	+31 10 7044333	Neurooncology	Neurology	Erasmus MC	Dr Molewaterplein 50	Rotterdam	2040CA	Netherlands	NONE	29377		GSM2881633
5	LGG_22033_X114	GSM2881634	11.07504568	Public on Mar 19 2019	Dec 08 2017	Mar 19 2019	RNA	1	low grade glioma	Homo sapiens	gender: Male	age: 27.7317	treatment therapy: RT	igs: IGS-16	type.of.sugery: Partial removal	histology: AOA GrII	performance: 1	idh.status: normal	progression free survival (pfs) event: No	pfs (days): 566	total RNA	Total RNA was extracted from resection material of	Biotin	As per manufacturer (Illumina)	9606	RNA was hybridized to each array according to the	The arrays were scanned using standard Illumina	raw data file and column: DASL.1; X102012.146	The data was quantile normalized using	GPL14951	Pim,,French	p.french@erasmusmc.nl	+31 10 7044333	Neurooncology	Neurology	Erasmus MC	Dr Molewaterplein 50	Rotterdam	2040CA	Netherlands	NONE	29377		GSM2881634
6	LGG_22033_X116	GSM2881635	10.21653415	Public on Mar 19 2019	Dec 08 2017	Mar 19 2019	RNA	1	low grade glioma	Homo sapiens	gender: Male	age: 22.3025	treatment therapy: RT	igs: IGS-17	type.of.sugery: Total removal	histology: AOA GrII	performance: 1	idh.status: mutated	progression free survival (pfs) event: Yes	pfs (days): 851	total RNA	Total RNA was extracted from resection material of	Biotin	As per manufacturer (Illumina)	9606	RNA was hybridized to each array according to the	The arrays were scanned using standard Illumina	raw data file and column: DASL.5; X102413.07	The data was quantile normalized using	GPL14951	Pim,,French	p.french@erasmusmc.nl	+31 10 7044333	Neurooncology	Neurology	Erasmus MC	Dr Molewaterplein 50	Rotterdam	2040CA	Netherlands	NONE	29377		GSM2881635
7	LGG_22033_X125	GSM2881636	10.16062188	Public on Mar 19 2019	Dec 08 2017	Mar 19 2019	RNA	1	low grade glioma	Homo sapiens	gender: Male	age: 63.4825	treatment therapy: TMZ	igs: IGS-17	type.of.sugery: Total removal	histology: AOA GrII	performance: 0	idh.status: mutated	progression free survival (pfs) event: No	pfs (days): 1935	total RNA	Total RNA was extracted from resection material of	Biotin	As per manufacturer (Illumina)	9606	RNA was hybridized to each array according to the	The arrays were scanned using standard Illumina	raw data file and column: DASL.2; 102217-24	The data was quantile normalized using	GPL14951	Pim,,French	p.french@erasmusmc.nl	+31 10 7044333	Neurooncology	Neurology	Erasmus MC	Dr Molewaterplein 50	Rotterdam	2040CA	Netherlands	NONE	29377		GSM2881636
8	LGG_22033_X127	GSM2881637	10.39520104	Public on Mar 19 2019	Dec 08 2017	Mar 19 2019	RNA	1	low grade glioma	Homo sapiens	gender: Female	age: 52.5722	treatment therapy: RT	igs: IGS-17	type.of.sugery: Biopsy	histology: AA GrII	performance: 0	idh.status: mutated	progression free survival (pfs) event: Yes	pfs (days): 1259	total RNA	Total RNA was extracted from resection material of	Biotin	As per manufacturer (Illumina)	9606	RNA was hybridized to each array according to the	The arrays were scanned using standard Illumina	raw data file and column: DASL.2; 102217-78	The data was quantile normalized using	GPL14951	Pim,,French	p.french@erasmusmc.nl	+31 10 7044333	Neurooncology	Neurology	Erasmus MC	Dr Molewaterplein 50	Rotterdam	2040CA	Netherlands	NONE	29377		GSM2881637
9	LGG_22033_X132	GSM2881638	11.34223198	Public on Mar 19 2019	Dec 08 2017	Mar 19 2019	RNA	1	low grade glioma	Homo sapiens	gender: Female	age: 39.4853	treatment therapy: RT	igs: IGS-17	type.of.sugery: Partial removal	histology: AOA GrII	performance: 1	idh.status: mutated	progression free survival (pfs) event: No	pfs (days): 2043	total RNA	Total RNA was extracted from resection material of	Biotin	As per manufacturer (Illumina)	9606	RNA was hybridized to each array according to the	The arrays were scanned using standard Illumina	raw data file and column: DASL.5; X102413.08	The data was quantile normalized using	GPL14951	Pim,,French	p.french@erasmusmc.nl	+31 10 7044333	Neurooncology	Neurology	Erasmus MC	Dr Molewaterplein 50	Rotterdam	2040CA	Netherlands	NONE	29377		GSM2881638
10	LGG_22033_X136	GSM2881639	11.13699006	Public on Mar 19 2019	Dec 08 2017	Mar 19 2019	RNA	1	low grade glioma	Homo sapiens	gender: Female	age: 20.9555	treatment therapy: RT	igs: IGS-9	type.of.sugery: Partial removal	histology: AOA GrII	performance: 0	idh.status: mutated	progression free survival (pfs) event: No	pfs (days): 1380	total RNA	Total RNA was extracted from resection material of	Biotin	As per manufacturer (Illumina)	9606	RNA was hybridized to each array according to the	The arrays were scanned using standard Illumina	raw data file and column: DASL.1; X102012.069	The data was quantile normalized using	GPL14951	Pim,,French	p.french@erasmusmc.nl	+31 10 7044333	Neurooncology	Neurology	Erasmus MC	Dr Molewaterplein 50	Rotterdam	2040CA	Netherlands	NONE	29377		GSM2881639
11	LGG_22033_X137	GSM2881640	8.686218457	Public on Mar 19 2019	Dec 08 2017	Mar 19 2019	RNA	1	low grade glioma	Homo sapiens	gender: Female	age: 59.2361	treatment therapy: RT	igs: IGS-17	type.of.sugery: Partial removal	histology: AOD GrII	performance: 1	idh.status: mutated	progression free survival (pfs) event: Yes	pfs (days): 575	total RNA	Total RNA was extracted from resection material of	Biotin	As per manufacturer (Illumina)	9606	RNA was hybridized to each array according to the	The arrays were scanned using standard Illumina	raw data file and column: DASL.1; X102012.075	The data was quantile normalized using	GPL14951	Pim,,French	p.french@erasmusmc.nl	+31 10 7044333	Neurooncology	Neurology	Erasmus MC	Dr Molewaterplein 50	Rotterdam	2040CA	Netherlands	NONE	29377		GSM2881640
12	LGG_22033_X138	GSM2881641	10.74734754	Public on Mar 19 2019	Dec 08 2017	Mar 19 2019	RNA	1	low grade glioma	Homo sapiens	gender: Male	age: 63.833	treatment therapy: TMZ	igs: IGS-9	type.of.sugery: Biopsy	histology: AOD GrII	performance: 0	idh.status: mutated	progression free survival (pfs) event: Yes	pfs (days): 350	total RNA	Total RNA was extracted from resection material of	Biotin	As per manufacturer (Illumina)	9606	RNA was hybridized to each array according to the	The arrays were scanned using standard Illumina	raw data file and column: DASL.3; X1022329.002	The data was quantile normalized using	GPL14951	Pim,,French	p.french@erasmusmc.nl	+31 10 7044333	Neurooncology	Neurology	Erasmus MC	Dr Molewaterplein 50	Rotterdam	2040CA	Netherlands	NONE	29377		GSM2881641
13	LGG_22033_X14	GSM2881642	9.365845158	Public on Mar 19 2019	Dec 08 2017	Mar 19 2019	RNA	1	low grade glioma	Homo sapiens	gender: Female	age: 34.371	treatment therapy: RT	igs: IGS-17	type.of.sugery: Partial removal	histology: AA GrII	performance: 1	idh.status: mutated	progression free survival (pfs) event: Yes	pfs (days): 1043	total RNA	Total RNA was extracted from resection material of	Biotin	As per manufacturer (Illumina)	9606	RNA was hybridized to each array according to the	The arrays were scanned using standard Illumina	raw data file and column: DASL.5; X102413.01	The data was quantile normalized using	GPL14951	Pim,,French	p.french@erasmusmc.nl	+31 10 7044333	Neurooncology	Neurology	Erasmus MC	Dr Molewaterplein 50	Rotterdam	2040CA	Netherlands	NONE	29377		GSM2881642
14	LGG_22033_X147	GSM2881643	10.99289837	Public on Mar 19 2019	Dec 08 2017	Mar 19 2019	RNA	1	low grade glioma	Homo sapiens	gender: Male	age: 50.2231	treatment therapy: TMZ	igs: r.X17	type.of.sugery: Partial removal	histology: AOA GrII	performance: 0	idh.status: normal	progression free survival (pfs) event: Yes	pfs (days): 173	total RNA	Total RNA was extracted from resection material of	Biotin	As per manufacturer (Illumina)	9606	RNA was hybridized to each array according to the	The arrays were scanned using standard Illumina	raw data file and column: DASL.1; X102012.066	The data was quantile normalized using	GPL14951	Pim,,French	p.french@erasmusmc.nl	+31 10 7044333	Neurooncology	Neurology	Erasmus MC	Dr Molewaterplein 50	Rotterdam	2040CA	Netherlands	NONE	29377		GSM2881643
15	LGG_22033_X149	GSM2881644	10.73323265	Public on Mar 19 2019	Dec 08 2017	Mar 19 2019	RNA	1	low grade glioma	Homo sapiens	gender: Female	age: 36.4052	treatment therapy: TMZ	igs: IGS-17	type.of.sugery: Partial removal	histology: AA GrII	performance: 0	idh.status: mutated	progression free survival (pfs) event: Yes	pfs (days): 915	total RNA	Total RNA was extracted from resection material of	Biotin	As per manufacturer (Illumina)	9606	RNA was hybridized to each array according to the	The arrays were scanned using standard Illumina	raw data file and column: DASL.1; X102012.123	The data was quantile normalized using	GPL14951	Pim,,French	p.french@erasmusmc.nl	+31 10 7044333	Neurooncology	Neurology	Erasmus MC	Dr Molewaterplein 50	Rotterdam	2040CA	Netherlands	NONE	29377		GSM2881644
16	LGG_22033_X150	GSM2881645	10.1688176	Public on Mar 19 2019	Dec 08 2017	Mar 19 2019	RNA	1	low grade glioma	Homo sapiens	gender: Female	age: 46.3737	treatment therapy: RT	igs: IGS-9	type.of.sugery: Biopsy	histology: AOD GrII	performance: 0	idh.status: mutated	progression free survival (pfs) event: No	pfs (days): 2063	total RNA	Total RNA was extracted from resection material of	Biotin	As per manufacturer (Illumina)	9606	RNA was hybridized to each array according to the	The arrays were scanned using standard Illumina	raw data file and column: DASL.2; 102217-71	The data was quantile normalized using	GPL14951	Pim,,French	p.french@erasmusmc.nl	+31 10 7044333	Neurooncology	Neurology	Erasmus MC	Dr Molewaterplein 50	Rotterdam	2040CA	Netherlands	NONE	29377		GSM2881645
17	LGG_22033_X151	GSM2881646	10.18022335	Public on Mar 19 2019	Dec 08 2017	Mar 19 2019	RNA	1	low grade glioma	Homo sapiens	gender: Female	age: 52.961	treatment therapy: TMZ	igs: r.X9	type.of.sugery: Partial removal	histology: AOD GrII	performance: 1	idh.status: mutated	progression free survival (pfs) event: No	pfs (days): 2015	total RNA	Total RNA was extracted from resection material of	Biotin	As per manufacturer (Illumina)	9606	RNA was hybridized to each array according to the	The arrays were scanned using standard Illumina	raw data file and column: DASL.5; X102413.09	The data was quantile normalized using	GPL14951	Pim,,French	p.french@erasmusmc.nl	+31 10 7044333	Neurooncology	Neurology	Erasmus MC	Dr Molewaterplein 50	Rotterdam	2040CA	Netherlands	NONE	29377		GSM2881646
18	LGG_22033_X152	GSM2881647	10.73504502	Public on Mar 19 2019	Dec 08 2017	Mar 19 2019	RNA	1	low grade glioma	Homo sapiens	gender: Male	age: 31.989	treatment therapy: RT	igs: IGS-17	type.of.sugery: Partial removal	histology: AA GrII	performance: 0	idh.status: mutated	progression free survival (pfs) event: No	pfs (days): 1988	total RNA	Total RNA was extracted from resection material of	Biotin	As per manufacturer (Illumina)	9606	RNA was hybridized to each array according to the	The arrays were scanned using standard Illumina	raw data file and column: DASL.2; 102217-56	The data was quantile normalized using	GPL14951	Pim,,French	p.french@erasmusmc.nl	+31 10 7044333	Neurooncology	Neurology	Erasmus MC	Dr Molewaterplein 50	Rotterdam	2040CA	Netherlands	NONE	29377		GSM2881647
19	LGG_22033_X156	GSM2881648	8.611345743	Public on Mar 19 2019	Dec 08 2017	Mar 19 2019	RNA	1	low grade glioma	Homo sapiens	gender: Male	age: 49.4018	treatment therapy: TMZ	igs: IGS-9	type.of.sugery: Biopsy	histology: AA GrII	performance: 0	idh.status: normal	progression free survival (pfs) event: No	pfs (days): 2051	total RNA	Total RNA was extracted from resection material of	Biotin	As per manufacturer (Illumina)	9606	RNA was hybridized to each array according to the	The arrays were scanned using standard Illumina	raw data file and column: DASL.1; X102012.152	The data was quantile normalized using	GPL14951	Pim,,French	p.french@erasmusmc.nl	+31 10 7044333	Neurooncology	Neurology	Erasmus MC	Dr Molewaterplein 50	Rotterdam	2040CA	Netherlands	NONE	29377		GSM2881648
20	LGG_22033_X161	GSM2881649	10.32996534	Public on Mar 19 2019	Dec 08 2017	Mar 19 2019	RNA	1	low grade glioma	Homo sapiens	gender: Female	age: 38.1629	treatment therapy: TMZ	igs: IGS-17	type.of.sugery: Total removal	histology: AOD GrII	performance: 1	idh.status: mutated	progression free survival (pfs) event: No	pfs (days): 1895	total RNA	Total RNA was extracted from resection material of	Biotin	As per manufacturer (Illumina)	9606	RNA was hybridized to each array according to the	The arrays were scanned using standard Illumina	raw data file and column: DASL.2; 102217-15	The data was quantile normalized using	GPL14951	Pim,,French	p.french@erasmusmc.nl	+31 10 7044333	Neurooncology	Neurology	Erasmus MC	Dr Molewaterplein 50	Rotterdam	2040CA	Netherlands	NONE	29377		GSM2881649
21	LGG_22033_X167	GSM2881650	10.56223436	Public on Mar 19 2019	Dec 08 2017	Mar 19 2019	RNA	1	low grade glioma	Homo sapiens	gender: Female	age: 53.2539	treatment therapy: TMZ	igs: IGS-9	type.of.sugery: Partial removal	histology: AOD GrII	performance: 0	idh.status: mutated	progression free survival (pfs) event: No	pfs (days): 1976	total RNA	Total RNA was extracted from resection material of	Biotin	As per manufacturer (Illumina)	9606	RNA was hybridized to each array according to the	The arrays were scanned using standard Illumina	raw data file and column: DASL.1; X102012.067	The data was quantile normalized using	GPL14951	Pim,,French	p.french@erasmusmc.nl	+31 10 7044333	Neurooncology	Neurology	Erasmus MC	Dr Molewaterplein 50	Rotterdam	2040CA	Netherlands	NONE	29377		GSM2881650
22	LGG_22033_X173	GSM2881651	9.979037181	Public on Mar 19 2019	Dec 08 2017	Mar 19 2019	RNA	1	low grade glioma	Homo sapiens	gender: Male	age: 54.2834	treatment therapy: TMZ	igs: IGS-17	type.of.sugery: Partial removal	histology: AA GrII	performance: 0	idh.status: mutated	progression free survival (pfs) event: No	pfs (days): 1928	total RNA	Total RNA was extracted from resection material of	Biotin	As per manufacturer (Illumina)	9606	RNA was hybridized to each array according to the	The arrays were scanned using standard Illumina	raw data file and column: DASL.1; X102012.068	The data was quantile normalized using	GPL14951	Pim,,French	p.french@erasmusmc.nl	+31 10 7044333	Neurooncology	Neurology	Erasmus MC	Dr Molewaterplein 50	Rotterdam	2040CA	Netherlands	NONE	29377		GSM2881651
23	LGG_22033_X178	GSM2881652	10.03398186	Public on Mar 19 2019	Dec 08 2017	Mar 19 2019	RNA	1	low grade glioma	Homo sapiens	gender: Female	age: 43.2471	treatment therapy: RT	igs: r.X17	type.of.sugery: Partial removal	histology: AOD GrII	performance: 0	idh.status: mutated	progression free survival (pfs) event: Yes	pfs (days): 1898	total RNA	Total RNA was extracted from resection material of	Biotin	As per manufacturer (Illumina)	9606	RNA was hybridized to each array according to the	The arrays were scanned using standard Illumina	raw data file and column: DASL.4; X102380.06	The data was quantile normalized using	GPL14951	Pim,,French	p.french@erasmusmc.nl	+31 10 7044333	Neurooncology	Neurology	Erasmus MC	Dr Molewaterplein 50	Rotterdam	2040CA	Netherlands	NONE	29377		GSM2881652
24	LGG_22033_X18	GSM2881653	11.74623706	Public on Mar 19 2019	Dec 08 2017	Mar 19 2019	RNA	1	low grade glioma	Homo sapiens	gender: Male	age: 63.7125	treatment therapy: RT	igs: IGS-9	type.of.sugery: Total removal	histology: AOA GrII	performance: 0	idh.status: mutated	progression free survival (pfs) event: No	pfs (days): 2450	total RNA	Total RNA was extracted from resection material of	Biotin	As per manufacturer (Illumina)	9606	RNA was hybridized to each array according to the	The arrays were scanned using standard Illumina	raw data file and column: DASL.4; X102380.01	The data was quantile normalized using	GPL14951	Pim,,French	p.french@erasmusmc.nl	+31 10 7044333	Neurooncology	Neurology	Erasmus MC	Dr Molewaterplein 50	Rotterdam	2040CA	Netherlands	NONE	29377		GSM2881653
25	LGG_22033_X181	GSM2881654	10.26545371	Public on Mar 19 2019	Dec 08 2017	Mar 19 2019	RNA	1	low grade glioma	Homo sapiens	gender: Male	age: 37.306	treatment therapy: RT	igs: IGS-9	type.of.sugery: Partial removal	histology: AOA GrII	performance: 0	idh.status: mutated	progression free survival (pfs) event: Yes	pfs (days): 556	total RNA	Total RNA was extracted from resection material of	Biotin	As per manufacturer (Illumina)	9606	RNA was hybridized to each array according to the	The arrays were scanned using standard Illumina	raw data file and column: DASL.1; X102012.063	The data was quantile normalized using	GPL14951	Pim,,French	p.french@erasmusmc.nl	+31 10 7044333	Neurooncology	Neurology	Erasmus MC	Dr Molewaterplein 50	Rotterdam	2040CA	Netherlands	NONE	29377		GSM2881654
26	LGG_22033_X188	GSM2881655	10.02621469	Public on Mar 19 2019	Dec 08 2017	Mar 19 2019	RNA	1	low grade glioma	Homo sapiens	gender: Male	age: 32.8706	treatment therapy: TMZ	igs: IGS-17	type.of.sugery: Total removal	histology: AA GrII	performance: 1	idh.status: mutated	progression free survival (pfs) event: No	pfs (days): 388	total RNA	Total RNA was extracted from resection material of	Biotin	As per manufacturer (Illumina)	9606	RNA was hybridized to each array according to the	The arrays were scanned using standard Illumina	raw data file and column: DASL.4; X102380.07	The data was quantile normalized using	GPL14951	Pim,,French	p.french@erasmusmc.nl	+31 10 7044333	Neurooncology	Neurology	Erasmus MC	Dr Molewaterplein 50	Rotterdam	2040CA	Netherlands	NONE	29377		GSM2881655
27	LGG_22033_X193	GSM2881656	10.30329528	Public on Mar 19 2019	Dec 08 2017	Mar 19 2019	RNA	1	low grade glioma	Homo sapiens	gender: Male	age: 58.3217	treatment therapy: TMZ	igs: IGS-9	type.of.sugery: Partial removal	histology: AOD GrII	performance: 0	idh.status: mutated	progression free survival (pfs) event: Yes	pfs (days): 719	total RNA	Total RNA was extracted from resection material of	Biotin	As per manufacturer (Illumina)	9606	RNA was hybridized to each array according to the	The arrays were scanned using standard Illumina	raw data file and column: DASL.2; 102217-73	The data was quantile normalized using	GPL14951	Pim,,French	p.french@erasmusmc.nl	+31 10 7044333	Neurooncology	Neurology	Erasmus MC	Dr Molewaterplein 50	Rotterdam	2040CA	Netherlands	NONE	29377		GSM2881656
28	LGG_22033_X194	GSM2881657	9.38623067	Public on Mar 19 2019	Dec 08 2017	Mar 19 2019	RNA	1	low grade glioma	Homo sapiens	gender: Female	age: 41.8289	treatment therapy: TMZ	igs: IGS-17	type.of.sugery: Partial removal	histology: AOA GrII	performance: 1	idh.status: mutated	progression free survival (pfs) event: Yes	pfs (days): 441	total RNA	Total RNA was extracted from resection material of	Biotin	As per manufacturer (Illumina)	9606	RNA was hybridized to each array according to the	The arrays were scanned using standard Illumina	raw data file and column: DASL.5; X102413.10	The data was quantile normalized using	GPL14951	Pim,,French	p.french@erasmusmc.nl	+31 10 7044333	Neurooncology	Neurology	Erasmus MC	Dr Molewaterplein 50	Rotterdam	2040CA	Netherlands	NONE	29377		GSM2881657
29	LGG_22033_X196	GSM2881658	11.40849633	Public on Mar 19 2019	Dec 08 2017	Mar 19 2019	RNA	1	low grade glioma	Homo sapiens	gender: Female	age: 37.9493	treatment therapy: RT	igs: IGS-17	type.of.sugery: Biopsy	histology: AA GrII	performance: 2	idh.status: mutated	progression free survival (pfs) event: Yes	pfs (days): 293	total RNA	Total RNA was extracted from resection material of	Biotin	As per manufacturer (Illumina)	9606	RNA was hybridized to each array according to the	The arrays were scanned using standard Illumina	raw data file and column: DASL.5; X102413.11	The data was quantile normalized using	GPL14951	Pim,,French	p.french@erasmusmc.nl	+31 10 7044333	Neurooncology	Neurology	Erasmus MC	Dr Molewaterplein 50	Rotterdam	2040CA	Netherlands	NONE	29377		GSM2881658
30	LGG_22033_X200	GSM2881659	11.15104367	Public on Mar 19 2019	Dec 08 2017	Mar 19 2019	RNA	1	low grade glioma	Homo sapiens	gender: Male	age: 42.6229	treatment therapy: TMZ	igs: IGS-17	type.of.sugery: Partial removal	histology: AA GrII	performance: 1	idh.status: mutated	progression free survival (pfs) event: Yes	pfs (days): 1470	total RNA	Total RNA was extracted from resection material of	Biotin	As per manufacturer (Illumina)	9606	RNA was hybridized to each array according to the	The arrays were scanned using standard Illumina	raw data file and column: DASL.3; X1022329.027	The data was quantile normalized using	GPL14951	Pim,,French	p.french@erasmusmc.nl	+31 10 7044333	Neurooncology	Neurology	Erasmus MC	Dr Molewaterplein 50	Rotterdam	2040CA	Netherlands	NONE	29377		GSM2881659
31	LGG_22033_X203	GSM2881660	10.36047488	Public on Mar 19 2019	Dec 08 2017	Mar 19 2019	RNA	1	low grade glioma	Homo sapiens	gender: Male	age: 52.8241	treatment therapy: RT	igs: IGS-17	type.of.sugery: Partial removal	histology: AOD GrII	performance: 0	idh.status: mutated	progression free survival (pfs) event: No	pfs (days): 1940	total RNA	Total RNA was extracted from resection material of	Biotin	As per manufacturer (Illumina)	9606	RNA was hybridized to each array according to the	The arrays were scanned using standard Illumina	raw data file and column: DASL.1; X102012.060	The data was quantile normalized using	GPL14951	Pim,,French	p.french@erasmusmc.nl	+31 10 7044333	Neurooncology	Neurology	Erasmus MC	Dr Molewaterplein 50	Rotterdam	2040CA	Netherlands	NONE	29377		GSM2881660
32	LGG_22033_X207	GSM2881661	11.34732688	Public on Mar 19 2019	Dec 08 2017	Mar 19 2019	RNA	1	low grade glioma	Homo sapiens	gender: Male	age: 71.2279	treatment therapy: RT	igs: IGS-17	type.of.sugery: Biopsy	histology: AOA GrII	performance: 1	idh.status: normal	progression free survival (pfs) event: Yes	pfs (days): 383	total RNA	Total RNA was extracted from resection material of	Biotin	As per manufacturer (Illumina)	9606	RNA was hybridized to each array according to the	The arrays were scanned using standard Illumina	raw data file and column: DASL.5; X102413.12	The data was quantile normalized using	GPL14951	Pim,,French	p.french@erasmusmc.nl	+31 10 7044333	Neurooncology	Neurology	Erasmus MC	Dr Molewaterplein 50	Rotterdam	2040CA	Netherlands	NONE	29377		GSM2881661

33	LGG_22033_X 208	GSM2881662	10.68970098	Public on Mar 19 2019	Dec 08 2017	Mar 19 2019	RNA	1	low grade glioma	Homo sapiens	gender: Female	age: 33.7002	treatment therapy: TMZ	igs: r.X9	type.of.sugery: Partial removal	histology: AOA GrII	performance: 0	idh.status: mutated	progression free survival (pfs) event: Yes	pfs (days): 1096	total RNA	Total RNA was extracted from resection material of	Biotin	As per manufacturer (Illumina)	9606	RNA was hybridized to each array according to the	The arrays were scanned using standard Illumina	raw data file and column: DASL.1; X102012.082	The data was quantile normalized using	GPL14951	Pim,,French	p.french@erasmusmc.nl	+31 10 70443 33	Neurooncology	Neurology	Erasmus MC	Dr Molewaterplein 50	Rotterdam	2040CA	Netherlands	NONE	29377		GSM2881662
34	LGG_22033_X 221	GSM2881663	11.33203542	Public on Mar 19 2019	Dec 08 2017	Mar 19 2019	RNA	1	low grade glioma	Homo sapiens	gender: Male	age: 34.6119	treatment therapy: TMZ	igs: IGS-17	type.of.sugery: Partial removal	histology: AOD GrII	performance: 1	idh.status: mutated	progression free survival (pfs) event: No	pfs (days): 1821	total RNA	Total RNA was extracted from resection material of	Biotin	As per manufacturer (Illumina)	9606	RNA was hybridized to each array according to the	The arrays were scanned using standard Illumina	raw data file and column: DASL.5; X102413.13	The data was quantile normalized using	GPL14951	Pim,,French	p.french@erasmusmc.nl	+31 10 70443 33	Neurooncology	Neurology	Erasmus MC	Dr Molewaterplein 50	Rotterdam	2040CA	Netherlands	NONE	29377		GSM2881663
35	LGG_22033_X 227	GSM2881664	10.15672694	Public on Mar 19 2019	Dec 08 2017	Mar 19 2019	RNA	1	low grade glioma	Homo sapiens	gender: Female	age: 47.7016	treatment therapy: TMZ	igs: r.X9	type.of.sugery: Total removal	histology: AOD GrII	performance: 0	idh.status: mutated	progression free survival (pfs) event: Yes	pfs (days): 371	total RNA	Total RNA was extracted from resection material of	Biotin	As per manufacturer (Illumina)	9606	RNA was hybridized to each array according to the	The arrays were scanned using standard Illumina	raw data file and column: DASL.2; 102217-54	The data was quantile normalized using	GPL14951	Pim,,French	p.french@erasmusmc.nl	+31 10 70443 33	Neurooncology	Neurology	Erasmus MC	Dr Molewaterplein 50	Rotterdam	2040CA	Netherlands	NONE	29377		GSM2881664
36	LGG_22033_X 232	GSM2881665	9.40623953	Public on Mar 19 2019	Dec 08 2017	Mar 19 2019	RNA	1	low grade glioma	Homo sapiens	gender: Female	age: 63.1951	treatment therapy: RT	igs: IGS-9	type.of.sugery: Partial removal	histology: AOD GrII	performance: 0	idh.status: mutated	progression free survival (pfs) event: No	pfs (days): 1798	total RNA	Total RNA was extracted from resection material of	Biotin	As per manufacturer (Illumina)	9606	RNA was hybridized to each array according to the	The arrays were scanned using standard Illumina	raw data file and column: DASL.1; X102012.062	The data was quantile normalized using	GPL14951	Pim,,French	p.french@erasmusmc.nl	+31 10 70443 33	Neurooncology	Neurology	Erasmus MC	Dr Molewaterplein 50	Rotterdam	2040CA	Netherlands	NONE	29377		GSM2881665
37	LGG_22033_X 235	GSM2881666	10.25667947	Public on Mar 19 2019	Dec 08 2017	Mar 19 2019	RNA	1	low grade glioma	Homo sapiens	gender: Male	age: 27.1923	treatment therapy: RT	igs: r.X9	type.of.sugery: Total removal	histology: AA GrII	performance: 0	idh.status: mutated	progression free survival (pfs) event: Yes	pfs (days): 1589	total RNA	Total RNA was extracted from resection material of	Biotin	As per manufacturer (Illumina)	9606	RNA was hybridized to each array according to the	The arrays were scanned using standard Illumina	raw data file and column: DASL.4; X102380.08	The data was quantile normalized using	GPL14951	Pim,,French	p.french@erasmusmc.nl	+31 10 70443 33	Neurooncology	Neurology	Erasmus MC	Dr Molewaterplein 50	Rotterdam	2040CA	Netherlands	NONE	29377		GSM2881666
38	LGG_22033_X 241	GSM2881667	10.74478574	Public on Mar 19 2019	Dec 08 2017	Mar 19 2019	RNA	1	low grade glioma	Homo sapiens	gender: Male	age: 62.9213	treatment therapy: TMZ	igs: IGS-17	type.of.sugery: Biopsy	histology: AOA GrII	performance: 1	idh.status: mutated	progression free survival (pfs) event: No	pfs (days): 1826	total RNA	Total RNA was extracted from resection material of	Biotin	As per manufacturer (Illumina)	9606	RNA was hybridized to each array according to the	The arrays were scanned using standard Illumina	raw data file and column: DASL.5; X102413.14	The data was quantile normalized using	GPL14951	Pim,,French	p.french@erasmusmc.nl	+31 10 70443 33	Neurooncology	Neurology	Erasmus MC	Dr Molewaterplein 50	Rotterdam	2040CA	Netherlands	NONE	29377		GSM2881667
39	LGG_22033_X 243	GSM2881668	9.769581664	Public on Mar 19 2019	Dec 08 2017	Mar 19 2019	RNA	1	low grade glioma	Homo sapiens	gender: Male	age: 36.2628	treatment therapy: RT	igs: r.X17	type.of.sugery: Biopsy	histology: AA GrII	performance: 0	idh.status: mutated	progression free survival (pfs) event: Yes	pfs (days): 1792	total RNA	Total RNA was extracted from resection material of	Biotin	As per manufacturer (Illumina)	9606	RNA was hybridized to each array according to the	The arrays were scanned using standard Illumina	raw data file and column: DASL.5; X102413.15	The data was quantile normalized using	GPL14951	Pim,,French	p.french@erasmusmc.nl	+31 10 70443 33	Neurooncology	Neurology	Erasmus MC	Dr Molewaterplein 50	Rotterdam	2040CA	Netherlands	NONE	29377		GSM2881668
40	LGG_22033_X 244	GSM2881669	10.990634	Public on Mar 19 2019	Dec 08 2017	Mar 19 2019	RNA	1	low grade glioma	Homo sapiens	gender: Male	age: 39.217	treatment therapy: TMZ	igs: r.X17	type.of.sugery: Partial removal	histology: AOA GrII	performance: 0	idh.status: mutated	progression free survival (pfs) event: No	pfs (days): 357	total RNA	Total RNA was extracted from resection material of	Biotin	As per manufacturer (Illumina)	9606	RNA was hybridized to each array according to the	The arrays were scanned using standard Illumina	raw data file and column: DASL.4; X102380.09	The data was quantile normalized using	GPL14951	Pim,,French	p.french@erasmusmc.nl	+31 10 70443 33	Neurooncology	Neurology	Erasmus MC	Dr Molewaterplein 50	Rotterdam	2040CA	Netherlands	NONE	29377		GSM2881669
41	LGG_22033_X 245	GSM2881670	8.825081229	Public on Mar 19 2019	Dec 08 2017	Mar 19 2019	RNA	1	low grade glioma	Homo sapiens	gender: Female	age: 35.5154	treatment therapy: TMZ	igs: IGS-9	type.of.sugery: Biopsy	histology: AOD GrII	performance: 0	idh.status: mutated	progression free survival (pfs) event: Yes	pfs (days): 1675	total RNA	Total RNA was extracted from resection material of	Biotin	As per manufacturer (Illumina)	9606	RNA was hybridized to each array according to the	The arrays were scanned using standard Illumina	raw data file and column: DASL.3; X1022329.019	The data was quantile normalized using	GPL14951	Pim,,French	p.french@erasmusmc.nl	+31 10 70443 33	Neurooncology	Neurology	Erasmus MC	Dr Molewaterplein 50	Rotterdam	2040CA	Netherlands	NONE	29377		GSM2881670
42	LGG_22033_X 246	GSM2881671	9.372095353	Public on Mar 19 2019	Dec 08 2017	Mar 19 2019	RNA	1	low grade glioma	Homo sapiens	gender: Female	age: 49.2402	treatment therapy: RT	igs: IGS-9	type.of.sugery: Total removal	histology: AOD GrII	performance: 1	idh.status: mutated	progression free survival (pfs) event: No	pfs (days): 1794	total RNA	Total RNA was extracted from resection material of	Biotin	As per manufacturer (Illumina)	9606	RNA was hybridized to each array according to the	The arrays were scanned using standard Illumina	raw data file and column: DASL.2; 102217-10	The data was quantile normalized using	GPL14951	Pim,,French	p.french@erasmusmc.nl	+31 10 70443 33	Neurooncology	Neurology	Erasmus MC	Dr Molewaterplein 50	Rotterdam	2040CA	Netherlands	NONE	29377		GSM2881671
43	LGG_22033_X 25	GSM2881672	9.510456421	Public on Mar 19 2019	Dec 08 2017	Mar 19 2019	RNA	1	low grade glioma	Homo sapiens	gender: Male	age: 37.4209	treatment therapy: RT	igs: IGS-9	type.of.sugery: Partial removal	histology: AOD GrII	performance: 0	idh.status: mutated	progression free survival (pfs) event: No	pfs (days): 707	total RNA	Total RNA was extracted from resection material of	Biotin	As per manufacturer (Illumina)	9606	RNA was hybridized to each array according to the	The arrays were scanned using standard Illumina	raw data file and column: DASL.1; X102012.085	The data was quantile normalized using	GPL14951	Pim,,French	p.french@erasmusmc.nl	+31 10 70443 33	Neurooncology	Neurology	Erasmus MC	Dr Molewaterplein 50	Rotterdam	2040CA	Netherlands	NONE	29377		GSM2881672
44	LGG_22033_X 253	GSM2881673	9.665613542	Public on Mar 19 2019	Dec 08 2017	Mar 19 2019	RNA	1	low grade glioma	Homo sapiens	gender: Female	age: 62.6393	treatment therapy: RT	igs: IGS-9	type.of.sugery: Total removal	histology: AOD GrII	performance: 0	idh.status: mutated	progression free survival (pfs) event: No	pfs (days): 1710	total RNA	Total RNA was extracted from resection material of	Biotin	As per manufacturer (Illumina)	9606	RNA was hybridized to each array according to the	The arrays were scanned using standard Illumina	raw data file and column: DASL.1; X102012.059	The data was quantile normalized using	GPL14951	Pim,,French	p.french@erasmusmc.nl	+31 10 70443 33	Neurooncology	Neurology	Erasmus MC	Dr Molewaterplein 50	Rotterdam	2040CA	Netherlands	NONE	29377		GSM2881673
45	LGG_22033_X 254	GSM2881674	10.55388311	Public on Mar 19 2019	Dec 08 2017	Mar 19 2019	RNA	1	low grade glioma	Homo sapiens	gender: Male	age: 57.1088	treatment therapy: TMZ	igs: IGS-9	type.of.sugery: Partial removal	histology: AOD GrII	performance: 0	idh.status: mutated	progression free survival (pfs) event: No	pfs (days): 1698	total RNA	Total RNA was extracted from resection material of	Biotin	As per manufacturer (Illumina)	9606	RNA was hybridized to each array according to the	The arrays were scanned using standard Illumina	raw data file and column: DASL.1; X102012.054	The data was quantile normalized using	GPL14951	Pim,,French	p.french@erasmusmc.nl	+31 10 70443 33	Neurooncology	Neurology	Erasmus MC	Dr Molewaterplein 50	Rotterdam	2040CA	Netherlands	NONE	29377		GSM2881674
46	LGG_22033_X 257	GSM2881675	10.54313293	Public on Mar 19 2019	Dec 08 2017	Mar 19 2019	RNA	1	low grade glioma	Homo sapiens	gender: Female	age: 25.7906	treatment therapy: RT	igs: IGS-17	type.of.sugery: Total removal	histology: AA GrII	performance: 2	idh.status: mutated	progression free survival (pfs) event: Yes	pfs (days): 454	total RNA	Total RNA was extracted from resection material of	Biotin	As per manufacturer (Illumina)	9606	RNA was hybridized to each array according to the	The arrays were scanned using standard Illumina	raw data file and column: DASL.1; X102012.053	The data was quantile normalized using	GPL14951	Pim,,French	p.french@erasmusmc.nl	+31 10 70443 33	Neurooncology	Neurology	Erasmus MC	Dr Molewaterplein 50	Rotterdam	2040CA	Netherlands	NONE	29377		GSM2881675
47	LGG_22033_X 26	GSM2881676	9.339000413	Public on Mar 19 2019	Dec 08 2017	Mar 19 2019	RNA	1	low grade glioma	Homo sapiens	gender: Male	age: 52.961	treatment therapy: TMZ	igs: IGS-17	type.of.sugery: Partial removal	histology: AOD GrII	performance: 1	idh.status: mutated	progression free survival (pfs) event: No	pfs (days): 2387	total RNA	Total RNA was extracted from resection material of	Biotin	As per manufacturer (Illumina)	9606	RNA was hybridized to each array according to the	The arrays were scanned using standard Illumina	raw data file and column: DASL.3; X1022329.014	The data was quantile normalized using	GPL14951	Pim,,French	p.french@erasmusmc.nl	+31 10 70443 33	Neurooncology	Neurology	Erasmus MC	Dr Molewaterplein 50	Rotterdam	2040CA	Netherlands	NONE	29377		GSM2881676
48	LGG_22033_X 262	GSM2881677	10.16471238	Public on Mar 19 2019	Dec 08 2017	Mar 19 2019	RNA	1	low grade glioma	Homo sapiens	gender: Male	age: 28.2491	treatment therapy: RT	igs: IGS-17	type.of.sugery: Total removal	histology: AOD GrII	performance: 1	idh.status: mutated	progression free survival (pfs) event: No	pfs (days): 1093	total RNA	Total RNA was extracted from resection material of	Biotin	As per manufacturer (Illumina)	9606	RNA was hybridized to each array according to the	The arrays were scanned using standard Illumina	raw data file and column: DASL.4; X102380.10	The data was quantile normalized using	GPL14951	Pim,,French	p.french@erasmusmc.nl	+31 10 70443 33	Neurooncology	Neurology	Erasmus MC	Dr Molewaterplein 50	Rotterdam	2040CA	Netherlands	NONE	29377		GSM2881677
49	LGG_22033_X 267	GSM2881678	9.809244186	Public on Mar 19 2019	Dec 08 2017	Mar 19 2019	RNA	1	low grade glioma	Homo sapiens	gender: Female	age: 31.8658	treatment therapy: RT	igs: IGS-17	type.of.sugery: Partial removal	histology: AA GrII	performance: 1	idh.status: mutated	progression free survival (pfs) event: No	pfs (days): 1133	total RNA	Total RNA was extracted from resection material of	Biotin	As per manufacturer (Illumina)	9606	RNA was hybridized to each array according to the	The arrays were scanned using standard Illumina	raw data file and column: DASL.2; 102217-53	The data was quantile normalized using	GPL14951	Pim,,French	p.french@erasmusmc.nl	+31 10 70443 33	Neurooncology	Neurology	Erasmus MC	Dr Molewaterplein 50	Rotterdam	2040CA	Netherlands	NONE	29377		GSM2881678
50	LGG_22033_X 268	GSM2881679	10.18339656	Public on Mar 19 2019	Dec 08 2017	Mar 19 2019	RNA	1	low grade glioma	Homo sapiens	gender: Male	age: 39.2745	treatment therapy: TMZ	igs: IGS-17	type.of.sugery: Partial removal	histology: AOA GrII	performance: 0	idh.status: mutated	progression free survival (pfs) event: Yes	pfs (days): 156	total RNA	Total RNA was extracted from resection material of	Biotin	As per manufacturer (Illumina)	9606	RNA was hybridized to each array according to the	The arrays were scanned using standard Illumina	raw data file and column: DASL.1; X102012.083	The data was quantile normalized using	GPL14951	Pim,,French	p.french@erasmusmc.nl	+31 10 70443 33	Neurooncology	Neurology	Erasmus MC	Dr Molewaterplein 50	Rotterdam	2040CA	Netherlands	NONE	29377		GSM2881679
51	LGG_22033_X 269	GSM2881680	9.642294407	Public on Mar 19 2019	Dec 08 2017	Mar 19 2019	RNA	1	low grade glioma	Homo sapiens	gender: Female	age: 28.6626	treatment therapy: TMZ	igs: IGS-17	type.of.sugery: Total removal	histology: AOD GrII	performance: 0	idh.status: mutated	progression free survival (pfs) event: Yes	pfs (days): 1148	total RNA	Total RNA was extracted from resection material of	Biotin	As per manufacturer (Illumina)	9606	RNA was hybridized to each array according to the	The arrays were scanned using standard Illumina	raw data file and column: DASL.3; X1022329.026	The data was quantile normalized using	GPL14951	Pim,,French	p.french@erasmusmc.nl	+31 10 70443 33	Neurooncology	Neurology	Erasmus MC	Dr Molewaterplein 50	Rotterdam	2040CA	Netherlands	NONE	29377		GSM2881680
52	LGG_22033_X 270	GSM2881681	11.08660247	Public on Mar 19 2019	Dec 08 2017	Mar 19 2019	RNA	1	low grade glioma	Homo sapiens	gender: Male	age: 26.9678	treatment therapy: TMZ	igs: IGS-17	type.of.sugery: Total removal	histology: AA GrII	performance: 0	idh.status: mutated	progression free survival (pfs) event: Yes	pfs (days): 263	total RNA	Total RNA was extracted from resection material of	Biotin	As per manufacturer (Illumina)	9606	RNA was hybridized to each array according to the	The arrays were scanned using standard Illumina	raw data file and column: DASL.2; 102217-97	The data was quantile normalized using	GPL14951	Pim,,French	p.french@erasmusmc.nl	+31 10 70443 33	Neurooncology	Neurology	Erasmus MC	Dr Molewaterplein 50	Rotterdam	2040CA	Netherlands	NONE	29377		GSM2881681
53	LGG_22033_X 271	GSM2881682	10.13415245	Public on Mar 19 2019	Dec 08 2017	Mar 19 2019	RNA	1	low grade glioma	Homo sapiens	gender: Female	age: 44.6927	treatment therapy: TMZ	igs: IGS-9	type.of.sugery: Partial removal	histology: AOD GrII	performance: 0	idh.status: mutated	progression free survival (pfs) event: Yes	pfs (days): 720	total RNA	Total RNA was extracted from resection material of	Biotin	As per manufacturer (Illumina)	9606	RNA was hybridized to each array according to the	The arrays were scanned using standard Illumina	raw data file and column: DASL.2; 102217-63	The data was quantile normalized using	GPL14951	Pim,,French	p.french@erasmusmc.nl	+31 10 70443 33	Neurooncology	Neurology	Erasmus MC	Dr Molewaterplein 50	Rotterdam	2040CA	Netherlands	NONE	29377		GSM2881682
54	LGG_22033_X 275	GSM2881683	10.79134519	Public on Mar 19 2019	Dec 08 2017	Mar 19 2019	RNA	1	low grade glioma	Homo sapiens	gender: Male	age: 36.8679	treatment therapy: TMZ	igs: IGS-17	type.of.sugery: Partial removal	histology: AOA GrII	performance: 0	idh.status: mutated	progression free survival (pfs) event: Yes	pfs (days): 1742	total RNA	Total RNA was extracted from resection material of	Biotin	As per manufacturer (Illumina)	9606	RNA was hybridized to each array according to the	The arrays were scanned using standard Illumina	raw data file and column: DASL.1; X102012.091	The data was quantile normalized using	GPL14951	Pim,,French	p.french@erasmusmc.nl	+31 10 70443 33	Neurooncology	Neurology	Erasmus MC	Dr Molewaterplein 50	Rotterdam	2040CA	Netherlands	NONE	29377		GSM2881683
55	LGG_22033_X 276	GSM2881684	11.03815844	Public on Mar 19 2019	Dec 08 2017	Mar 19 2019	RNA	1	low grade glioma	Homo sapiens	gender: Male	age: 37.6263	treatment therapy: RT	igs: IGS-17	type.of.sugery: Partial removal	histology: AOD GrII	performance: 0	idh.status: mutated	progression free survival (pfs) event: No	pfs (days): 1682	total RNA	Total RNA was extracted from resection material of	Biotin	As per manufacturer (Illumina)	9606	RNA was hybridized to each array according to the	The arrays were scanned using standard Illumina	raw data file and column: DASL.1; X102012.090	The data was quantile normalized using	GPL14951	Pim,,French	p.french@erasmusmc.nl	+31 10 70443 33	Neurooncology	Neurology	Erasmus MC	Dr Molewaterplein 50	Rotterdam	2040CA	Netherlands	NONE	29377		GSM2881684
56	LGG_22033_X 277	GSM2881685	10.1200951	Public on Mar 19 2019	Dec 08 2017	Mar 19 2019	RNA	1	low grade glioma	Homo sapiens	gender: Female	age: 33.9603	treatment therapy: RT	igs: IGS-17	type.of.sugery: Biopsy	histology: AA GrII	performance: 0	idh.status: mutated	progression free survival (pfs) event: Yes	pfs (days): 757	total RNA	Total RNA was extracted from resection material of	Biotin	As per manufacturer (Illumina)	9606	RNA was hybridized to each array according to the	The arrays were scanned using standard Illumina	raw data file and column: DASL.2; 102217-51	The data was quantile normalized using	GPL14951	Pim,,French	p.french@erasmusmc.nl	+31 10 70443 33	Neurooncology	Neurology	Erasmus MC	Dr Molewaterplein 50	Rotterdam	2040CA	Netherlands	NONE	29377		GSM2881685
57	LGG_22033_X 279	GSM2881686	9.049409911	Public on Mar 19 2019	Dec 08 2017	Mar 19 2019	RNA	1	low grade glioma	Homo sapiens	gender: Female	age: 41.191	treatment therapy: TMZ	igs: IGS-17	type.of.sugery: Partial removal	histology: AA GrII	performance: 0	idh.status: mutated	progression free survival (pfs) event: No	pfs (days): 1665	total RNA	Total RNA was extracted from resection material of	Biotin	As per manufacturer (Illumina)	9606	RNA was hybridized to each array according to the	The arrays were scanned using standard Illumina	raw data file and column: DASL.1; X102012.092	The data was quantile normalized using	GPL14951	Pim,,French	p.french@erasmusmc.nl	+31 10 70443 33	Neurooncology	Neurology	Erasmus MC	Dr Molewaterplein 50	Rotterdam	2040CA	Netherlands	NONE	29377		GSM2881686
58	LGG_22033_X 281	GSM2881687	10.25262395	Public on Mar 19 2019	Dec 08 2017	Mar 19 2019	RNA	1	low grade glioma	Homo sapiens	gender: Female	age: 41.796	treatment therapy: RT	igs: IGS-17	type.of.sugery: Biopsy	histology: AOD GrII	performance: 1	idh.status: undetermined	progression free survival (pfs) event: Yes	pfs (days): 392	total RNA	Total RNA was extracted from resection material of	Biotin	As per manufacturer (Illumina)	9606	RNA was hybridized to each array according to the	The arrays were scanned using standard Illumina	raw data file and column: DASL.5; X102413.16	The data was quantile normalized using	GPL14951	Pim,,French	p.french@erasmusmc.nl	+31 10 70443 33	Neurooncology	Neurology	Erasmus MC	Dr Molewaterplein 50	Rotterdam	2040CA	Netherlands	NONE	29377		GSM2881687
59	LGG_22033_X 283	GSM2881688	10.52105652	Public on Mar 19 2019	Dec 08 2017	Mar 19 2019	RNA	1	low grade glioma	Homo sapiens	gender: Female	age: 21.9329	treatment therapy: RT	igs: IGS-17	type.of.sugery: Partial removal	histology: AA GrII	performance: 0	idh.status: mutated	progression free survival (pfs) event: No	pfs (days): 1	total RNA	Total RNA was extracted from resection material of	Biotin	As per manufacturer (Illumina)	9606	RNA was hybridized to each array according to the	The arrays were scanned using standard Illumina	raw data file and column: DASL.3; X1022329.003	The data was quantile normalized using	GPL14951	Pim,,French	p.french@erasmusmc.nl	+31 10 70443 33	Neurooncology	Neurology	Erasmus MC	Dr Molewaterplein 50	Rotterdam	2040CA	Netherlands	NONE	29377		GSM2881688
60	LGG_22033_X 284	GSM2881689	9.787455634	Public on Mar 19 2019	Dec 08 2017	Mar 19 2019	RNA	1	low grade glioma	Homo sapiens	gender: Male	age: 50.6448	treatment therapy: RT	igs: r.X9	type.of.sugery: Biopsy	histology: AOD GrII	performance: 0	idh.status: mutated	progression free survival (pfs) event: Yes	pfs (days): 696	total RNA	Total RNA was extracted from resection material of	Biotin	As per manufacturer (Illumina)	9606	RNA was hybridized to each array according to the	The arrays were scanned using standard Illumina	raw data file and column: DASL.2; 102217-39	The data was quantile normalized using	GPL14951	Pim,,French	p.french@erasmusmc.nl	+31 10 70443 33	Neurooncology	Neurology	Erasmus MC	Dr Molewaterplein 50	Rotterdam	2040CA	Netherlands	NONE	29377		GSM2881689
61	LGG_22033_X 286	GSM2881690	8.342206794	Public on Mar 19 2019	Dec 08 2017	Mar 19 2019	RNA	1	low grade glioma	Homo sapiens	gender: Female	age: 36.2656	treatment therapy: RT	igs: IGS-17	type.of.sugery: Partial removal	histology: AOA GrII	performance: 1	idh.status: mutated	progression free survival (pfs) event: Yes	pfs (days): 691	total RNA	Total RNA was extracted from resection material of	Biotin	As per manufacturer (Illumina)	9606	RNA was hybridized to each array according to the	The arrays were scanned using standard Illumina	raw data file and column: DASL.2; 102217-55	The data was quantile normalized using	GPL14951	Pim,,French	p.french@erasmusmc.nl	+31 10 70443 33	Neurooncology	Neurology	Erasmus MC	Dr Molewaterplein 50	Rotterdam	2040CA	Netherlands	NONE	29377		GSM2881690
62	LGG_22033_X 289	GSM2881691	10.23753762	Public on Mar 19 2019	Dec 08 2017	Mar 19 2019	RNA	1	low grade glioma	Homo sapiens	gender: Female	age: 48.2081	treatment therapy: RT	igs: IGS-9	type.of.sugery: Total removal	histology: AOD GrII	performance: 0	idh.status: undetermined	progression free survival (pfs) event: No	pfs (days): 1624	total RNA	Total RNA was extracted from resection material of	Biotin	As per manufacturer (Illumina)	9606	RNA was hybridized to each array according to the	The arrays were scanned using standard Illumina	raw data file and column: DASL.2; 102217-64	The data was quantile normalized using	GPL14951	Pim,,French	p.french@erasmusmc.nl	+31 10 70443 33	Neurooncology	Neurology	Erasmus MC	Dr Molewaterplein 50	Rotterdam	2040CA	Netherlands	NONE	29377		GSM2881691
63	LGG_22033_X 292	GSM2881692	9.520620984	Public on Mar 19 2019	Dec 08 2017	Mar 19 2019	RNA	1	low grade glioma	Homo sapiens	gender: Male	age: 50.7926	treatment therapy: RT	igs: IGS-9	type.of.sugery: Biopsy	histology: AA GrII	performance: 1	idh.status: mutated	progression free survival (pfs) event: No	pfs (days): 1686	total RNA	Total RNA was extracted from resection material of	Biotin	As per manufacturer (Illumina)	9606	RNA was hybridized to each array according to the	The arrays were scanned using standard Illumina	raw data file and column: DASL.1; X102012.051	The data was quantile normalized using	GPL14951	Pim,,French	p.french@erasmusmc.nl	+31 10 70443 33	Neurooncology	Neurology	Erasmus MC	Dr Molewaterplein 50	Rotterdam	2040CA	Netherlands	NONE	29377		GSM2881692
64	LGG_22033_X 296	GSM2881693	10.21968611	Public on Mar 19 2019	Dec 08 2017	Mar 19 2019	RNA	1	low grade glioma	Homo sapiens	gender: Male	age: 46.59	treatment therapy: RT	igs: IGS-9	type.of.sugery: Partial removal	histology: AOD GrII	performance: 0	idh.status: mutated	progression free survival (pfs) event: No	pfs (days): 1618	total RNA	Total RNA was extracted from resection material of	Biotin	As per manufacturer (Illumina)	9606	RNA was hybridized to each array according to the	The arrays were scanned using standard Illumina	raw data file and column: DASL.1; X102012.050	The data was quantile normalized using	GPL14951	Pim,,French	p.french@erasmusmc.nl	+31 10 70443 33	Neurooncology	Neurology	Erasmus MC	Dr Molewaterplein 50	Rotterdam	2040CA	Netherlands	NONE	29377		GSM2881693
65	LGG_22033_X 300	GSM2881694	10.33840077	Public on Mar 19 2019	Dec 08 2017	Mar 19 2019	RNA	1	low grade glioma	Homo sapiens	gender: Female	age: 42.5708	treatment therapy: TMZ	igs: r.X9	type.of.sugery: Partial removal	histology: AA GrII	performance: 0	idh.status: mutated	progression free survival (pfs) event: Yes	pfs (days): 1322	total RNA	Total RNA was extracted from resection material of	Biotin	As per manufacturer (Illumina)	9606	RNA was hybridized to each array according to the	The arrays were scanned using standard Illumina	raw data file and column: DASL.1; X102012.089	The data was quantile normalized using	GPL14951	Pim,,French	p.french@erasmusmc.nl	+31 10 70443 33	Neurooncology	Neurology	Erasmus MC	Dr Molewaterplein 50	Rotterdam	2040CA	Netherlands	NONE	29377		GSM2881694
66	LGG_22033_X 31	GSM2881695	10.8135417	Public on Mar 19 2019	Dec 08 2017	Mar 19 2019	RNA	1	low grade glioma	Homo sapiens	gender: Female	age: 53.1389	treatment therapy: RT	igs: r.X17	type.of.sugery: Partial removal	histology: AA GrII	performance: 0	idh.status: mutated	progression free survival (pfs) event: Yes	pfs (days): 1728	total RNA	Total RNA was extracted from resection material of	Biotin	As per manufacturer (Illumina)	9606	RNA was hybridized to each array according to the	The arrays were scanned using standard Illumina	raw data file and column: DASL.2; 102217-82	The data was quantile normalized using	GPL14951	Pim,,French	p.french@erasmusmc.nl	+31 10 70443 33	Neurooncology	Neurology	Erasmus MC	Dr Molewaterplein 50	Rotterdam	2040CA	Netherlands	NONE	29377		GSM2881695

67	LGG_22033_X322	GSM2881696	11.06338577	Public on Mar 19 2019	Dec 08 2017	Mar 19 2019	RNA	1	low grade glioma	Homo sapiens	gender: Male	age: 51.6277	treatment therapy: TMZ	igs: IGS-17	type.of.sugery: Biopsy	histology: AOD GrII	performance: 1	idh.status: normal	progression free survival (pfs) event: No	pfs (days): 1569	total RNA	Total RNA was extracted from resection material of	Biotin	As per manufacturer (Illumina)	9606	RNA was hybridized to each array according to the	The arrays were scanned using standard Illumina	raw data file and column: DASL.3; X1022329.012	The data was quantile normalized using	GPL14951	Pim,,French	p.french@erasmusmc.nl	+31 10 70443 33	Neurooncology	Neurology	Erasmus MC	Dr Molewaterplein 50	Rotterdam	2040CA	Netherlands	NONE	29377		GSM2881696
68	LGG_22033_X323	GSM2881697	11.3019979	Public on Mar 19 2019	Dec 08 2017	Mar 19 2019	RNA	1	low grade glioma	Homo sapiens	gender: Male	age: 40.0931	treatment therapy: TMZ	igs: IGS-17	type.of.sugery: Partial removal	histology: AOA GrII	performance: 1	idh.status: mutated	progression free survival (pfs) event: Yes	pfs (days): 865	total RNA	Total RNA was extracted from resection material of	Biotin	As per manufacturer (Illumina)	9606	RNA was hybridized to each array according to the	The arrays were scanned using standard Illumina	raw data file and column: DASL.0; 1394	The data was quantile normalized using	GPL14951	Pim,,French	p.french@erasmusmc.nl	+31 10 70443 33	Neurooncology	Neurology	Erasmus MC	Dr Molewaterplein 50	Rotterdam	2040CA	Netherlands	NONE	29377		GSM2881697
69	LGG_22033_X329	GSM2881698	12.00926901	Public on Mar 19 2019	Dec 08 2017	Mar 19 2019	RNA	1	low grade glioma	Homo sapiens	gender: Male	age: 32.104	treatment therapy: RT	igs: IGS-17	type.of.sugery: Partial removal	histology: AOA GrII	performance: 0	idh.status: mutated	progression free survival (pfs) event: Yes	pfs (days): 201	total RNA	Total RNA was extracted from resection material of	Biotin	As per manufacturer (Illumina)	9606	RNA was hybridized to each array according to the	The arrays were scanned using standard Illumina	raw data file and column: DASL.2; 102217-91	The data was quantile normalized using	GPL14951	Pim,,French	p.french@erasmusmc.nl	+31 10 70443 33	Neurooncology	Neurology	Erasmus MC	Dr Molewaterplein 50	Rotterdam	2040CA	Netherlands	NONE	29377		GSM2881698
70	LGG_22033_X33	GSM2881699	10.49338326	Public on Mar 19 2019	Dec 08 2017	Mar 19 2019	RNA	1	low grade glioma	Homo sapiens	gender: Male	age: 50.1383	treatment therapy: RT	igs: IGS-17	type.of.sugery: Partial removal	histology: AOD GrII	performance: 1	idh.status: mutated	progression free survival (pfs) event: Yes	pfs (days): 665	total RNA	Total RNA was extracted from resection material of	Biotin	As per manufacturer (Illumina)	9606	RNA was hybridized to each array according to the	The arrays were scanned using standard Illumina	raw data file and column: DASL.2; 102217-83	The data was quantile normalized using	GPL14951	Pim,,French	p.french@erasmusmc.nl	+31 10 70443 33	Neurooncology	Neurology	Erasmus MC	Dr Molewaterplein 50	Rotterdam	2040CA	Netherlands	NONE	29377		GSM2881699
71	LGG_22033_X338	GSM2881700	10.93450039	Public on Mar 19 2019	Dec 08 2017	Mar 19 2019	RNA	1	low grade glioma	Homo sapiens	gender: Male	age: 37.1663	treatment therapy: TMZ	igs: IGS-17	type.of.sugery: Total removal	histology: AOA GrII	performance: 0	idh.status: mutated	progression free survival (pfs) event: Yes	pfs (days): 1429	total RNA	Total RNA was extracted from resection material of	Biotin	As per manufacturer (Illumina)	9606	RNA was hybridized to each array according to the	The arrays were scanned using standard Illumina	raw data file and column: DASL.2; 102217-65	The data was quantile normalized using	GPL14951	Pim,,French	p.french@erasmusmc.nl	+31 10 70443 33	Neurooncology	Neurology	Erasmus MC	Dr Molewaterplein 50	Rotterdam	2040CA	Netherlands	NONE	29377		GSM2881700
72	LGG_22033_X346	GSM2881701	10.54156083	Public on Mar 19 2019	Dec 08 2017	Mar 19 2019	RNA	1	low grade glioma	Homo sapiens	gender: Male	age: 19.4771	treatment therapy: TMZ	igs: IGS-9	type.of.sugery: Partial removal	histology: AA GrII	performance: 0	idh.status: undetermined	progression free survival (pfs) event: Yes	pfs (days): 670	total RNA	Total RNA was extracted from resection material of	Biotin	As per manufacturer (Illumina)	9606	RNA was hybridized to each array according to the	The arrays were scanned using standard Illumina	raw data file and column: DASL.2; 102217-66	The data was quantile normalized using	GPL14951	Pim,,French	p.french@erasmusmc.nl	+31 10 70443 33	Neurooncology	Neurology	Erasmus MC	Dr Molewaterplein 50	Rotterdam	2040CA	Netherlands	NONE	29377		GSM2881701
73	LGG_22033_X348	GSM2881702	10.71621967	Public on Mar 19 2019	Dec 08 2017	Mar 19 2019	RNA	1	low grade glioma	Homo sapiens	gender: Female	age: 53.3689	treatment therapy: TMZ	igs: IGS-17	type.of.sugery: Partial removal	histology: AOD GrII	performance: 0	idh.status: mutated	progression free survival (pfs) event: No	pfs (days): 1421	total RNA	Total RNA was extracted from resection material of	Biotin	As per manufacturer (Illumina)	9606	RNA was hybridized to each array according to the	The arrays were scanned using standard Illumina	raw data file and column: DASL.1; X102012.107	The data was quantile normalized using	GPL14951	Pim,,French	p.french@erasmusmc.nl	+31 10 70443 33	Neurooncology	Neurology	Erasmus MC	Dr Molewaterplein 50	Rotterdam	2040CA	Netherlands	NONE	29377		GSM2881702
74	LGG_22033_X35	GSM2881703	10.58995288	Public on Mar 19 2019	Dec 08 2017	Mar 19 2019	RNA	1	low grade glioma	Homo sapiens	gender: Male	age: 30.527	treatment therapy: RT	igs: IGS-17	type.of.sugery: Total removal	histology: AOD GrII	performance: 0	idh.status: mutated	progression free survival (pfs) event: Yes	pfs (days): 668	total RNA	Total RNA was extracted from resection material of	Biotin	As per manufacturer (Illumina)	9606	RNA was hybridized to each array according to the	The arrays were scanned using standard Illumina	raw data file and column: DASL.1; X102012.084	The data was quantile normalized using	GPL14951	Pim,,French	p.french@erasmusmc.nl	+31 10 70443 33	Neurooncology	Neurology	Erasmus MC	Dr Molewaterplein 50	Rotterdam	2040CA	Netherlands	NONE	29377		GSM2881703
75	LGG_22033_X350	GSM2881704	10.34539253	Public on Mar 19 2019	Dec 08 2017	Mar 19 2019	RNA	1	low grade glioma	Homo sapiens	gender: Male	age: 40.8049	treatment therapy: TMZ	igs: IGS-9	type.of.sugery: Biopsy	histology: AOD GrII	performance: 1	idh.status: mutated	progression free survival (pfs) event: Yes	pfs (days): 1244	total RNA	Total RNA was extracted from resection material of	Biotin	As per manufacturer (Illumina)	9606	RNA was hybridized to each array according to the	The arrays were scanned using standard Illumina	raw data file and column: DASL.3; X1022329.013	The data was quantile normalized using	GPL14951	Pim,,French	p.french@erasmusmc.nl	+31 10 70443 33	Neurooncology	Neurology	Erasmus MC	Dr Molewaterplein 50	Rotterdam	2040CA	Netherlands	NONE	29377		GSM2881704
76	LGG_22033_X351	GSM2881705	9.750495027	Public on Mar 19 2019	Dec 08 2017	Mar 19 2019	RNA	1	low grade glioma	Homo sapiens	gender: Female	age: 69.4237	treatment therapy: RT	igs: IGS-9	type.of.sugery: Biopsy	histology: AOD GrII	performance: 1	idh.status: mutated	progression free survival (pfs) event: No	pfs (days): 1581	total RNA	Total RNA was extracted from resection material of	Biotin	As per manufacturer (Illumina)	9606	RNA was hybridized to each array according to the	The arrays were scanned using standard Illumina	raw data file and column: DASL.2; 102217-52	The data was quantile normalized using	GPL14951	Pim,,French	p.french@erasmusmc.nl	+31 10 70443 33	Neurooncology	Neurology	Erasmus MC	Dr Molewaterplein 50	Rotterdam	2040CA	Netherlands	NONE	29377		GSM2881705
77	LGG_22033_X352	GSM2881706	10.3203221	Public on Mar 19 2019	Dec 08 2017	Mar 19 2019	RNA	1	low grade glioma	Homo sapiens	gender: Male	age: 26.0643	treatment therapy: RT	igs: IGS-17	type.of.sugery: Partial removal	histology: AA GrII	performance: 0	idh.status: mutated	progression free survival (pfs) event: Yes	pfs (days): 380	total RNA	Total RNA was extracted from resection material of	Biotin	As per manufacturer (Illumina)	9606	RNA was hybridized to each array according to the	The arrays were scanned using standard Illumina	raw data file and column: DASL.1; X102012.106	The data was quantile normalized using	GPL14951	Pim,,French	p.french@erasmusmc.nl	+31 10 70443 33	Neurooncology	Neurology	Erasmus MC	Dr Molewaterplein 50	Rotterdam	2040CA	Netherlands	NONE	29377		GSM2881706
78	LGG_22033_X357	GSM2881707	8.800223681	Public on Mar 19 2019	Dec 08 2017	Mar 19 2019	RNA	1	low grade glioma	Homo sapiens	gender: Male	age: 38.3354	treatment therapy: TMZ	igs: IGS-17	type.of.sugery: Total removal	histology: AA GrII	performance: 0	idh.status: mutated	progression free survival (pfs) event: Yes	pfs (days): 1288	total RNA	Total RNA was extracted from resection material of	Biotin	As per manufacturer (Illumina)	9606	RNA was hybridized to each array according to the	The arrays were scanned using standard Illumina	raw data file and column: DASL.1; X102012.102	The data was quantile normalized using	GPL14951	Pim,,French	p.french@erasmusmc.nl	+31 10 70443 33	Neurooncology	Neurology	Erasmus MC	Dr Molewaterplein 50	Rotterdam	2040CA	Netherlands	NONE	29377		GSM2881707
79	LGG_22033_X362	GSM2881708	11.49699461	Public on Mar 19 2019	Dec 08 2017	Mar 19 2019	RNA	1	low grade glioma	Homo sapiens	gender: Female	age: 51.4962	treatment therapy: RT	igs: r.X17	type.of.sugery: Partial removal	histology: AOD GrII	performance: 1	idh.status: undetermined	progression free survival (pfs) event: Yes	pfs (days): 499	total RNA	Total RNA was extracted from resection material of	Biotin	As per manufacturer (Illumina)	9606	RNA was hybridized to each array according to the	The arrays were scanned using standard Illumina	raw data file and column: DASL.4; X102380.11	The data was quantile normalized using	GPL14951	Pim,,French	p.french@erasmusmc.nl	+31 10 70443 33	Neurooncology	Neurology	Erasmus MC	Dr Molewaterplein 50	Rotterdam	2040CA	Netherlands	NONE	29377		GSM2881708
80	LGG_22033_X364	GSM2881709	10.42586227	Public on Mar 19 2019	Dec 08 2017	Mar 19 2019	RNA	1	low grade glioma	Homo sapiens	gender: Male	age: 58.7324	treatment therapy: RT	igs: IGS-17	type.of.sugery: Partial removal	histology: AOA GrII	performance: 0	idh.status: mutated	progression free survival (pfs) event: No	pfs (days): 1350	total RNA	Total RNA was extracted from resection material of	Biotin	As per manufacturer (Illumina)	9606	RNA was hybridized to each array according to the	The arrays were scanned using standard Illumina	raw data file and column: DASL.1; X102012.103	The data was quantile normalized using	GPL14951	Pim,,French	p.french@erasmusmc.nl	+31 10 70443 33	Neurooncology	Neurology	Erasmus MC	Dr Molewaterplein 50	Rotterdam	2040CA	Netherlands	NONE	29377		GSM2881709
81	LGG_22033_X369	GSM2881710	10.03643269	Public on Mar 19 2019	Dec 08 2017	Mar 19 2019	RNA	1	low grade glioma	Homo sapiens	gender: Male	age: 47.1732	treatment therapy: TMZ	igs: IGS-9	type.of.sugery: Total removal	histology: AOA GrII	performance: 0	idh.status: mutated	progression free survival (pfs) event: No	pfs (days): 1400	total RNA	Total RNA was extracted from resection material of	Biotin	As per manufacturer (Illumina)	9606	RNA was hybridized to each array according to the	The arrays were scanned using standard Illumina	raw data file and column: DASL.3; X1022329.005	The data was quantile normalized using	GPL14951	Pim,,French	p.french@erasmusmc.nl	+31 10 70443 33	Neurooncology	Neurology	Erasmus MC	Dr Molewaterplein 50	Rotterdam	2040CA	Netherlands	NONE	29377		GSM2881710
82	LGG_22033_X374	GSM2881711	10.01176926	Public on Mar 19 2019	Dec 08 2017	Mar 19 2019	RNA	1	low grade glioma	Homo sapiens	gender: Male	age: 21.8836	treatment therapy: RT	igs: IGS-17	type.of.sugery: Partial removal	histology: AA GrII	performance: 0	idh.status: mutated	progression free survival (pfs) event: Yes	pfs (days): 646	total RNA	Total RNA was extracted from resection material of	Biotin	As per manufacturer (Illumina)	9606	RNA was hybridized to each array according to the	The arrays were scanned using standard Illumina	raw data file and column: DASL.1; X102012.101	The data was quantile normalized using	GPL14951	Pim,,French	p.french@erasmusmc.nl	+31 10 70443 33	Neurooncology	Neurology	Erasmus MC	Dr Molewaterplein 50	Rotterdam	2040CA	Netherlands	NONE	29377		GSM2881711
83	LGG_22033_X380	GSM2881712	11.59408266	Public on Mar 19 2019	Dec 08 2017	Mar 19 2019	RNA	1	low grade glioma	Homo sapiens	gender: Male	age: 43.8084	treatment therapy: TMZ	igs: IGS-17	type.of.sugery: Partial removal	histology: AOD GrII	performance: 0	idh.status: mutated	progression free survival (pfs) event: No	pfs (days): 1175	total RNA	Total RNA was extracted from resection material of	Biotin	As per manufacturer (Illumina)	9606	RNA was hybridized to each array according to the	The arrays were scanned using standard Illumina	raw data file and column: DASL.1; X102012.105	The data was quantile normalized using	GPL14951	Pim,,French	p.french@erasmusmc.nl	+31 10 70443 33	Neurooncology	Neurology	Erasmus MC	Dr Molewaterplein 50	Rotterdam	2040CA	Netherlands	NONE	29377		GSM2881712
84	LGG_22033_X382	GSM2881713	9.353492426	Public on Mar 19 2019	Dec 08 2017	Mar 19 2019	RNA	1	low grade glioma	Homo sapiens	gender: Male	age: 41.6263	treatment therapy: RT	igs: IGS-17	type.of.sugery: Total removal	histology: AA GrII	performance: 1	idh.status: mutated	progression free survival (pfs) event: Yes	pfs (days): 1238	total RNA	Total RNA was extracted from resection material of	Biotin	As per manufacturer (Illumina)	9606	RNA was hybridized to each array according to the	The arrays were scanned using standard Illumina	raw data file and column: DASL.1; X102012.093	The data was quantile normalized using	GPL14951	Pim,,French	p.french@erasmusmc.nl	+31 10 70443 33	Neurooncology	Neurology	Erasmus MC	Dr Molewaterplein 50	Rotterdam	2040CA	Netherlands	NONE	29377		GSM2881713
85	LGG_22033_X386	GSM2881714	10.97990476	Public on Mar 19 2019	Dec 08 2017	Mar 19 2019	RNA	1	low grade glioma	Homo sapiens	gender: Male	age: 49.6646	treatment therapy: RT	igs: IGS-9	type.of.sugery: Partial removal	histology: AOD GrII	performance: 0	idh.status: mutated	progression free survival (pfs) event: Yes	pfs (days): 1521	total RNA	Total RNA was extracted from resection material of	Biotin	As per manufacturer (Illumina)	9606	RNA was hybridized to each array according to the	The arrays were scanned using standard Illumina	raw data file and column: DASL.5; X102413.17	The data was quantile normalized using	GPL14951	Pim,,French	p.french@erasmusmc.nl	+31 10 70443 33	Neurooncology	Neurology	Erasmus MC	Dr Molewaterplein 50	Rotterdam	2040CA	Netherlands	NONE	29377		GSM2881714
86	LGG_22033_X395	GSM2881715	11.42566822	Public on Mar 19 2019	Dec 08 2017	Mar 19 2019	RNA	1	low grade glioma	Homo sapiens	gender: Male	age: 41.1307	treatment therapy: TMZ	igs: IGS-17	type.of.sugery: Partial removal	histology: AOD GrII	performance: 0	idh.status: mutated	progression free survival (pfs) event: No	pfs (days): 1449	total RNA	Total RNA was extracted from resection material of	Biotin	As per manufacturer (Illumina)	9606	RNA was hybridized to each array according to the	The arrays were scanned using standard Illumina	raw data file and column: DASL.1; X102012.104	The data was quantile normalized using	GPL14951	Pim,,French	p.french@erasmusmc.nl	+31 10 70443 33	Neurooncology	Neurology	Erasmus MC	Dr Molewaterplein 50	Rotterdam	2040CA	Netherlands	NONE	29377		GSM2881715
87	LGG_22033_X396	GSM2881716	10.94724964	Public on Mar 19 2019	Dec 08 2017	Mar 19 2019	RNA	1	low grade glioma	Homo sapiens	gender: Female	age: 43.6057	treatment therapy: RT	igs: IGS-17	type.of.sugery: Total removal	histology: AOA GrII	performance: 1	idh.status: mutated	progression free survival (pfs) event: No	pfs (days): 1493	total RNA	Total RNA was extracted from resection material of	Biotin	As per manufacturer (Illumina)	9606	RNA was hybridized to each array according to the	The arrays were scanned using standard Illumina	raw data file and column: DASL.5; X102413.18	The data was quantile normalized using	GPL14951	Pim,,French	p.french@erasmusmc.nl	+31 10 70443 33	Neurooncology	Neurology	Erasmus MC	Dr Molewaterplein 50	Rotterdam	2040CA	Netherlands	NONE	29377		GSM2881716
88	LGG_22033_X403	GSM2881717	10.95206645	Public on Mar 19 2019	Dec 08 2017	Mar 19 2019	RNA	1	low grade glioma	Homo sapiens	gender: Male	age: 27.4689	treatment therapy: TMZ	igs: IGS-17	type.of.sugery: Partial removal	histology: AOA GrII	performance: 0	idh.status: undetermined	progression free survival (pfs) event: Yes	pfs (days): 1071	total RNA	Total RNA was extracted from resection material of	Biotin	As per manufacturer (Illumina)	9606	RNA was hybridized to each array according to the	The arrays were scanned using standard Illumina	raw data file and column: DASL.5; X102413.20	The data was quantile normalized using	GPL14951	Pim,,French	p.french@erasmusmc.nl	+31 10 70443 33	Neurooncology	Neurology	Erasmus MC	Dr Molewaterplein 50	Rotterdam	2040CA	Netherlands	NONE	29377		GSM2881717
89	LGG_22033_X405	GSM2881718	10.55663585	Public on Mar 19 2019	Dec 08 2017	Mar 19 2019	RNA	1	low grade glioma	Homo sapiens	gender: Male	age: 53.5688	treatment therapy: TMZ	igs: IGS-9	type.of.sugery: Partial removal	histology: AOD GrII	performance: 0	idh.status: mutated	progression free survival (pfs) event: Yes	pfs (days): 336	total RNA	Total RNA was extracted from resection material of	Biotin	As per manufacturer (Illumina)	9606	RNA was hybridized to each array according to the	The arrays were scanned using standard Illumina	raw data file and column: DASL.2; 102217-41	The data was quantile normalized using	GPL14951	Pim,,French	p.french@erasmusmc.nl	+31 10 70443 33	Neurooncology	Neurology	Erasmus MC	Dr Molewaterplein 50	Rotterdam	2040CA	Netherlands	NONE	29377		GSM2881718
90	LGG_22033_X406	GSM2881719	8.292509361	Public on Mar 19 2019	Dec 08 2017	Mar 19 2019	RNA	1	low grade glioma	Homo sapiens	gender: Female	age: 42.4695	treatment therapy: RT	igs: r.X9	type.of.sugery: Total removal	histology: AA GrII	performance: 0	idh.status: mutated	progression free survival (pfs) event: No	pfs (days): 1447	total RNA	Total RNA was extracted from resection material of	Biotin	As per manufacturer (Illumina)	9606	RNA was hybridized to each array according to the	The arrays were scanned using standard Illumina	raw data file and column: DASL.5; X102413.21	The data was quantile normalized using	GPL14951	Pim,,French	p.french@erasmusmc.nl	+31 10 70443 33	Neurooncology	Neurology	Erasmus MC	Dr Molewaterplein 50	Rotterdam	2040CA	Netherlands	NONE	29377		GSM2881719
91	LGG_22033_X407	GSM2881720	10.57242946	Public on Mar 19 2019	Dec 08 2017	Mar 19 2019	RNA	1	low grade glioma	Homo sapiens	gender: Male	age: 31.8932	treatment therapy: TMZ	igs: IGS-17	type.of.sugery: Biopsy	histology: AA GrII	performance: 1	idh.status: undetermined	progression free survival (pfs) event: No	pfs (days): 91	total RNA	Total RNA was extracted from resection material of	Biotin	As per manufacturer (Illumina)	9606	RNA was hybridized to each array according to the	The arrays were scanned using standard Illumina	raw data file and column: DASL.2; 102217-67	The data was quantile normalized using	GPL14951	Pim,,French	p.french@erasmusmc.nl	+31 10 70443 33	Neurooncology	Neurology	Erasmus MC	Dr Molewaterplein 50	Rotterdam	2040CA	Netherlands	NONE	29377		GSM2881720
92	LGG_22033_X408	GSM2881721	8.983816127	Public on Mar 19 2019	Dec 08 2017	Mar 19 2019	RNA	1	low grade glioma	Homo sapiens	gender: Female	age: 30.5243	treatment therapy: TMZ	igs: IGS-9	type.of.sugery: Partial removal	histology: AOD GrII	performance: 0	idh.status: mutated	progression free survival (pfs) event: Yes	pfs (days): 555	total RNA	Total RNA was extracted from resection material of	Biotin	As per manufacturer (Illumina)	9606	RNA was hybridized to each array according to the	The arrays were scanned using standard Illumina	raw data file and column: DASL.1; X102012.114	The data was quantile normalized using	GPL14951	Pim,,French	p.french@erasmusmc.nl	+31 10 70443 33	Neurooncology	Neurology	Erasmus MC	Dr Molewaterplein 50	Rotterdam	2040CA	Netherlands	NONE	29377		GSM2881721
93	LGG_22033_X41	GSM2881722	9.495767414	Public on Mar 19 2019	Dec 08 2017	Mar 19 2019	RNA	1	low grade glioma	Homo sapiens	gender: Male	age: 22.3272	treatment therapy: RT	igs: IGS-17	type.of.sugery: Partial removal	histology: AOD GrII	performance: 0	idh.status: mutated	progression free survival (pfs) event: Yes	pfs (days): 1422	total RNA	Total RNA was extracted from resection material of	Biotin	As per manufacturer (Illumina)	9606	RNA was hybridized to each array according to the	The arrays were scanned using standard Illumina	raw data file and column: DASL.1; X102012.086	The data was quantile normalized using	GPL14951	Pim,,French	p.french@erasmusmc.nl	+31 10 70443 33	Neurooncology	Neurology	Erasmus MC	Dr Molewaterplein 50	Rotterdam	2040CA	Netherlands	NONE	29377		GSM2881722
94	LGG_22033_X410	GSM2881723	9.858017349	Public on Mar 19 2019	Dec 08 2017	Mar 19 2019	RNA	1	low grade glioma	Homo sapiens	gender: Female	age: 48.742	treatment therapy: TMZ	igs: IGS-9	type.of.sugery: Partial removal	histology: AOD GrII	performance: 0	idh.status: mutated	progression free survival (pfs) event: No	pfs (days): 902	total RNA	Total RNA was extracted from resection material of	Biotin	As per manufacturer (Illumina)	9606	RNA was hybridized to each array according to the	The arrays were scanned using standard Illumina	raw data file and column: DASL.1; X102012.109	The data was quantile normalized using	GPL14951	Pim,,French	p.french@erasmusmc.nl	+31 10 70443 33	Neurooncology	Neurology	Erasmus MC	Dr Molewaterplein 50	Rotterdam	2040CA	Netherlands	NONE	29377		GSM2881723
95	LGG_22033_X414	GSM2881724	10.62879611	Public on Mar 19 2019	Dec 08 2017	Mar 19 2019	RNA	1	low grade glioma	Homo sapiens	gender: Female	age: 47.0144	treatment therapy: RT	igs: IGS-17	type.of.sugery: Total removal	histology: AOD GrII	performance: 0	idh.status: mutated	progression free survival (pfs) event: No	pfs (days): 1313	total RNA	Total RNA was extracted from resection material of	Biotin	As per manufacturer (Illumina)	9606	RNA was hybridized to each array according to the	The arrays were scanned using standard Illumina	raw data file and column: DASL.1; X102012.158	The data was quantile normalized using	GPL14951	Pim,,French	p.french@erasmusmc.nl	+31 10 70443 33	Neurooncology	Neurology	Erasmus MC	Dr Molewaterplein 50	Rotterdam	2040CA	Netherlands	NONE	29377		GSM2881724
96	LGG_22033_X417	GSM2881725	9.142910818	Public on Mar 19 2019	Dec 08 2017	Mar 19 2019	RNA	1	low grade glioma	Homo sapiens	gender: Male	age: 55.1513	treatment therapy: TMZ	igs: IGS-9	type.of.sugery: Partial removal	histology: AOD GrII	performance: 1	idh.status: mutated	progression free survival (pfs) event: No	pfs (days): 619	total RNA	Total RNA was extracted from resection material of	Biotin	As per manufacturer (Illumina)	9606	RNA was hybridized to each array according to the	The arrays were scanned using standard Illumina	raw data file and column: DASL.3; X1022329.009	The data was quantile normalized using	GPL14951	Pim,,French	p.french@erasmusmc.nl	+31 10 70443 33	Neurooncology	Neurology	Erasmus MC	Dr Molewaterplein 50	Rotterdam	2040CA	Netherlands	NONE	29377		GSM2881725
97	LGG_22033_X421	GSM2881726	10.88731964	Public on Mar 19 2019	Dec 08 2017	Mar 19 2019	RNA	1	low grade glioma	Homo sapiens	gender: Male	age: 40.2546	treatment therapy: RT	igs: IGS-17	type.of.sugery: Partial removal	histology: AOD GrII	performance: 2	idh.status: mutated	progression free survival (pfs) event: Yes	pfs (days): 1334	total RNA	Total RNA was extracted from resection material of	Biotin	As per manufacturer (Illumina)	9606	RNA was hybridized to each array according to the	The arrays were scanned using standard Illumina	raw data file and column: DASL.4; X102380.12	The data was quantile normalized using	GPL14951	Pim,,French	p.french@erasmusmc.nl	+31 10 70443 33	Neurooncology	Neurology	Erasmus MC	Dr Molewaterplein 50	Rotterdam	2040CA	Netherlands	NONE	29377		GSM2881726
98	LGG_22033_X423	GSM2881727	10.26732555	Public on Mar 19 2019	Dec 08 2017	Mar 19 2019	RNA	1	low grade glioma	Homo sapiens	gender: Male	age: 49.1061	treatment therapy: RT	igs: IGS-17	type.of.sugery: Biopsy	histology: AOA GrII	performance: 0	idh.status: mutated	progression free survival (pfs) event: No	pfs (days): 1375	total RNA	Total RNA was extracted from resection material of	Biotin	As per manufacturer (Illumina)	9606	RNA was hybridized to each array according to the	The arrays were scanned using standard Illumina	raw data file and column: DASL.2; 102217-12	The data was quantile normalized using	GPL14951	Pim,,French	p.french@erasmusmc.nl	+31 10 70443 33	Neurooncology	Neurology	Erasmus MC	Dr Molewaterplein 50	Rotterdam	2040CA	Netherlands	NONE	29377		GSM2881727
99	LGG_22033_X429	GSM2881728	9.315063984	Public on Mar 19 2019	Dec 08 2017	Mar 19 2019	RNA	1	low grade glioma	Homo sapiens	gender: Female	age: 47.0253	treatment therapy: TMZ	igs: r.X9	type.of.sugery: Total removal	histology: AOD GrII	performance: 1	idh.status: mutated	progression free survival (pfs) event: Yes	pfs (days): 1071	total RNA	Total RNA was extracted from resection material of	Biotin	As per manufacturer (Illumina)	9606	RNA was hybridized to each array according to the	The arrays were scanned using standard Illumina	raw data file and column: DASL.4; X102380.13	The data was quantile normalized using	GPL14951	Pim,,French	p.french@erasmusmc.nl	+31 10 70443 33	Neurooncology	Neurology	Erasmus MC	Dr Molewaterplein 50	Rotterdam	2040CA	Netherlands	NONE	29377		GSM2881728
100	LGG_22033_X43	GSM2881729	10.253632	Public on Mar 19 2019	Dec 08 2017	Mar 19 2019	RNA	1	low grade glioma	Homo sapiens	gender: Male	age: 43.9863	treatment therapy: TMZ	igs: IGS-17	type.of.sugery: Partial removal	histology: AA GrII	performance: 1	idh.status: mutated	progression free survival (pfs) event: Yes	pfs (days): 1659	total RNA	Total RNA was extracted from resection material of	Biotin	As per manufacturer (Illumina)	9606	RNA was hybridized to each array according to the	The arrays were scanned using standard Illumina	raw data file and column: DASL.1; X102012.079	The data was quantile normalized using	GPL14951	Pim,,French	p.french@erasmusmc.nl	+31 10 70443 33	Neurooncology	Neurology	Erasmus MC	Dr Molewaterplein 50	Rotterdam	2040CA	Netherlands	NONE	29377		GSM2881729

101	LGG_22033_X 431	GSM2881730	10.33547866	Public on Mar 19 2019	Dec 08 2017	Mar 19 2019	RNA	1	low grade glioma	Homo sapiens	gender: Female	age: 46.4148	treatment therapy: TMZ	igs: IGS-9	type.of.sugery: Partial removal	histology: AOA GrII	performance: 0	idh.status: mutated	progression free survival (pfs) event: No	pfs (days): 1232	total RNA	Total RNA was extracted from resection material of	Biotin	As per manufacturer (Illumina)	9606	RNA was hybridized to each array according to the	The arrays were scanned using standard Illumina	raw data file and column: DASL.2; 102217-07	The data was quantile normalized using	GPL14951	Pim,,French	p.french@erasmusmc.nl	+31 10 70443 33	Neurooncology	Neurology	Erasmus MC	Dr Molewaterplein 50	Rotterdam	2040CA	Netherlands	NONE	29377		GSM2881730
102	LGG_22033_X 435	GSM2881731	9.7121301	Public on Mar 19 2019	Dec 08 2017	Mar 19 2019	RNA	1	low grade glioma	Homo sapiens	gender: Male	age: 31.5099	treatment therapy: TMZ	igs: IGS-17	type.of.sugery: Partial removal	histology: AA GrII	performance: 0	idh.status: mutated	progression free survival (pfs) event: No	pfs (days): 1137	total RNA	Total RNA was extracted from resection material of	Biotin	As per manufacturer (Illumina)	9606	RNA was hybridized to each array according to the	The arrays were scanned using standard Illumina	raw data file and column: DASL.1; X102012.116	The data was quantile normalized using	GPL14951	Pim,,French	p.french@erasmusmc.nl	+31 10 70443 33	Neurooncology	Neurology	Erasmus MC	Dr Molewaterplein 50	Rotterdam	2040CA	Netherlands	NONE	29377		GSM2881731
103	LGG_22033_X 437	GSM2881732	9.239162537	Public on Mar 19 2019	Dec 08 2017	Mar 19 2019	RNA	1	low grade glioma	Homo sapiens	gender: Female	age: 58.8255	treatment therapy: RT	igs: IGS-9	type.of.sugery: Partial removal	histology: AOD GrII	performance: 0	idh.status: mutated	progression free survival (pfs) event: No	pfs (days): 1364	total RNA	Total RNA was extracted from resection material of	Biotin	As per manufacturer (Illumina)	9606	RNA was hybridized to each array according to the	The arrays were scanned using standard Illumina	raw data file and column: DASL.2; 102217-69	The data was quantile normalized using	GPL14951	Pim,,French	p.french@erasmusmc.nl	+31 10 70443 33	Neurooncology	Neurology	Erasmus MC	Dr Molewaterplein 50	Rotterdam	2040CA	Netherlands	NONE	29377		GSM2881732
104	LGG_22033_X 438	GSM2881733	10.03979765	Public on Mar 19 2019	Dec 08 2017	Mar 19 2019	RNA	1	low grade glioma	Homo sapiens	gender: Male	age: 53.6537	treatment therapy: TMZ	igs: IGS-9	type.of.sugery: Biopsy	histology: AOD GrII	performance: 0	idh.status: mutated	progression free survival (pfs) event: No	pfs (days): 1364	total RNA	Total RNA was extracted from resection material of	Biotin	As per manufacturer (Illumina)	9606	RNA was hybridized to each array according to the	The arrays were scanned using standard Illumina	raw data file and column: DASL.3; X1022329.008	The data was quantile normalized using	GPL14951	Pim,,French	p.french@erasmusmc.nl	+31 10 70443 33	Neurooncology	Neurology	Erasmus MC	Dr Molewaterplein 50	Rotterdam	2040CA	Netherlands	NONE	29377		GSM2881733
105	LGG_22033_X 440	GSM2881734	11.19805996	Public on Mar 19 2019	Dec 08 2017	Mar 19 2019	RNA	1	low grade glioma	Homo sapiens	gender: Male	age: 47.7016	treatment therapy: TMZ	igs: IGS-17	type.of.sugery: Partial removal	histology: AOD GrII	performance: 0	idh.status: undetermined	progression free survival (pfs) event: Yes	pfs (days): 661	total RNA	Total RNA was extracted from resection material of	Biotin	As per manufacturer (Illumina)	9606	RNA was hybridized to each array according to the	The arrays were scanned using standard Illumina	raw data file and column: DASL.5; X102413.23	The data was quantile normalized using	GPL14951	Pim,,French	p.french@erasmusmc.nl	+31 10 70443 33	Neurooncology	Neurology	Erasmus MC	Dr Molewaterplein 50	Rotterdam	2040CA	Netherlands	NONE	29377		GSM2881734
106	LGG_22033_X 441	GSM2881735	11.29409388	Public on Mar 19 2019	Dec 08 2017	Mar 19 2019	RNA	1	low grade glioma	Homo sapiens	gender: Male	age: 28.386	treatment therapy: RT	igs: IGS-9	type.of.sugery: Partial removal	histology: AA GrII	performance: 0	idh.status: undetermined	progression free survival (pfs) event: Yes	pfs (days): 526	total RNA	Total RNA was extracted from resection material of	Biotin	As per manufacturer (Illumina)	9606	RNA was hybridized to each array according to the	The arrays were scanned using standard Illumina	raw data file and column: DASL.4; X102380.14	The data was quantile normalized using	GPL14951	Pim,,French	p.french@erasmusmc.nl	+31 10 70443 33	Neurooncology	Neurology	Erasmus MC	Dr Molewaterplein 50	Rotterdam	2040CA	Netherlands	NONE	29377		GSM2881735
107	LGG_22033_X 45	GSM2881736	10.45899298	Public on Mar 19 2019	Dec 08 2017	Mar 19 2019	RNA	1	low grade glioma	Homo sapiens	gender: Male	age: 58.36	treatment therapy: TMZ	igs: IGS-17	type.of.sugery: Total removal	histology: AOD GrII	performance: 0	idh.status: mutated	progression free survival (pfs) event: Yes	pfs (days): 1073	total RNA	Total RNA was extracted from resection material of	Biotin	As per manufacturer (Illumina)	9606	RNA was hybridized to each array according to the	The arrays were scanned using standard Illumina	raw data file and column: DASL.5; X102413.02	The data was quantile normalized using	GPL14951	Pim,,French	p.french@erasmusmc.nl	+31 10 70443 33	Neurooncology	Neurology	Erasmus MC	Dr Molewaterplein 50	Rotterdam	2040CA	Netherlands	NONE	29377		GSM2881736
108	LGG_22033_X 450	GSM2881737	10.27276414	Public on Mar 19 2019	Dec 08 2017	Mar 19 2019	RNA	1	low grade glioma	Homo sapiens	gender: Female	age: 47.2936	treatment therapy: TMZ	igs: IGS-17	type.of.sugery: Biopsy	histology: AOA GrII	performance: 0	idh.status: undetermined	progression free survival (pfs) event: Yes	pfs (days): 82	total RNA	Total RNA was extracted from resection material of	Biotin	As per manufacturer (Illumina)	9606	RNA was hybridized to each array according to the	The arrays were scanned using standard Illumina	raw data file and column: DASL.1; X102012.125	The data was quantile normalized using	GPL14951	Pim,,French	p.french@erasmusmc.nl	+31 10 70443 33	Neurooncology	Neurology	Erasmus MC	Dr Molewaterplein 50	Rotterdam	2040CA	Netherlands	NONE	29377		GSM2881737
109	LGG_22033_X 457	GSM2881738	10.775871	Public on Mar 19 2019	Dec 08 2017	Mar 19 2019	RNA	1	low grade glioma	Homo sapiens	gender: Male	age: 63.5127	treatment therapy: TMZ	igs: r.X17	type.of.sugery: Biopsy	histology: AA GrII	performance: 1	idh.status: undetermined	progression free survival (pfs) event: Yes	pfs (days): 57	total RNA	Total RNA was extracted from resection material of	Biotin	As per manufacturer (Illumina)	9606	RNA was hybridized to each array according to the	The arrays were scanned using standard Illumina	raw data file and column: DASL.3; X1022329.023	The data was quantile normalized using	GPL14951	Pim,,French	p.french@erasmusmc.nl	+31 10 70443 33	Neurooncology	Neurology	Erasmus MC	Dr Molewaterplein 50	Rotterdam	2040CA	Netherlands	NONE	29377		GSM2881738
110	LGG_22033_X 458	GSM2881739	9.685175516	Public on Mar 19 2019	Dec 08 2017	Mar 19 2019	RNA	1	low grade glioma	Homo sapiens	gender: Male	age: 36.0767	treatment therapy: RT	igs: IGS-17	type.of.sugery: Partial removal	histology: AOA GrII	performance: 0	idh.status: mutated	progression free survival (pfs) event: No	pfs (days): 404	total RNA	Total RNA was extracted from resection material of	Biotin	As per manufacturer (Illumina)	9606	RNA was hybridized to each array according to the	The arrays were scanned using standard Illumina	raw data file and column: DASL.4; X102380.15	The data was quantile normalized using	GPL14951	Pim,,French	p.french@erasmusmc.nl	+31 10 70443 33	Neurooncology	Neurology	Erasmus MC	Dr Molewaterplein 50	Rotterdam	2040CA	Netherlands	NONE	29377		GSM2881739
111	LGG_22033_X 46	GSM2881740	10.4220173	Public on Mar 19 2019	Dec 08 2017	Mar 19 2019	RNA	1	low grade glioma	Homo sapiens	gender: Female	age: 33.588	treatment therapy: RT	igs: IGS-17	type.of.sugery: Total removal	histology: AOD GrII	performance: 0	idh.status: mutated	progression free survival (pfs) event: Yes	pfs (days): 137	total RNA	Total RNA was extracted from resection material of	Biotin	As per manufacturer (Illumina)	9606	RNA was hybridized to each array according to the	The arrays were scanned using standard Illumina	raw data file and column: DASL.2; 102217-05	The data was quantile normalized using	GPL14951	Pim,,French	p.french@erasmusmc.nl	+31 10 70443 33	Neurooncology	Neurology	Erasmus MC	Dr Molewaterplein 50	Rotterdam	2040CA	Netherlands	NONE	29377		GSM2881740
112	LGG_22033_X 463	GSM2881741	10.65056432	Public on Mar 19 2019	Dec 08 2017	Mar 19 2019	RNA	1	low grade glioma	Homo sapiens	gender: Female	age: 43.1841	treatment therapy: TMZ	igs: IGS-17	type.of.sugery: Partial removal	histology: AOA GrII	performance: 0	idh.status: mutated	progression free survival (pfs) event: No	pfs (days): 1266	total RNA	Total RNA was extracted from resection material of	Biotin	As per manufacturer (Illumina)	9606	RNA was hybridized to each array according to the	The arrays were scanned using standard Illumina	raw data file and column: DASL.1; X102012.126	The data was quantile normalized using	GPL14951	Pim,,French	p.french@erasmusmc.nl	+31 10 70443 33	Neurooncology	Neurology	Erasmus MC	Dr Molewaterplein 50	Rotterdam	2040CA	Netherlands	NONE	29377		GSM2881741
113	LGG_22033_X 464	GSM2881742	9.556862169	Public on Mar 19 2019	Dec 08 2017	Mar 19 2019	RNA	1	low grade glioma	Homo sapiens	gender: Female	age: 42.9131	treatment therapy: RT	igs: IGS-9	type.of.sugery: Biopsy	histology: AA GrII	performance: 0	idh.status: undetermined	progression free survival (pfs) event: No	pfs (days): 1316	total RNA	Total RNA was extracted from resection material of	Biotin	As per manufacturer (Illumina)	9606	RNA was hybridized to each array according to the	The arrays were scanned using standard Illumina	raw data file and column: DASL.1; X102012.153	The data was quantile normalized using	GPL14951	Pim,,French	p.french@erasmusmc.nl	+31 10 70443 33	Neurooncology	Neurology	Erasmus MC	Dr Molewaterplein 50	Rotterdam	2040CA	Netherlands	NONE	29377		GSM2881742
114	LGG_22033_X 466	GSM2881743	9.812142143	Public on Mar 19 2019	Dec 08 2017	Mar 19 2019	RNA	1	low grade glioma	Homo sapiens	gender: Female	age: 63.8385	treatment therapy: RT	igs: r.X9	type.of.sugery: Partial removal	histology: AOD GrII	performance: 0	idh.status: mutated	progression free survival (pfs) event: No	pfs (days): 1341	total RNA	Total RNA was extracted from resection material of	Biotin	As per manufacturer (Illumina)	9606	RNA was hybridized to each array according to the	The arrays were scanned using standard Illumina	raw data file and column: DASL.2; 102217-21	The data was quantile normalized using	GPL14951	Pim,,French	p.french@erasmusmc.nl	+31 10 70443 33	Neurooncology	Neurology	Erasmus MC	Dr Molewaterplein 50	Rotterdam	2040CA	Netherlands	NONE	29377		GSM2881743
115	LGG_22033_X 474	GSM2881744	11.63967614	Public on Mar 19 2019	Dec 08 2017	Mar 19 2019	RNA	1	low grade glioma	Homo sapiens	gender: Male	age: 32.9473	treatment therapy: TMZ	igs: IGS-17	type.of.sugery: Partial removal	histology: AOD GrII	performance: 1	idh.status: mutated	progression free survival (pfs) event: Yes	pfs (days): 383	total RNA	Total RNA was extracted from resection material of	Biotin	As per manufacturer (Illumina)	9606	RNA was hybridized to each array according to the	The arrays were scanned using standard Illumina	raw data file and column: DASL.1; X102012.112	The data was quantile normalized using	GPL14951	Pim,,French	p.french@erasmusmc.nl	+31 10 70443 33	Neurooncology	Neurology	Erasmus MC	Dr Molewaterplein 50	Rotterdam	2040CA	Netherlands	NONE	29377		GSM2881744
116	LGG_22033_X 476	GSM2881745	9.228699498	Public on Mar 19 2019	Dec 08 2017	Mar 19 2019	RNA	1	low grade glioma	Homo sapiens	gender: Male	age: 51.1348	treatment therapy: TMZ	igs: IGS-9	type.of.sugery: Partial removal	histology: AOD GrII	performance: 0	idh.status: mutated	progression free survival (pfs) event: Yes	pfs (days): 529	total RNA	Total RNA was extracted from resection material of	Biotin	As per manufacturer (Illumina)	9606	RNA was hybridized to each array according to the	The arrays were scanned using standard Illumina	raw data file and column: DASL.1; X102012.115	The data was quantile normalized using	GPL14951	Pim,,French	p.french@erasmusmc.nl	+31 10 70443 33	Neurooncology	Neurology	Erasmus MC	Dr Molewaterplein 50	Rotterdam	2040CA	Netherlands	NONE	29377		GSM2881745
117	LGG_22033_X 477	GSM2881746	9.936699177	Public on Mar 19 2019	Dec 08 2017	Mar 19 2019	RNA	1	low grade glioma	Homo sapiens	gender: Female	age: 46.1739	treatment therapy: TMZ	igs: IGS-9	type.of.sugery: Partial removal	histology: AOD GrII	performance: 0	idh.status: mutated	progression free survival (pfs) event: No	pfs (days): 979	total RNA	Total RNA was extracted from resection material of	Biotin	As per manufacturer (Illumina)	9606	RNA was hybridized to each array according to the	The arrays were scanned using standard Illumina	raw data file and column: DASL.2; 102217-70	The data was quantile normalized using	GPL14951	Pim,,French	p.french@erasmusmc.nl	+31 10 70443 33	Neurooncology	Neurology	Erasmus MC	Dr Molewaterplein 50	Rotterdam	2040CA	Netherlands	NONE	29377		GSM2881746
118	LGG_22033_X 478	GSM2881747	9.900054743	Public on Mar 19 2019	Dec 08 2017	Mar 19 2019	RNA	1	low grade glioma	Homo sapiens	gender: Female	age: 45.2156	treatment therapy: RT	igs: IGS-9	type.of.sugery: Total removal	histology: AA GrII	performance: 0	idh.status: mutated	progression free survival (pfs) event: No	pfs (days): 1146	total RNA	Total RNA was extracted from resection material of	Biotin	As per manufacturer (Illumina)	9606	RNA was hybridized to each array according to the	The arrays were scanned using standard Illumina	raw data file and column: DASL.2; 102217-09	The data was quantile normalized using	GPL14951	Pim,,French	p.french@erasmusmc.nl	+31 10 70443 33	Neurooncology	Neurology	Erasmus MC	Dr Molewaterplein 50	Rotterdam	2040CA	Netherlands	NONE	29377		GSM2881747
119	LGG_22033_X 483	GSM2881748	10.37570459	Public on Mar 19 2019	Dec 08 2017	Mar 19 2019	RNA	1	low grade glioma	Homo sapiens	gender: Male	age: 45.7851	treatment therapy: RT	igs: IGS-9	type.of.sugery: Partial removal	histology: AOA GrII	performance: 1	idh.status: mutated	progression free survival (pfs) event: Yes	pfs (days): 617	total RNA	Total RNA was extracted from resection material of	Biotin	As per manufacturer (Illumina)	9606	RNA was hybridized to each array according to the	The arrays were scanned using standard Illumina	raw data file and column: DASL.1; X102012.119	The data was quantile normalized using	GPL14951	Pim,,French	p.french@erasmusmc.nl	+31 10 70443 33	Neurooncology	Neurology	Erasmus MC	Dr Molewaterplein 50	Rotterdam	2040CA	Netherlands	NONE	29377		GSM2881748
120	LGG_22033_X 487	GSM2881749	11.19365873	Public on Mar 19 2019	Dec 08 2017	Mar 19 2019	RNA	1	low grade glioma	Homo sapiens	gender: Male	age: 50.4832	treatment therapy: RT	igs: IGS-9	type.of.sugery: Partial removal	histology: AOD GrII	performance: 1	idh.status: mutated	progression free survival (pfs) event: Yes	pfs (days): 566	total RNA	Total RNA was extracted from resection material of	Biotin	As per manufacturer (Illumina)	9606	RNA was hybridized to each array according to the	The arrays were scanned using standard Illumina	raw data file and column: DASL.1; X102012.154	The data was quantile normalized using	GPL14951	Pim,,French	p.french@erasmusmc.nl	+31 10 70443 33	Neurooncology	Neurology	Erasmus MC	Dr Molewaterplein 50	Rotterdam	2040CA	Netherlands	NONE	29377		GSM2881749
121	LGG_22033_X 488	GSM2881750	11.48314754	Public on Mar 19 2019	Dec 08 2017	Mar 19 2019	RNA	1	low grade glioma	Homo sapiens	gender: Male	age: 58.3354	treatment therapy: RT	igs: IGS-17	type.of.sugery: Biopsy	histology: AOD GrII	performance: 1	idh.status: mutated	progression free survival (pfs) event: No	pfs (days): 38	total RNA	Total RNA was extracted from resection material of	Biotin	As per manufacturer (Illumina)	9606	RNA was hybridized to each array according to the	The arrays were scanned using standard Illumina	raw data file and column: DASL.1; X102012.118	The data was quantile normalized using	GPL14951	Pim,,French	p.french@erasmusmc.nl	+31 10 70443 33	Neurooncology	Neurology	Erasmus MC	Dr Molewaterplein 50	Rotterdam	2040CA	Netherlands	NONE	29377		GSM2881750
122	LGG_22033_X 494	GSM2881751	10.75422818	Public on Mar 19 2019	Dec 08 2017	Mar 19 2019	RNA	1	low grade glioma	Homo sapiens	gender: Male	age: 28.0274	treatment therapy: TMZ	igs: IGS-9	type.of.sugery: Partial removal	histology: AOD GrII	performance: 0	idh.status: mutated	progression free survival (pfs) event: Yes	pfs (days): 484	total RNA	Total RNA was extracted from resection material of	Biotin	As per manufacturer (Illumina)	9606	RNA was hybridized to each array according to the	The arrays were scanned using standard Illumina	raw data file and column: DASL.1; X102012.117	The data was quantile normalized using	GPL14951	Pim,,French	p.french@erasmusmc.nl	+31 10 70443 33	Neurooncology	Neurology	Erasmus MC	Dr Molewaterplein 50	Rotterdam	2040CA	Netherlands	NONE	29377		GSM2881751
123	LGG_22033_X 496	GSM2881752	9.1471202	Public on Mar 19 2019	Dec 08 2017	Mar 19 2019	RNA	1	low grade glioma	Homo sapiens	gender: Male	age: 35.5647	treatment therapy: RT	igs: IGS-9	type.of.sugery: Partial removal	histology: AOD GrII	performance: 1	idh.status: mutated	progression free survival (pfs) event: No	pfs (days): 507	total RNA	Total RNA was extracted from resection material of	Biotin	As per manufacturer (Illumina)	9606	RNA was hybridized to each array according to the	The arrays were scanned using standard Illumina	raw data file and column: DASL.1; X102012.121	The data was quantile normalized using	GPL14951	Pim,,French	p.french@erasmusmc.nl	+31 10 70443 33	Neurooncology	Neurology	Erasmus MC	Dr Molewaterplein 50	Rotterdam	2040CA	Netherlands	NONE	29377		GSM2881752
124	LGG_22033_X 499	GSM2881753	9.875530889	Public on Mar 19 2019	Dec 08 2017	Mar 19 2019	RNA	1	low grade glioma	Homo sapiens	gender: Female	age: 37.4045	treatment therapy: RT	igs: IGS-17	type.of.sugery: Partial removal	histology: AOA GrII	performance: 0	idh.status: mutated	progression free survival (pfs) event: Yes	pfs (days): 797	total RNA	Total RNA was extracted from resection material of	Biotin	As per manufacturer (Illumina)	9606	RNA was hybridized to each array according to the	The arrays were scanned using standard Illumina	raw data file and column: DASL.2; 102217-47	The data was quantile normalized using	GPL14951	Pim,,French	p.french@erasmusmc.nl	+31 10 70443 33	Neurooncology	Neurology	Erasmus MC	Dr Molewaterplein 50	Rotterdam	2040CA	Netherlands	NONE	29377		GSM2881753
125	LGG_22033_X 5	GSM2881754	9.389700134	Public on Mar 19 2019	Dec 08 2017	Mar 19 2019	RNA	1	low grade glioma	Homo sapiens	gender: Male	age: 33.191	treatment therapy: TMZ	igs: IGS-17	type.of.sugery: Partial removal	histology: AA GrII	performance: 1	idh.status: normal	progression free survival (pfs) event: No	pfs (days): 1992	total RNA	Total RNA was extracted from resection material of	Biotin	As per manufacturer (Illumina)	9606	RNA was hybridized to each array according to the	The arrays were scanned using standard Illumina	raw data file and column: DASL.2; 102217-27	The data was quantile normalized using	GPL14951	Pim,,French	p.french@erasmusmc.nl	+31 10 70443 33	Neurooncology	Neurology	Erasmus MC	Dr Molewaterplein 50	Rotterdam	2040CA	Netherlands	NONE	29377		GSM2881754
126	LGG_22033_X 500	GSM2881755	8.518051372	Public on Mar 19 2019	Dec 08 2017	Mar 19 2019	RNA	1	low grade glioma	Homo sapiens	gender: Female	age: 31.6386	treatment therapy: RT	igs: IGS-17	type.of.sugery: Total removal	histology: AOA GrII	performance: 1	idh.status: mutated	progression free survival (pfs) event: No	pfs (days): 928	total RNA	Total RNA was extracted from resection material of	Biotin	As per manufacturer (Illumina)	9606	RNA was hybridized to each array according to the	The arrays were scanned using standard Illumina	raw data file and column: DASL.2; 102217-43	The data was quantile normalized using	GPL14951	Pim,,French	p.french@erasmusmc.nl	+31 10 70443 33	Neurooncology	Neurology	Erasmus MC	Dr Molewaterplein 50	Rotterdam	2040CA	Netherlands	NONE	29377		GSM2881755
127	LGG_22033_X 503	GSM2881756	8.730796918	Public on Mar 19 2019	Dec 08 2017	Mar 19 2019	RNA	1	low grade glioma	Homo sapiens	gender: Male	age: 48.23	treatment therapy: TMZ	igs: IGS-17	type.of.sugery: Total removal	histology: AOA GrII	performance: 1	idh.status: mutated	progression free survival (pfs) event: No	pfs (days): 1147	total RNA	Total RNA was extracted from resection material of	Biotin	As per manufacturer (Illumina)	9606	RNA was hybridized to each array according to the	The arrays were scanned using standard Illumina	raw data file and column: DASL.1; X102012.127	The data was quantile normalized using	GPL14951	Pim,,French	p.french@erasmusmc.nl	+31 10 70443 33	Neurooncology	Neurology	Erasmus MC	Dr Molewaterplein 50	Rotterdam	2040CA	Netherlands	NONE	29377		GSM2881756
128	LGG_22033_X 506	GSM2881757	11.89064774	Public on Mar 19 2019	Dec 08 2017	Mar 19 2019	RNA	1	low grade glioma	Homo sapiens	gender: Male	age: 61.8289	treatment therapy: TMZ	igs: IGS-17	type.of.sugery: Biopsy	histology: AA GrII	performance: 0	idh.status: mutated	progression free survival (pfs) event: Yes	pfs (days): 1093	total RNA	Total RNA was extracted from resection material of	Biotin	As per manufacturer (Illumina)	9606	RNA was hybridized to each array according to the	The arrays were scanned using standard Illumina	raw data file and column: DASL.2; 102217-08	The data was quantile normalized using	GPL14951	Pim,,French	p.french@erasmusmc.nl	+31 10 70443 33	Neurooncology	Neurology	Erasmus MC	Dr Molewaterplein 50	Rotterdam	2040CA	Netherlands	NONE	29377		GSM2881757
129	LGG_22033_X 509	GSM2881758	10.38936143	Public on Mar 19 2019	Dec 08 2017	Mar 19 2019	RNA	1	low grade glioma	Homo sapiens	gender: Female	age: 42.3847	treatment therapy: RT	igs: IGS-17	type.of.sugery: Partial removal	histology: AOD GrII	performance: 1	idh.status: mutated	progression free survival (pfs) event: No	pfs (days): 1255	total RNA	Total RNA was extracted from resection material of	Biotin	As per manufacturer (Illumina)	9606	RNA was hybridized to each array according to the	The arrays were scanned using standard Illumina	raw data file and column: DASL.4; X102380.16	The data was quantile normalized using	GPL14951	Pim,,French	p.french@erasmusmc.nl	+31 10 70443 33	Neurooncology	Neurology	Erasmus MC	Dr Molewaterplein 50	Rotterdam	2040CA	Netherlands	NONE	29377		GSM2881758
130	LGG_22033_X 510	GSM2881759	11.23434294	Public on Mar 19 2019	Dec 08 2017	Mar 19 2019	RNA	1	low grade glioma	Homo sapiens	gender: Male	age: 30.9706	treatment therapy: TMZ	igs: IGS-17	type.of.sugery: Partial removal	histology: AOA GrII	performance: 1	idh.status: mutated	progression free survival (pfs) event: Yes	pfs (days): 742	total RNA	Total RNA was extracted from resection material of	Biotin	As per manufacturer (Illumina)	9606	RNA was hybridized to each array according to the	The arrays were scanned using standard Illumina	raw data file and column: DASL.1; X102012.155	The data was quantile normalized using	GPL14951	Pim,,French	p.french@erasmusmc.nl	+31 10 70443 33	Neurooncology	Neurology	Erasmus MC	Dr Molewaterplein 50	Rotterdam	2040CA	Netherlands	NONE	29377		GSM2881759
131	LGG_22033_X 514	GSM2881760	9.231378694	Public on Mar 19 2019	Dec 08 2017	Mar 19 2019	RNA	1	low grade glioma	Homo sapiens	gender: Male	age: 57.1034	treatment therapy: RT	igs: r.X9	type.of.sugery: Partial removal	histology: AOD GrII	performance: 0	idh.status: mutated	progression free survival (pfs) event: No	pfs (days): 1189	total RNA	Total RNA was extracted from resection material of	Biotin	As per manufacturer (Illumina)	9606	RNA was hybridized to each array according to the	The arrays were scanned using standard Illumina	raw data file and column: DASL.1; X102012.120	The data was quantile normalized using	GPL14951	Pim,,French	p.french@erasmusmc.nl	+31 10 70443 33	Neurooncology	Neurology	Erasmus MC	Dr Molewaterplein 50	Rotterdam	2040CA	Netherlands	NONE	29377		GSM2881760
132	LGG_22033_X 516	GSM2881761	9.289560019	Public on Mar 19 2019	Dec 08 2017	Mar 19 2019	RNA	1	low grade glioma	Homo sapiens	gender: Female	age: 39.3539	treatment therapy: TMZ	igs: IGS-17	type.of.sugery: Partial removal	histology: AOA GrII	performance: 0	idh.status: mutated	progression free survival (pfs) event: No	pfs (days): 30	total RNA	Total RNA was extracted from resection material of	Biotin	As per manufacturer (Illumina)	9606	RNA was hybridized to each array according to the	The arrays were scanned using standard Illumina	raw data file and column: DASL.2; 102217-22	The data was quantile normalized using	GPL14951	Pim,,French	p.french@erasmusmc.nl	+31 10 70443 33	Neurooncology	Neurology	Erasmus MC	Dr Molewaterplein 50	Rotterdam	2040CA	Netherlands	NONE	29377		GSM2881761
133	LGG_22033_X 518	GSM2881762	9.343009953	Public on Mar 19 2019	Dec 08 2017	Mar 19 2019	RNA	1	low grade glioma	Homo sapiens	gender: Male	age: 53.2074	treatment therapy: RT	igs: IGS-9	type.of.sugery: Total removal	histology: AOD GrII	performance: 0	idh.status: mutated	progression free survival (pfs) event: No	pfs (days): 1139	total RNA	Total RNA was extracted from resection material of	Biotin	As per manufacturer (Illumina)	9606	RNA was hybridized to each array according to the	The arrays were scanned using standard Illumina	raw data file and column: DASL.1; X102012.098	The data was quantile normalized using	GPL14951	Pim,,French	p.french@erasmusmc.nl	+31 10 70443 33	Neurooncology	Neurology	Erasmus MC	Dr Molewaterplein 50	Rotterdam	2040CA	Netherlands	NONE	29377		GSM2881762
134	LGG_22033_X 520	GSM2881763	10.32540258	Public on Mar 19 2019	Dec 08 2017	Mar 19 2019	RNA	1	low grade glioma	Homo sapiens	gender: Male	age: 62.987	treatment therapy: TMZ	igs: IGS-17	type.of.sugery: Partial removal	histology: AOA GrII	performance: 1	idh.status: mutated	progression free survival (pfs) event: No	pfs (days): 504	total RNA	Total RNA was extracted from resection material of	Biotin	As per manufacturer (Illumina)	9606	RNA was hybridized to each array according to the	The arrays were scanned using standard Illumina	raw data file and column: DASL.2; 102217-46	The data was quantile normalized using	GPL14951	Pim,,French	p.french@erasmusmc.nl	+31 10 70443 33	Neurooncology	Neurology	Erasmus MC	Dr Molewaterplein 50	Rotterdam	2040CA	Netherlands	NONE	29377		GSM2881763

135	LGG_22033_X 522	GSM2881764	11.35829112	Public on Mar 19 2019	Dec 08 2017	Mar 19 2019	RNA	1	low grade glioma	Homo sapiens	gender: Female	age: 44.9911	treatment therapy: TMZ	igs: IGS-17	type.of.sugery: Partial removal	histology: AOA GrII	performance: 0	idh.status: mutated	progression free survival (pfs) event: Yes	pfs (days): 714	total RNA	Total RNA was extracted from resection material of	Biotin	As per manufacturer (Illumina)	9606	RNA was hybridized to each array according to the	The arrays were scanned using standard Illumina	raw data file and column: DASL.2; 102217-81	The data was quantile normalized using	GPL14951	Pim,,French	p.french@erasmusmc.nl	+31 10 70443 33	Neurooncology	Neurology	Erasmus MC	Dr Molewaterplein 50	Rotterdam	2040CA	Netherlands	NONE	29377		GSM2881764
136	LGG_22033_X 532	GSM2881765	10.28918424	Public on Mar 19 2019	Dec 08 2017	Mar 19 2019	RNA	1	low grade glioma	Homo sapiens	gender: Female	age: 54.026	treatment therapy: RT	igs: IGS-9	type.of.sugery: Partial removal	histology: AOD GrII	performance: 0	idh.status: mutated	progression free survival (pfs) event: Yes	pfs (days): 915	total RNA	Total RNA was extracted from resection material of	Biotin	As per manufacturer (Illumina)	9606	RNA was hybridized to each array according to the	The arrays were scanned using standard Illumina	raw data file and column: DASL.2; 102217-98	The data was quantile normalized using	GPL14951	Pim,,French	p.french@erasmusmc.nl	+31 10 70443 33	Neurooncology	Neurology	Erasmus MC	Dr Molewaterplein 50	Rotterdam	2040CA	Netherlands	NONE	29377		GSM2881765
137	LGG_22033_X 543	GSM2881766	9.512298214	Public on Mar 19 2019	Dec 08 2017	Mar 19 2019	RNA	1	low grade glioma	Homo sapiens	gender: Male	age: 40.742	treatment therapy: RT	igs: r.X9	type.of.sugery: Biopsy	histology: AA GrII	performance: 0	idh.status: mutated	progression free survival (pfs) event: No	pfs (days): 1232	total RNA	Total RNA was extracted from resection material of	Biotin	As per manufacturer (Illumina)	9606	RNA was hybridized to each array according to the	The arrays were scanned using standard Illumina	raw data file and column: DASL.2; 102217-40	The data was quantile normalized using	GPL14951	Pim,,French	p.french@erasmusmc.nl	+31 10 70443 33	Neurooncology	Neurology	Erasmus MC	Dr Molewaterplein 50	Rotterdam	2040CA	Netherlands	NONE	29377		GSM2881766
138	LGG_22033_X 544	GSM2881767	9.707989895	Public on Mar 19 2019	Dec 08 2017	Mar 19 2019	RNA	1	low grade glioma	Homo sapiens	gender: Female	age: 37.4045	treatment therapy: TMZ	igs: IGS-17	type.of.sugery: Partial removal	histology: AOA GrII	performance: 1	idh.status: mutated	progression free survival (pfs) event: No	pfs (days): 1051	total RNA	Total RNA was extracted from resection material of	Biotin	As per manufacturer (Illumina)	9606	RNA was hybridized to each array according to the	The arrays were scanned using standard Illumina	raw data file and column: DASL.5; X102413.24	The data was quantile normalized using	GPL14951	Pim,,French	p.french@erasmusmc.nl	+31 10 70443 33	Neurooncology	Neurology	Erasmus MC	Dr Molewaterplein 50	Rotterdam	2040CA	Netherlands	NONE	29377		GSM2881767
139	LGG_22033_X 550	GSM2881768	10.78042325	Public on Mar 19 2019	Dec 08 2017	Mar 19 2019	RNA	1	low grade glioma	Homo sapiens	gender: Male	age: 58.1081	treatment therapy: TMZ	igs: IGS-9	type.of.sugery: Biopsy	histology: AOD GrII	performance: 0	idh.status: mutated	progression free survival (pfs) event: No	pfs (days): 1161	total RNA	Total RNA was extracted from resection material of	Biotin	As per manufacturer (Illumina)	9606	RNA was hybridized to each array according to the	The arrays were scanned using standard Illumina	raw data file and column: DASL.3; X1022329.022	The data was quantile normalized using	GPL14951	Pim,,French	p.french@erasmusmc.nl	+31 10 70443 33	Neurooncology	Neurology	Erasmus MC	Dr Molewaterplein 50	Rotterdam	2040CA	Netherlands	NONE	29377		GSM2881768
140	LGG_22033_X 555	GSM2881769	11.16845942	Public on Mar 19 2019	Dec 08 2017	Mar 19 2019	RNA	1	low grade glioma	Homo sapiens	gender: Female	age: 42.4969	treatment therapy: RT	igs: IGS-17	type.of.sugery: Partial removal	histology: AOA GrII	performance: 0	idh.status: mutated	progression free survival (pfs) event: No	pfs (days): 1190	total RNA	Total RNA was extracted from resection material of	Biotin	As per manufacturer (Illumina)	9606	RNA was hybridized to each array according to the	The arrays were scanned using standard Illumina	raw data file and column: DASL.1; X102012.128	The data was quantile normalized using	GPL14951	Pim,,French	p.french@erasmusmc.nl	+31 10 70443 33	Neurooncology	Neurology	Erasmus MC	Dr Molewaterplein 50	Rotterdam	2040CA	Netherlands	NONE	29377		GSM2881769
141	LGG_22033_X 557	GSM2881770	9.937890712	Public on Mar 19 2019	Dec 08 2017	Mar 19 2019	RNA	1	low grade glioma	Homo sapiens	gender: Female	age: 36.7474	treatment therapy: TMZ	igs: IGS-9	type.of.sugery: Partial removal	histology: AOD GrII	performance: 0	idh.status: mutated	progression free survival (pfs) event: No	pfs (days): 753	total RNA	Total RNA was extracted from resection material of	Biotin	As per manufacturer (Illumina)	9606	RNA was hybridized to each array according to the	The arrays were scanned using standard Illumina	raw data file and column: DASL.1; X102012.096	The data was quantile normalized using	GPL14951	Pim,,French	p.french@erasmusmc.nl	+31 10 70443 33	Neurooncology	Neurology	Erasmus MC	Dr Molewaterplein 50	Rotterdam	2040CA	Netherlands	NONE	29377		GSM2881770
142	LGG_22033_X 558	GSM2881771	9.43098229	Public on Mar 19 2019	Dec 08 2017	Mar 19 2019	RNA	1	low grade glioma	Homo sapiens	gender: Male	age: 50.4695	treatment therapy: TMZ	igs: r.X9	type.of.sugery: Partial removal	histology: AOD GrII	performance: 0	idh.status: mutated	progression free survival (pfs) event: No	pfs (days): 98	total RNA	Total RNA was extracted from resection material of	Biotin	As per manufacturer (Illumina)	9606	RNA was hybridized to each array according to the	The arrays were scanned using standard Illumina	raw data file and column: DASL.4; X102380.17	The data was quantile normalized using	GPL14951	Pim,,French	p.french@erasmusmc.nl	+31 10 70443 33	Neurooncology	Neurology	Erasmus MC	Dr Molewaterplein 50	Rotterdam	2040CA	Netherlands	NONE	29377		GSM2881771
143	LGG_22033_X 56	GSM2881772	10.96754385	Public on Mar 19 2019	Dec 08 2017	Mar 19 2019	RNA	1	low grade glioma	Homo sapiens	gender: Male	age: 59.2225	treatment therapy: RT	igs: r.X17	type.of.sugery: Total removal	histology: AOD GrII	performance: 1	idh.status: normal	progression free survival (pfs) event: Yes	pfs (days): 625	total RNA	Total RNA was extracted from resection material of	Biotin	As per manufacturer (Illumina)	9606	RNA was hybridized to each array according to the	The arrays were scanned using standard Illumina	raw data file and column: DASL.4; X102380.03	The data was quantile normalized using	GPL14951	Pim,,French	p.french@erasmusmc.nl	+31 10 70443 33	Neurooncology	Neurology	Erasmus MC	Dr Molewaterplein 50	Rotterdam	2040CA	Netherlands	NONE	29377		GSM2881772
144	LGG_22033_X 562	GSM2881773	10.04134522	Public on Mar 19 2019	Dec 08 2017	Mar 19 2019	RNA	1	low grade glioma	Homo sapiens	gender: Female	age: 45.5359	treatment therapy: RT	igs: IGS-9	type.of.sugery: Partial removal	histology: AOA GrII	performance: 0	idh.status: mutated	progression free survival (pfs) event: Yes	pfs (days): 643	total RNA	Total RNA was extracted from resection material of	Biotin	As per manufacturer (Illumina)	9606	RNA was hybridized to each array according to the	The arrays were scanned using standard Illumina	raw data file and column: DASL.5; X102413.25	The data was quantile normalized using	GPL14951	Pim,,French	p.french@erasmusmc.nl	+31 10 70443 33	Neurooncology	Neurology	Erasmus MC	Dr Molewaterplein 50	Rotterdam	2040CA	Netherlands	NONE	29377		GSM2881773
145	LGG_22033_X 564	GSM2881774	9.028872233	Public on Mar 19 2019	Dec 08 2017	Mar 19 2019	RNA	1	low grade glioma	Homo sapiens	gender: Male	age: 49.1663	treatment therapy: RT	igs: IGS-17	type.of.sugery: Partial removal	histology: AA GrII	performance: 1	idh.status: mutated	progression free survival (pfs) event: No	pfs (days): 1148	total RNA	Total RNA was extracted from resection material of	Biotin	As per manufacturer (Illumina)	9606	RNA was hybridized to each array according to the	The arrays were scanned using standard Illumina	raw data file and column: DASL.5; X102413.26	The data was quantile normalized using	GPL14951	Pim,,French	p.french@erasmusmc.nl	+31 10 70443 33	Neurooncology	Neurology	Erasmus MC	Dr Molewaterplein 50	Rotterdam	2040CA	Netherlands	NONE	29377		GSM2881774
146	LGG_22033_X 566	GSM2881775	10.34974192	Public on Mar 19 2019	Dec 08 2017	Mar 19 2019	RNA	1	low grade glioma	Homo sapiens	gender: Male	age: 53.4894	treatment therapy: TMZ	igs: r.X9	type.of.sugery: Biopsy	histology: AOD GrII	performance: 0	idh.status: undetermined	progression free survival (pfs) event: Yes	pfs (days): 95	total RNA	Total RNA was extracted from resection material of	Biotin	As per manufacturer (Illumina)	9606	RNA was hybridized to each array according to the	The arrays were scanned using standard Illumina	raw data file and column: DASL.2; 102217-75	The data was quantile normalized using	GPL14951	Pim,,French	p.french@erasmusmc.nl	+31 10 70443 33	Neurooncology	Neurology	Erasmus MC	Dr Molewaterplein 50	Rotterdam	2040CA	Netherlands	NONE	29377		GSM2881775
147	LGG_22033_X 568	GSM2881776	10.21481118	Public on Mar 19 2019	Dec 08 2017	Mar 19 2019	RNA	1	low grade glioma	Homo sapiens	gender: Female	age: 33.4456	treatment therapy: TMZ	igs: IGS-17	type.of.sugery: Partial removal	histology: AA GrII	performance: 0	idh.status: mutated	progression free survival (pfs) event: Yes	pfs (days): 345	total RNA	Total RNA was extracted from resection material of	Biotin	As per manufacturer (Illumina)	9606	RNA was hybridized to each array according to the	The arrays were scanned using standard Illumina	raw data file and column: DASL.1; X102012.099	The data was quantile normalized using	GPL14951	Pim,,French	p.french@erasmusmc.nl	+31 10 70443 33	Neurooncology	Neurology	Erasmus MC	Dr Molewaterplein 50	Rotterdam	2040CA	Netherlands	NONE	29377		GSM2881776
148	LGG_22033_X 57	GSM2881777	11.09611811	Public on Mar 19 2019	Dec 08 2017	Mar 19 2019	RNA	1	low grade glioma	Homo sapiens	gender: Male	age: 40.6379	treatment therapy: RT	igs: IGS-17	type.of.sugery: Biopsy	histology: AOD GrII	performance: 0	idh.status: undetermined	progression free survival (pfs) event: Yes	pfs (days): 1246	total RNA	Total RNA was extracted from resection material of	Biotin	As per manufacturer (Illumina)	9606	RNA was hybridized to each array according to the	The arrays were scanned using standard Illumina	raw data file and column: DASL.3; X1022329.017	The data was quantile normalized using	GPL14951	Pim,,French	p.french@erasmusmc.nl	+31 10 70443 33	Neurooncology	Neurology	Erasmus MC	Dr Molewaterplein 50	Rotterdam	2040CA	Netherlands	NONE	29377		GSM2881777
149	LGG_22033_X 570	GSM2881778	10.36453678	Public on Mar 19 2019	Dec 08 2017	Mar 19 2019	RNA	1	low grade glioma	Homo sapiens	gender: Female	age: 48.5366	treatment therapy: RT	igs: r.X17	type.of.sugery: Partial removal	histology: AOA GrII	performance: 0	idh.status: mutated	progression free survival (pfs) event: No	pfs (days): 1111	total RNA	Total RNA was extracted from resection material of	Biotin	As per manufacturer (Illumina)	9606	RNA was hybridized to each array according to the	The arrays were scanned using standard Illumina	raw data file and column: DASL.2; 102217-14	The data was quantile normalized using	GPL14951	Pim,,French	p.french@erasmusmc.nl	+31 10 70443 33	Neurooncology	Neurology	Erasmus MC	Dr Molewaterplein 50	Rotterdam	2040CA	Netherlands	NONE	29377		GSM2881778
150	LGG_22033_X 572	GSM2881779	10.67136213	Public on Mar 19 2019	Dec 08 2017	Mar 19 2019	RNA	1	low grade glioma	Homo sapiens	gender: Female	age: 26.0315	treatment therapy: RT	igs: IGS-17	type.of.sugery: Biopsy	histology: AA GrII	performance: 1	idh.status: mutated	progression free survival (pfs) event: Yes	pfs (days): 497	total RNA	Total RNA was extracted from resection material of	Biotin	As per manufacturer (Illumina)	9606	RNA was hybridized to each array according to the	The arrays were scanned using standard Illumina	raw data file and column: DASL.5; X102413.27	The data was quantile normalized using	GPL14951	Pim,,French	p.french@erasmusmc.nl	+31 10 70443 33	Neurooncology	Neurology	Erasmus MC	Dr Molewaterplein 50	Rotterdam	2040CA	Netherlands	NONE	29377		GSM2881779
151	LGG_22033_X 574	GSM2881780	9.52363061	Public on Mar 19 2019	Dec 08 2017	Mar 19 2019	RNA	1	low grade glioma	Homo sapiens	gender: Female	age: 47.2526	treatment therapy: RT	igs: IGS-17	type.of.sugery: Partial removal	histology: AOA GrII	performance: 1	idh.status: mutated	progression free survival (pfs) event: No	pfs (days): 314	total RNA	Total RNA was extracted from resection material of	Biotin	As per manufacturer (Illumina)	9606	RNA was hybridized to each array according to the	The arrays were scanned using standard Illumina	raw data file and column: DASL.5; X102413.28	The data was quantile normalized using	GPL14951	Pim,,French	p.french@erasmusmc.nl	+31 10 70443 33	Neurooncology	Neurology	Erasmus MC	Dr Molewaterplein 50	Rotterdam	2040CA	Netherlands	NONE	29377		GSM2881780
152	LGG_22033_X 58	GSM2881781	11.43218052	Public on Mar 19 2019	Dec 08 2017	Mar 19 2019	RNA	1	low grade glioma	Homo sapiens	gender: Female	age: 34.9459	treatment therapy: TMZ	igs: IGS-17	type.of.sugery: Biopsy	histology: AA GrII	performance: 0	idh.status: mutated	progression free survival (pfs) event: Yes	pfs (days): 848	total RNA	Total RNA was extracted from resection material of	Biotin	As per manufacturer (Illumina)	9606	RNA was hybridized to each array according to the	The arrays were scanned using standard Illumina	raw data file and column: DASL.3; 1022329.016	The data was quantile normalized using	GPL14951	Pim,,French	p.french@erasmusmc.nl	+31 10 70443 33	Neurooncology	Neurology	Erasmus MC	Dr Molewaterplein 50	Rotterdam	2040CA	Netherlands	NONE	29377		GSM2881781
153	LGG_22033_X 580	GSM2881782	10.65312645	Public on Mar 19 2019	Dec 08 2017	Mar 19 2019	RNA	1	low grade glioma	Homo sapiens	gender: Male	age: 32.7474	treatment therapy: RT	igs: IGS-16	type.of.sugery: Biopsy	histology: AOD GrII	performance: 1	idh.status: normal	progression free survival (pfs) event: Yes	pfs (days): 181	total RNA	Total RNA was extracted from resection material of	Biotin	As per manufacturer (Illumina)	9606	RNA was hybridized to each array according to the	The arrays were scanned using standard Illumina	raw data file and column: DASL.1; X102012.122	The data was quantile normalized using	GPL14951	Pim,,French	p.french@erasmusmc.nl	+31 10 70443 33	Neurooncology	Neurology	Erasmus MC	Dr Molewaterplein 50	Rotterdam	2040CA	Netherlands	NONE	29377		GSM2881782
154	LGG_22033_X 59	GSM2881783	10.76157162	Public on Mar 19 2019	Dec 08 2017	Mar 19 2019	RNA	1	low grade glioma	Homo sapiens	gender: Female	age: 31.9562	treatment therapy: TMZ	igs: IGS-17	type.of.sugery: Total removal	histology: AOD GrII	performance: 0	idh.status: normal	progression free survival (pfs) event: Yes	pfs (days): 1252	total RNA	Total RNA was extracted from resection material of	Biotin	As per manufacturer (Illumina)	9606	RNA was hybridized to each array according to the	The arrays were scanned using standard Illumina	raw data file and column: DASL.5; X102413.03	The data was quantile normalized using	GPL14951	Pim,,French	p.french@erasmusmc.nl	+31 10 70443 33	Neurooncology	Neurology	Erasmus MC	Dr Molewaterplein 50	Rotterdam	2040CA	Netherlands	NONE	29377		GSM2881783
155	LGG_22033_X 591	GSM2881784	10.60923962	Public on Mar 19 2019	Dec 08 2017	Mar 19 2019	RNA	1	low grade glioma	Homo sapiens	gender: Male	age: 50.4559	treatment therapy: RT	igs: IGS-17	type.of.sugery: Partial removal	histology: AOA GrII	performance: 0	idh.status: mutated	progression free survival (pfs) event: No	pfs (days): 7	total RNA	Total RNA was extracted from resection material of	Biotin	As per manufacturer (Illumina)	9606	RNA was hybridized to each array according to the	The arrays were scanned using standard Illumina	raw data file and column: DASL.1; X102012.139	The data was quantile normalized using	GPL14951	Pim,,French	p.french@erasmusmc.nl	+31 10 70443 33	Neurooncology	Neurology	Erasmus MC	Dr Molewaterplein 50	Rotterdam	2040CA	Netherlands	NONE	29377		GSM2881784
156	LGG_22033_X 593	GSM2881785	10.34398513	Public on Mar 19 2019	Dec 08 2017	Mar 19 2019	RNA	1	low grade glioma	Homo sapiens	gender: Female	age: 61.1198	treatment therapy: TMZ	igs: IGS-17	type.of.sugery: Total removal	histology: AOA GrII	performance: 1	idh.status: mutated	progression free survival (pfs) event: No	pfs (days): 1113	total RNA	Total RNA was extracted from resection material of	Biotin	As per manufacturer (Illumina)	9606	RNA was hybridized to each array according to the	The arrays were scanned using standard Illumina	raw data file and column: DASL.1; X102012.130	The data was quantile normalized using	GPL14951	Pim,,French	p.french@erasmusmc.nl	+31 10 70443 33	Neurooncology	Neurology	Erasmus MC	Dr Molewaterplein 50	Rotterdam	2040CA	Netherlands	NONE	29377		GSM2881785
157	LGG_22033_X 594	GSM2881786	11.38081267	Public on Mar 19 2019	Dec 08 2017	Mar 19 2019	RNA	1	low grade glioma	Homo sapiens	gender: Female	age: 66.8665	treatment therapy: TMZ	igs: IGS-9	type.of.sugery: Partial removal	histology: AOD GrII	performance: 0	idh.status: mutated	progression free survival (pfs) event: Yes	pfs (days): 461	total RNA	Total RNA was extracted from resection material of	Biotin	As per manufacturer (Illumina)	9606	RNA was hybridized to each array according to the	The arrays were scanned using standard Illumina	raw data file and column: DASL.2; 102217-19	The data was quantile normalized using	GPL14951	Pim,,French	p.french@erasmusmc.nl	+31 10 70443 33	Neurooncology	Neurology	Erasmus MC	Dr Molewaterplein 50	Rotterdam	2040CA	Netherlands	NONE	29377		GSM2881786
158	LGG_22033_X 6	GSM2881787	9.979723645	Public on Mar 19 2019	Dec 08 2017	Mar 19 2019	RNA	1	low grade glioma	Homo sapiens	gender: Male	age: 50.3737	treatment therapy: RT	igs: IGS-17	type.of.sugery: Total removal	histology: AA GrII	performance: 2	idh.status: normal	progression free survival (pfs) event: Yes	pfs (days): 817	total RNA	Total RNA was extracted from resection material of	Biotin	As per manufacturer (Illumina)	9606	RNA was hybridized to each array according to the	The arrays were scanned using standard Illumina	raw data file and column: DASL.1; X102012.087	The data was quantile normalized using	GPL14951	Pim,,French	p.french@erasmusmc.nl	+31 10 70443 33	Neurooncology	Neurology	Erasmus MC	Dr Molewaterplein 50	Rotterdam	2040CA	Netherlands	NONE	29377		GSM2881787
159	LGG_22033_X 60	GSM2881788	10.83417232	Public on Mar 19 2019	Dec 08 2017	Mar 19 2019	RNA	1	low grade glioma	Homo sapiens	gender: Female	age: 39.0089	treatment therapy: RT	igs: IGS-17	type.of.sugery: Biopsy	histology: AOD GrII	performance: 1	idh.status: mutated	progression free survival (pfs) event: No	pfs (days): 2136	total RNA	Total RNA was extracted from resection material of	Biotin	As per manufacturer (Illumina)	9606	RNA was hybridized to each array according to the	The arrays were scanned using standard Illumina	raw data file and column: DASL.5; X102413.04	The data was quantile normalized using	GPL14951	Pim,,French	p.french@erasmusmc.nl	+31 10 70443 33	Neurooncology	Neurology	Erasmus MC	Dr Molewaterplein 50	Rotterdam	2040CA	Netherlands	NONE	29377		GSM2881788
160	LGG_22033_X 600	GSM2881789	10.5171462	Public on Mar 19 2019	Dec 08 2017	Mar 19 2019	RNA	1	low grade glioma	Homo sapiens	gender: Male	age: 33.4894	treatment therapy: TMZ	igs: r.X9	type.of.sugery: Partial removal	histology: AOD GrII	performance: 0	idh.status: mutated	progression free survival (pfs) event: Yes	pfs (days): 967	total RNA	Total RNA was extracted from resection material of	Biotin	As per manufacturer (Illumina)	9606	RNA was hybridized to each array according to the	The arrays were scanned using standard Illumina	raw data file and column: DASL.4; X102380.18	The data was quantile normalized using	GPL14951	Pim,,French	p.french@erasmusmc.nl	+31 10 70443 33	Neurooncology	Neurology	Erasmus MC	Dr Molewaterplein 50	Rotterdam	2040CA	Netherlands	NONE	29377		GSM2881789
161	LGG_22033_X 603	GSM2881790	10.25829098	Public on Mar 19 2019	Dec 08 2017	Mar 19 2019	RNA	1	low grade glioma	Homo sapiens	gender: Female	age: 40.7885	treatment therapy: RT	igs: r.X9	type.of.sugery: Partial removal	histology: AOA GrII	performance: 1	idh.status: mutated	progression free survival (pfs) event: No	pfs (days): 1142	total RNA	Total RNA was extracted from resection material of	Biotin	As per manufacturer (Illumina)	9606	RNA was hybridized to each array according to the	The arrays were scanned using standard Illumina	raw data file and column: DASL.4; X102380.19	The data was quantile normalized using	GPL14951	Pim,,French	p.french@erasmusmc.nl	+31 10 70443 33	Neurooncology	Neurology	Erasmus MC	Dr Molewaterplein 50	Rotterdam	2040CA	Netherlands	NONE	29377		GSM2881790
162	LGG_22033_X 604	GSM2881791	10.7051621	Public on Mar 19 2019	Dec 08 2017	Mar 19 2019	RNA	1	low grade glioma	Homo sapiens	gender: Male	age: 35.4661	treatment therapy: RT	igs: IGS-9	type.of.sugery: Total removal	histology: AOD GrII	performance: 0	idh.status: mutated	progression free survival (pfs) event: Yes	pfs (days): 741	total RNA	Total RNA was extracted from resection material of	Biotin	As per manufacturer (Illumina)	9606	RNA was hybridized to each array according to the	The arrays were scanned using standard Illumina	raw data file and column: DASL.1; X102012.143	The data was quantile normalized using	GPL14951	Pim,,French	p.french@erasmusmc.nl	+31 10 70443 33	Neurooncology	Neurology	Erasmus MC	Dr Molewaterplein 50	Rotterdam	2040CA	Netherlands	NONE	29377		GSM2881791
163	LGG_22033_X 612	GSM2881792	9.132357163	Public on Mar 19 2019	Dec 08 2017	Mar 19 2019	RNA	1	low grade glioma	Homo sapiens	gender: Male	age: 50.5544	treatment therapy: TMZ	igs: IGS-17	type.of.sugery: Biopsy	histology: AA GrII	performance: 1	idh.status: undetermined	progression free survival (pfs) event: Yes	pfs (days): 467	total RNA	Total RNA was extracted from resection material of	Biotin	As per manufacturer (Illumina)	9606	RNA was hybridized to each array according to the	The arrays were scanned using standard Illumina	raw data file and column: DASL.3; X1022329.007	The data was quantile normalized using	GPL14951	Pim,,French	p.french@erasmusmc.nl	+31 10 70443 33	Neurooncology	Neurology	Erasmus MC	Dr Molewaterplein 50	Rotterdam	2040CA	Netherlands	NONE	29377		GSM2881792
164	LGG_22033_X 613	GSM2881793	10.24698254	Public on Mar 19 2019	Dec 08 2017	Mar 19 2019	RNA	1	low grade glioma	Homo sapiens	gender: Male	age: 56.104	treatment therapy: RT	igs: r.X9	type.of.sugery: Partial removal	histology: AOD GrII	performance: 0	idh.status: mutated	progression free survival (pfs) event: Yes	pfs (days): 1052	total RNA	Total RNA was extracted from resection material of	Biotin	As per manufacturer (Illumina)	9606	RNA was hybridized to each array according to the	The arrays were scanned using standard Illumina	raw data file and column: DASL.4; X102380.20	The data was quantile normalized using	GPL14951	Pim,,French	p.french@erasmusmc.nl	+31 10 70443 33	Neurooncology	Neurology	Erasmus MC	Dr Molewaterplein 50	Rotterdam	2040CA	Netherlands	NONE	29377		GSM2881793
165	LGG_22033_X 615	GSM2881794	9.841694274	Public on Mar 19 2019	Dec 08 2017	Mar 19 2019	RNA	1	low grade glioma	Homo sapiens	gender: Male	age: 47.2416	treatment therapy: TMZ	igs: IGS-9	type.of.sugery: Total removal	histology: AOD GrII	performance: 1	idh.status: mutated	progression free survival (pfs) event: No	pfs (days): 1122	total RNA	Total RNA was extracted from resection material of	Biotin	As per manufacturer (Illumina)	9606	RNA was hybridized to each array according to the	The arrays were scanned using standard Illumina	raw data file and column: DASL.4; X102380.21	The data was quantile normalized using	GPL14951	Pim,,French	p.french@erasmusmc.nl	+31 10 70443 33	Neurooncology	Neurology	Erasmus MC	Dr Molewaterplein 50	Rotterdam	2040CA	Netherlands	NONE	29377		GSM2881794
166	LGG_22033_X 623	GSM2881795	10.30509774	Public on Mar 19 2019	Dec 08 2017	Mar 19 2019	RNA	1	low grade glioma	Homo sapiens	gender: Male	age: 63.5264	treatment therapy: RT	igs: IGS-9	type.of.sugery: Partial removal	histology: AA GrII	performance: 0	idh.status: mutated	progression free survival (pfs) event: No	pfs (days): 1072	total RNA	Total RNA was extracted from resection material of	Biotin	As per manufacturer (Illumina)	9606	RNA was hybridized to each array according to the	The arrays were scanned using standard Illumina	raw data file and column: DASL.1; X102012.133	The data was quantile normalized using	GPL14951	Pim,,French	p.french@erasmusmc.nl	+31 10 70443 33	Neurooncology	Neurology	Erasmus MC	Dr Molewaterplein 50	Rotterdam	2040CA	Netherlands	NONE	29377		GSM2881795
167	LGG_22033_X 628	GSM2881796	10.55161342	Public on Mar 19 2019	Dec 08 2017	Mar 19 2019	RNA	1	low grade glioma	Homo sapiens	gender: Male	age: 54.6557	treatment therapy: TMZ	igs: r.X9	type.of.sugery: Partial removal	histology: AOD GrII	performance: 1	idh.status: mutated	progression free survival (pfs) event: No	pfs (days): 1100	total RNA	Total RNA was extracted from resection material of	Biotin	As per manufacturer (Illumina)	9606	RNA was hybridized to each array according to the	The arrays were scanned using standard Illumina	raw data file and column: DASL.2; 102217-58	The data was quantile normalized using	GPL14951	Pim,,French	p.french@erasmusmc.nl	+31 10 70443 33	Neurooncology	Neurology	Erasmus MC	Dr Molewaterplein 50	Rotterdam	2040CA	Netherlands	NONE	29377		GSM2881796
168	LGG_22033_X 631	GSM2881797	9.447903251	Public on Mar 19 2019	Dec 08 2017	Mar 19 2019	RNA	1	low grade glioma	Homo sapiens	gender: Female	age: 36.5092	treatment therapy: RT	igs: IGS-17	type.of.sugery: Partial removal	histology: AOD GrII	performance: 2	idh.status: mutated	progression free survival (pfs) event: No	pfs (days): 779	total RNA	Total RNA was extracted from resection material of	Biotin	As per manufacturer (Illumina)	9606	RNA was hybridized to each array according to the	The arrays were scanned using standard Illumina	raw data file and column: DASL.4; X102380.22	The data was quantile normalized using	GPL14951	Pim,,French	p.french@erasmusmc.nl	+31 10 70443 33	Neurooncology	Neurology	Erasmus MC	Dr Molewaterplein 50	Rotterdam	2040CA	Netherlands	NONE	29377		GSM2881797

169	LGG_22033_X635	GSM2881798	10.40245489	Public on Mar 19 2019	Dec 08 2017	Mar 19 2019	RNA	1	low grade glioma	Homo sapiens	gender: Female	age: 40.4654	treatment therapy: TMZ	igs: IGS-17	type.of.sugery: Partial removal	histology: AOD GrII	performance: 0	idh.status: mutated	progression free survival (pfs) event: No	pfs (days): 978	total RNA	Total RNA was extracted from resection material of	Biotin	As per manufacturer (Illumina)	9606	RNA was hybridized to each array according to the	The arrays were scanned using standard Illumina	raw data file and column: DASL.2; 102217-95	The data was quantile normalized using	GPL14951	Pim,,French	p.french@erasmusmc.nl	+31 10 7044333	Neurooncology	Neurology	Erasmus MC	Dr Molewaterplein 50	Rotterdam	2040CA	Netherlands	NONE	29377		GSM2881798
170	LGG_22033_X64	GSM2881799	10.32844402	Public on Mar 19 2019	Dec 08 2017	Mar 19 2019	RNA	1	low grade glioma	Homo sapiens	gender: Female	age: 60.2081	treatment therapy: TMZ	igs: r.X17	type.of.sugery: Partial removal	histology: AOD GrII	performance: 2	idh.status: normal	progression free survival (pfs) event: Yes	pfs (days): 62	total RNA	Total RNA was extracted from resection material of	Biotin	As per manufacturer (Illumina)	9606	RNA was hybridized to each array according to the	The arrays were scanned using standard Illumina	raw data file and column: DASL.4; X102380.04	The data was quantile normalized using	GPL14951	Pim,,French	p.french@erasmusmc.nl	+31 10 7044333	Neurooncology	Neurology	Erasmus MC	Dr Molewaterplein 50	Rotterdam	2040CA	Netherlands	NONE	29377		GSM2881799
171	LGG_22033_X642	GSM2881800	9.484506974	Public on Mar 19 2019	Dec 08 2017	Mar 19 2019	RNA	1	low grade glioma	Homo sapiens	gender: Male	age: 46.3107	treatment therapy: RT	igs: IGS-18	type.of.sugery: Biopsy	histology: AA GrII	performance: 0	idh.status: normal	progression free survival (pfs) event: Yes	pfs (days): 291	total RNA	Total RNA was extracted from resection material of	Biotin	As per manufacturer (Illumina)	9606	RNA was hybridized to each array according to the	The arrays were scanned using standard Illumina	raw data file and column: DASL.5; X102413.29	The data was quantile normalized using	GPL14951	Pim,,French	p.french@erasmusmc.nl	+31 10 7044333	Neurooncology	Neurology	Erasmus MC	Dr Molewaterplein 50	Rotterdam	2040CA	Netherlands	NONE	29377		GSM2881800
172	LGG_22033_X645	GSM2881801	10.20623476	Public on Mar 19 2019	Dec 08 2017	Mar 19 2019	RNA	1	low grade glioma	Homo sapiens	gender: Male	age: 42.8255	treatment therapy: TMZ	igs: IGS-17	type.of.sugery: Biopsy	histology: AOA GrII	performance: 1	idh.status: mutated	progression free survival (pfs) event: No	pfs (days): 1028	total RNA	Total RNA was extracted from resection material of	Biotin	As per manufacturer (Illumina)	9606	RNA was hybridized to each array according to the	The arrays were scanned using standard Illumina	raw data file and column: DASL.5; X102413.30	The data was quantile normalized using	GPL14951	Pim,,French	p.french@erasmusmc.nl	+31 10 7044333	Neurooncology	Neurology	Erasmus MC	Dr Molewaterplein 50	Rotterdam	2040CA	Netherlands	NONE	29377		GSM2881801
173	LGG_22033_X647	GSM2881802	10.32015375	Public on Mar 19 2019	Dec 08 2017	Mar 19 2019	RNA	1	low grade glioma	Homo sapiens	gender: Male	age: 48.7392	treatment therapy: RT	igs: r.X9	type.of.sugery: Partial removal	histology: AOA GrII	performance: 1	idh.status: mutated	progression free survival (pfs) event: Yes	pfs (days): 884	total RNA	Total RNA was extracted from resection material of	Biotin	As per manufacturer (Illumina)	9606	RNA was hybridized to each array according to the	The arrays were scanned using standard Illumina	raw data file and column: DASL.3; X1022329.025	The data was quantile normalized using	GPL14951	Pim,,French	p.french@erasmusmc.nl	+31 10 7044333	Neurooncology	Neurology	Erasmus MC	Dr Molewaterplein 50	Rotterdam	2040CA	Netherlands	NONE	29377		GSM2881802
174	LGG_22033_X654	GSM2881803	9.578142572	Public on Mar 19 2019	Dec 08 2017	Mar 19 2019	RNA	1	low grade glioma	Homo sapiens	gender: Male	age: 45.5469	treatment therapy: RT	igs: r.X17	type.of.sugery: Biopsy	histology: AOD GrII	performance: 0	idh.status: mutated	progression free survival (pfs) event: No	pfs (days): 1045	total RNA	Total RNA was extracted from resection material of	Biotin	As per manufacturer (Illumina)	9606	RNA was hybridized to each array according to the	The arrays were scanned using standard Illumina	raw data file and column: DASL.5; X102413.31	The data was quantile normalized using	GPL14951	Pim,,French	p.french@erasmusmc.nl	+31 10 7044333	Neurooncology	Neurology	Erasmus MC	Dr Molewaterplein 50	Rotterdam	2040CA	Netherlands	NONE	29377		GSM2881803
175	LGG_22033_X662	GSM2881804	10.56958149	Public on Mar 19 2019	Dec 08 2017	Mar 19 2019	RNA	1	low grade glioma	Homo sapiens	gender: Female	age: 41.2731	treatment therapy: RT	igs: IGS-9	type.of.sugery: Partial removal	histology: AOD GrII	performance: 0	idh.status: mutated	progression free survival (pfs) event: Yes	pfs (days): 968	total RNA	Total RNA was extracted from resection material of	Biotin	As per manufacturer (Illumina)	9606	RNA was hybridized to each array according to the	The arrays were scanned using standard Illumina	raw data file and column: DASL.1; X102012.148	The data was quantile normalized using	GPL14951	Pim,,French	p.french@erasmusmc.nl	+31 10 7044333	Neurooncology	Neurology	Erasmus MC	Dr Molewaterplein 50	Rotterdam	2040CA	Netherlands	NONE	29377		GSM2881804
176	LGG_22033_X663	GSM2881805	11.0067965	Public on Mar 19 2019	Dec 08 2017	Mar 19 2019	RNA	1	low grade glioma	Homo sapiens	gender: Female	age: 45.8563	treatment therapy: TMZ	igs: IGS-17	type.of.sugery: Partial removal	histology: AA GrII	performance: 0	idh.status: mutated	progression free survival (pfs) event: Yes	pfs (days): 85	total RNA	Total RNA was extracted from resection material of	Biotin	As per manufacturer (Illumina)	9606	RNA was hybridized to each array according to the	The arrays were scanned using standard Illumina	raw data file and column: DASL.2; 102217-90	The data was quantile normalized using	GPL14951	Pim,,French	p.french@erasmusmc.nl	+31 10 7044333	Neurooncology	Neurology	Erasmus MC	Dr Molewaterplein 50	Rotterdam	2040CA	Netherlands	NONE	29377		GSM2881805
177	LGG_22033_X664	GSM2881806	9.44555487	Public on Mar 19 2019	Dec 08 2017	Mar 19 2019	RNA	1	low grade glioma	Homo sapiens	gender: Female	age: 28.9528	treatment therapy: RT	igs: IGS-17	type.of.sugery: Partial removal	histology: AA GrII	performance: 1	idh.status: mutated	progression free survival (pfs) event: Yes	pfs (days): 910	total RNA	Total RNA was extracted from resection material of	Biotin	As per manufacturer (Illumina)	9606	RNA was hybridized to each array according to the	The arrays were scanned using standard Illumina	raw data file and column: DASL.2; 102217-77	The data was quantile normalized using	GPL14951	Pim,,French	p.french@erasmusmc.nl	+31 10 7044333	Neurooncology	Neurology	Erasmus MC	Dr Molewaterplein 50	Rotterdam	2040CA	Netherlands	NONE	29377		GSM2881806
178	LGG_22033_X666	GSM2881807	10.68568388	Public on Mar 19 2019	Dec 08 2017	Mar 19 2019	RNA	1	low grade glioma	Homo sapiens	gender: Male	age: 55.6769	treatment therapy: TMZ	igs: IGS-17	type.of.sugery: Total removal	histology: AA GrII	performance: 0	idh.status: mutated	progression free survival (pfs) event: No	pfs (days): 235	total RNA	Total RNA was extracted from resection material of	Biotin	As per manufacturer (Illumina)	9606	RNA was hybridized to each array according to the	The arrays were scanned using standard Illumina	raw data file and column: DASL.1; X102012.157	The data was quantile normalized using	GPL14951	Pim,,French	p.french@erasmusmc.nl	+31 10 7044333	Neurooncology	Neurology	Erasmus MC	Dr Molewaterplein 50	Rotterdam	2040CA	Netherlands	NONE	29377		GSM2881807
179	LGG_22033_X667	GSM2881808	10.53500619	Public on Mar 19 2019	Dec 08 2017	Mar 19 2019	RNA	1	low grade glioma	Homo sapiens	gender: Male	age: 31.9014	treatment therapy: TMZ	igs: IGS-9	type.of.sugery: Partial removal	histology: AOA GrII	performance: 0	idh.status: mutated	progression free survival (pfs) event: Yes	pfs (days): 570	total RNA	Total RNA was extracted from resection material of	Biotin	As per manufacturer (Illumina)	9606	RNA was hybridized to each array according to the	The arrays were scanned using standard Illumina	raw data file and column: DASL.1; X102012.144	The data was quantile normalized using	GPL14951	Pim,,French	p.french@erasmusmc.nl	+31 10 7044333	Neurooncology	Neurology	Erasmus MC	Dr Molewaterplein 50	Rotterdam	2040CA	Netherlands	NONE	29377		GSM2881808
180	LGG_22033_X668	GSM2881809	8.237541406	Public on Mar 19 2019	Dec 08 2017	Mar 19 2019	RNA	1	low grade glioma	Homo sapiens	gender: Female	age: 40.2409	treatment therapy: TMZ	igs: r.X9	type.of.sugery: Total removal	histology: AOD GrII	performance: 1	idh.status: mutated	progression free survival (pfs) event: No	pfs (days): 1033	total RNA	Total RNA was extracted from resection material of	Biotin	As per manufacturer (Illumina)	9606	RNA was hybridized to each array according to the	The arrays were scanned using standard Illumina	raw data file and column: DASL.4; X102380.23	The data was quantile normalized using	GPL14951	Pim,,French	p.french@erasmusmc.nl	+31 10 7044333	Neurooncology	Neurology	Erasmus MC	Dr Molewaterplein 50	Rotterdam	2040CA	Netherlands	NONE	29377		GSM2881809
181	LGG_22033_X669	GSM2881810	9.414037415	Public on Mar 19 2019	Dec 08 2017	Mar 19 2019	RNA	1	low grade glioma	Homo sapiens	gender: Female	age: 37.024	treatment therapy: TMZ	igs: IGS-17	type.of.sugery: Partial removal	histology: AOD GrII	performance: 1	idh.status: mutated	progression free survival (pfs) event: No	pfs (days): 939	total RNA	Total RNA was extracted from resection material of	Biotin	As per manufacturer (Illumina)	9606	RNA was hybridized to each array according to the	The arrays were scanned using standard Illumina	raw data file and column: DASL.5; X102413.32	The data was quantile normalized using	GPL14951	Pim,,French	p.french@erasmusmc.nl	+31 10 7044333	Neurooncology	Neurology	Erasmus MC	Dr Molewaterplein 50	Rotterdam	2040CA	Netherlands	NONE	29377		GSM2881810
182	LGG_22033_X68	GSM2881811	11.91647106	Public on Mar 19 2019	Dec 08 2017	Mar 19 2019	RNA	1	low grade glioma	Homo sapiens	gender: Male	age: 64.8022	treatment therapy: TMZ	igs: IGS-17	type.of.sugery: Total removal	histology: AOD GrII	performance: 2	idh.status: normal	progression free survival (pfs) event: Yes	pfs (days): 175	total RNA	Total RNA was extracted from resection material of	Biotin	As per manufacturer (Illumina)	9606	RNA was hybridized to each array according to the	The arrays were scanned using standard Illumina	raw data file and column: DASL.1; X102012.074	The data was quantile normalized using	GPL14951	Pim,,French	p.french@erasmusmc.nl	+31 10 7044333	Neurooncology	Neurology	Erasmus MC	Dr Molewaterplein 50	Rotterdam	2040CA	Netherlands	NONE	29377		GSM2881811
183	LGG_22033_X682	GSM2881812	9.259509818	Public on Mar 19 2019	Dec 08 2017	Mar 19 2019	RNA	1	low grade glioma	Homo sapiens	gender: Female	age: 42.2806	treatment therapy: RT	igs: IGS-9	type.of.sugery: Partial removal	histology: AOD GrII	performance: 0	idh.status: mutated	progression free survival (pfs) event: No	pfs (days): 955	total RNA	Total RNA was extracted from resection material of	Biotin	As per manufacturer (Illumina)	9606	RNA was hybridized to each array according to the	The arrays were scanned using standard Illumina	raw data file and column: DASL.1; X102012.135	The data was quantile normalized using	GPL14951	Pim,,French	p.french@erasmusmc.nl	+31 10 7044333	Neurooncology	Neurology	Erasmus MC	Dr Molewaterplein 50	Rotterdam	2040CA	Netherlands	NONE	29377		GSM2881812
184	LGG_22033_X684	GSM2881813	10.06612894	Public on Mar 19 2019	Dec 08 2017	Mar 19 2019	RNA	1	low grade glioma	Homo sapiens	gender: Male	age: 52.115	treatment therapy: TMZ	igs: IGS-17	type.of.sugery: Total removal	histology: AOA GrII	performance: 0	idh.status: mutated	progression free survival (pfs) event: No	pfs (days): 805	total RNA	Total RNA was extracted from resection material of	Biotin	As per manufacturer (Illumina)	9606	RNA was hybridized to each array according to the	The arrays were scanned using standard Illumina	raw data file and column: DASL.1; X102012.150	The data was quantile normalized using	GPL14951	Pim,,French	p.french@erasmusmc.nl	+31 10 7044333	Neurooncology	Neurology	Erasmus MC	Dr Molewaterplein 50	Rotterdam	2040CA	Netherlands	NONE	29377		GSM2881813
185	LGG_22033_X685	GSM2881814	10.44867734	Public on Mar 19 2019	Dec 08 2017	Mar 19 2019	RNA	1	low grade glioma	Homo sapiens	gender: Female	age: 59.0144	treatment therapy: RT	igs: IGS-9	type.of.sugery: Partial removal	histology: AOD GrII	performance: 0	idh.status: mutated	progression free survival (pfs) event: No	pfs (days): 836	total RNA	Total RNA was extracted from resection material of	Biotin	As per manufacturer (Illumina)	9606	RNA was hybridized to each array according to the	The arrays were scanned using standard Illumina	raw data file and column: DASL.2; 102217-01	The data was quantile normalized using	GPL14951	Pim,,French	p.french@erasmusmc.nl	+31 10 7044333	Neurooncology	Neurology	Erasmus MC	Dr Molewaterplein 50	Rotterdam	2040CA	Netherlands	NONE	29377		GSM2881814
186	LGG_22033_X698	GSM2881815	11.1632641	Public on Mar 19 2019	Dec 08 2017	Mar 19 2019	RNA	1	low grade glioma	Homo sapiens	gender: Female	age: 37.8946	treatment therapy: TMZ	igs: IGS-9	type.of.sugery: Partial removal	histology: AOD GrII	performance: 0	idh.status: mutated	progression free survival (pfs) event: No	pfs (days): 404	total RNA	Total RNA was extracted from resection material of	Biotin	As per manufacturer (Illumina)	9606	RNA was hybridized to each array according to the	The arrays were scanned using standard Illumina	raw data file and column: DASL.1; X102012.147	The data was quantile normalized using	GPL14951	Pim,,French	p.french@erasmusmc.nl	+31 10 7044333	Neurooncology	Neurology	Erasmus MC	Dr Molewaterplein 50	Rotterdam	2040CA	Netherlands	NONE	29377		GSM2881815
187	LGG_22033_X70	GSM2881816	12.34916024	Public on Mar 19 2019	Dec 08 2017	Mar 19 2019	RNA	1	low grade glioma	Homo sapiens	gender: Female	age: 46.6557	treatment therapy: RT	igs: IGS-16	type.of.sugery: Biopsy	histology: AA GrII	performance: 0	idh.status: normal	progression free survival (pfs) event: Yes	pfs (days): 343	total RNA	Total RNA was extracted from resection material of	Biotin	As per manufacturer (Illumina)	9606	RNA was hybridized to each array according to the	The arrays were scanned using standard Illumina	raw data file and column: DASL.2; 102217-31	The data was quantile normalized using	GPL14951	Pim,,French	p.french@erasmusmc.nl	+31 10 7044333	Neurooncology	Neurology	Erasmus MC	Dr Molewaterplein 50	Rotterdam	2040CA	Netherlands	NONE	29377		GSM2881816
188	LGG_22033_X71	GSM2881817	10.92586536	Public on Mar 19 2019	Dec 08 2017	Mar 19 2019	RNA	1	low grade glioma	Homo sapiens	gender: Female	age: 30.6639	treatment therapy: TMZ	igs: IGS-17	type.of.sugery: Partial removal	histology: AOA GrII	performance: 1	idh.status: mutated	progression free survival (pfs) event: No	pfs (days): 2266	total RNA	Total RNA was extracted from resection material of	Biotin	As per manufacturer (Illumina)	9606	RNA was hybridized to each array according to the	The arrays were scanned using standard Illumina	raw data file and column: DASL.2; 102217-79	The data was quantile normalized using	GPL14951	Pim,,French	p.french@erasmusmc.nl	+31 10 7044333	Neurooncology	Neurology	Erasmus MC	Dr Molewaterplein 50	Rotterdam	2040CA	Netherlands	NONE	29377		GSM2881817
189	LGG_22033_X75	GSM2881818	9.300634777	Public on Mar 19 2019	Dec 08 2017	Mar 19 2019	RNA	1	low grade glioma	Homo sapiens	gender: Male	age: 40.6242	treatment therapy: TMZ	igs: IGS-17	type.of.sugery: Total removal	histology: AOD GrII	performance: 1	idh.status: mutated	progression free survival (pfs) event: Yes	pfs (days): 1542	total RNA	Total RNA was extracted from resection material of	Biotin	As per manufacturer (Illumina)	9606	RNA was hybridized to each array according to the	The arrays were scanned using standard Illumina	raw data file and column: DASL.1; X102012.071	The data was quantile normalized using	GPL14951	Pim,,French	p.french@erasmusmc.nl	+31 10 7044333	Neurooncology	Neurology	Erasmus MC	Dr Molewaterplein 50	Rotterdam	2040CA	Netherlands	NONE	29377		GSM2881818
190	LGG_22033_X81	GSM2881819	11.85491427	Public on Mar 19 2019	Dec 08 2017	Mar 19 2019	RNA	1	low grade glioma	Homo sapiens	gender: Male	age: 39.6824	treatment therapy: TMZ	igs: IGS-23	type.of.sugery: Partial removal	histology: AOD GrII	performance: 0	idh.status: undetermined	progression free survival (pfs) event: Yes	pfs (days): 521	total RNA	Total RNA was extracted from resection material of	Biotin	As per manufacturer (Illumina)	9606	RNA was hybridized to each array according to the	The arrays were scanned using standard Illumina	raw data file and column: DASL.5; X102413.06	The data was quantile normalized using	GPL14951	Pim,,French	p.french@erasmusmc.nl	+31 10 7044333	Neurooncology	Neurology	Erasmus MC	Dr Molewaterplein 50	Rotterdam	2040CA	Netherlands	NONE	29377		GSM2881819
191	LGG_22033_X84	GSM2881820	11.4295398	Public on Mar 19 2019	Dec 08 2017	Mar 19 2019	RNA	1	low grade glioma	Homo sapiens	gender: Male	age: 29.2868	treatment therapy: RT	igs: r.X17	type.of.sugery: Partial removal	histology: AOD GrII	performance: 2	idh.status: mutated	progression free survival (pfs) event: Yes	pfs (days): 223	total RNA	Total RNA was extracted from resection material of	Biotin	As per manufacturer (Illumina)	9606	RNA was hybridized to each array according to the	The arrays were scanned using standard Illumina	raw data file and column: DASL.1; X102012.070	The data was quantile normalized using	GPL14951	Pim,,French	p.french@erasmusmc.nl	+31 10 7044333	Neurooncology	Neurology	Erasmus MC	Dr Molewaterplein 50	Rotterdam	2040CA	Netherlands	NONE	29377		GSM2881820
192	LGG_22033_X85	GSM2881821	11.13385024	Public on Mar 19 2019	Dec 08 2017	Mar 19 2019	RNA	1	low grade glioma	Homo sapiens	gender: Male	age: 49.4045	treatment therapy: TMZ	igs: IGS-17	type.of.sugery: Partial removal	histology: AA GrII	performance: 0	idh.status: mutated	progression free survival (pfs) event: Yes	pfs (days): 1414	total RNA	Total RNA was extracted from resection material of	Biotin	As per manufacturer (Illumina)	9606	RNA was hybridized to each array according to the	The arrays were scanned using standard Illumina	raw data file and column: DASL.1; X102012.072	The data was quantile normalized using	GPL14951	Pim,,French	p.french@erasmusmc.nl	+31 10 7044333	Neurooncology	Neurology	Erasmus MC	Dr Molewaterplein 50	Rotterdam	2040CA	Netherlands	NONE	29377		GSM2881821
193	LGG_22033_X86	GSM2881822	10.11927529	Public on Mar 19 2019	Dec 08 2017	Mar 19 2019	RNA	1	low grade glioma	Homo sapiens	gender: Male	age: 53.3662	treatment therapy: TMZ	igs: IGS-9	type.of.sugery: Total removal	histology: AOD GrII	performance: 1	idh.status: mutated	progression free survival (pfs) event: Yes	pfs (days): 1232	total RNA	Total RNA was extracted from resection material of	Biotin	As per manufacturer (Illumina)	9606	RNA was hybridized to each array according to the	The arrays were scanned using standard Illumina	raw data file and column: DASL.4; X102380.05	The data was quantile normalized using	GPL14951	Pim,,French	p.french@erasmusmc.nl	+31 10 7044333	Neurooncology	Neurology	Erasmus MC	Dr Molewaterplein 50	Rotterdam	2040CA	Netherlands	NONE	29377		GSM2881822
194	LGG_22033_X92	GSM2881823	10.50839968	Public on Mar 19 2019	Dec 08 2017	Mar 19 2019	RNA	1	low grade glioma	Homo sapiens	gender: Male	age: 50.883	treatment therapy: TMZ	igs: IGS-17	type.of.sugery: Partial removal	histology: AOD GrII	performance: 0	idh.status: mutated	progression free survival (pfs) event: Yes	pfs (days): 528	total RNA	Total RNA was extracted from resection material of	Biotin	As per manufacturer (Illumina)	9606	RNA was hybridized to each array according to the	The arrays were scanned using standard Illumina	raw data file and column: DASL.2; 102217-04	The data was quantile normalized using	GPL14951	Pim,,French	p.french@erasmusmc.nl	+31 10 7044333	Neurooncology	Neurology	Erasmus MC	Dr Molewaterplein 50	Rotterdam	2040CA	Netherlands	NONE	29377		GSM2881823
195	LGG_22033_X97	GSM2881824	8.461625417	Public on Mar 19 2019	Dec 08 2017	Mar 19 2019	RNA	1	low grade glioma	Homo sapiens	gender: Female	age: 39.6112	treatment therapy: TMZ	igs: IGS-23	type.of.sugery: Partial removal	histology: AOA GrII	performance: 1	idh.status: mutated	progression free survival (pfs) event: Yes	pfs (days): 364	total RNA	Total RNA was extracted from resection material of	Biotin	As per manufacturer (Illumina)	9606	RNA was hybridized to each array according to the	The arrays were scanned using standard Illumina	raw data file and column: DASL.2; 102217-60	The data was quantile normalized using	GPL14951	Pim,,French	p.french@erasmusmc.nl	+31 10 7044333	Neurooncology	Neurology	Erasmus MC	Dr Molewaterplein 50	Rotterdam	2040CA	Netherlands	NONE	29377		GSM2881824
